# Exploring the Druggability of Conserved RNA Regulatory Elements in the SARS‐CoV‐2 Genome

**DOI:** 10.1002/anie.202103693

**Published:** 2021-08-03

**Authors:** Sridhar Sreeramulu, Christian Richter, Hannes Berg, Maria A. Wirtz Martin, Betül Ceylan, Tobias Matzel, Jennifer Adam, Nadide Altincekic, Kamal Azzaoui, Jasleen Kaur Bains, Marcel J. J. Blommers, Jan Ferner, Boris Fürtig, Michael Göbel, J. Tassilo Grün, Martin Hengesbach, Katharina F. Hohmann, Daniel Hymon, Bozana Knezic, Jason N. Martins, Klara R. Mertinkus, Anna Niesteruk, Stephen A. Peter, Dennis J. Pyper, Nusrat S. Qureshi, Ute Scheffer, Andreas Schlundt, Robbin Schnieders, Elke Stirnal, Alexey Sudakov, Alix Tröster, Jennifer Vögele, Anna Wacker, Julia E. Weigand, Julia Wirmer‐Bartoschek, Jens Wöhnert, Harald Schwalbe

**Affiliations:** ^1^ Institute for Organic Chemistry and Chemical Biology Center for Biomolecular Magnetic Resonance (BMRZ) Johann Wolfgang Goethe-University Max-von-Laue-Str. 7+9 60438 Frankfurt/M. Germany; ^2^ Institute for Molecular Biosciences Center for Biomolecular Magnetic Resonance (BMRZ) Johann Wolfgang Goethe-University Max-von-Laue-Str. 7+9 60438 Frankfurt/M. Germany; ^3^ Department of Biology Technical University of Darmstadt Schnittspahnstr. 10 64287 Darmstadt Germany; ^4^ Saverna Therapeutics Pumpmattenweg 3 4105 Biel-Benken Switzerland; ^5^ Present address: EMBL Heidelberg Meyerhofstr. 1 69117 Heidelberg Germany

**Keywords:** Covid19-nmr, fragment screening, NMR spectroscopy, RNA, SARS-CoV-2

## Abstract

SARS‐CoV‐2 contains a positive single‐stranded RNA genome of approximately 30 000 nucleotides. Within this genome, 15 RNA elements were identified as conserved between SARS‐CoV and SARS‐CoV‐2. By nuclear magnetic resonance (NMR) spectroscopy, we previously determined that these elements fold independently, in line with data from in vivo and *ex‐vivo* structural probing experiments. These elements contain non‐base‐paired regions that potentially harbor ligand‐binding pockets. Here, we performed an NMR‐based screening of a poised fragment library of 768 compounds for binding to these RNAs, employing three different ^1^H‐based 1D NMR binding assays. The screening identified common as well as RNA‐element specific hits. The results allow selection of the most promising of the 15 RNA elements as putative drug targets. Based on the identified hits, we derive key functional units and groups in ligands for effective targeting of the RNA of SARS‐CoV‐2.

## Graphical abstract

The RNA genome of SARS‐CoV‐2 contains 15 independently folded regulatory RNA elements. By NMR‐based fragment screening, the RNA target space for small molecule binding, functional mapping and inhibition is defined.

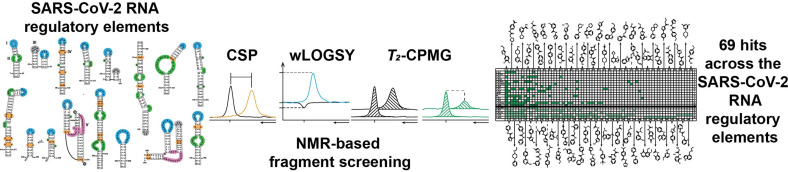

## Introduction

Since early 2020, enormous scientific efforts are geared towards antiviral treatment for SARS‐CoV‐2 (SCoV‐2). Impressive advancements are reported in vaccine development. Furthermore, experimental approaches are being actively pursued in drug repurposing and in design and synthesis of new drugs. Such studies are supported by virtual screening and molecular docking campaigns.[^1^, ^2^, ^3^, ^4^, ^5^, ^6^, ^7^] Compound libraries with more than 10^6^ molecules have been screened virtually. So far, most efforts focus on targeting proteins to inhibit viral propagation, while few attempts have been reported for direct targeting of the large viral RNA genome.^[8]^ This focus on proteins as drug targets comes despite the fact that structured RNA elements have been increasingly recognized as drug targets in recent years,[^9^, ^10^, ^11^, ^12^, ^13^, ^14^, ^15^, ^16^] including SARS‐CoV (SCoV). In SCoV, for example, the pseudoknot (PK) element involved in ribosomal frameshifting was identified as a potent drug target.^[17]^ The abundance of viral RNA in infected host cells opens a further opportunity for pharmaceutical intervention: for coronaviruses in general and for SCoV‐2 infected cells in particular, viral RNA accounts for approximately two thirds of the total RNA.^[18]^ In fact, the transcriptome of SCoV‐2 is discussed to contain numerous potential druggable sites.[^19^, ^20^] Currently, however, only few experimental screenings for ligands directly targeting the structured RNA elements in SCoV‐2 have been reported.[^8^, ^21^, ^22^]

Here, we report a holistic NMR‐based screening campaign investigating previously identified structured RNA elements.^[23]^ We determine the druggability of these regulatory RNA elements by NMR‐based screening,^[24]^ using a previously implemented workflow.^[25]^ We use a well‐characterized,^[26]^ highly diverse fragment library poised for follow‐up chemistry. The screen of 768 fragments conducted here yielded a high hit rate, despite the stringent hit definition from three independent NMR experiments, demonstrating the general druggability of the viral RNA targets.

Previously, we identified secondary structures in these RNA targets (Supporting Information, Figure S1) by NMR spectroscopy.^[23]^ Our selection of 15 SCoV‐2 RNA targets from the genome was based on functional and structural conservation among Betacoronaviruses.

In the context of screening the viral RNA targets, we briefly describe the functional roles of the 15 RNA elements. In the 5′‐genomic end, stem loop 1 (5_SL1) is associated with viral mRNA escape from Nsp1‐mediated translational shutdown.^[27]^ 5_SL2+3 harbors a stringently conserved stem‐loop (Supporting Information, Figure S1)^[28]^ and the transcription regulatory sequence regulating the production of subgenomic RNAs that is embedded within a second, more labile stem‐loop. It further comprises a nucleotide stretch involved in long‐range interactions.^[29]^ The stem‐loop 5_SL4 is indispensable for replication and contains a short upstream open reading frame (uORF).^[30]^ We dissected the large SL5 element into the three sub‐elements 5_SL5a, 5_SL5b+c and 5_SLstem, the latter of which contains the ORF1 start codon. The functions of SL6, 7 and 8 are less well defined, but their high degree of structure conservation between SCoV and SCoV‐2 suggests regulatory roles as well.

The frameshifting region is a well‐established target for binding of ligands of low molecular weight.[^17^, ^31^, ^32^] We thus screened the attenuator hairpin (att HP) and PK. Within the 3′‐UTR, a putative structural switch is supposed to play a role in the initiation events of (−)‐strand synthesis.^[33]^ The individual elements forming this elaborate RNA architecture are 3_SL1, 3_SL2 and 3_SL3base. Furthermore, the hypervariable region of the 3′‐UTR harbours the 3_s2m motif, which is recurring among a variety of RNA viruses, and seems to be involved in coronavirus pathogenicity.^[34]^ For each of these 15 RNA elements chosen for fragment‐based screening, their existence and structural integrity within the SCoV‐2 genome was demonstrated by us^[23]^ and others.[^35^, ^36^, ^37^] Correct folding prior to screening experiments was ensured as detailed in the Material and Methods section.

## Results and Discussion

We analyzed the non‐canonical RNA elements (Supporting Information, Table S1), revealing four different classes of non‐canonical segments:

- Capping loops (blue and grey in Supporting Information, Figure S1, Table S1): In general, loops are ca. 6 nts long and uridine enriched, while adenosine depleted, with respect to the genomic nucleobase composition (Supporting Information, Table S3). We here distinguish the capping loops by their different degrees of structure, with the loops of 5_SL2 and 5_SL5c adopting typical tetraloop conformations.^[28]^ A hexaloop 5′‐UUUCGU‐3′ found in 5_SL5a also caps 5_SL5b. It is interesting to note that one of the few viral mutations described since early 2020 includes a C to U mutation at position 4 in almost all SCoV‐2 strains.^[38]^ The loops of the PK, 3_s2m and especially 3_SL2 are exceptionally large (9, 9 and 13 nts, respectively). The 3_SL2 loop has been proposed to engage in a 3′‐pseudoknot structure.[^33^, ^39^]
- Bulges/internal loops (green in Supporting Information, Figure S1, Table S1). Bulges and internal loops dictate the relative orientations of helical segments. They are key elements in RNA tertiary architecture, and are often interaction sites for proteins, RNAs, metal ions or small molecules. Frequently, the helical segments of the SCoV‐2 RNA elements are interrupted either by a single unpaired nucleotide or by stretches of unpaired residues. We classify single and tandem mismatched residues separately (under iii). With this classification, the remaining internal loops are asymmetric. The average size of internal loops is ca. 7 nts, and only 5_SL6, 3_SL1 and 3_s2m contain larger internal loops. In contrast to the capping loops, the internal loops and bulges are uridine depleted and adenosine enriched with respect to the genomic base composition (Supporting Information, Table S3). Most bulges consist of only one nucleotide, but up to four bulged‐out nucleotides are found.
- Non‐canonical base‐pairs/mismatches (orange in Supporting Information, Figure S1). Next to the well‐known non‐canonical G‐U/U‐G base‐pair, which disrupts A‐helical conformation only marginally,^[40]^ the RNA elements show a variety of mismatched residues. Interestingly, tandem pyrimidine‐pyrimidine (Y‐Y) base pairs are identified in 3_SL1 and 3_SL3base by their base‐pair connectivities, whereas isolated Y‐Y mismatches in 5_SL4, 5_SL5a, 5_SL5b and 5_SL8 do not form base pairs with detectable H‐bonds.
- Three‐helix junctions (pink in Supporting Information, Figure S1). Single‐stranded regions connecting three helices are found in the PK and 3_SL3base and are structurally flexible. Helix junctions are generally considered high‐potential binding pockets for small molecules, reminiscent to bacterial riboswitches.^[41]^

Supporting Information, Table S1 gives an overview of loop and bulge sequences for the 15 RNAs. Column 3 shows the frequency of a sequence in the SCoV‐2 genome^[42]^ compared to its abundance in predicted structured regions therein.^[20]^ Column 4 correlates each sequence to the number of actual loops or bulges predicted (or, if deviating, experimentally determined^[23]^) for this sequence. To estimate the relevance of a given loop or bulge sequence, its conservation (resistance to mutation) is stated in column 5.^[43]^ Supporting Information, Table S1 also provides information on unique loop or bulge motifs within the structured RNA genome as defined by Rangan et al.^[20]^

The DSI‐poised library (DSI‐PL, Supporting Information, Table S2. DSI‐PL‐768_Ligands) is the second generation of the initially developed poised library,^[44]^ composed of 768 highly diverse and poised fragments specifically designed to facilitate easy downstream synthesis.

This library has already been successfully used to screen the main protease nsp5 from SCoV‐2.^[45]^ Ligand‐observed NMR‐based screening is a versatile method routinely adapted in the discovery of fragments binding to a target. Screening individual fragments for individual targets is, however, time‐consuming from a library with hundreds of fragments. Instead, evaluation of binding in mixtures ranging from 5 to 20 compounds is highly advantageous. One of the prerequisites in designing an ideal mixture is minimal signal overlap between the ^1^H‐NMR signals of the individual compounds so that spectral changes in the presence of the RNA target can be associated to a single compound. Previously, we performed molecular clustering analysis of the DSI‐PL fragment library. We found a total of ca. 400 distinguished chemical clusters, from which ca. 200 clusters contained singletons, suggesting a high chemical diversity of the library.^[26]^ We hypothesized that the chemical diversity of ligands might also result in significantly diverse ^1^H‐NMR spectra of the fragments in each sub‐library mix. In total, we generated 64 mixtures with 12 fragments (Supporting Information, Figure S2C) each from the 768 compounds of the DSI‐PL. Indeed, the ^1^H‐NMR spectra of each of the randomly chosen 12 fragments is significantly different from one another (Supporting Information, Figure S2A and B; black spectra), so that the signals of the individual compounds could be traced back in the ^1^H‐NMR spectrum (Supporting Information, Figure S2A and B; blue spectra).

**Figure 1:**
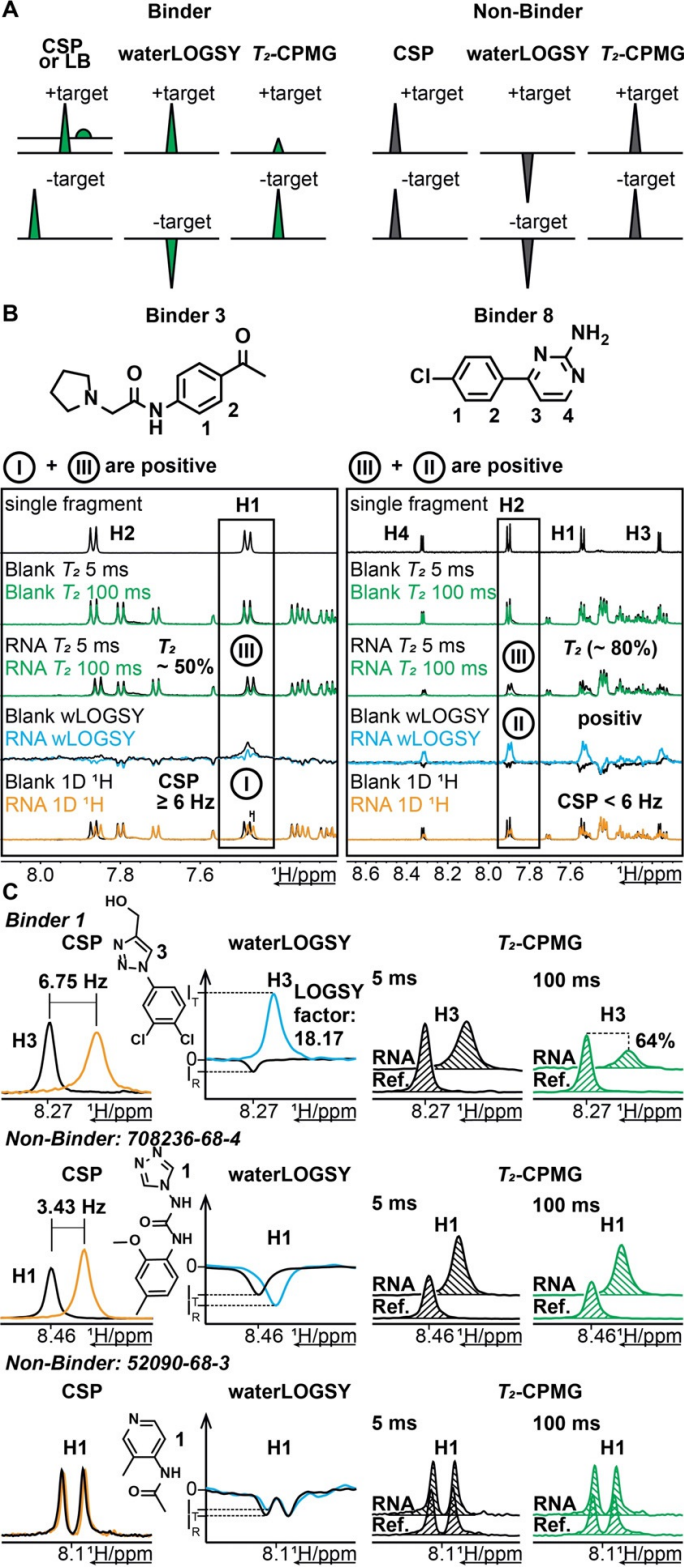
NMR‐based fragment screening and hit identification: A, Schematic representation of the experiments and criteria used to identify binders/ non‐binders. B, Chemical structures of Binder 3 and Binder 8 along with an overlay of their corresponding NMR spectra [1D ^1^H (i), waterLOGSY (ii) and *T*_2_‐CPMG (iii); (5 ms and 100 ms)] of the mixture in absence (blank) and presence of RNA. The top 1D ^1^H‐spectrum corresponds to the single fragment used for chemical shift deconvolution in the mixture. The inset highlights the signal corresponding to the hit used for analysis. Visual inspection of the overlay indicates that Binder 3 displays CSP (≥6 Hz) and *T*_2_ reduction of ca. 50 %. Binder 8 shows no CSP, but a clear sign change in waterLOGSY and *T*_2_‐reduction of ca. 80 %. C, 1D ^1^H‐NMR spectral regions of fragments with strong (top row), weak (middle row) or no binding (lower row) to 3_SL2. Binder 1 shows a CSP of 6.75 Hz, a positive waterLOGSY signal and a *T*_2_‐reduction of 64 % suggesting that it binds to 3_SL2 (top row). 708236–68‐4 shows a minor CSP of 3.43 Hz, but neither a positive waterLOGSY signal nor a *T*_2_‐reduction, indicating either very weak or no binding to 3_SL2 (middle row). 52090–68‐3 does not bind to 3_SL2 (lower row of the mixture (Supporting Information, Figure S2, blue spectrum).

We applied ligand‐observed ^1^H‐NMR experiments against the 15 RNAs ranging in size from 29–90 nts depicted in Supporting Information, Figure S1 and five additional larger RNAs with sizes between 118–472 nts that comprise several RNA elements (Supporting Information, Table S1 and Files “1–21_Hits” detailing all experimental hits). In screening experiments, changes in the ^1^H signals of the ligand in the presence and absence of the RNA served as readout for binding. After optimization (Supporting Information, Figure S3), we recorded ^1^H 1D spectra at *T*=293 K to detect chemical shift perturbations (CSPs) or line broadening (LB) (Figure 1 A), waterLOGSY[^46^, ^47^] and *T*_2_‐relaxation^[48]^ experiments (Supporting Information, Table S4). Previously, these experiments have successfully been utilized to identify small molecule binders for RNA.[^49^, ^50^] NMR signals of each of the 64 fragment mixtures were monitored at a ratio of each ligand to one RNA target of 20:1. 190 μl of a 10 μM RNA in screening buffer (25 mM KPi, 50 mM KCl, pH 6.2) was manually pipetted into 3 mm NMR tubes. 10 μl of the fragment mixture was added using a pipetting robot to a final concentration of 200 μM for each fragment. In waterLOGSY, the signal from a bound fragment is positive while the signal of a free ligand is negative. *T*_2_‐relaxation‐based experiments take advantage of the fact that large biomolecular targets including all RNAs studied here relax faster than small fragments (Figure 1 A).

For identifying binders within the mixtures, we first compared spectra from all three of the above experiments and analysed differences by visual inspection (Figure 1 B). As stringent criteria, CSPs ≥6 Hz or severe line broadening, sign change in the waterLOGSY or ≥20 % decrease of signal intensity in the *T*_2_‐relaxation experiment was used to identify binders. Ligands were assigned as a hit if two of the three criteria were satisfied. For example, binder **3** was defined as a hit by a CSP ≥6 Hz, reduced signal intensity of *T*_2_ (ca. 50 %), while the waterLOGSY effect cannot unambiguously identified (Figure 1 B, left). Similarly, binder **8** qualifies as a hit, showing changes in waterLOGSY and *T*_2_, but not CSP (Figure 1 B, right). In the second stage of analysis, for the hits thus identified, we quantified CSP, LOGSY effect[^51^, ^52^] and *T*_2_‐reduction (Q^bind^) in intensity.^[25]^

**Figure 2:**
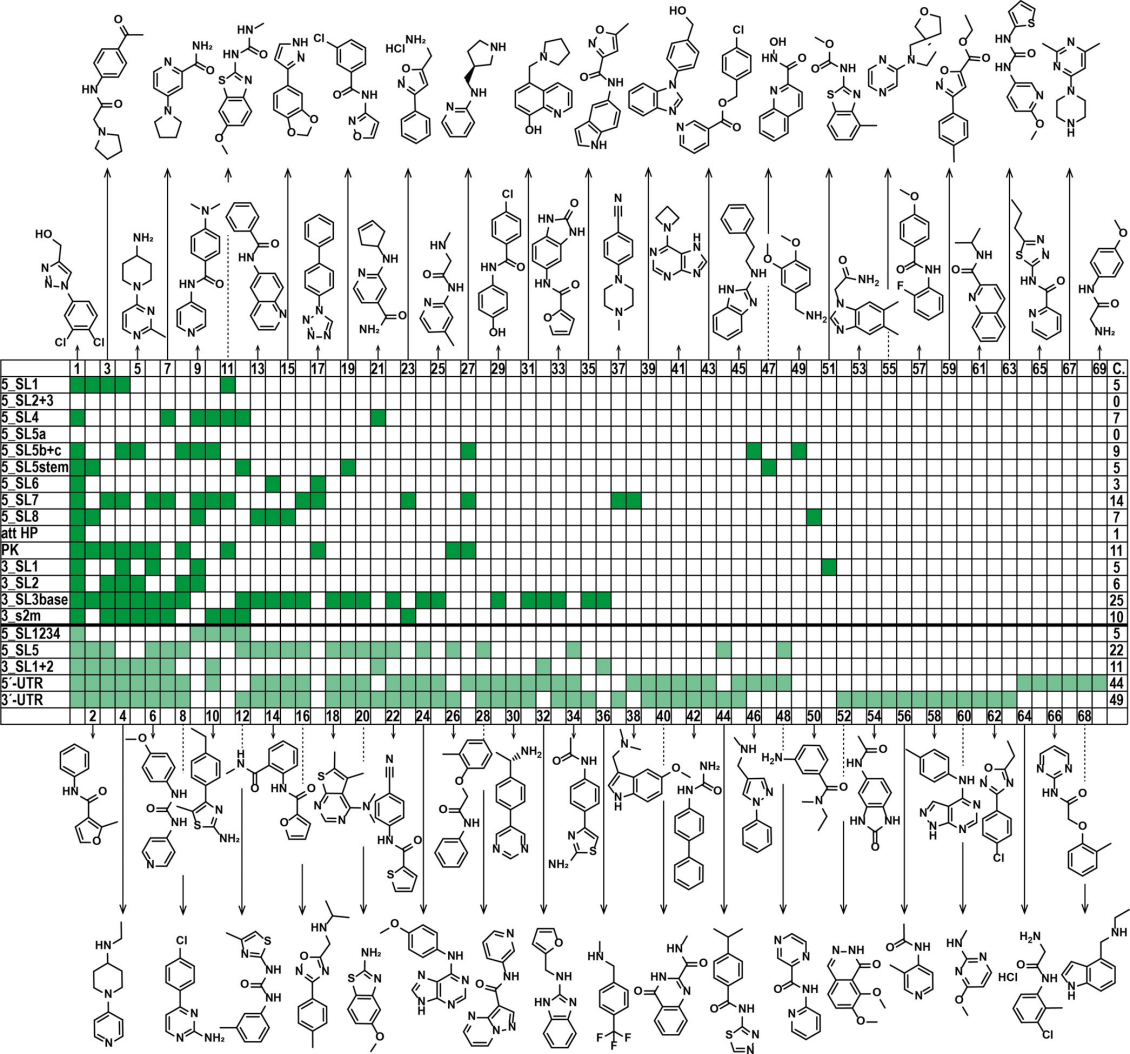
NMR‐based fragment screening identifies 69 fragment hits across the SCoV‐2 RNAs. Tabular column summarizing screened SCoV‐2 RNA elements and their corresponding hits. Dark green indicates hits for individual RNA elements; light green indicates hits for larger RNAs. The last column (C.) lists the number of binders to the investigated RNA target. Chemical structures of respective fragment hits are shown.

Previously, several reports attempted to delineate common scaffolds binding to RNA.[^9^, ^53^, ^54^] Our results here show that 40 different fragments are found to bind to the 15 RNA elements and an additional 29 fragments bind to the five multi‐element RNAs. In total, we observed 108 binding events. For 5_SL2+3 and 5_SL5a, no fragment binding was detected. For three RNAs, we identified hits that only bind to this specific RNA element (5_SL5b+c, 5_SL8, 3_SL1). Furthermore, we identified 48 fragments that recognize more than one target RNA. The range of binding promiscuity ranges from binding of two RNA targets (ligands 35–48, Figure 2) up to 18 out of the 20 screened RNA targets (ligand 1, Figure 2).

We observed no clear correlation between the number of non‐helical residues (those forming loops, bulges and mismatches) in an RNA and the number of hits, although the non‐targeted RNAs 5_SL2+3 and 5_SL5a belong to the smallest RNAs investigated. Strikingly, 5_SL7 appears to harbor little non‐helical structure, but shows 14 hits. By contrast, 3_SL1 contains roughly three times as much non‐helical space and has only four hits. However, the RNA elements with the most complex structures, 3_SL3base and PK, are also among those with the highest hit rates (Supporting Information, Table S5).

Inspection of all hits reveals the most significant molecular descriptors distinguishing between binders and non‐binders are the number of aromatic rings and the sp^3^‐character of the compounds. In general, binders have one to three aromatic rings and less sp^3^‐carbon atoms than non‐binders (see Supporting Information, Table S6).

**Scheme 1:**
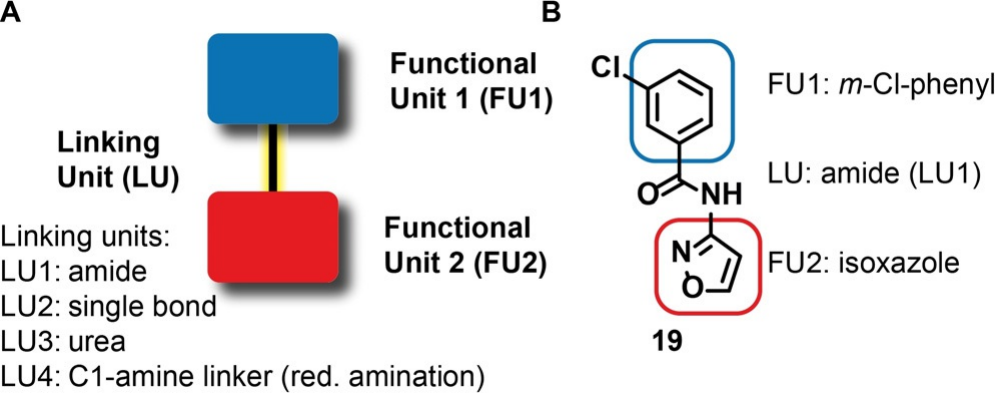
Molecular descriptors and chemical diversity of RNA binders in the DSI‐poised library. About 75 % of the fragments showing binding to SCoV‐2 RNA contain two functional units, connected by four different linking units. In case of LU3, the urea functional group likely contributes to binding affinity.

We noticed that 30 out of 40 fragments have a modular architecture: They consist of two functional units, which are mainly non‐, mono‐ or di‐substituted rings of the size 5 and 6 (Scheme 1) and connected by four different types of linker units. The mostly aromatic or heteroaromatic core motifs are often modified with a small set of substituents, which are key elements for generating affinity towards the RNA target. The remaining 10 fragments contain a single functional unit (class 1), often substituted with a functional group. Analysis of the additional 29 hits for which binding is detected only to the larger RNA elements are given in the Supporting Information, Figure S5.

**Scheme 2:**
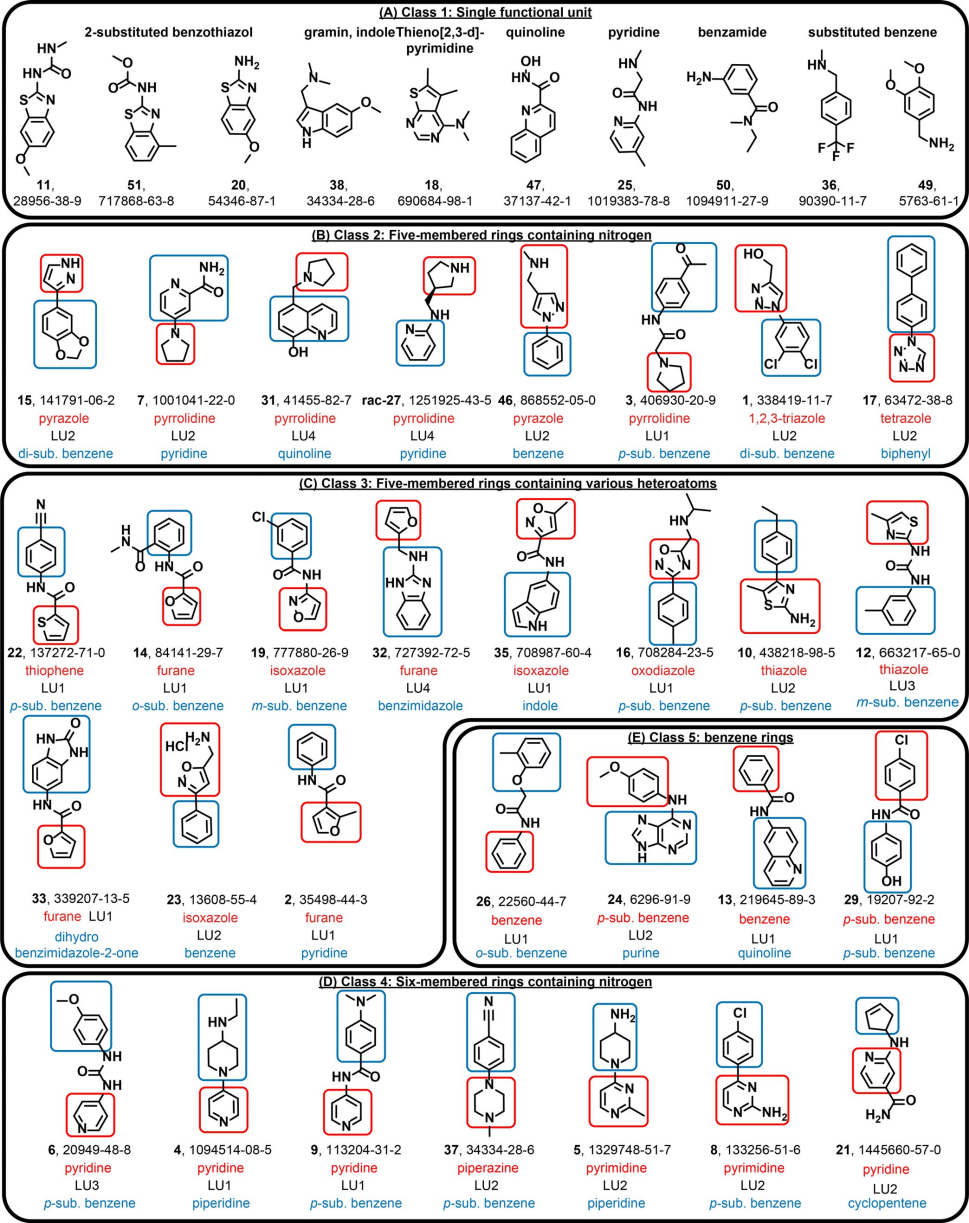
Classification into five classes of the 40 ligands that bind to the 15 regulatory RNAs. LU=linking unit.

Within the functional units, six functional groups are frequently found (Schemes 1 and 2). In the majority of hits, the two functional units are connected either by an amide bond or by a single bond. We further find urea and carbamates as linkers. They contain hydrogen bond donors and acceptors. Additionally, functional groups are often linked by a C1‐linker resulting from reductive amination. Furthermore, similar simultaneous donor and acceptor properties are found in the six‐membered (pyridine‐acetamide, pyrimidine‐2‐amine) as well as five membered heterocyclic aromatic rings (2‐amine‐thiazole, 2‐amine‐imidazole). Primary, secondary and tertiary amine groups are often found and their categorization into functional group or linker unit is somewhat arbitrary. Some of these amines have p*K*_a_ values that allow for a change in protonation state under physiological conditions, such as imidazole with a p*K*_a_ of 6.95. The classification of the fragments into functional units (see Scheme 2) leads to 26 different functional units (Supporting Information, Figure S6).

**Scheme 3:**
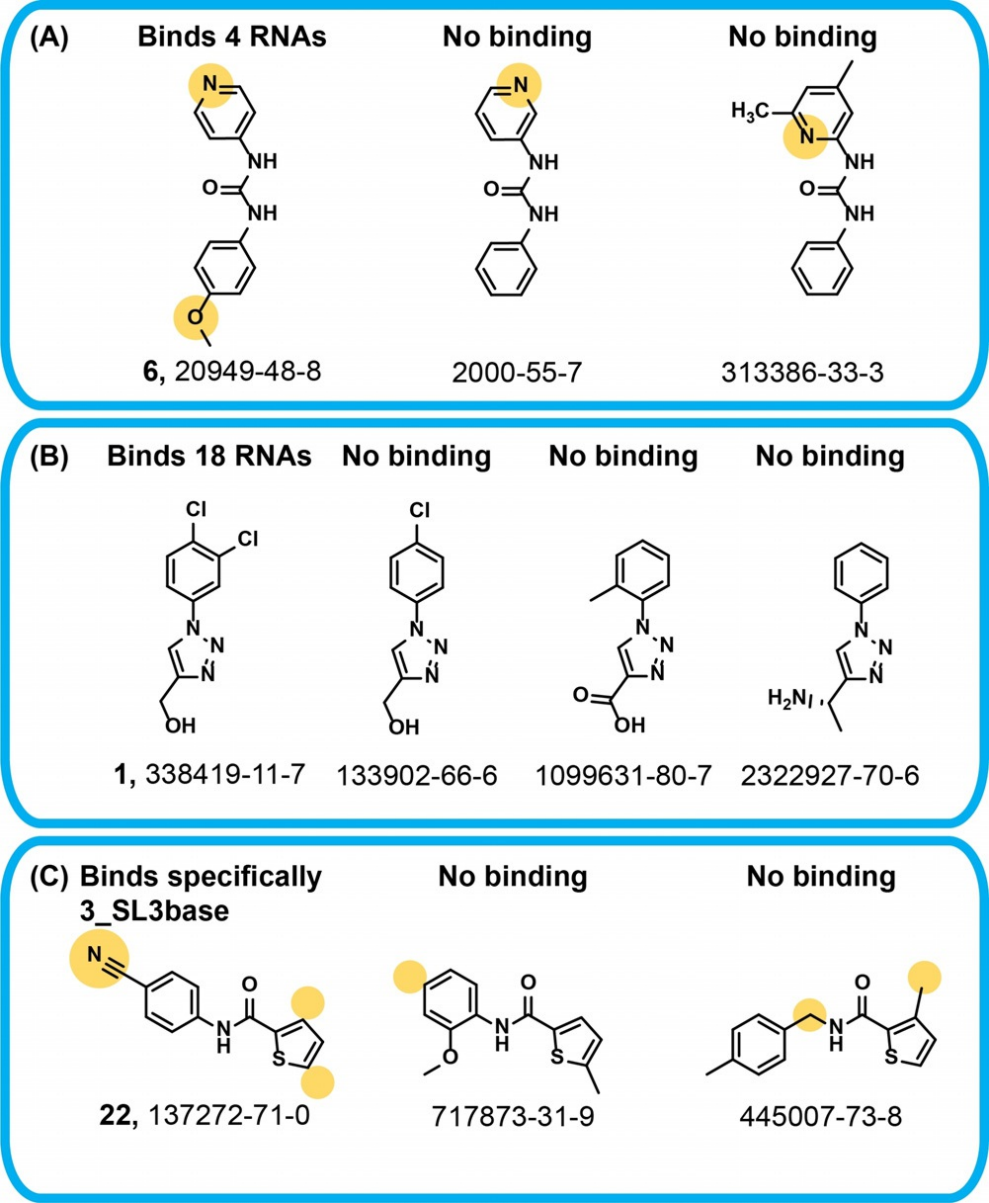
Comparison of binding and non‐binding fragments with closely related chemical structure.

Next to the analysis of binders, comparison to functionally closely related fragments that do not bind is insightful (Scheme 3). Neither fragments containing carboxylic (842971‐05‐5, for compound identity, Supporting Information, Table S2. DSI‐PL‐768_Ligands) nor benzoic acid (3303‐18‐2, 1152510‐62‐7, 693776‐70‐4) functional groups, nor sulfone (24092‐75‐9) or sulfamide (1423029‐76‐8, 1388691‐56‐2), nor fragments with hydroxyl groups (74548‐62‐2, 1849283‐80‐2), and none of the 17 acetamides within DSI‐PL are found among the hits. The library contains not only the para‐substituted pyridine derivative 6, but also ortho‐ and meta‐substituted pyridines 2000 55‐7 and 313386‐33‐3 (Scheme 3 A), but only para‐substituted pyridines bind. Only thiophene‐containing fragment 22, but not methyl‐substituted thiophenes (717873‐31‐9 and 445007‐73‐8 shows binding to three RNA targets. A particularly intriguing example of surprisingly high selectivity for binding over not binding are four triazole‐containing ligands (Scheme 3 B). While triazole 1 is the most promiscuous binder, the closely related ligands 133902‐66‐6, 1099631‐80‐7, and 2322927‐70‐6 do not bind at all.

Hits containing two functional units can be classified into four additional classes. Class 2: five‐membered heterocyclic rings containing a single nitrogen heteroatom, Class 3: five‐membered rings with several heteroatoms, in particular nitrogen, oxygen and sulfur, Class 4: six‐membered heterocyclic rings containing nitrogen atom(s), and Class 5: substituted and unsubstituted benzenes. In class 1, we find three differently 2‐substituted benzo‐thiazoles with either an amino group (compound **20**) or two different linker units attached (methyl urea linker (**11**) and methyl carbamate (**51**)). Related to the benzothiazoles are the fragment hits thieno[2,3‐*d*]pyrimidine (18) and the C7‐methoxy‐substituted gramin (**37**). A last single functional unit‐containing fragment is quinoline, substituted at C2 with a hydroxamic acid functional group (**47**). Furthermore, we find two methylene‐amino‐substituted benzenes (**36** and **49**).

**Figure 3:**
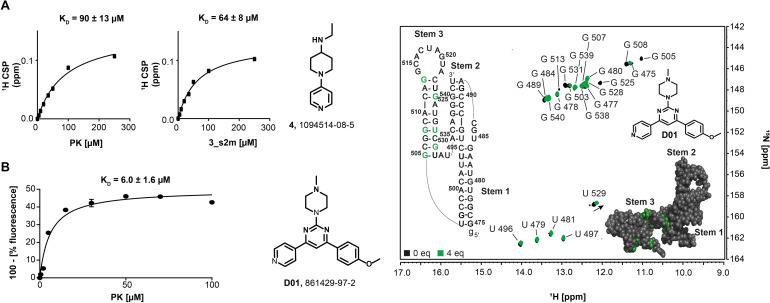
Affinities of binders and mapping of binding sites in SCoV‐2 RNAs. A) NMR‐based (ligand‐observed; protons chosen to follow CSP are indicated) titration curves for the interaction of fragment **4** with PK and 3_s2m. B) Fluorescence‐based titration curve and dissociation constant of **D01** with PK. C) Interaction of **D01** with PK monitored by RNA‐observed NMR. ^1^H, ^15^ N‐HSQC overlay of PK alone and in presence of four equivalents of **D01** (green). Addition of **D01** to PK results in CSPs or line broadening beyond detection. Affected nucleotides are mapped onto the PK secondary and 3D structures (PDB:7O7Z^[61]^).

For a subset of hits (Table 1) that bound PK and 3_s2m, we determined the dissociations constants using ligand‐observed ^1^H NMR‐based titrations. In general, the determined dissociation constants *K*_D_ for the fragment hits ranged between 64 and 1318 μM (Table 1 and Supporting Information, Figure S7). Fragment 4 bound with highest affinity both towards PK and 3_s2m (Figure 3 A). In addition to the quantification of the *T*_2_‐reduction obtained from the primary screen in mixtures at 20‐fold excess of ligand over RNA ([RNA]=10 μM) (Supporting Information, Figure S4), we determined the transverse relaxation time *T*_2_ for the binding ligands at 100 μM concentration at an RNA concentration of 35 μM. Within the subset of the fragment hits, the ligands with the highest degree of *T*_2_‐reduction also showed the strongest affinity. For ligands with a *K*_D_ <100 μM, binding affinity correlated with the *T*_2_‐reduction (Q^bind^). No correlation was, however, observed for *K*_D_ >100 μM. This finding suggests that *T*_2_ is a qualitative indicator for binding strength of fragments to one specific target for binding in fast exchange and with a low micromolar *K*_D_.

**Table 1:** Affinities and *T*_2_ relaxation times of the RNA binders.

ID	PK			3_s2 m		
	*T*_2_ [Q^bind^] primary screen^[a]^	*T*_2_ [ms] determined^[b]^	*K*_D_ [μM]^[c]^	*T*_2_ [Q^bind^] primary screen^[a]^	*T*_2_ [ms] determined^[b]^	*K*_D_ [μM]^[c]^
4	54	83±6	90±13	48	125±12	64±8
5	38	68±12	160±6	15	98±15	206±5
3	39	84±6	336±18	39	106±7	212±30
23	n.a.	n.a.	n.a.	26	131±10	678±25
2	31	87	456±33	n.a.	n.a.	n.a.
D01	n.a.	n.a.	6±1.6	n.a.	n.a.	4.4±2.3

[a] Primary screen; ligand in mixtures ([RNA]:[Ligand]=1:20), [RNA]=10 μM. [b] Single ligand measurements ([RNA]:[Ligand]=1:3), [RNA]=35 μM. [c] ^1^H NMR‐based ligand observed titrations [Ligand 100 μM; 0 to 250 μM].

As a follow‐up and in the quest for finding molecules with improved affinity, we used the fragment hits obtained for five RNAs (3_s2m, 3_SL1, 5_SL4, PK and 5′‐UTR) to identify commercially available potential binders and obtained one compound (D01) that bound to both PK and 3_s2m in single‐digit μM affinity (Figure 3 B). At the same time, D01 showed no saturable binding towards control RNAs including the most stable RNA UUCG‐tetraloop (Supporting Information, Figure S8). For fragments binding with *K*_D_ above 200 μM, it is our experience[^55^, ^56^] that ligand binding sites can often not be detected even at maximum possible excess of ligand (limited by solubility) over RNA for a number of reasons. In fact, we show this lack of binding site detection also for one of the fragments that binds with a *K*_D_ of 46 μM (fragment 8, 133256‐51‐6) in the Supporting Information, Figure S9A. By contrast, for the high‐affinity binder D01, we provide experimental mapping of its binding site to PK (Figure 3), which is in line with the binding site determined by in‐line probing (Supporting Information, Figure S9C). NMR titration experiments were performed using ^1^H, ^15^ N/^13^C ‐HSQC of PK. In these experiments, many of the signals showed either severe line‐broadening or CSPs strongly indicating binding. Mapping of these signals onto the cryo‐electron microscopy structure of PK shows that D01 mainly binds to the stem 3 (Figure 3 C and Supporting Information, Figure S9B).

## Conclusion

We conducted here an NMR‐based fragment screening against all regulatory RNA elements of the viral genome of SCoV‐2. Our results show that the fragments of the DSI‐PL library can cover almost the entire structural space of the viral RNA genome. Thus, the SCoV‐2‐RNA genome can be targeted by ligands of low molecular weight. We detect that several fragments already show specificity at this very first stage of drug development (Supporting Information, Figure S6). Assuming that increasing structural complexity enhances the potential to achieve specificity for small molecules,[^32^, ^57^] among the 15 RNAs, PK and 3_SL3base represent the most promising targets for follow‐up development of lead molecules.

The current state‐of‐the‐art discovery of small molecules targeting RNAs (RNA drug discovery) and the current status of this field have been described by Juru and Hargrove,^[53]^ by Meyer et al^[9]^ and by Warner et al.^[57]^ The screening campaign conducted here supports previous reports that identified the existence of a privileged chemical space for targeting RNA.^[58]^ Delineating RNA target space into secondary structure motifs, definable from studies of isolated RNA elements, finds further support by a recent paper from the Das group that provides FARFAR models for all conserved viral RNA elements.^[59]^

Here, we use a fragment library that has previously been used to screen proteins,^[44]^ but also 14 different RNAs, and five DNAs.^[55]^ We use NMR as primary screen because it not only provides information on chemical purity of both ligand and RNA target, but also on affinity and binding site. Identifying RNA fragments with affinities between 60–400 μM allows us to identify known and commercially available binders containing similar structural motifs. A thus identified binder, D01, binds with 6 μM affinity to PK and 3_s2m, allowing fragment linking approaches.[^32^, ^60^]

It is apparent that all ligands contain at least one, but most often two aromatic or heteroaromatic rings (Supporting Information, Figure S6). Heteroaromatic rings with two condensed rings including indoles, purines, quinolones (class I, Scheme 2) are found but not particularly enriched. Many of the hits do not have large planar moieties nor very basic sites. For example, **1** as tertiary amine is strongly basic, 2‐aminopyridine has a p*K*_a_ of around 3.5. By contrast, 1,2,3 triazole is already not very basic but shows very specific binding (Scheme 3). Further, comprehensively comparing the distribution of functional units between the hits and the non‐hits across the library suggests that the hits are enriched with pyrimidines and benzimdazoles (Supporting Information, Table S7). Thus, the absence of trivial functional units in the DSI‐PL appears particularly striking: fusion of monocyclic aromatic or heteroaromatic ring systems with H‐bond donors or ‐acceptors seems sufficient to reach binding.

The future analysis of our screening results might open new routes towards binding selectivity. The screening campaign was conducted for 20 different RNA elements. It also included RNAs that combined two or more of the individually screened elements (e.g. 5_SL1234) and even the entire 5′‐ and 3′‐untranslated regions of SCoV‐2 comprising more than 337 nts for each of the constructs. The size range of these targets is markedly different. At this point, exact quantification of the NMR screening experiments relies on approximations including effective overall rotational correlation times of the RNA targets, relative population of free vs. bound ligand, and their on‐ and off‐rates. These assumptions are, however, no longer fulfilled when one compares pools of RNAs with significantly different molecular weight and potential anisotropic tumbling. Within a given target, however, the *T*_2_‐reduction recorded within the NMR screening of all 768 fragments provides a qualitative indication for binding with *K*_D_ values below 100 μM (Table 1).

We analyzed the investigated RNA target space towards sequence‐derived properties and derived unique target sites within the SCoV‐2 genome that are conserved among coronaviruses. Exceptionally rare mutations in SCoV‐2 during 2020 have maintained the target space for ligands up to now. By analysis of the experimental hits, we can derive privileged RNA target space from a medicinal chemistry perspective. Further, our approach relies on the hypothesis that the viral genome can be specifically targeted even in the presence of human cellular RNA. The herein provided analysis of secondary structure abundance (Supporting Information, Table S1) is restricted to the viral RNA genome and will have to be extended to the entire cell‐specific human transcriptome. However, given all these theoretical considerations it is striking to recognize that small molecules have been developed that target splicing[^11^, ^56^] in a highly specific manner.

The results as well as the methodological approach presented here will impact medicinal chemistry approaches but also cellular targeting of SCoV‐2 RNA. For example, as immediate follow‐up, we currently conduct secondary NMR screens with commercially available compounds using the identified ligands as guide. We consider our broad, systematic and coherent identification of binders to be a significant step forward. The focus of our study is to experimentally validate the druggability of the SCoV‐2 genome. In fact, this potential had so far only been predicted from theoretical studies.^[59]^ Our efforts extend on‐going studies targeting the viral proteome. In conclusion, we establish here the conserved RNA elements as potential space for small molecule targeting towards Coronavirus‐specific medication, even beyond SCoV‐2.

## Conflict of interest

The authors declare no conflict of interest.

## Supporting information

As a service to our authors and readers, this journal provides supporting information supplied by the authors. Such materials are peer reviewed and may be re‐organized for online delivery, but are not copy‐edited or typeset. Technical support issues arising from supporting information (other than missing files) should be addressed to the authors.

Supporting Information

Supporting Information

Supporting Information
