# Supplementary material for: Exploring the Druggability of Conserved RNA Regulatory Elements in the SARS‐CoV‐2 Genome
Source: Angew Chem Int Ed Engl. 2021 Aug 3;60(35):19191–200. doi: 10.1002/anie.202103693 (PMC8426693; doi:10.1002/anie.202103693)
Supplement: Supplementary file 2 — Supporting Information [file ANIE-60-19191-s003.pdf]

Angewandte Chemie
Eine Zeitschrift der Gesellschaft Deutscher Chemiker

Supporting Information

Exploring the Druggability of Conserved RNA Regulatory Elements in the SARS-CoV-2 Genome

*Sridhar Sreeramulu[+], Christian Richter[+], Hannes Berg, Maria A. Wirtz Martin, Betül Ceylan, Tobias Matzel, Jennifer Adam, Nadide Altincekic, Kamal Azzaoui, Jasleen Kaur Bains, Marcel J. J. Blommers, Jan Ferner, Boris Fürtig, Michael Göbel, J. Tassilo Grün, Martin Hengesbach, Katharina F. Hohmann, Daniel Hymon, Bozana Knezic, Jason N. Martins, Klara R. Mertinkus, Anna Niesteruk, Stephen A. Peter, Dennis J. Pyper, Nusrat S. Qureshi, Ute Scheffer, Andreas Schlundt, Robbin Schnieders, Elke Stirnal, Alexey Sudakov, Alix Tröster, Jennifer Vögele, Anna Wacker, Julia E. Weigand, Julia Wirmer-Bartoschek, Jens Wöhnert, and Harald Schwalbe**

anie_202103693_sm_miscellaneous_information.pdf
anie_202103693_sm_xlsx_Dateien.zip

Suppl Table 2: Details of the 768 compounds from the DSI-Poised fragment Library (DSI-PL)

Sl. No.	Structures	No. in Manuscript	CAS Registry Number	"ID"	1H NMR Data Repository BMRB ID	ALATIS InChI
**1**	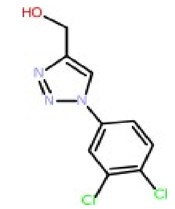	1	338419-11-7	Z1891776064	bmse011464	InChI=1S/C9H7Cl2N3O/c10-8-2-1-7(3-9(8)11)14-4-6(5-15)12-13-14/h1-4,15H,5H2
**2**	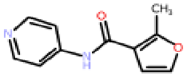	2	35498-44-3	Z136583524	bmse011087	InChI=1S/C11H10N2O2/c1-8-10(4-7-15-8)11(14)13-9-2-5-12-6-3-9/h2-7H,1H3,(H,12,13,14)
**3**	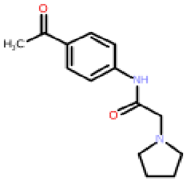	3	406930-20-9	Z2856434916	bmse011371	InChI=1S/C14H18N2O2/c1-11(17)12-4-6-13(7-5-12)15-14(18)10-16-8-2-3-9-16/h4-7H,2-3,8-10H2,1H3,(H,15,18)
**4**	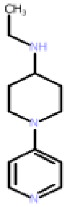	4	1094514-08-5	Z2065616520	bmse011605	InChI=1S/C12H19N3/c1-2-14-11-5-9-15(10-6-11)12-3-7-13-8-4-12/h3-4,7-8,11,14H,2,5-6,9-10H2,1H3
**5**	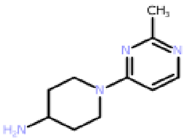	5	1329748-51-7	Z1333043417	bmse011525	InChI=1S/C10H16N4/c1-8-12-5-2-10(13-8)14-6-3-9(11)4-7-14/h2,5,9H,3-4,6-7,11H2,1H3

Suppl Table 2: Details of the 768 compounds from the DSI-Poised fragment Library (DSI-PL)

Sl. No.	Structures	No. in Manuscript	CAS Registry Number	"ID"	1H NMR Data Repository BMRB ID	ALATIS InChI
**6**	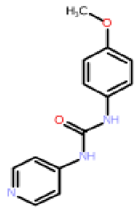	6	20949-48-8	Z321318226	bmse011713	InChI=1S/C13H13N3O2/c1-18-12-4-2-10(3-5-12)15-13(17)16-11-6-8-14-9-7-11/h2-9H,1H3,(H2,14,15,16,17)
**7**	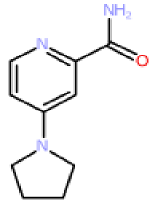	7	1001041-22-0	Z145119960	bmse011137	InChI=1S/C10H13N3O/c11-10(14)9-7-8(3-4-12-9)13-5-1-2-6-13/h3-4,7H,1-2,5-6H2,(H2,11,14)
**8**	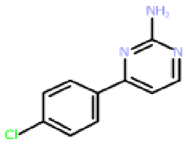	8	133256-51-6	Z363993198	bmse011744	InChI=1S/C10H8ClN3/c11-8-3-1-7(2-4-8)9-5-6-13-10(12)14-9/h1-6H,(H2,12,13,14)
**9**	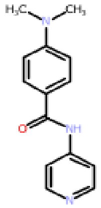	9	113204-31-2	Z85517292	bmse011180	InChI=1S/C14H15N3O/c1-17(2)13-5-3-11(4-6-13)14(18)16-12-7-9-15-10-8-12/h3-10H,1-2H3,(H,15,16,18)
**10**	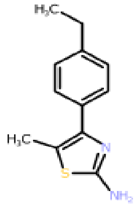	10	438218-98-5	Z48847639	bmse011448	InChI=1S/C12H14N2S/c1-3-9-4-6-10(7-5-9)11-8(2)15-12(13)14-11/h4-7H,3H2,1-2H3,(H2,13,14)

Suppl Table 2: Details of the 768 compounds from the DSI-Poised fragment Library (DSI-PL)

Sl. No.	Structures	No. in Manuscript	CAS Registry Number	"ID"	^{1}H NMR Data Repository BMRB ID	ALATIS InChI
**11**	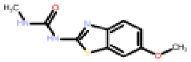	11	28956-38-9	Z371866204	bmse011187	InChI=1S/C10H11N3O2S/c1-11-9(14)13-10-12-7-4-3-6(15-2)5-8(7)16-10/h3-5H,1-2H3,(H2,11,12,13,14)
**12**	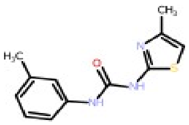	12	663217-65-0	Z44602971	bmse011185	InChI=1S/C12H13N3OS/c1-8-4-3-5-10(6-8)14-11(16)15-12-13-9(2)7-17-12/h3-7H,1-2H3,(H2,13,14,15,16)
**13**	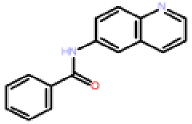	13	219645-89-3	Z453319206	bmse011146	InChI=1S/C16H12N2O/c19-16(12-5-2-1-3-6-12)18-14-8-9-15-13(11-14)7-4-10-17-15/h1-11H,(H,18,19)
**14**	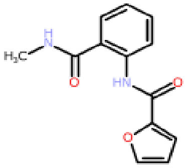	14	84141-29-7	Z30272547	bmse011189	InChI=1S/C13H12N2O3/c1-14-12(16)9-5-2-3-6-10(9)15-13(17)11-7-4-8-18-11/h2-8H,1H3,(H,14,16)(H,15,17)
**15**	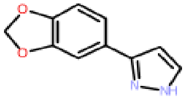	15	141791-06-2	Z2204875953	bmse011337	InChI=1S/C10H8N2O2/c1-2-9-10(14-6-13-9)5-7(1)8-3-4-11-12-8/h1-5H,6H2,(H,11,12)

Suppl Table 2: Details of the 768 compounds from the DSI-Poised fragment Library (DSI-PL)

Sl. No.	Structures	No. in Manuscript	CAS Registry Number	"ID"	^{1}H NMR Data Repository BMRB ID	ALATIS InChI
**16**	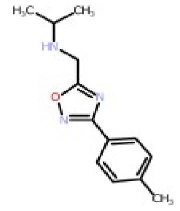	16	708284-23-5	Z2856434849	bmse011650	InChI=1S/C13H17N3O/c1-9(2)14-8-12-15-13(16-17-12)11-6-4-10(3)5-7-11/h4-7,9,14H,8H2,1-3H3
**17**	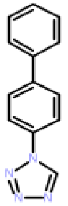	17	63472-38-8	Z57479285	bmse011344	nChI=1S/C13H10N4/c1-2-4-11(5-3-1)12-6-8-13(9-7-12)17-10-14-15-16-17/h1-10
**18**	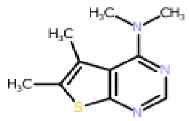	18	690684-98-1	Z31222641	bmse011491	InChI=1S/C10H13N3S/c1-6-7(2)14-10-8(6)9(13(3)4)11-5-12-10/h5H,1-4H3
**19**	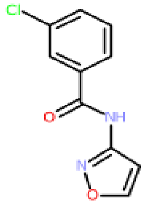	19	777880-26-9	Z86622311	bmse011719	InChI=1S/C10H7ClN2O2/c11-8-3-1-2-7(6-8)10(14)12-9-4-5-15-13-9/h1-6H,(H,12,13,14)
**20**	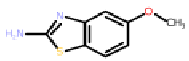	20	54346-87-1	Z1954800564	bmse011353	InChI=1S/C8H8N2OS/c1-11-5-2-3-7-6(4-5)10-8(9)12-7/h2-4H,1H3,(H2,9,10)

Suppl Table 2: Details of the 768 compounds from the DSI-Poised fragment Library (DSI-PL)

Sl. No.	Structures	No. in Manuscript	CAS Registry Number	"ID"	^{1}H NMR Data Repository BMRB ID	ALATIS InChI
**21**	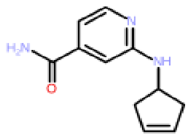	21	1445660-57-0	Z1416571195	bmse011416	InChI=1S/C11H13N3O/c12-11(15)8-5-6-13-10(7-8)14-9-3-1-2-4-9/h1-2,5-7,9H,3-4H2,(H2,12,15)(H,13,14)
**22**	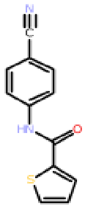	22	137272-71-0	Z30612387	bmse011033	InChI=1S/C12H8N2OS/c13-8-9-3-5-10(6-4-9)14-12(15)11-2-1-7-16-11/h1-7H,(H,14,15)
**23**	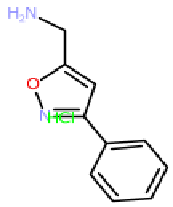	23	13608-55-4	Z425449682	bmse011093	InChI=1S/C10H10N2O.ClH/c11-7-9-6-10(12-13-9)8-4-2-1-3-5-8;/h1-6H,7,11H2;1H
**24**	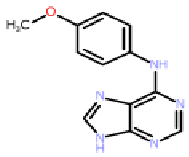	24	6296-91-9	Z276545932	bmse011059	InChI=1S/C12H11N5O/c1-18-9-4-2-8(3-5-9)17-12-10-11(14-6-13-10)15-7-16-12/h2-7H,1H3,(H2,13,14,15,16,17)
**25**	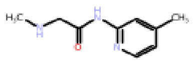	25	1019383-78-8	Z927746322	bmse011463	InChI=1S/C9H13N3O/c1-7-3-4-11-8(5-7)12-9(13)6-10-2/h3-5,10H,6H2,1-2H3,(H,11,12,13)

Suppl Table 2: Details of the 768 compounds from the DSI-Poised fragment Library (DSI-PL)

Sl. No.	Structures	No. in Manuscript	CAS Registry Number	"ID"	^{1}H NMR Data Repository BMRB ID	ALATIS InChI
**26**	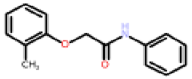	26	22560-44-7	Z19733979	bmse011328	InChI=1S/C15H15NO2/c1-12-7-5-6-10-14(12)18-11-15(17)16-13-8-3-2-4-9-13/h2-10H,11H2,1H3,(H,16,17)
**27**	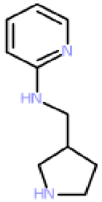	27	1251925-43-5	Z1262327505	bmse011522	InChI=1S/C10H15N3/c1-2-5-12-10(3-1)13-8-9-4-6-11-7-9/h1-3,5,9,11H,4,6-8H2,(H,12,13)/t9-/m0/s1
**28**	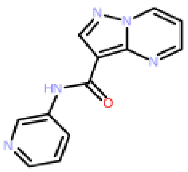	28	717831-80-6	Z296054478	bmse011360	InChI=1S/C12H9N5O/c18-12(16-9-3-1-4-13-7-9)10-8-15-17-6-2-5-14-11(10)17/h1-8H,(H,16,18)
**29**	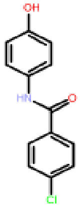	29	19207-92-2	Z56346825	bmse011001	InChI=1S/C13H10ClNO2/c14-10-3-1-9(2-4-10)13(17)15-11-5-7-12(16)8-6-11/h1-8,16H,(H,15,17)
**30**	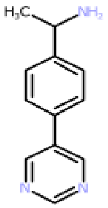	30	1250290-30-2	Z1696822287	bmse011426	InChI=1S/C12H13N3/c1-9(13)10-2-4-11(5-3-10)12-6-14-8-15-7-12/h2-9H,13H2,1H3/t9-/m0/s1

Suppl Table 2: Details of the 768 compounds from the DSI-Poised fragment Library (DSI-PL)

Sl. No.	Structures	No. in Manuscript	CAS Registry Number	"ID"	^{1}H NMR Data Repository BMRB ID	ALATIS InChI
**31**	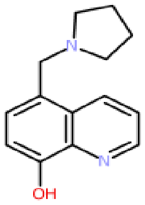	31	41455-82-7	Z2856434902	bmse011447	InChI=1S/C14H16N2O/c17-13-6-5-11(10-16-8-1-2-9-16)12-4-3-7-15-14(12)13/h3-7,17H,1-2,8-10H2
**32**	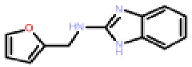	32	727392-72-5	Z57299526	bmse011727	InChI=1S/C12H11N3O/c1-2-6-11-10(5-1)14-12(15-11)13-8-9-4-3-7-16-9/h1-7H,8H2,(H2,13,14,15)
**33**	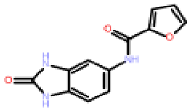	33	339207-13-5	Z26794338	bmse011698	InChI=1S/C12H9N3O3/c16-11(10-2-1-5-18-10)13-7-3-4-8-9(6-7)15-12(17)14-8/h1-6H,(H,13,16)(H2,14,15,17)
**34**	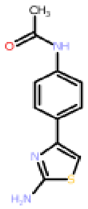	34	21674-96-4	Z48847594	bmse011438	InChI=1S/C11H11N3OS/c1-7(15)13-9-4-2-8(3-5-9)10-6-16-11(12)14-10/h2-6H,1H3,(H2,12,14)(H,13,15)
**35**	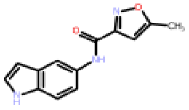	35	708987-60-4	Z1190787729	bmse011725	InChI=1S/C13H11N3O2/c1-8-6-12(16-18-8)13(17)15-10-2-3-11-9(7-10)4-5-14-11/h2-7,14H,1H3,(H,15,17)

Suppl Table 2: Details of the 768 compounds from the DSI-Poised fragment Library (DSI-PL)

Sl. No.	Structures	No. in Manuscript	CAS Registry Number	"ID"	1H NMR Data Repository BMRB ID	ALATIS InChI
**36**	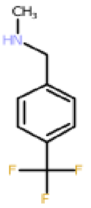	36	90390-11-7	Z2856434809	bmse011474	InChI=1S/C9H10F3N/c1-13-6-7-2-4-8(5-3-7)9(10,11)12/h2-5,13H,6H2,1H3
**37**	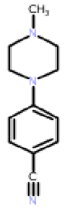	37	34334-28-6	Z2856434840	bmse011037	InChI=1S/C12H15N3/c1-14-6-8-15(9-7-14)12-4-2-11(10-13)3-5-12/h2-5H,6-9H2,1H3
**38**	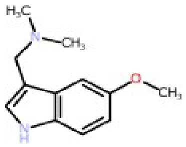	38	16620-52-3	Z2856434938	bmse011469	InChI=1S/C12H16N2O/c1-14(2)8-9-7-13-12-5-4-10(15-3)6-11(9)12/h4-7,13H,8H2,1-3H3
**39**	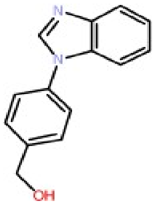	39	451485-67-9	Z235449082	bmse011062	InChI=1S/C14H12N2O/c17-9-11-5-7-12(8-6-11)16-10-15-13-3-1-2-4-14(13)16/h1-8,10,17H,9H2
**40**	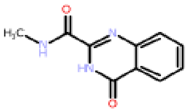	40	53115-17-6	Z332370018	bmse011072	InChI=1S/C10H9N3O2/c1-11-10(15)8-12-7-5-3-2-4-6(7)9(14)13-8/h2-5H,1H3,(H,11,15)(H,12,13,14)

Suppl Table 2: Details of the 768 compounds from the DSI-Poised fragment Library (DSI-PL)

Sl. No.	Structures	No. in Manuscript	CAS Registry Number	"ID"	^{1}H NMR Data Repository BMRB ID	ALATIS InChI
**41**	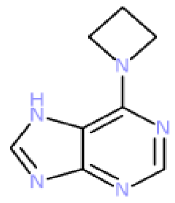	41	1379186-26-1	Z373221060	bmse011127	InChI=1S/C8H9N5/c1-2-13(3-1)8-6-7(10-4-9-6)11-5-12-8/h4-5H,1-3H2,(H,9,10,11,12)
**42**	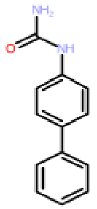	42	13262-48-1	Z198195774	bmse011251	InChI=1S/C13H12N2O/c14-13(16)15-12-8-6-11(7-9-12)10-4-2-1-3-5-10/h1-9H,(H3,14,15,16)
**43**	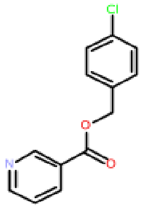	43	100398-39-8	Z57515803	bmse011305	InChI=1S/C13H10ClNO2/c14-12-5-3-10(4-6-12)9-17-13(16)11-2-1-7-15-8-11/h1-8H,9H2
**44**	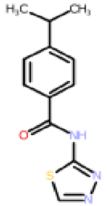	44	544690-34-8	Z86416893	bmse011331	InChI=1S/C12H13N3OS/c1-8(2)9-3-5-10(6-4-9)11(16)14-12-15-13-7-17-12/h3-8H,1-2H3,(H,14,15,16)
**45**	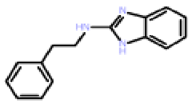	45	727392-70-3	Z57299529	bmse011629	InChI=1S/C15H15N3/c1-2-6-12(7-3-1)10-11-16-15-17-13-8-4-5-9-14(13)18-15/h1-9H,10-11H2,(H2,16,17,18)

Suppl Table 2: Details of the 768 compounds from the DSI-Poised fragment Library (DSI-PL)

Sl. No.	Structures	No. in Manuscript	CAS Registry Number	"ID"	1H NMR Data Repository BMRB ID	ALATIS InChI
**46**	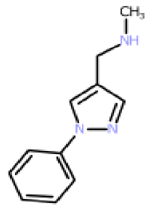	46	868552-05-0	Z102768020	bmse011449	InChI=1S/C11H13N3/c1-12-7-10-8-13-14(9-10)11-5-3-2-4-6-11/h2-6,8-9,12H,7H2,1H3
**47**	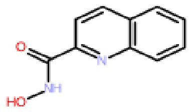	47	37137-42-1	Z59181945	bmse011401	InChI=1S/C10H8N2O2/c13-10(12-14)9-6-5-7-3-1-2-4-8(7)11-9/h1-6,14H,(H,12,13)
**48**	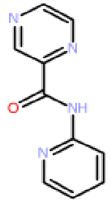	48	484039-13-6	Z85525386	bmse011215	InChI=1S/C10H8N4O/c15-10(8-7-11-5-6-12-8)14-9-3-1-2-4-13-9/h1-7H,(H,13,14,15)
**49**	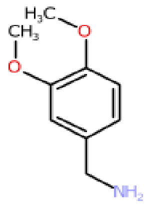	49	5763-61-1	Z2856434917	bmse011473	InChI=1S/C9H13NO2/c1-11-8-4-3-7(6-10)5-9(8)12-2/h3-5H,6,10H2,1-2H3
**50**	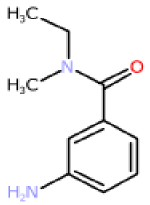	50	1094911-27-9	Z2092370954	bmse011131	InChI=1S/C10H14N2O/c1-3-12(2)10(13)8-5-4-6-9(11)7-8/h4-7H,3,11H2,1-2H3

Suppl Table 2: Details of the 768 compounds from the DSI-Poised fragment Library (DSI-PL)

Sl. No.	Structures	No. in Manuscript	CAS Registry Number	"ID"	^{1}H NMR Data Repository BMRB ID	ALATIS InChI
**51**	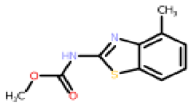	51	717868-63-8	Z192981502	bmse011679	InChI=1S/C10H10N2O2S/c1-6-4-3-5-7-8(6)11-9(15-7)12-10(13)14-2/h3-5H,1-2H3,(H,11,12,13)
**52**	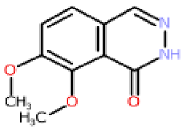	52	4821-89-0	Z102895082	bmse011050	InChI=1S/C10H10N2O3/c1-14-7-4-3-6-5-11-12-10(13)8(6)9(7)15-2/h3-5H,1-2H3,(H,12,13)
**53**	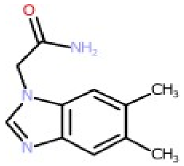	53	953847-76-2	Z26251905	bmse011083	InChI=1S/C11H13N3O/c1-7-3-9-10(4-8(7)2)14(6-13-9)5-11(12)15/h3-4,6H,5H2,1-2H3,(H2,12,15)
**54**	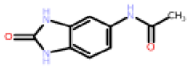	54	91085-68-6	Z26794305	bmse011177	InChI=1S/C9H9N3O2/c1-5(13)10-6-2-3-7-8(4-6)12-9(14)11-7/h2-4H,1H3,(H,10,13)(H2,11,12,14)
**55**	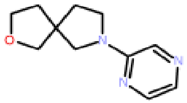	55	1607251-73-9	Z1401276297	bmse011101	InChI=1S/C11H15N3O/c1-5-14(10-7-12-3-4-13-10)8-11(1)2-6-15-9-11/h3-4,7H,1-2,5-6,8-9H2/t11-/m0/s1

Suppl Table 2: Details of the 768 compounds from the DSI-Poised fragment Library (DSI-PL)

Sl. No.	Structures	No. in Manuscript	CAS Registry Number	"ID"	^{1}H NMR Data Repository BMRB ID	ALATIS InChI
**56**	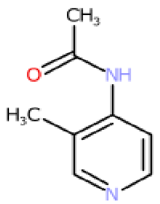	56	104915-66-4	Z1148747945	bmse011159	InChI=1S/C8H10N2O/c1-6-5-9-4-3-8(6)10-7(2)11/h3-5H,1-2H3,(H,9,10,11)
**57**	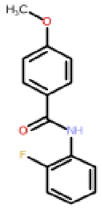	57	143925-52-4	Z28290321	bmse011232	InChI=1S/C14H12FNO2/c1-18-11-8-6-10(7-9-11)14(17)16-13-5-3-2-4-12(13)15/h2-9H,1H3,(H,16,17)
**58**	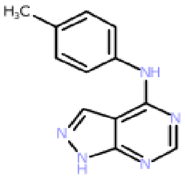	58	369396-26-9	Z57101343	bmse011290	InChI=1S/C12H11N5/c1-8-2-4-9(5-3-8)16-11-10-6-15-17-12(10)14-7-13-11/h2-7H,1H3,(H2,13,14,15,16,17)
**59**	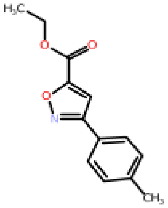	59	377062-62-9	Z793500562	bmse011326	InChI=1S/C13H13NO3/c1-3-16-13(15)12-8-11(14-17-12)10-6-4-9(2)5-7-10/h4-8H,3H2,1-2H3
**60**	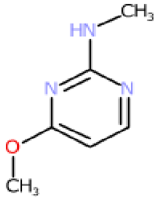	60	66131-71-3	Z281802060	bmse011406	InChI=1S/C6H9N3O/c1-7-6-8-4-3-5(9-6)10-2/h3-4H,1-2H3,(H,7,8,9)

Suppl Table 2: Details of the 768 compounds from the DSI-Poised fragment Library (DSI-PL)

Sl. No.	Structures	No. in Manuscript	CAS Registry Number	"ID"	^{1}H NMR Data Repository BMRB ID	ALATIS InChI
**61**	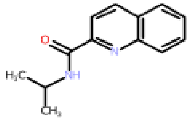	61	586982-18-5	Z31385861	bmse011586	InChI=1S/C13H14N2O/c1-9(2)14-13(16)12-8-7-10-5-3-4-6-11(10)15-12/h3-9H,1-2H3,(H,14,16)
**62**	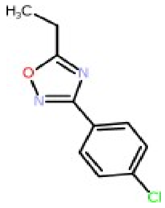	62	196301-93-6	Z57792053	bmse011692	InChI=1S/C10H9ClN2O/c1-2-9-12-10(13-14-9)7-3-5-8(11)6-4-7/h3-6H,2H2,1H3
**63**	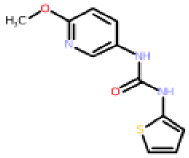	63	719269-39-3	Z1152242726	bmse011711	InChI=1S/C11H11N3O2S/c1-16-9-5-4-8(7-12-9)13-11(15)14-10-3-2-6-17-10/h2-7H,1H3,(H2,13,14,15)
**64**	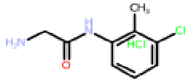	64	1052545-13-7	Z85956652	bmse011008	InChI=1S/C9H11ClN2O.ClH/c1-6-7(10)3-2-4-8(6)12-9(13)5-11;/h2-4H,5,11H2,1H3,(H,12,13);1H
**65**	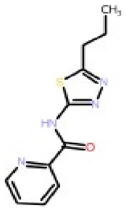	65	691382-96-4	Z52425517	bmse011130	InChI=1S/C11H12N4OS/c1-2-5-9-14-15-11(17-9)13-10(16)8-6-3-4-7-12-8/h3-4,6-7H,2,5H2,1H3,(H,13,15,16)

Suppl Table 2: Details of the 768 compounds from the DSI-Poised fragment Library (DSI-PL)

Sl. No.	Structures	No. in Manuscript	CAS Registry Number	"ID"	^{1}H NMR Data Repository BMRB ID	ALATIS InChI
**66**	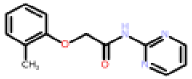	66	723737-15-3	Z53834613	bmse011182	InChI=1S/C13H13N3O2/c1-10-5-2-3-6-11(10)18-9-12(17)16-13-14-7-4-8-15-13/h2-8H,9H2,1H3,(H,14,15,16,17)
**67**	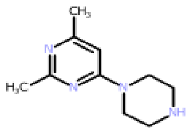	67	887686-65-9	Z274575916	bmse011270	InChI=1S/C10H16N4/c1-8-7-10(13-9(2)12-8)14-5-3-11-4-6-14/h7,11H,3-6H2,1-2H3
**68**	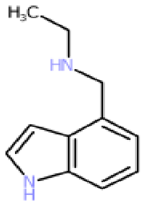	68	342412-30-0	Z1137725943	bmse011556	InChI=1S/C11H14N2/c1-2-12-8-9-4-3-5-11-10(9)6-7-13-11/h3-7,12-13H,2,8H2,1H3
**69**	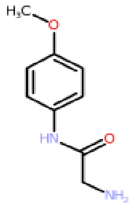	69	148627-63-8	Z1449748885	bmse011624	InChI=1S/C9H12N2O2/c1-13-8-4-2-7(3-5-8)11-9(12)6-10/h2-5H,6,10H2,1H3,(H,11,12)
**70**	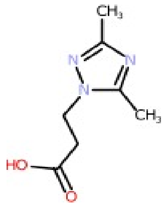	n.a.	842971-05-5	Z274553586	bmse011095	InChI=1S/C7H11N3O2/c1-5-8-6(2)10(9-5)4-3-7(11)12/h3-4H2,1-2H3,(H,11,12)

Suppl Table 2: Details of the 768 compounds from the DSI-Poised fragment Library (DSI-PL)

Sl. No.	Structures	No. in Manuscript	CAS Registry Number	"ID"	1H NMR Data Repository BMRB ID	ALATIS InChI
**71**	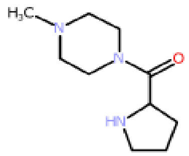	n.a.	100158-68-7	Z241832786	bmse011528	InChI=1S/C10H19N3O/c1-12-5-7-13(8-6-12)10(14)9-3-2-4-11-9/h9,11H,2-8H2,1H3/t9-/m1/s1
**72**	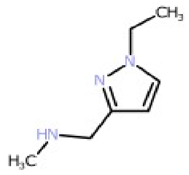	n.a.	1002651-65-1	Z2856434888	bmse011472	InChI=1S/C7H13N3/c1-3-10-5-4-7(9-10)6-8-2/h4-5,8H,3,6H2,1-2H3
**73**	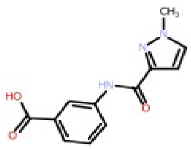	n.a.	1002651-89-9	Z2856434892	bmse011746	InChI=1S/C12H11N3O3/c1-15-6-5-10(14-15)11(16)13-9-4-2-3-8(7-9)12(17)18/h2-7H,1H3,(H,13,16)(H,17,18)
**74**	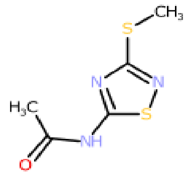	n.a.	100517-04-2	Z26804467	bmse011432	InChI=1S/C5H7N3OS2/c1-3(9)6-4-7-5(10-2)8-11-4/h1-2H3,(H,6,7,8,9)
**75**	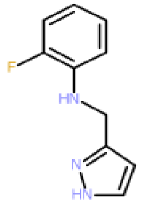	n.a.	1006438-26-1	Z1545196403	bmse011560	InChI=1S/C10H10FN3/c11-9-3-1-2-4-10(9)12-7-8-5-6-13-14-8/h1-6,12H,7H2,(H,13,14)

Suppl Table 2: Details of the 768 compounds from the DSI-Poised fragment Library (DSI-PL)

Sl. No.	Structures	No. in Manuscript	CAS Registry Number	"ID"	^{1}H NMR Data Repository BMRB ID	ALATIS InChI
**76**	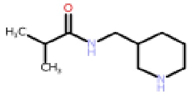	n.a.	1016685-94-1	Z1262327459	bmse011604	InChI=1S/C10H20N2O/c1-8(2)10(13)12-7-9-4-3-5-11-6-9/h8-9,11H,3-7H2,1-2H3,(H,12,13)/t9-/m0/s1
**77**	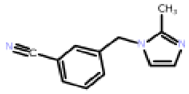	n.a.	1016716-83-8	Z319545618	bmse011254	InChI=1S/C12H11N3/c1-10-14-5-6-15(10)9-12-4-2-3-11(7-12)8-13/h2-7H,9H2,1H3
**78**	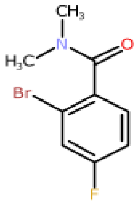	n.a.	1016749-54-4	Z100642432	bmse011257	InChI=1S/C9H9BrFNO/c1-12(2)9(13)7-4-3-6(11)5-8(7)10/h3-5H,1-2H3
**79**	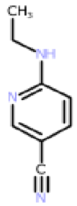	n.a.	1016813-34-5	Z219104216	bmse011084	InChI=1S/C8H9N3/c1-2-10-8-4-3-7(5-9)6-11-8/h3-4,6H,2H2,1H3,(H,10,11)
**80**	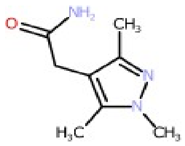	n.a.	1017502-16-7	Z287484230	bmse011244	InChI=1S/C8H13N3O/c1-5-7(4-8(9)12)6(2)11(3)10-5/h4H2,1-3H3,(H2,9,12)

Suppl Table 2: Details of the 768 compounds from the DSI-Poised fragment Library (DSI-PL)

Sl. No.	Structures	No. in Manuscript	CAS Registry Number	"ID"	1H NMR Data Repository BMRB ID	ALATIS InChI
**81**	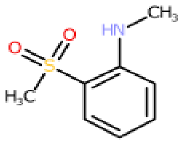	n.a.	10224-69-8	Z285782452	bmse011392	InChI=1S/C8H11NO2S/c1-9-7-5-3-4-6-8(7)12(2,10)11/h3-6,9H,1-2H3
**82**	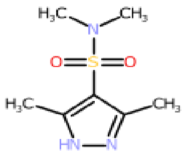	n.a.	1025067-61-1	Z94597856	bmse011194	InChI=1S/C7H13N3O2S/c1-5-7(6(2)9-8-5)13(11,12)10(3)4/h1-4H3,(H,8,9)
**83**	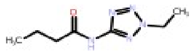	n.a.	1025922-90-0	Z57475877	bmse011310	InChI=1S/C7H13N5O/c1-3-5-6(13)8-7-9-11-12(4-2)10-7/h3-5H2,1-2H3,(H,8,10,13)
**84**	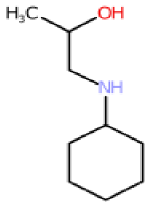	n.a.	103-00-4	Z90526999	bmse011272	InChI=1S/C9H19NO/c1-8(11)7-10-9-5-3-2-4-6-9/h8-11H,2-7H2,1H3/t8-/m0/s1
**85**	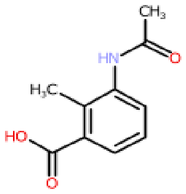	n.a.	103204-68-8	Z364368134	bmse011107	InChI=1S/C10H11NO3/c1-6-8(10(13)14)4-3-5-9(6)11-7(2)12/h3-5H,1-2H3,(H,11,12)(H,13,14)

Suppl Table 2: Details of the 768 compounds from the DSI-Poised fragment Library (DSI-PL)

Sl. No.	Structures	No. in Manuscript	CAS Registry Number	"ID"	^{1}H NMR Data Repository BMRB ID	ALATIS InChI
**86**	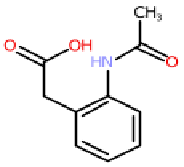	n.a.	103205-34-1	Z1444783243	bmse011411	InChI=1S/C10H11NO3/c1-7(12)11-9-5-3-2-4-8(9)6-10(13)14/h2-5H,6H2,1H3,(H,11,12)(H,13,14)
**87**	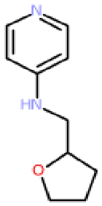	n.a.	1036514-53-0	Z1267882044	bmse011541	InChI=1S/C10H14N2O/c1-2-10(13-7-1)8-12-9-3-5-11-6-4-9/h3-6,10H,1-2,7-8H2,(H,11,12)/t10-/m1/s1
**88**	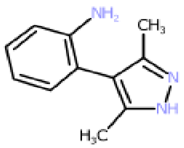	n.a.	103858-98-6	Z1270312110	bmse011386	InChI=1S/C11H13N3/c1-7-11(8(2)14-13-7)9-5-3-4-6-10(9)12/h3-6H,12H2,1-2H3,(H,13,14)
**89**	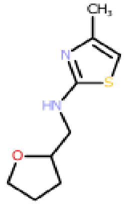	n.a.	1039996-30-9	Z48852953	bmse011223	InChI=1S/C9H14N2OS/c1-7-6-13-9(11-7)10-5-8-3-2-4-12-8/h6,8H,2-5H2,1H3,(H,10,11)/t8-/m1/s1
**90**	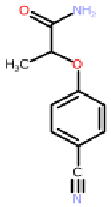	n.a.	1040041-89-1	Z19739650	bmse011221	InChI=1S/C10H10N2O2/c1-7(10(12)13)14-9-4-2-8(6-11)3-5-9/h2-5,7H,1H3,(H2,12,13)/t7-/m0/s1

Suppl Table 2: Details of the 768 compounds from the DSI-Poised fragment Library (DSI-PL)

Sl. No.	Structures	No. in Manuscript	CAS Registry Number	"ID"	^{1}H NMR Data Repository BMRB ID	ALATIS InChI
**91**	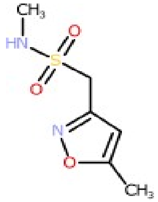	n.a.	1042787-05-2	Z763030030	bmse011201	InChI=1S/C6H10N2O3S/c1-5-3-6(8-11-5)4-12(9,10)7-2/h3,7H,4H2,1-2H3
**92**	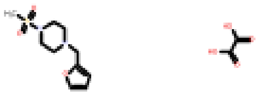	n.a.	1047978-76-6	Z2241115980	bmse011220	InChI=1S/C10H16N2O3S.C2H2O4/c1-16(13,14)12-6-4-11(5-7-12)9-10-3-2-8-15-10;3-1(4)2(5)6/h2-3,8H,4-7,9H2,1H3;(H,3,4)(H,5,6)
**93**	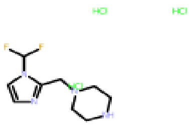	n.a.	1049726-54-6	Z2444672448	bmse011450	InChI=1S/C9H14F2N4.3ClH/c10-9(11)15-6-3-13-8(15)7-14-4-1-12-2-5-14;;;/h3,6,9,12H,1-2,4-5,7H2;3*1H
**94**	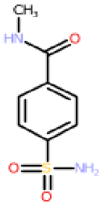	n.a.	10518-85-1	Z165170770	bmse011212	InChI=1S/C8H10N2O3S/c1-10-8(11)6-2-4-7(5-3-6)14(9,12)13/h2-5H,1H3,(H,10,11)(H2,9,12,13)
**95**	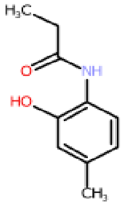	n.a.	105296-19-3	Z33452106	bmse011202	InChI=1S/C10H13NO2/c1-3-10(13)11-8-5-4-7(2)6-9(8)12/h4-6,12H,3H2,1-2H3,(H,11,13)

Suppl Table 2: Details of the 768 compounds from the DSI-Poised fragment Library (DSI-PL)

Sl. No.	Structures	No. in Manuscript	CAS Registry Number	"ID"	1H NMR Data Repository BMRB ID	ALATIS InChI
**96**	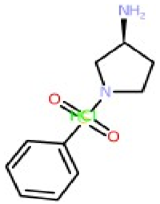	n.a.	1056998-45-8	Z2443429438	bmse011132	InChI=1S/C10H14N2O2S.ClH/c11-9-6-7-12(8-9)15(13,14)10-4-2-1-3-5-10;/h1-5,9H,6-8,11H2;1H/t9-;/m0./s1
**97**	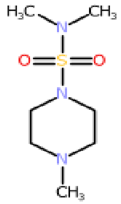	n.a.	105871-27-0	Z2735592898	bmse011704	InChI=1S/C7H17N3O2S/c1-8(2)13(11,12)10-6-4-9(3)5-7-10/h4-7H2,1-3H3
**98**	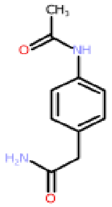	n.a.	1060283-53-5	Z87615031	bmse011281	InChI=1S/C10H12N2O2/c1-7(13)12-9-4-2-8(3-5-9)6-10(11)14/h2-5H,6H2,1H3,(H2,11,14)(H,12,13)
**99**	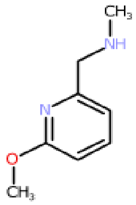	n.a.	1060806-94-1	Z1603775714	bmse011748	InChI=1S/C8H12N2O/c1-9-6-7-4-3-5-8(10-7)11-2/h3-5,9H,6H2,1-2H3
**100**	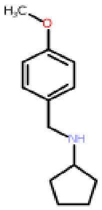	n.a.	108157-23-9	Z2856434940	bmse011715	InChI=1S/C13H19NO/c1-15-13-8-6-11(7-9-13)10-14-12-4-2-3-5-12/h6-9,12,14H,2-5,10H2,1H3

Suppl Table 2: Details of the 768 compounds from the DSI-Poised fragment Library (DSI-PL)

Sl. No.	Structures	No. in Manuscript	CAS Registry Number	"ID"	1H NMR Data Repository BMRB ID	ALATIS InChI
**101**	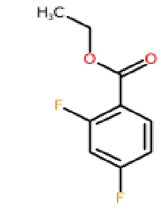	n.a.	108928-00-3	Z53836105	bmse011644	InChI=1S/C9H8F2O2/c1-2-13-9(12)7-4-3-6(10)5-8(7)11/h3-5H,2H2,1H3
**102**	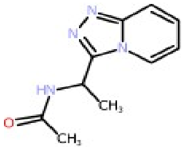	n.a.	1090718-29-8	Z131516158	bmse011239	InChI=1S/C10H12N4O/c1-7(11-8(2)15)10-13-12-9-5-3-4-6-14(9)10/h3-7H,1-2H3,(H,11,15)/t7-/m0/s1
**103**	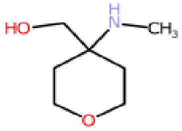	n.a.	1094072-05-5	Z1169060901	bmse011557	InChI=1S/C7H15NO2/c1-8-7(6-9)2-4-10-5-3-7/h8-9H,2-6H2,1H3
**104**	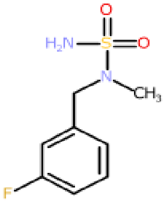	n.a.	1094315-37-3	Z300245038	bmse011222	InChI=1S/C8H11FN2O2S/c1-11(14(10,12)13)6-7-3-2-4-8(9)5-7/h2-5H,6H2,1H3,(H2,10,12,13)
**105**	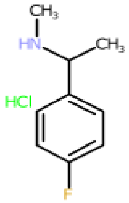	n.a.	1095514-42-3	Z166605480	bmse011451	InChI=1S/C9H12FN.ClH/c1-7(11-2)8-3-5-9(10)6-4-8;/h3-7,11H,1-2H3;1H/t7-;/m0./s1

Suppl Table 2: Details of the 768 compounds from the DSI-Poised fragment Library (DSI-PL)

Sl. No.	Structures	No. in Manuscript	CAS Registry Number	"ID"	^{1}H NMR Data Repository BMRB ID	ALATIS InChI
**106**	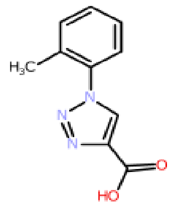	n.a.	1099631-80-7	Z2327226104	bmse011142	InChI=1S/C10H9N3O2/c1-7-4-2-3-5-9(7)13-6-8(10(14)15)11-12-13/h2-6H,1H3,(H,14,15)
**107**	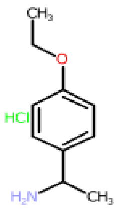	n.a.	1108684-07-6	Z210803634	bmse011126	InChI=1S/C10H15NO.ClH/c1-3-12-10-6-4-9(5-7-10)8(2)11;/h4-8H,3,11H2,1-2H3;1H/t8-;/m0./s1
**108**	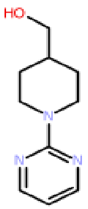	n.a.	111247-61-1	Z645232558	bmse011666	InChI=1S/C10H15N3O/c14-8-9-2-6-13(7-3-9)10-11-4-1-5-12-10/h1,4-5,9,14H,2-3,6-8H2
**109**	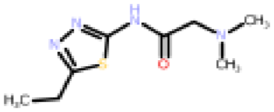	n.a.	111750-46-0	Z2856434846	bmse011363	InChI=1S/C8H14N4OS/c1-4-7-10-11-8(14-7)9-6(13)5-12(2)3/h4-5H2,1-3H3,(H,9,11,13)
**110**	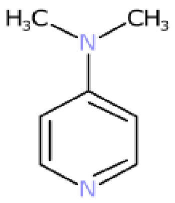	n.a.	1122-58-3	Z2856434762	bmse011259	InChI=1S/C7H10N2/c1-9(2)7-3-5-8-6-4-7/h3-6H,1-2H3

Suppl Table 2: Details of the 768 compounds from the DSI-Poised fragment Library (DSI-PL)

Sl. No.	Structures	No. in Manuscript	CAS Registry Number	"ID"	^{1}H NMR Data Repository BMRB ID	ALATIS InChI
**111**	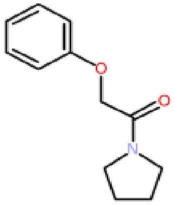	n.a.	112283-41-7	Z19735981	bmse011701	InChI=1S/C12H15NO2/c14-12(13-8-4-5-9-13)10-15-11-6-2-1-3-7-11/h1-3,6-7H,4-5,8-10H2
**112**	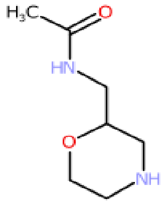	n.a.	112913-95-8	Z1269638430	bmse011461	InChI=1S/C7H14N2O2/c1-6(10)9-5-7-4-8-2-3-11-7/h7-8H,2-5H2,1H3,(H,9,10)/t7-/m0/s1
**113**	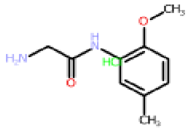	n.a.	1147208-19-2	Z235341991	bmse011152	InChI=1S/C10H14N2O2.ClH/c1-7-3-4-9(14-2)8(5-7)12-10(13)6-11;/h3-5H,6,11H2,1-2H3,(H,12,13);1H
**114**	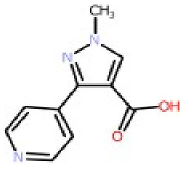	n.a.	1152510-62-7	Z815264062	bmse011206	InChI=1S/C10H9N3O2/c1-13-6-8(10(14)15)9(12-13)7-2-4-11-5-3-7/h2-6H,1H3,(H,14,15)
**115**	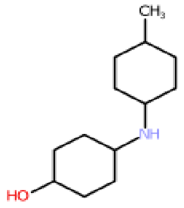	n.a.	1152964-08-3	Z2241963319	bmse011271	InChI=1S/C13H25NO/c1-10-2-4-11(5-3-10)14-12-6-8-13(15)9-7-12/h10-15H,2-9H2,1H3/t10-,11-,12-,13-

Suppl Table 2: Details of the 768 compounds from the DSI-Poised fragment Library (DSI-PL)

Sl. No.	Structures	No. in Manuscript	CAS Registry Number	"ID"	^{1}H NMR Data Repository BMRB ID	ALATIS InChI
**116**	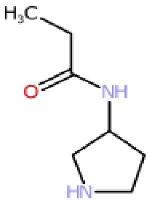	n.a.	1154969-50-2	Z1650040241	bmse011531	InChI=1S/C7H14N2O/c1-2-7(10)9-6-3-4-8-5-6/h6,8H,2-5H2,1H3,(H,9,10)/t6-/m0/s1
**117**	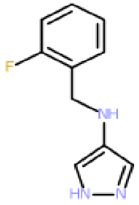	n.a.	1156177-23-9	Z1454310449	bmse011116	InChI=1S/C10H10FN3/c11-10-4-2-1-3-8(10)5-12-9-6-13-14-7-9/h1-4,6-7,12H,5H2,(H,13,14)
**118**	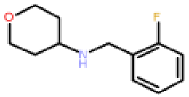	n.a.	1157009-10-3	Z823455846	bmse011737	InChI=1S/C12H16FNO/c13-12-4-2-1-3-10(12)9-14-11-5-7-15-8-6-11/h1-4,11,14H,5-9H2
**119**	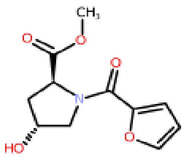	n.a.	1164125-09-0	Z1614545742	bmse011301	InChI=1S/C11H13NO5/c1-16-11(15)8-5-7(13)6-12(8)10(14)9-3-2-4-17-9/h2-4,7-8,13H,5-6H2,1H3/t7-,8+/m1/s1
**120**	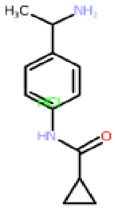	n.a.	1170962-96-5	Z1891773393	bmse011091	InChI=1S/C12H16N2O.ClH/c1-8(13)9-4-6-11(7-5-9)14-12(15)10-2-3-10;/h4-8,10H,2-3,13H2,1H3,(H,14,15);1H/t8-;/m0./s1

Suppl Table 2: Details of the 768 compounds from the DSI-Poised fragment Library (DSI-PL)

Sl. No.	Structures	No. in Manuscript	CAS Registry Number	"ID"	^{1}H NMR Data Repository BMRB ID	ALATIS InChI
**121**	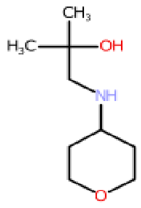	n.a.	1178195-37-3	Z1103351268	bmse011592	InChI=1S/C9H19NO2/c1-9(2,11)7-10-8-3-5-12-6-4-8/h8,10-11H,3-7H2,1-2H3
**122**	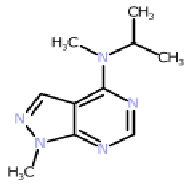	n.a.	117864-83-2	Z328695024	bmse011240	InChI=1S/C10H15N5/c1-7(2)14(3)9-8-5-13-15(4)10(8)12-6-11-9/h5-7H,1-4H3
**123**	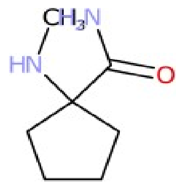	n.a.	1179878-43-3	Z1348559502	bmse011412	InChI=1S/C7H14N2O/c1-9-7(6(8)10)4-2-3-5-7/h9H,2-5H2,1H3,(H2,8,10)
**124**	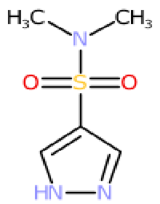	n.a.	1179901-00-8	Z805551440	bmse011106	InChI=1S/C5H9N3O2S/c1-8(2)11(9,10)5-3-6-7-4-5/h3-4H,1-2H3,(H,6,7)
**125**	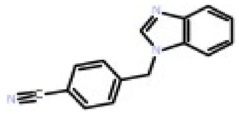	n.a.	118001-91-5	Z26333434	bmse011048	InChI=1S/C15H11N3/c16-9-12-5-7-13(8-6-12)10-18-11-17-14-3-1-2-4-15(14)18/h1-8,11H,10H2

Suppl Table 2: Details of the 768 compounds from the DSI-Poised fragment Library (DSI-PL)

Sl. No.	Structures	No. in Manuscript	CAS Registry Number	"ID"	^{1}H NMR Data Repository BMRB ID	ALATIS InChI
**126**	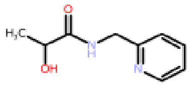	n.a.	118201-19-7	Z1551688335	bmse011120	InChI=1S/C9H12N2O2/c1-7(12)9(13)11-6-8-4-2-3-5-10-8/h2-5,7,12H,6H2,1H3,(H,11,13)/t7-/m0/s1
**127**	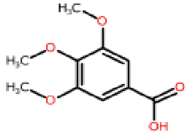	n.a.	118-41-2	Z2734782702	bmse011052	InChI=1S/C10H12O5/c1-13-7-4-6(10(11)12)5-8(14-2)9(7)15-3/h4-5H,1-3H3,(H,11,12)
**128**	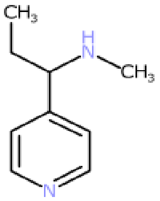	n.a.	1184131-82-5	Z1250132788	bmse011551	InChI=1S/C9H14N2/c1-3-9(10-2)8-4-6-11-7-5-8/h4-7,9-10H,3H2,1-2H3/t9-/m0/s1
**129**	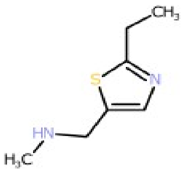	n.a.	1184195-56-9	Z1267773633	bmse011602	InChI=1S/C7H12N2S/c1-3-7-9-5-6(10-7)4-8-2/h5,8H,3-4H2,1-2H3
**130**	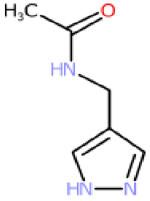	n.a.	1184541-34-1	Z383325512	bmse011164	InChI=1S/C6H9N3O/c1-5(10)7-2-6-3-8-9-4-6/h3-4H,2H2,1H3,(H,7,10)(H,8,9)

Suppl Table 2: Details of the 768 compounds from the DSI-Poised fragment Library (DSI-PL)

Sl. No.	Structures	No. in Manuscript	CAS Registry Number	"ID"	1H NMR Data Repository BMRB ID	ALATIS InChI
**131**	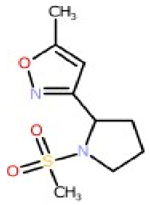	n.a.	1185124-04-2	Z319891284	bmse011102	InChI=1S/C9H14N2O3S/c1-7-6-8(10-14-7)9-4-3-5-11(9)15(2,12)13/h6,9H,3-5H2,1-2H3/t9-/m1/s1
**132**	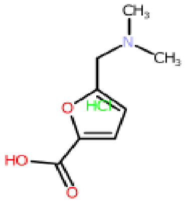	n.a.	1185300-64-4	Z359352902	bmse011173	InChI=1S/C8H11NO3.ClH/c1-9(2)5-6-3-4-7(12-6)8(10)11;/h3-4H,5H2,1-2H3,(H,10,11);1H
**133**	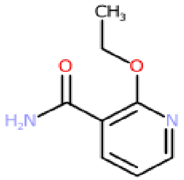	n.a.	119646-51-4	Z272156568	bmse011225	InChI=1S/C8H10N2O2/c1-2-12-8-6(7(9)11)4-3-5-10-8/h3-5H,2H2,1H3,(H2,9,11)
**134**	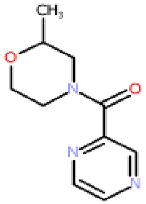	n.a.	1197773-69-5	Z212851096	bmse011489	InChI=1S/C10H13N3O2/c1-8-7-13(4-5-15-8)10(14)9-6-11-2-3-12-9/h2-3,6,8H,4-5,7H2,1H3/t8-/m1/s1
**135**	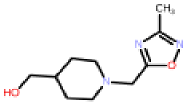	n.a.	1197809-26-9	Z416877194	bmse011496	InChI=1S/C10H17N3O2/c1-8-11-10(15-12-8)6-13-4-2-9(7-14)3-5-13/h9,14H,2-7H2,1H3

Suppl Table 2: Details of the 768 compounds from the DSI-Poised fragment Library (DSI-PL)

Sl. No.	Structures	No. in Manuscript	CAS Registry Number	"ID"	^{1}H NMR Data Repository BMRB ID	ALATIS InChI
**136**	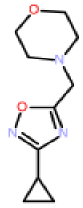	n.a.	1197900-26-7	Z295848548	bmse011490	InChI=1S/C10H15N3O2/c1-2-8(1)10-11-9(15-12-10)7-13-3-5-14-6-4-13/h8H,1-7H2
**137**	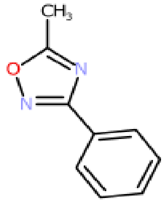	n.a.	1198-98-7	Z57791725	bmse011643	InChI=1S/C9H8N2O/c1-7-10-9(11-12-7)8-5-3-2-4-6-8/h2-6H,1H3
**138**	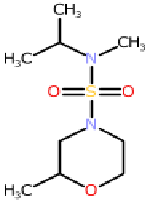	n.a.	1208453-10-4	Z416341642	bmse011492	InChI=1S/C9H20N2O3S/c1-8(2)10(4)15(12,13)11-5-6-14-9(3)7-11/h8-9H,5-7H2,1-4H3/t9-/m1/s1
**139**	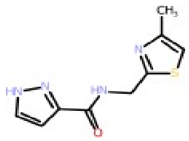	n.a.	1209647-58-4	Z466628048	bmse011208	InChI=1S/C9H10N4OS/c1-6-5-15-8(12-6)4-10-9(14)7-2-3-11-13-7/h2-3,5H,4H2,1H3,(H,10,14)(H,11,13)
**140**	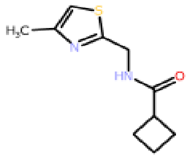	n.a.	1210016-75-3	Z212122838	bmse011420	InChI=1S/C10H14N2OS/c1-7-6-14-9(12-7)5-11-10(13)8-3-2-4-8/h6,8H,2-5H2,1H3,(H,11,13)

Suppl Table 2: Details of the 768 compounds from the DSI-Poised fragment Library (DSI-PL)

Sl. No.	Structures	No. in Manuscript	CAS Registry Number	"ID"	^{1}H NMR Data Repository BMRB ID	ALATIS InChI
**141**	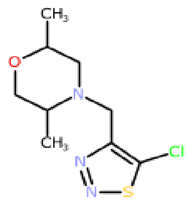	n.a.	1210287-28-7	Z354992234	bmse011516	InChI=1S/C9H14ClN3OS/c1-6-5-14-7(2)3-13(6)4-8-9(10)15-12-11-8/h6-7H,3-5H2,1-2H3/t6-,7+/m0/s1
**142**	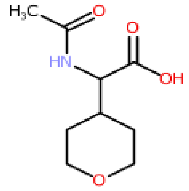	n.a.	1219171-26-2	Z1891772663	bmse011155	InChI=1S/C9H15NO4/c1-6(11)10-8(9(12)13)7-2-4-14-5-3-7/h7-8H,2-5H2,1H3,(H,10,11)(H,12,13)/t8-/m0/s1
**143**	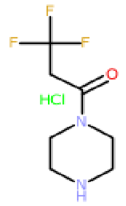	n.a.	1221722-69-5	Z1262246195	bmse011269	InChI=1S/C7H11F3N2O.ClH/c8-7(9,10)5-6(13)12-3-1-11-2-4-12;/h11H,1-5H2;1H
**144**	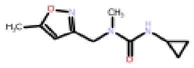	n.a.	1223010-53-4	Z369936976	bmse011501	InChI=1S/C10H15N3O2/c1-7-5-9(12-15-7)6-13(2)10(14)11-8-3-4-8/h5,8H,3-4,6H2,1-2H3,(H,11,14)
**145**	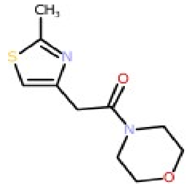	n.a.	1223036-54-1	Z31720228	bmse011497	InChI=1S/C10H14N2O2S/c1-8-11-9(7-15-8)6-10(13)12-2-4-14-5-3-12/h7H,2-6H2,1H3

Suppl Table 2: Details of the 768 compounds from the DSI-Poised fragment Library (DSI-PL)

Sl. No.	Structures	No. in Manuscript	CAS Registry Number	"ID"	^{1}H NMR Data Repository BMRB ID	ALATIS InChI
**146**	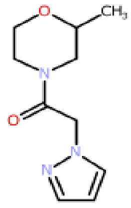	n.a.	1223167-92-7	Z217038356	bmse011210	InChI=1S/C10H15N3O2/c1-9-7-12(5-6-15-9)10(14)8-13-4-2-3-11-13/h2-4,9H,5-8H2,1H3/t9-/m1/s1
**147**	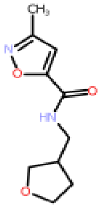	n.a.	1223482-48-1	Z384361454	bmse011030	InChI=1S/C10H14N2O3/c1-7-4-9(15-12-7)10(13)11-5-8-2-3-14-6-8/h4,8H,2-3,5-6H2,1H3,(H,11,13)/t8-/m1/s1
**148**	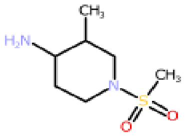	n.a.	1228453-26-6	Z1346370629	bmse011612	InChI=1S/C7H16N2O2S/c1-6-5-9(12(2,10)11)4-3-7(6)8/h6-7H,3-5,8H2,1-2H3/t6-,7+/m1/s1
**149**	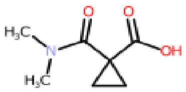	n.a.	1229625-39-1	Z1874937335	bmse011113	InChI=1S/C7H11NO3/c1-8(2)5(9)7(3-4-7)6(10)11/h3-4H2,1-2H3,(H,10,11)
**150**	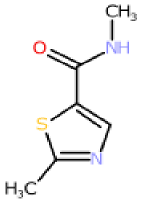	n.a.	1235439-10-7	Z969560582	bmse011218	InChI=1S/C6H8N2OS/c1-4-8-3-5(10-4)6(9)7-2/h3H,1-2H3,(H,7,9)

Suppl Table 2: Details of the 768 compounds from the DSI-Poised fragment Library (DSI-PL)

Sl. No.	Structures	No. in Manuscript	CAS Registry Number	"ID"	^{1}H NMR Data Repository BMRB ID	ALATIS InChI
**151**	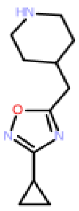	n.a.	1239851-15-0	Z2442270563	bmse011575	InChI=1S/C11H17N3O/c1-2-9(1)11-13-10(15-14-11)7-8-3-5-12-6-4-8/h8-9,12H,1-7H2
**152**	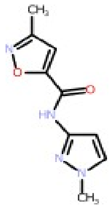	n.a.	1241174-55-9	Z240297434	bmse011217	InChI=1S/C9H10N4O2/c1-6-5-7(15-12-6)9(14)10-8-3-4-13(2)11-8/h3-5H,1-2H3,(H,10,11,14)
**153**	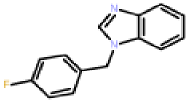	n.a.	124443-67-0	Z26333448	bmse011005	InChI=1S/C14H11FN2/c15-12-7-5-11(6-8-12)9-17-10-16-13-3-1-2-4-14(13)17/h1-8,10H,9H2
**154**	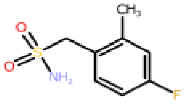	n.a.	1247342-88-6	Z1849009686	bmse011112	InChI=1S/C8H10FNO2S/c1-6-4-8(9)3-2-7(6)5-13(10,11)12/h2-4H,5H2,1H3,(H2,10,11,12)
**155**	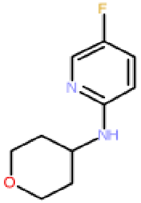	n.a.	1248980-68-8	Z1172243962	bmse011250	InChI=1S/C10H13FN2O/c11-8-1-2-10(12-7-8)13-9-3-5-14-6-4-9/h1-2,7,9H,3-6H2,(H,12,13)

Suppl Table 2: Details of the 768 compounds from the DSI-Poised fragment Library (DSI-PL)

Sl. No.	Structures	No. in Manuscript	CAS Registry Number	"ID"	^{1}H NMR Data Repository BMRB ID	ALATIS InChI
**156**	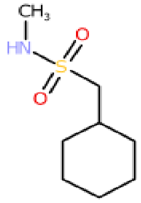	n.a.	1249194-40-8	Z1003207278	bmse011487	InChI=1S/C8H17NO2S/c1-9-12(10,11)7-8-5-3-2-4-6-8/h8-9H,2-7H2,1H3
**157**	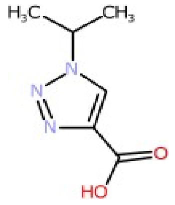	n.a.	1249780-66-2	Z2510259379	bmse011156	InChI=1S/C6H9N3O2/c1-4(2)9-3-5(6(10)11)7-8-9/h3-4H,1-2H3,(H,10,11)
**158**	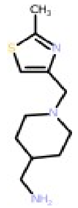	n.a.	1249955-95-0	Z1259335913	bmse011548	InChI=1S/C11H19N3S/c1-9-13-11(8-15-9)7-14-4-2-10(6-12)3-5-14/h8,10H,2-7,12H2,1H3
**159**	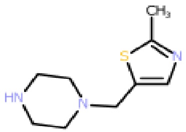	n.a.	1250118-20-7	Z1171217421	bmse011523	InChI=1S/C9H15N3S/c1-8-11-6-9(13-8)7-12-4-2-10-3-5-12/h6,10H,2-5,7H2,1H3
**160**	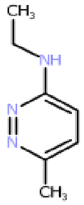	n.a.	1250628-49-9	Z1267773786	bmse011283	InChI=1S/C7H11N3/c1-3-8-7-5-4-6(2)9-10-7/h4-5H,3H2,1-2H3,(H,8,10)

Suppl Table 2: Details of the 768 compounds from the DSI-Poised fragment Library (DSI-PL)

Sl. No.	Structures	No. in Manuscript	CAS Registry Number	"ID"	^{1}H NMR Data Repository BMRB ID	ALATIS InChI
**161**	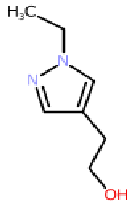	n.a.	1251131-12-0	Z1674937530	bmse011552	InChI=1S/C7H12N2O/c1-2-9-6-7(3-4-10)5-8-9/h5-6,10H,2-4H2,1H3
**162**	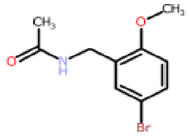	n.a.	1252130-18-9	Z955369596	bmse011230	InChI=1S/C10H12BrNO2/c1-7(13)12-6-8-5-9(11)3-4-10(8)14-2/h3-5H,6H2,1-2H3,(H,12,13)
**163**	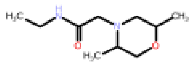	n.a.	1252134-18-1	Z355728146	bmse011500	InChI=1S/C10H20N2O2/c1-4-11-10(13)6-12-5-9(3)14-7-8(12)2/h8-9H,4-7H2,1-3H3,(H,11,13)/t8-,9+/m0/s1
**164**	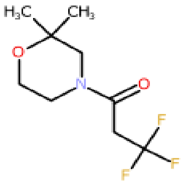	n.a.	1252136-02-9	Z394039592	bmse011234	InChI=1S/C9H14F3NO2/c1-8(2)6-13(3-4-15-8)7(14)5-9(10,11)12/h3-6H2,1-2H3
**165**	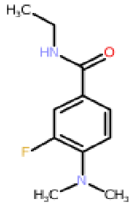	n.a.	1252274-64-8	Z907784200	bmse011236	InChI=1S/C11H15FN2O/c1-4-13-11(15)8-5-6-10(14(2)3)9(12)7-8/h5-7H,4H2,1-3H3,(H,13,15)

Suppl Table 2: Details of the 768 compounds from the DSI-Poised fragment Library (DSI-PL)

Sl. No.	Structures	No. in Manuscript	CAS Registry Number	"ID"	^{1}H NMR Data Repository BMRB ID	ALATIS InChI
**166**	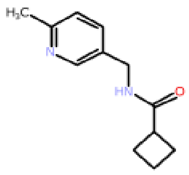	n.a.	1252297-64-5	Z437516460	bmse011235	InChI=1S/C12H16N2O/c1-9-5-6-10(7-13-9)8-14-12(15)11-3-2-4-11/h5-7,11H,2-4,8H2,1H3,(H,14,15)
**167**	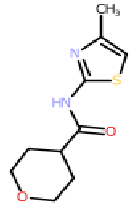	n.a.	1252372-19-2	Z73240835	bmse011238	InChI=1S/C10H14N2O2S/c1-7-6-15-10(11-7)12-9(13)8-2-4-14-5-3-8/h6,8H,2-5H2,1H3,(H,11,12,13)
**168**	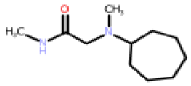	n.a.	1252443-78-9	Z204776284	bmse011498	InChI=1S/C11H22N2O/c1-12-11(14)9-13(2)10-7-5-3-4-6-8-10/h10H,3-9H2,1-2H3,(H,12,14)
**169**	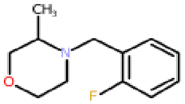	n.a.	1252499-28-7	Z369042042	bmse011499	InChI=1S/C12H16FNO/c1-10-9-15-7-6-14(10)8-11-4-2-3-5-12(11)13/h2-5,10H,6-9H2,1H3/t10-/m0/s1
**170**	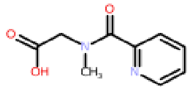	n.a.	125686-77-3	Z1171978788	bmse011249	InChI=1S/C9H10N2O3/c1-11(6-8(12)13)9(14)7-4-2-3-5-10-7/h2-5H,6H2,1H3,(H,12,13)

Suppl Table 2: Details of the 768 compounds from the DSI-Poised fragment Library (DSI-PL)

Sl. No.	Structures	No. in Manuscript	CAS Registry Number	"ID"	^{1}H NMR Data Repository BMRB ID	ALATIS InChI
**171**	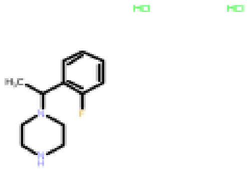	n.a.	1258649-78-3	Z2466069494	bmse011282	InChI=1S/C12H17FN2.2ClH/c1-10(15-8-6-14-7-9-15)11-4-2-3-5-12(11)13;;/h2-5,10,14H,6-9H2,1H3;2*1H/t10-;;/m0../s1
**172**	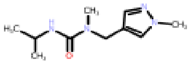	n.a.	1258705-03-1	Z183344018	bmse011515	InChI=1S/C10H18N4O/c1-8(2)12-10(15)13(3)6-9-5-11-14(4)7-9/h5,7-8H,6H2,1-4H3,(H,12,15)
**173**	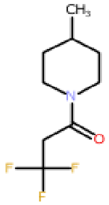	n.a.	1258720-49-8	Z316425948	bmse011503	InChI=1S/C9H14F3NO/c1-7-2-4-13(5-3-7)8(14)6-9(10,11)12/h7H,2-6H2,1H3
**174**	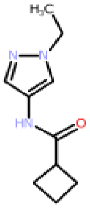	n.a.	1259209-34-1	Z373768900	bmse011511	InChI=1S/C10H15N3O/c1-2-13-7-9(6-11-13)12-10(14)8-4-3-5-8/h6-8H,2-5H2,1H3,(H,12,14)
**175**	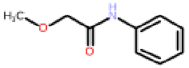	n.a.	126191-21-7	Z31504642	bmse011585	InChI=1S/C9H11NO2/c1-12-7-9(11)10-8-5-3-2-4-6-8/h2-6H,7H2,1H3,(H,10,11)

Suppl Table 2: Details of the 768 compounds from the DSI-Poised fragment Library (DSI-PL)

Sl. No.	Structures	No. in Manuscript	CAS Registry Number	"ID"	^{1}H NMR Data Repository BMRB ID	ALATIS InChI
**176**	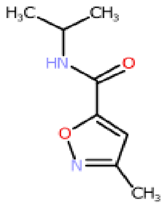	n.a.	126243-11-6	Z135394292	bmse011226	InChI=1S/C8H12N2O2/c1-5(2)9-8(11)7-4-6(3)10-12-7/h4-5H,1-3H3,(H,9,11)
**177**	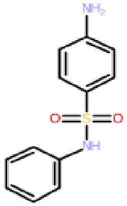	n.a.	127-77-5	Z56923284	bmse011436	InChI=1S/C12H12N2O2S/c13-10-6-8-12(9-7-10)17(15,16)14-11-4-2-1-3-5-11/h1-9,14H,13H2
**178**	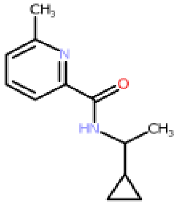	n.a.	1280825-17-3	Z287256168	bmse011597	InChI=1S/C12H16N2O/c1-8-4-3-5-11(13-8)12(15)14-9(2)10-6-7-10/h3-5,9-10H,6-7H2,1-2H3,(H,14,15)/t9-/m1/s1
**179**	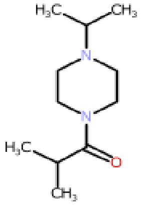	n.a.	1280837-87-7	Z106307058	bmse011601	InChI=1S/C11H22N2O/c1-9(2)11(14)13-7-5-12(6-8-13)10(3)4/h9-10H,5-8H2,1-4H3
**180**	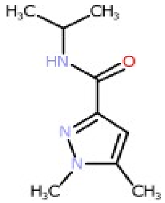	n.a.	1280844-52-1	Z733898630	bmse011599	InChI=1S/C9H15N3O/c1-6(2)10-9(13)8-5-7(3)12(4)11-8/h5-6H,1-4H3,(H,10,13)

Suppl Table 2: Details of the 768 compounds from the DSI-Poised fragment Library (DSI-PL)

Sl. No.	Structures	No. in Manuscript	CAS Registry Number	"ID"	^{1}H NMR Data Repository BMRB ID	ALATIS InChI
**181**	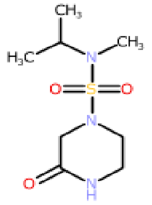	n.a.	1280851-40-2	Z431807512	bmse011233	InChI=1S/C8H17N3O3S/c1-7(2)10(3)15(13,14)11-5-4-9-8(12)6-11/h7H,4-6H2,1-3H3,(H,9,12)
**182**	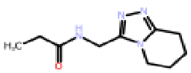	n.a.	1280931-26-1	Z438067480	bmse011243	InChI=1S/C10H16N4O/c1-2-10(15)11-7-9-13-12-8-5-3-4-6-14(8)9/h2-7H2,1H3,(H,11,15)
**183**	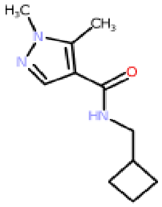	n.a.	1281013-68-0	Z396380540	bmse011248	InChI=1S/C11H17N3O/c1-8-10(7-13-14(8)2)11(15)12-6-9-4-3-5-9/h7,9H,3-6H2,1-2H3,(H,12,15)
**184**	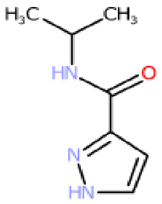	n.a.	1281118-68-0	Z381729066	bmse011224	InChI=1S/C7H11N3O/c1-5(2)9-7(11)6-3-4-8-10-6/h3-5H,1-2H3,(H,8,10)(H,9,11)
**185**	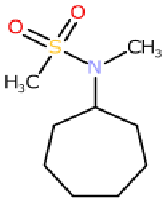	n.a.	1281160-88-0	Z216450634	bmse011507	InChI=1S/C9H19NO2S/c1-10(13(2,11)12)9-7-5-3-4-6-8-9/h9H,3-8H2,1-2H3

Suppl Table 2: Details of the 768 compounds from the DSI-Poised fragment Library (DSI-PL)

Sl. No.	Structures	No. in Manuscript	CAS Registry Number	"ID"	^{1}H NMR Data Repository BMRB ID	ALATIS InChI
**186**	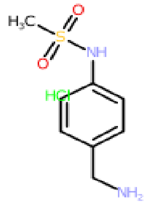	n.a.	128263-66-1	Z426041412	bmse011094	InChI=1S/C8H12N2O2S.ClH/c1-13(11,12)10-8-4-2-7(6-9)3-5-8;/h2-5,10H,6,9H2,1H3;1H
**187**	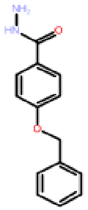	n.a.	128958-65-6	Z58982727	bmse011454	InChI=1S/C14H14N2O2/c15-16-14(17)12-6-8-13(9-7-12)18-10-11-4-2-1-3-5-11/h1-9H,10,15H2,(H,16,17)
**188**	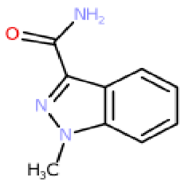	n.a.	129137-93-5	Z2697514548	bmse011754	InChI=1S/C9H9N3O/c1-12-7-5-3-2-4-6(7)8(11-12)9(10)13/h2-5H,1H3,(H2,10,13)
**189**	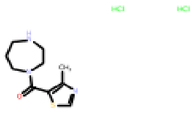	n.a.	1306606-53-0	Z803153598	bmse011172	InChI=1S/C10H15N3OS.2ClH/c1-8-9(15-7-12-8)10(14)13-5-2-3-11-4-6-13;;/h7,11H,2-6H2,1H3;2*1H
**190**	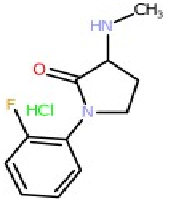	n.a.	1311313-52-6	Z1186029914	bmse011763	InChI=1S/C11H13FN2O.ClH/c1-13-9-6-7-14(11(9)15)10-5-3-2-4-8(10)12;/h2-5,9,13H,6-7H2,1H3;1H/t9-;/m0./s1

Suppl Table 2: Details of the 768 compounds from the DSI-Poised fragment Library (DSI-PL)

Sl. No.	Structures	No. in Manuscript	CAS Registry Number	"ID"	1H NMR Data Repository BMRB ID	ALATIS InChI
**191**	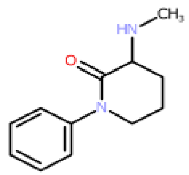	n.a.	1311313-94-6	Z1212984951	bmse011286	InChI=1S/C12H16N2O/c1-13-11-8-5-9-14(12(11)15)10-6-3-2-4-7-10/h2-4,6-7,11,13H,5,8-9H2,1H3/t11-/m0/s1
**192**	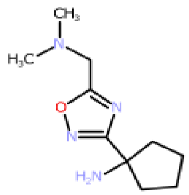	n.a.	1311318-30-5	Z1259339735	bmse011518	InChI=1S/C10H18N4O/c1-14(2)7-8-12-9(13-15-8)10(11)5-3-4-6-10/h3-7,11H2,1-2H3
**193**	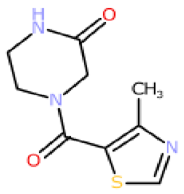	n.a.	1311779-47-1	Z422344882	bmse011096	InChI=1S/C9H11N3O2S/c1-6-8(15-5-11-6)9(14)12-3-2-10-7(13)4-12/h5H,2-4H2,1H3,(H,10,13)
**194**	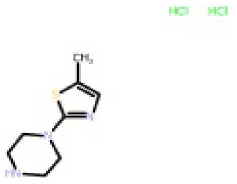	n.a.	1315368-08-1	Z1741975423	bmse011287	InChI=1S/C8H13N3S.2ClH/c1-7-6-10-8(12-7)11-4-2-9-3-5-11;;/h6,9H,2-5H2,1H3;2*1H
**195**	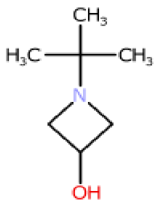	n.a.	13156-04-2	Z2856434900	bmse011510	InChI=1S/C7H15NO/c1-7(2,3)8-4-6(9)5-8/h6,9H,4-5H2,1-3H3

Suppl Table 2: Details of the 768 compounds from the DSI-Poised fragment Library (DSI-PL)

Sl. No.	Structures	No. in Manuscript	CAS Registry Number	"ID"	^{1}H NMR Data Repository BMRB ID	ALATIS InChI
**196**	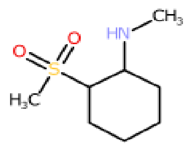	n.a.	131946-93-5	Z1259161657	bmse011520	InChI=1S/C8H17NO2S/c1-9-7-5-3-4-6-8(7)12(2,10)11/h7-9H,3-6H2,1-2H3/t7-,8-/m0/s1
**197**	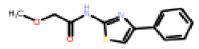	n.a.	132214-37-0	Z62645406	bmse011325	InChI=1S/C12H12N2O2S/c1-16-7-11(15)14-12-13-10(8-17-12)9-5-3-2-4-6-9/h2-6,8H,7H2,1H3,(H,13,14,15)
**198**	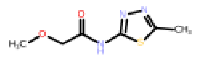	n.a.	132214-40-5	Z263785508	bmse011203	InChI=1S/C6H9N3O2S/c1-4-8-9-6(12-4)7-5(10)3-11-2/h3H2,1-2H3,(H,7,9,10)
**199**	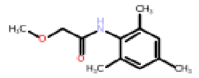	n.a.	133058-08-9	Z57260516	bmse011085	InChI=1S/C12H17NO2/c1-8-5-9(2)12(10(3)6-8)13-11(14)7-15-4/h5-6H,7H2,1-4H3,(H,13,14)
**200**	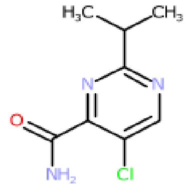	n.a.	1333872-58-4	Z906021418	bmse011255	InChI=1S/C8H10ClN3O/c1-4(2)8-11-3-5(9)6(12-8)7(10)13/h3-4H,1-2H3,(H2,10,13)

Suppl Table 2: Details of the 768 compounds from the DSI-Poised fragment Library (DSI-PL)

Sl. No.	Structures	No. in Manuscript	CAS Registry Number	"ID"	1H NMR Data Repository BMRB ID	ALATIS InChI
**201**	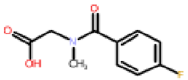	n.a.	133604-67-8	Z85895198	bmse011385	InChI=1S/C10H10FNO3/c1-12(6-9(13)14)10(15)7-2-4-8(11)5-3-7/h2-5H,6H2,1H3,(H,13,14)
**202**	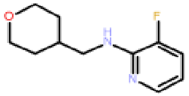	n.a.	1338988-79-6	Z1373430305	bmse011590	InChI=1S/C11H15FN2O/c12-10-2-1-5-13-11(10)14-8-9-3-6-15-7-4-9/h1-2,5,9H,3-4,6-8H2,(H,13,14)
**203**	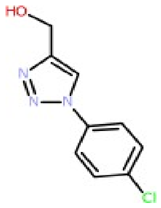	n.a.	133902-66-6	Z1374778753	bmse011397	InChI=1S/C9H8ClN3O/c10-7-1-3-9(4-2-7)13-5-8(6-14)11-12-13/h1-5,14H,6H2
**204**	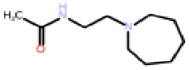	n.a.	1340208-96-9	Z169226638	bmse011569	InChI=1S/C10H20N2O/c1-10(13)11-6-9-12-7-4-2-3-5-8-12/h2-9H2,1H3,(H,11,13)
**205**	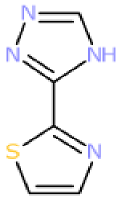	n.a.	1340241-97-5	Z2027049478	bmse011124	InChI=1S/C5H4N4S/c1-2-10-5(6-1)4-7-3-8-9-4/h1-3H,(H,7,8,9)

Suppl Table 2: Details of the 768 compounds from the DSI-Poised fragment Library (DSI-PL)

Sl. No.	Structures	No. in Manuscript	CAS Registry Number	"ID"	^{1}H NMR Data Repository BMRB ID	ALATIS InChI
**206**	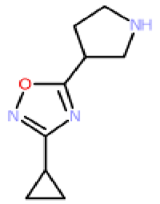	n.a.	1341019-84-8	Z2177153697	bmse011721	InChI=1S/C9H13N3O/c1-2-6(1)8-11-9(13-12-8)7-3-4-10-5-7/h6-7,10H,1-5H2/t7-/m0/s1
**207**	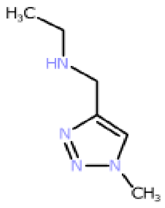	n.a.	1341638-33-2	Z1271660837	bmse011607	InChI=1S/C6H12N4/c1-3-7-4-6-5-10(2)9-8-6/h5,7H,3-4H2,1-2H3
**208**	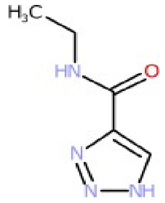	n.a.	1342762-99-5	Z755044716	bmse011417	InChI=1S/C5H8N4O/c1-2-6-5(10)4-3-7-9-8-4/h3H,2H2,1H3,(H,6,10)(H,7,8,9)
**209**	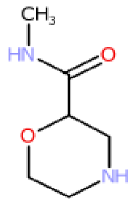	n.a.	135072-21-8	Z1636723439	bmse011460	InChI=1S/C6H12N2O2/c1-7-6(9)5-4-8-2-3-10-5/h5,8H,2-4H2,1H3,(H,7,9)/t5-/m1/s1
**210**	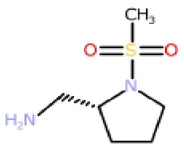	n.a.	1353897-54-7	Z1713595338	bmse011478	InChI=1S/C6H14N2O2S/c1-11(9,10)8-4-2-3-6(8)5-7/h6H,2-5,7H2,1H3/t6-/m1/s1

Suppl Table 2: Details of the 768 compounds from the DSI-Poised fragment Library (DSI-PL)

Sl. No.	Structures	No. in Manuscript	CAS Registry Number	"ID"	1H NMR Data Repository BMRB ID	ALATIS InChI
**211**	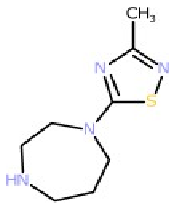	n.a.	1354961-32-2	Z1578665941	bmse011611	InChI=1S/C8H14N4S/c1-7-10-8(13-11-7)12-5-2-3-9-4-6-12/h9H,2-6H2,1H3
**212**	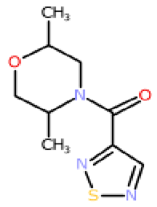	n.a.	1355694-70-0	Z768399682	bmse011506	InChI=1S/C9H13N3O2S/c1-6-5-14-7(2)4-12(6)9(13)8-3-10-15-11-8/h3,6-7H,4-5H2,1-2H3/t6-,7+/m0/s1
**213**	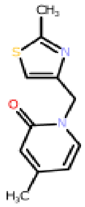	n.a.	1355889-65-4	Z1134990241	bmse011275	InChI=1S/C11H12N2OS/c1-8-3-4-13(11(14)5-8)6-10-7-15-9(2)12-10/h3-5,7H,6H2,1-2H3
**214**	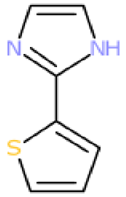	n.a.	136103-77-0	Z744754722	bmse011199	InChI=1S/C7H6N2S/c1-2-6(10-5-1)7-8-3-4-9-7/h1-5H,(H,8,9)
**215**	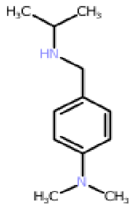	n.a.	137379-64-7	Z2856434894	bmse011509	InChI=1S/C12H20N2/c1-10(2)13-9-11-5-7-12(8-6-11)14(3)4/h5-8,10,13H,9H2,1-4H3

Suppl Table 2: Details of the 768 compounds from the DSI-Poised fragment Library (DSI-PL)

Sl. No.	Structures	No. in Manuscript	CAS Registry Number	"ID"	1H NMR Data Repository BMRB ID	ALATIS InChI
**216**	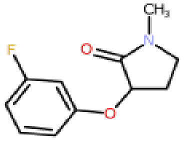	n.a.	1375168-80-1	Z1217960891	bmse011150	InChI=1S/C11H12FNO2/c1-13-6-5-10(11(13)14)15-9-4-2-3-8(12)7-9/h2-4,7,10H,5-6H2,1H3/t10-/m0/s1
**217**	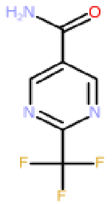	n.a.	1378340-42-1	Z1745658474	bmse011349	InChI=1S/C6H4F3N3O/c7-6(8,9)5-11-1-3(2-12-5)4(10)13/h1-2H,(H2,10,13)
**218**	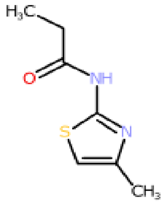	n.a.	13808-59-8	Z30820160	bmse011077	InChI=1S/C7H10N2OS/c1-3-6(10)9-7-8-5(2)4-11-7/h4H,3H2,1-2H3,(H,8,9,10)
**219**	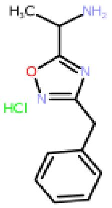	n.a.	1384428-19-6	Z2442288995	bmse011398	InChI=1S/C11H13N3O.ClH/c1-8(12)11-13-10(14-15-11)7-9-5-3-2-4-6-9;/h2-6,8H,7,12H2,1H3;1H/t8-;/m0./s1
**220**	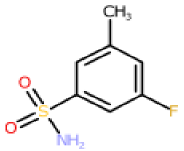	n.a.	1384429-42-8	Z1324080698	bmse011423	InChI=1S/C7H8FNO2S/c1-5-2-6(8)4-7(3-5)12(9,10)11/h2-4H,1H3,(H2,9,10,11)

Suppl Table 2: Details of the 768 compounds from the DSI-Poised fragment Library (DSI-PL)

Sl. No.	Structures	No. in Manuscript	CAS Registry Number	"ID"	^{1}H NMR Data Repository BMRB ID	ALATIS InChI
**221**	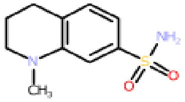	n.a.	1384429-63-3	Z1367324110	bmse011402	InChI=1S/C10H14N2O2S/c1-12-6-2-3-8-4-5-9(7-10(8)12)15(11,13)14/h4-5,7H,2-3,6H2,1H3,(H2,11,13,14)
**222**	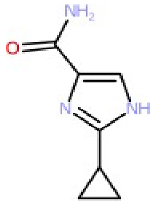	n.a.	1384430-46-9	Z1407672867	bmse011117	InChI=1S/C7H9N3O/c8-6(11)5-3-9-7(10-5)4-1-2-4/h3-4H,1-2H2,(H2,8,11)(H,9,10)
**223**	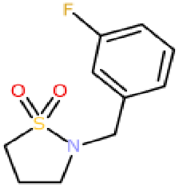	n.a.	1388691-56-2	Z729352906	bmse011562	InChI=1S/C10H12FNO2S/c11-10-4-1-3-9(7-10)8-12-5-2-6-15(12,13)14/h1,3-4,7H,2,5-6,8H2
**224**	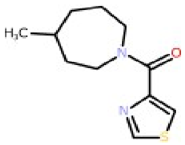	n.a.	1389617-98-4	Z1101755952	bmse011563	InChI=1S/C11H16N2OS/c1-9-3-2-5-13(6-4-9)11(14)10-7-15-8-12-10/h7-9H,2-6H2,1H3/t9-/m0/s1
**225**	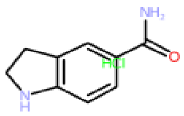	n.a.	1394041-29-2	Z2434225559	bmse011425	InChI=1S/C9H10N2O.ClH/c10-9(12)7-1-2-8-6(5-7)3-4-11-8;/h1-2,5,11H,3-4H2,(H2,10,12);1H

Suppl Table 2: Details of the 768 compounds from the DSI-Poised fragment Library (DSI-PL)

Sl. No.	Structures	No. in Manuscript	CAS Registry Number	"ID"	1H NMR Data Repository BMRB ID	ALATIS InChI
**226**	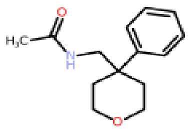	n.a.	14006-33-8	Z384468096	bmse011186	InChI=1S/C14H19NO2/c1-12(16)15-11-14(7-9-17-10-8-14)13-5-3-2-4-6-13/h2-6H,7-11H2,1H3,(H,15,16)
**227**	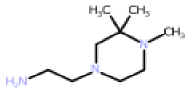	n.a.	1408418-82-5	Z1992316315	bmse011565	InChI=1S/C9H21N3/c1-9(2)8-12(5-4-10)7-6-11(9)3/h4-8,10H2,1-3H3
**228**	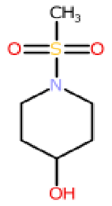	n.a.	141482-19-1	Z1247413608	bmse011391	InChI=1S/C6H13NO3S/c1-11(9,10)7-4-2-6(8)3-5-7/h6,8H,2-5H2,1H3
**229**	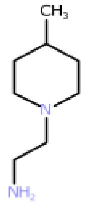	n.a.	14156-95-7	Z1245580461	bmse011546	InChI=1S/C8H18N2/c1-8-2-5-10(6-3-8)7-4-9/h8H,2-7,9H2,1H3
**230**	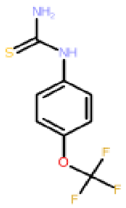	n.a.	142229-74-1	Z1741960769	bmse011755	InChI=1S/C8H7F3N2OS/c9-8(10,11)14-6-3-1-5(2-4-6)13-7(12)15/h1-4H,(H3,12,13,15)

Suppl Table 2: Details of the 768 compounds from the DSI-Poised fragment Library (DSI-PL)

Sl. No.	Structures	No. in Manuscript	CAS Registry Number	"ID"	^{1}H NMR Data Repository BMRB ID	ALATIS InChI
**231**	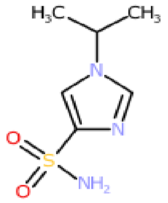	n.a.	1423029-76-8	Z1509882419	bmse011410	InChI=1S/C6H11N3O2S/c1-5(2)9-3-6(8-4-9)12(7,10)11/h3-5H,1-2H3,(H2,7,10,11)
**232**	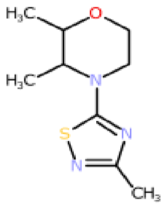	n.a.	1423709-57-2	Z1439422127	bmse011512	InChI=1S/C9H15N3OS/c1-6-7(2)13-5-4-12(6)9-10-8(3)11-14-9/h6-7H,4-5H2,1-3H3/t6-,7+/m0/s1
**233**	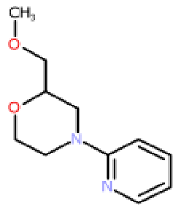	n.a.	1424464-95-8	Z1272480091	bmse011594	InChI=1S/C11H16N2O2/c1-14-9-10-8-13(6-7-15-10)11-4-2-3-5-12-11/h2-5,10H,6-9H2,1H3/t10-/m0/s1
**234**	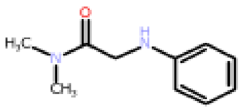	n.a.	14307-89-2	Z104584152	bmse011256	InChI=1S/C10H14N2O/c1-12(2)10(13)8-11-9-6-4-3-5-7-9/h3-7,11H,8H2,1-2H3
**235**	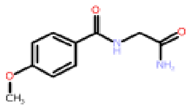	n.a.	143153-70-2	Z33546965	bmse011638	InChI=1S/C10H12N2O3/c1-15-8-4-2-7(3-5-8)10(14)12-6-9(11)13/h2-5H,6H2,1H3,(H2,11,13)(H,12,14)

Suppl Table 2: Details of the 768 compounds from the DSI-Poised fragment Library (DSI-PL)

Sl. No.	Structures	No. in Manuscript	CAS Registry Number	"ID"	^{1}H NMR Data Repository BMRB ID	ALATIS InChI
236	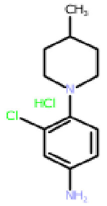	n.a.	1431965-45-5	Z57345491	bmse011760	InChI=1S/C12H17ClN2.ClH/c1-9-4-6-15(7-5-9)12-3-2-10(14)8-11(12)13;/h2-3,8-9H,4-7,14H2,1H3;1H
237	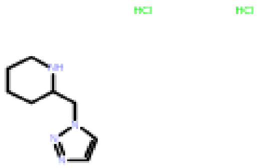	n.a.	1432679-74-7	Z1891776952	bmse011471	InChI=1S/C8H14N4.2ClH/c1-2-4-9-8(3-1)7-12-6-5-10-11-12;;/h5-6,8-9H,1-4,7H2;2*1H/t8-;;/m1../s1
238	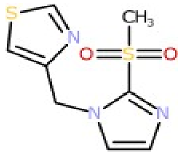	n.a.	1436099-85-2	Z1328968520	bmse011403	InChI=1S/C8H9N3O2S2/c1-15(12,13)8-9-2-3-11(8)4-7-5-14-6-10-7/h2-3,5-6H,4H2,1H3
239	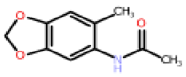	n.a.	1443980-28-6	Z1635496816	bmse011418	InChI=1S/C10H11NO3/c1-6-3-9-10(14-5-13-9)4-8(6)11-7(2)12/h3-4H,5H2,1-2H3,(H,11,12)
240	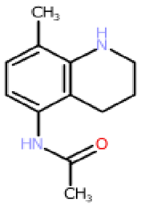	n.a.	1443980-33-3	Z1650168321	bmse011419	InChI=1S/C12H16N2O/c1-8-5-6-11(14-9(2)15)10-4-3-7-13-12(8)10/h5-6,13H,3-4,7H2,1-2H3,(H,14,15)

Suppl Table 2: Details of the 768 compounds from the DSI-Poised fragment Library (DSI-PL)

Sl. No.	Structures	No. in Manuscript	CAS Registry Number	"ID"	1H NMR Data Repository BMRB ID	ALATIS InChI
241	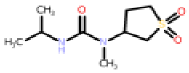	n.a.	1444157-22-5	Z445856640	bmse011288	InChI=1S/C9H18N2O3S/c1-7(2)10-9(12)11(3)8-4-5-15(13,14)6-8/h7-8H,4-6H2,1-3H3,(H,10,12)/t8-/m0/s1
242	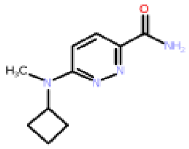	n.a.	1444314-07-1	Z1349163663	bmse011414	InChI=1S/C10H14N4O/c1-14(7-3-2-4-7)9-6-5-8(10(11)15)12-13-9/h5-7H,2-4H2,1H3,(H2,11,15)
243	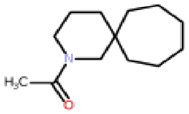	n.a.	1444594-73-3	Z1275599911	bmse011517	InChI=1S/C13H23NO/c1-12(15)14-10-6-9-13(11-14)7-4-2-3-5-8-13/h2-11H2,1H3
244	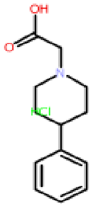	n.a.	1445133-23-2	Z287148438	bmse011415	InChI=1S/C13H17NO2.ClH/c15-13(16)10-14-8-6-12(7-9-14)11-4-2-1-3-5-11;/h1-5,12H,6-10H2,(H,15,16);1H
245	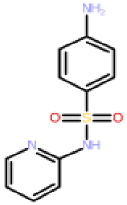	n.a.	144-83-2	Z271004858	bmse011054	InChI=1S/C11H11N3O2S/c12-9-4-6-10(7-5-9)17(15,16)14-11-3-1-2-8-13-11/h1-8H,12H2,(H,13,14)

Suppl Table 2: Details of the 768 compounds from the DSI-Poised fragment Library (DSI-PL)

Sl. No.	Structures	No. in Manuscript	CAS Registry Number	"ID"	^{1}H NMR Data Repository BMRB ID	ALATIS InChI
**246**	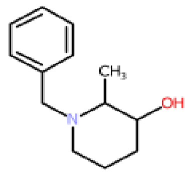	n.a.	1462379-90-3	Z2217052426	bmse011485	InChI=1S/C13H19NO/c1-11-13(15)8-5-9-14(11)10-12-6-3-2-4-7-12/h2-4,6-7,11,13,15H,5,8-10H2,1H3/t11-,13-/m0/s1
**247**	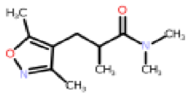	n.a.	1465302-15-1	Z1246465616	bmse011543	InChI=1S/C11H18N2O2/c1-7(11(14)13(4)5)6-10-8(2)12-15-9(10)3/h7H,6H2,1-5H3/t7-/m0/s1
**248**	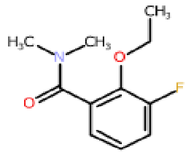	n.a.	1465358-31-9	Z1273312142	bmse011544	InChI=1S/C11H14FNO2/c1-4-15-10-8(11(14)13(2)3)6-5-7-9(10)12/h5-7H,4H2,1-3H3
**249**	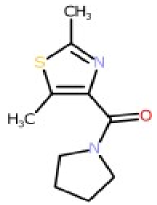	n.a.	1465360-84-2	Z1497321453	bmse011545	InChI=1S/C10H14N2OS/c1-7-9(11-8(2)14-7)10(13)12-5-3-4-6-12/h3-6H2,1-2H3
**250**	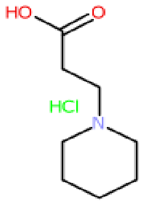	n.a.	14788-15-9	Z53115945	bmse011200	InChI=1S/C8H15NO2.ClH/c10-8(11)4-7-9-5-2-1-3-6-9;/h1-7H2,(H,10,11);1H

Suppl Table 2: Details of the 768 compounds from the DSI-Poised fragment Library (DSI-PL)

Sl. No.	Structures	No. in Manuscript	CAS Registry Number	"ID"	^{1}H NMR Data Repository BMRB ID	ALATIS InChI
**251**	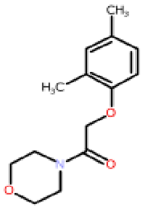	n.a.	148183-90-8	Z17497990	bmse011347	InChI=1S/C14H19NO3/c1-11-3-4-13(12(2)9-11)18-10-14(16)15-5-7-17-8-6-15/h3-4,9H,5-8,10H2,1-2H3
**252**	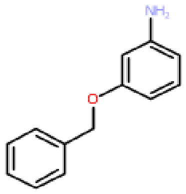	n.a.	1484-26-0	Z933326822	bmse011148	InChI=1S/C13H13NO/c14-12-7-4-8-13(9-12)15-10-11-5-2-1-3-6-11/h1-9H,10,14H2
**253**	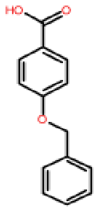	n.a.	1486-51-7	Z2856434845	bmse011433	InChI=1S/C14H12O3/c15-14(16)12-6-8-13(9-7-12)17-10-11-4-2-1-3-5-11/h1-9H,10H2,(H,15,16)
**254**	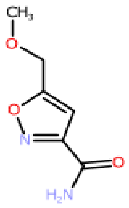	n.a.	1491320-49-0	Z1273141646	bmse011141	InChI=1S/C6H8N2O3/c1-10-3-4-2-5(6(7)9)8-11-4/h2H,3H2,1H3,(H2,7,9)
**255**	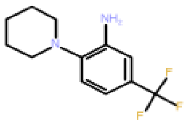	n.a.	1496-40-8	Z56792776	bmse011377	InChI=1S/C12H15F3N2/c13-12(14,15)9-4-5-11(10(16)8-9)17-6-2-1-3-7-17/h4-5,8H,1-3,6-7,16H2

Suppl Table 2: Details of the 768 compounds from the DSI-Poised fragment Library (DSI-PL)

Sl. No.	Structures	No. in Manuscript	CAS Registry Number	"ID"	^{1}H NMR Data Repository BMRB ID	ALATIS InChI
**256**	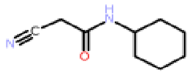	n.a.	15029-38-6	Z56813508	bmse011382	InChI=1S/C9H14N2O/c10-7-6-9(12)11-8-4-2-1-3-5-8/h8H,1-6H2,(H,11,12)
**257**	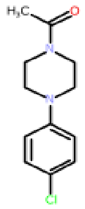	n.a.	150557-99-6	Z275165822	bmse011140	InChI=1S/C12H15ClN2O/c1-10(16)14-6-8-15(9-7-14)12-4-2-11(13)3-5-12/h2-5H,6-9H2,1H3
**258**	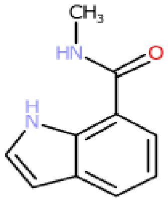	n.a.	1519477-17-8	Z1273312153	bmse011136	InChI=1S/C10H10N2O/c1-11-10(13)8-4-2-3-7-5-6-12-9(7)8/h2-6,12H,1H3,(H,11,13)
**259**	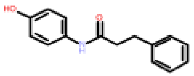	n.a.	152189-77-0	Z69118333	bmse011333	InChI=1S/C15H15NO2/c17-14-9-7-13(8-10-14)16-15(18)11-6-12-4-2-1-3-5-12/h1-5,7-10,17H,6,11H2,(H,16,18)
**260**	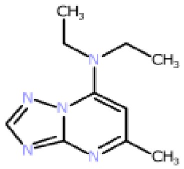	n.a.	15421-84-8	Z56791867	bmse011006	InChI=1S/C10H15N5/c1-4-14(5-2)9-6-8(3)13-10-11-7-12-15(9)10/h6-7H,4-5H2,1-3H3

Suppl Table 2: Details of the 768 compounds from the DSI-Poised fragment Library (DSI-PL)

Sl. No.	Structures	No. in Manuscript	CAS Registry Number	"ID"	^{1}H NMR Data Repository BMRB ID	ALATIS InChI
**261**	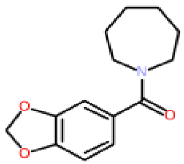	n.a.	154235-79-7	Z31432226	bmse011527	InChI=1S/C14H17NO3/c16-14(15-7-3-1-2-4-8-15)11-5-6-12-13(9-11)18-10-17-12/h5-6,9H,1-4,7-8,10H2
**262**	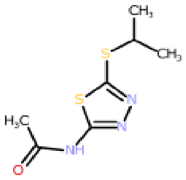	n.a.	154347-50-9	Z1134240160	bmse011178	InChI=1S/C7H11N3OS2/c1-4(2)12-7-10-9-6(13-7)8-5(3)11/h4H,1-3H3,(H,8,9,11)
**263**	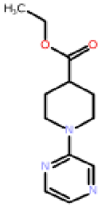	n.a.	154348-18-2	Z31217395	bmse011508	InChI=1S/C12H17N3O2/c1-2-17-12(16)10-3-7-15(8-4-10)11-9-13-5-6-14-11/h5-6,9-10H,2-4,7-8H2,1H3
**264**	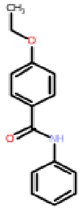	n.a.	15437-13-5	Z27782760	bmse011718	InChI=1S/C15H15NO2/c1-2-18-14-10-8-12(9-11-14)15(17)16-13-6-4-3-5-7-13/h3-11H,2H2,1H3,(H,16,17)
**265**	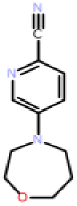	n.a.	1556733-66-4	Z1348371854	bmse011111	InChI=1S/C11H13N3O/c12-8-10-2-3-11(9-13-10)14-4-1-6-15-7-5-14/h2-3,9H,1,4-7H2

Suppl Table 2: Details of the 768 compounds from the DSI-Poised fragment Library (DSI-PL)

Sl. No.	Structures	No. in Manuscript	CAS Registry Number	"ID"	^{1}H NMR Data Repository BMRB ID	ALATIS InChI
**266**	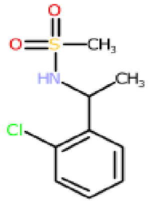	n.a.	1568201-21-7	Z133729708	bmse011430	InChI=1S/C9H12ClNO2S/c1-7(11-14(2,12)13)8-5-3-4-6-9(8)10/h3-7,11H,1-2H3/t7-/m1/s1
**267**	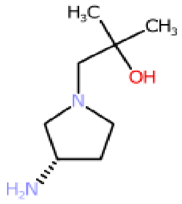	n.a.	1568206-90-5	Z2429435052	bmse011481	InChI=1S/C8H18N2O/c1-8(2,11)6-10-4-3-7(9)5-10/h7,11H,3-6,9H2,1-2H3/t7-/m0/s1
**268**	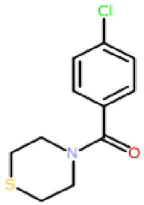	n.a.	158077-85-1	Z437584380	bmse011204	InChI=1S/C11H12ClNOS/c12-10-3-1-9(2-4-10)11(14)13-5-7-15-8-6-13/h1-4H,5-8H2
**269**	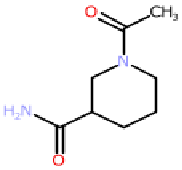	n.a.	15827-99-3	Z220816104	bmse011245	InChI=1S/C8H14N2O2/c1-6(11)10-4-2-3-7(5-10)8(9)12/h7H,2-5H2,1H3,(H2,9,12)/t7-/m0/s1
**270**	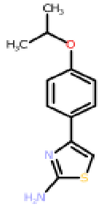	n.a.	15850-31-4	Z969560308	bmse011219	InChI=1S/C12H14N2OS/c1-8(2)15-10-5-3-9(4-6-10)11-7-16-12(13)14-11/h3-8H,1-2H3,(H2,13,14)

Suppl Table 2: Details of the 768 compounds from the DSI-Poised fragment Library (DSI-PL)

Sl. No.	Structures	No. in Manuscript	CAS Registry Number	"ID"	^{1}H NMR Data Repository BMRB ID	ALATIS InChI
**271**	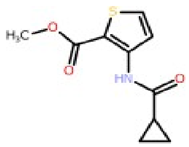	n.a.	158806-51-0	Z109092588	bmse011040	InChI=1S/C10H11NO3S/c1-14-10(13)8-7(4-5-15-8)11-9(12)6-2-3-6/h4-6H,2-3H2,1H3,(H,11,12)
**272**	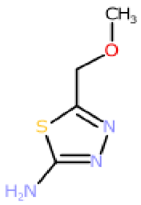	n.a.	15884-86-3	Z57478994	bmse011749	InChI=1S/C4H7N3OS/c1-8-2-3-6-7-4(5)9-3/h2H2,1H3,(H2,5,7)
**273**	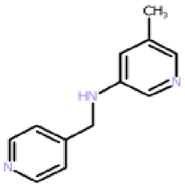	n.a.	1592679-07-6	Z1478435544	bmse011764	InChI=1S/C12H13N3/c1-10-6-12(9-14-7-10)15-8-11-2-4-13-5-3-11/h2-7,9,15H,8H2,1H3
**274**	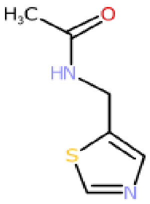	n.a.	1597981-65-1	Z1667545918	bmse011589	InChI=1S/C6H8N2OS/c1-5(9)8-3-6-2-7-4-10-6/h2,4H,3H2,1H3,(H,8,9)
**275**	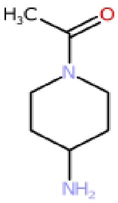	n.a.	160357-94-8	Z90664455	bmse011135	InChI=1S/C7H14N2O/c1-6(10)9-4-2-7(8)3-5-9/h7H,2-5,8H2,1H3

Suppl Table 2: Details of the 768 compounds from the DSI-Poised fragment Library (DSI-PL)

Sl. No.	Structures	No. in Manuscript	CAS Registry Number	"ID"	^{1}H NMR Data Repository BMRB ID	ALATIS InChI
**276**	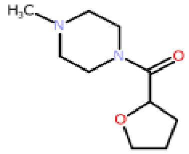	n.a.	1604302-01-3	Z31432964	bmse011756	InChI=1S/C10H18N2O2/c1-11-4-6-12(7-5-11)10(13)9-3-2-8-14-9/h9H,2-8H2,1H3/t9-/m1/s1
**277**	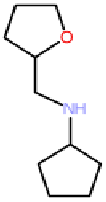	n.a.	1604372-25-9	Z90504169	bmse011535	InChI=1S/C10H19NO/c1-2-5-9(4-1)11-8-10-6-3-7-12-10/h9-11H,1-8H2/t10-/m1/s1
**278**	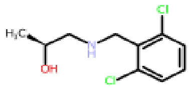	n.a.	1604441-87-3	Z2856434909	bmse011341	InChI=1S/C10H13Cl2NO/c1-7(14)5-13-6-8-9(11)3-2-4-10(8)12/h2-4,7,13-14H,5-6H2,1H3/t7-/m0/s1
**279**	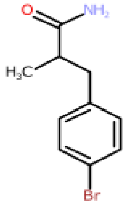	n.a.	1607316-72-2	Z1551999220	bmse011099	InChI=1S/C10H12BrNO/c1-7(10(12)13)6-8-2-4-9(11)5-3-8/h2-5,7H,6H2,1H3,(H2,12,13)/t7-/m1/s1
**280**	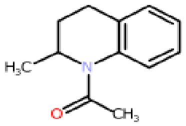	n.a.	16078-42-5	Z28870646	bmse011578	InChI=1S/C12H15NO/c1-9-7-8-11-5-3-4-6-12(11)13(9)10(2)14/h3-6,9H,7-8H2,1-2H3/t9-/m1/s1

Suppl Table 2: Details of the 768 compounds from the DSI-Poised fragment Library (DSI-PL)

Sl. No.	Structures	No. in Manuscript	CAS Registry Number	"ID"	^{1}H NMR Data Repository BMRB ID	ALATIS InChI
**281**	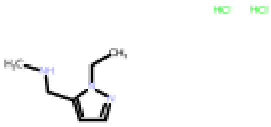	n.a.	1645405-95-3	Z2856434839	bmse011115	InChI=1S/C7H13N3.2ClH/c1-3-10-7(6-8-2)4-5-9-10;;/h4-5,8H,3,6H2,1-2H3;2*1H
**282**	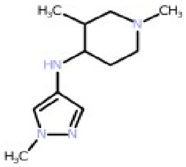	n.a.	1645500-53-3	Z1694504496	bmse011558	InChI=1S/C11H20N4/c1-9-7-14(2)5-4-11(9)13-10-6-12-15(3)8-10/h6,8-9,11,13H,4-5,7H2,1-3H3/t9-,11-/m0/s1
**283**	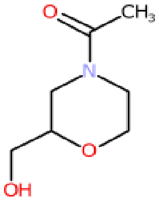	n.a.	1664380-98-6	Z1454840342	bmse011619	InChI=1S/C7H13NO3/c1-6(10)8-2-3-11-7(4-8)5-9/h7,9H,2-5H2,1H3/t7-/m0/s1
**284**	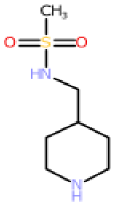	n.a.	166815-15-2	Z1741966151	bmse011125	InChI=1S/C7H16N2O2S/c1-12(10,11)9-6-7-2-4-8-5-3-7/h7-9H,2-6H2,1H3
**285**	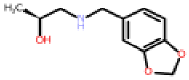	n.a.	1690164-82-9	Z2856434941	bmse011300	InChI=1S/C11H15NO3/c1-8(13)5-12-6-9-2-3-10-11(4-9)15-7-14-10/h2-4,8,12-13H,5-7H2,1H3/t8-/m0/s1

Suppl Table 2: Details of the 768 compounds from the DSI-Poised fragment Library (DSI-PL)

Sl. No.	Structures	No. in Manuscript	CAS Registry Number	"ID"	^{1}H NMR Data Repository BMRB ID	ALATIS InChI
**286**	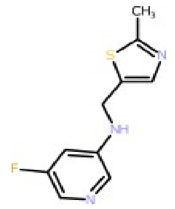	n.a.	1699685-98-7	Z1983897532	bmse011621	InChI=1S/C10H10FN3S/c1-7-13-5-10(15-7)6-14-9-2-8(11)3-12-4-9/h2-5,14H,6H2,1H3
**287**	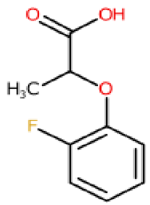	n.a.	17088-71-0	Z65532537	bmse011157	InChI=1S/C9H9FO3/c1-6(9(11)12)13-8-5-3-2-4-7(8)10/h2-6H,1H3,(H,11,12)/t6-/m0/s1
**288**	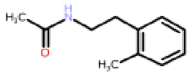	n.a.	171880-36-7	Z52314092	bmse011502	InChI=1S/C11H15NO/c1-9-5-3-4-6-11(9)7-8-12-10(2)13/h3-6H,7-8H2,1-2H3,(H,12,13)
**289**	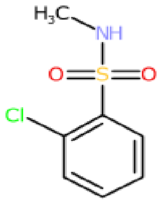	n.a.	17260-67-2	Z45527714	bmse011028	InChI=1S/C7H8ClNO2S/c1-9-12(10,11)7-5-3-2-4-6(7)8/h2-5,9H,1H3
**290**	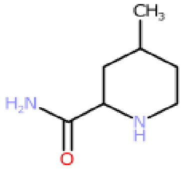	n.a.	172703-83-2	Z1741785925	bmse011104	InChI=1S/C7H14N2O/c1-5-2-3-9-6(4-5)7(8)10/h5-6,9H,2-4H2,1H3,(H2,8,10)/t5-,6-/m1/s1

Suppl Table 2: Details of the 768 compounds from the DSI-Poised fragment Library (DSI-PL)

Sl. No.	Structures	No. in Manuscript	CAS Registry Number	"ID"	^{1}H NMR Data Repository BMRB ID	ALATIS InChI
291	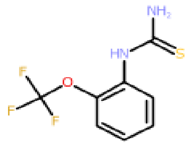	n.a.	175205-24-0	Z291279160	bmse011143	InChI=1S/C8H7F3N2OS/c9-8(10,11)14-6-4-2-1-3-5(6)13-7(12)15/h1-4H,(H3,12,13,15)
292	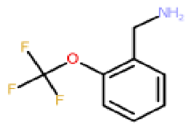	n.a.	175205-64-8	Z2856434776	bmse011584	InChI=1S/C8H8F3NO/c9-8(10,11)13-7-4-2-1-3-6(7)5-12/h1-4H,5,12H2
293	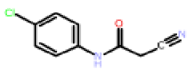	n.a.	17722-17-7	Z56837087	bmse011431	InChI=1S/C9H7ClN2O/c10-7-1-3-8(4-2-7)12-9(13)5-6-11/h1-4H,5H2,(H,12,13)
294	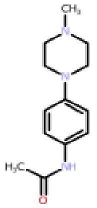	n.a.	17761-86-3	Z2856434903	bmse011145	InChI=1S/C13H19N3O/c1-11(17)14-12-3-5-13(6-4-12)16-9-7-15(2)8-10-16/h3-6H,7-10H2,1-2H3,(H,14,17)
295	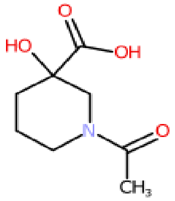	n.a.	1779359-88-4	Z2293643386	bmse011635	InChI=1S/C8H13NO4/c1-6(10)9-4-2-3-8(13,5-9)7(11)12/h13H,2-5H2,1H3,(H,11,12)/t8-/m0/s1

Suppl Table 2: Details of the 768 compounds from the DSI-Poised fragment Library (DSI-PL)

Sl. No.	Structures	No. in Manuscript	CAS Registry Number	"ID"	^{1}H NMR Data Repository BMRB ID	ALATIS InChI
**296**	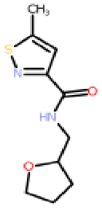	n.a.	1788976-17-9	Z1881545321	bmse011570	InChI=1S/C10H14N2O2S/c1-7-5-9(12-15-7)10(13)11-6-8-3-2-4-14-8/h5,8H,2-4,6H2,1H3,(H,11,13)/t8-/m1/s1
**297**	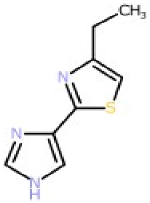	n.a.	1791353-66-6	Z1530301542	bmse011114	InChI=1S/C8H9N3S/c1-2-6-4-12-8(11-6)7-3-9-5-10-7/h3-5H,2H2,1H3,(H,9,10)
**298**	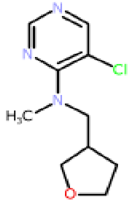	n.a.	1797334-69-0	Z1787627869	bmse011554	InChI=1S/C10H14ClN3O/c1-14(5-8-2-3-15-6-8)10-9(11)4-12-7-13-10/h4,7-8H,2-3,5-6H2,1H3/t8-/m1/s1
**299**	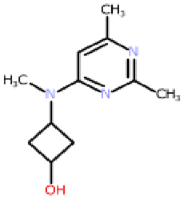	n.a.	1797358-96-3	Z1675346324	bmse011108	InChI=1S/C11H17N3O/c1-7-4-11(13-8(2)12-7)14(3)9-5-10(15)6-9/h4,9-10,15H,5-6H2,1-3H3/t9-,10-
**300**	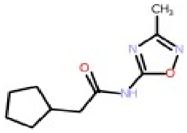	n.a.	1797931-68-0	Z729726784	bmse011229	InChI=1S/C10H15N3O2/c1-7-11-10(15-13-7)12-9(14)6-8-4-2-3-5-8/h8H,2-6H2,1H3,(H,11,12,13,14)

Suppl Table 2: Details of the 768 compounds from the DSI-Poised fragment Library (DSI-PL)

Sl. No.	Structures	No. in Manuscript	CAS Registry Number	"ID"	^{1}H NMR Data Repository BMRB ID	ALATIS InChI
**301**	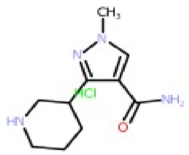	n.a.	1803589-18-5	Z2377835233	bmse011119	InChI=1S/C10H16N4O.ClH/c1-14-6-8(10(11)15)9(13-14)7-3-2-4-12-5-7;/h6-7,12H,2-5H2,1H3,(H2,11,15);1H/t7-;/m0./s1
**302**	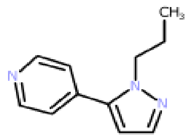	n.a.	1803589-88-9	Z1381484542	bmse011759	InChI=1S/C11H13N3/c1-2-9-14-11(5-8-13-14)10-3-6-12-7-4-10/h3-8H,2,9H2,1H3
**303**	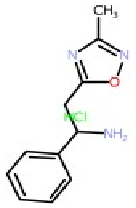	n.a.	1803590-55-7	Z2442269480	bmse011476	InChI=1S/C11H13N3O.ClH/c1-8-13-11(15-14-8)7-10(12)9-5-3-2-4-6-9;/h2-6,10H,7,12H2,1H3;1H/t10-;/m0./s1
**304**	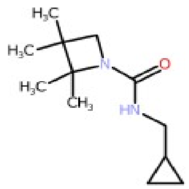	n.a.	1808326-78-4	Z1328078283	bmse011118	InChI=1S/C12H22N2O/c1-11(2)8-14(12(11,3)4)10(15)13-7-9-5-6-9/h9H,5-8H2,1-4H3,(H,13,15)
**305**	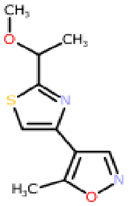	n.a.	1808426-16-5	Z1945710531	bmse011568	InChI=1S/C10H12N2O2S/c1-6-8(4-11-14-6)9-5-15-10(12-9)7(2)13-3/h4-5,7H,1-3H3/t7-/m0/s1

Suppl Table 2: Details of the 768 compounds from the DSI-Poised fragment Library (DSI-PL)

Sl. No.	Structures	No. in Manuscript	CAS Registry Number	"ID"	^{1}H NMR Data Repository BMRB ID	ALATIS InChI
**306**	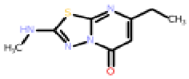	n.a.	1808817-91-5	Z1896598013	bmse011123	InChI=1S/C8H10N4OS/c1-3-5-4-6(13)12-8(10-5)14-7(9-2)11-12/h4H,3H2,1-2H3,(H,9,11)
**307**	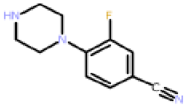	n.a.	182181-38-0	Z939944666	bmse011213	InChI=1S/C11H12FN3/c12-10-7-9(8-13)1-2-11(10)15-5-3-14-4-6-15/h1-2,7,14H,3-6H2
**308**	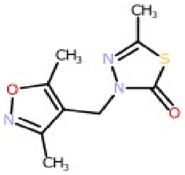	n.a.	1825462-54-1	Z1899842917	bmse011572	InChI=1S/C9H11N3O2S/c1-5-8(6(2)14-11-5)4-12-9(13)15-7(3)10-12/h4H2,1-3H3
**309**	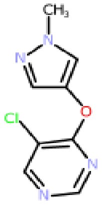	n.a.	1825505-53-0	Z1918536193	bmse011577	InChI=1S/C8H7ClN4O/c1-13-4-6(2-12-13)14-8-7(9)3-10-5-11-8/h2-5H,1H3
**310**	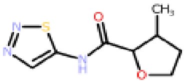	n.a.	1825622-15-8	Z1827602749	bmse011128	InChI=1S/C8H11N3O2S/c1-5-2-3-13-7(5)8(12)10-6-4-9-11-14-6/h4-5,7H,2-3H2,1H3,(H,10,12)/t5-,7+/m0/s1

Suppl Table 2: Details of the 768 compounds from the DSI-Poised fragment Library (DSI-PL)

Sl. No.	Structures	No. in Manuscript	CAS Registry Number	"ID"	1H NMR Data Repository BMRB ID	ALATIS InChI
**311**	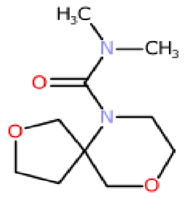	n.a.	1825684-74-9	Z1998104358	bmse011608	InChI=1S/C10H18N2O3/c1-11(2)9(13)12-4-6-15-8-10(12)3-5-14-7-10/h3-8H2,1-2H3/t10-/m1/s1
**312**	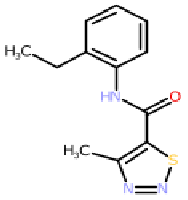	n.a.	183306-35-6	Z89385775	bmse011350	InChI=1S/C12H13N3OS/c1-3-9-6-4-5-7-10(9)13-12(16)11-8(2)14-15-17-11/h4-7H,3H2,1-2H3,(H,13,16)
**313**	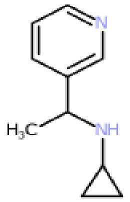	n.a.	183609-12-3	Z1263529721	bmse011542	InChI=1S/C10H14N2/c1-8(12-10-4-5-10)9-3-2-6-11-7-9/h2-3,6-8,10,12H,4-5H2,1H3/t8-/m1/s1
**314**	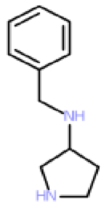	n.a.	184107-56-0	Z1827898537	bmse011555	InChI=1S/C11H16N2/c1-2-4-10(5-3-1)8-13-11-6-7-12-9-11/h1-5,11-13H,6-9H2/t11-/m1/s1
**315**	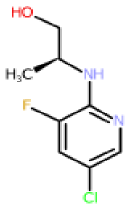	n.a.	1841274-96-1	Z1432018343	bmse011169	InChI=1S/C8H10ClFN2O/c1-5(4-13)12-8-7(10)2-6(9)3-11-8/h2-3,5,13H,4H2,1H3,(H,11,12)/t5-/m0/s1

Suppl Table 2: Details of the 768 compounds from the DSI-Poised fragment Library (DSI-PL)

Sl. No.	Structures	No. in Manuscript	CAS Registry Number	"ID"	1H NMR Data Repository BMRB ID	ALATIS InChI
**316**	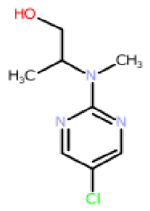	n.a.	1845036-67-0	Z1546887028	bmse011547	InChI=1S/C8H12ClN3O/c1-6(5-13)12(2)8-10-3-7(9)4-11-8/h3-4,6,13H,5H2,1-2H3/t6-/m1/s1
**317**	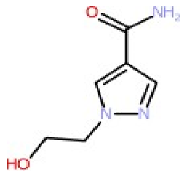	n.a.	1849283-80-2	Z1562205518	bmse011160	InChI=1S/C6H9N3O2/c7-6(11)5-3-8-9(4-5)1-2-10/h3-4,10H,1-2H2,(H2,7,11)
**318**	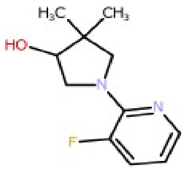	n.a.	1853078-72-4	Z1929757385	bmse011620	InChI=1S/C11H15FN2O/c1-11(2)7-14(6-9(11)15)10-8(12)4-3-5-13-10/h3-5,9,15H,6-7H2,1-2H3/t9-/m0/s1
**319**	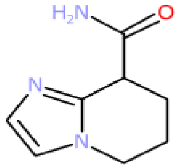	n.a.	185797-36-8	Z1324853681	bmse011404	InChI=1S/C8H11N3O/c9-7(12)6-2-1-4-11-5-3-10-8(6)11/h3,5-6H,1-2,4H2,(H2,9,12)/t6-/m0/s1
**320**	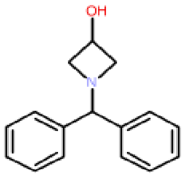	n.a.	18621-17-5	Z1741964527	bmse011097	InChI=1S/C16H17NO/c18-15-11-17(12-15)16(13-7-3-1-4-8-13)14-9-5-2-6-10-14/h1-10,15-16,18H,11-12H2

Suppl Table 2: Details of the 768 compounds from the DSI-Poised fragment Library (DSI-PL)

Sl. No.	Structures	No. in Manuscript	CAS Registry Number	"ID"	^{1}H NMR Data Repository BMRB ID	ALATIS InChI
**321**	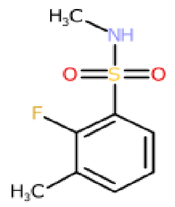	n.a.	1864268-87-0	Z1639162606	bmse011166	InChI=1S/C8H10FNO2S/c1-6-4-3-5-7(8(6)9)13(11,12)10-2/h3-5,10H,1-2H3
**322**	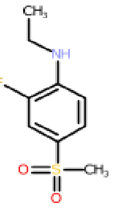	n.a.	1864906-89-7	Z2142244288	bmse011170	InChI=1S/C9H12FNO2S/c1-3-11-9-5-4-7(6-8(9)10)14(2,12)13/h4-6,11H,3H2,1-2H3
**323**	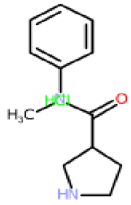	n.a.	1867579-24-5	Z2447286438	bmse011122	InChI=1S/C12H16N2O.ClH/c1-14(11-5-3-2-4-6-11)12(15)10-7-8-13-9-10;/h2-6,10,13H,7-9H2,1H3;1H/t10-;/m0./s1
**324**	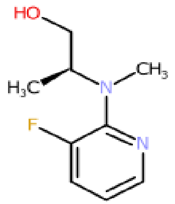	n.a.	1867629-67-1	Z2017168803	bmse011616	InChI=1S/C9H13FN2O/c1-7(6-13)12(2)9-8(10)4-3-5-11-9/h3-5,7,13H,6H2,1-2H3/t7-/m0/s1
**325**	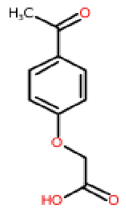	n.a.	1878-81-5	Z2856434937	bmse011435	InChI=1S/C10H10O4/c1-7(11)8-2-4-9(5-3-8)14-6-10(12)13/h2-5H,6H2,1H3,(H,12,13)

Suppl Table 2: Details of the 768 compounds from the DSI-Poised fragment Library (DSI-PL)

Sl. No.	Structures	No. in Manuscript	CAS Registry Number	"ID"	^{1}H NMR Data Repository BMRB ID	ALATIS InChI
**326**	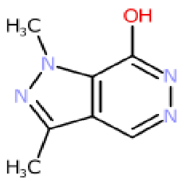	n.a.	1909337-15-0	Z2301438417	bmse011139	InChI=1S/C7H8N4O/c1-4-5-3-8-9-7(12)6(5)11(2)10-4/h3H,1-2H3,(H,9,12)
**327**	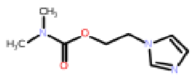	n.a.	191539-21-6	Z1722836661	bmse011630	InChI=1S/C8H13N3O2/c1-10(2)8(12)13-6-5-11-4-3-9-7-11/h3-4,7H,5-6H2,1-2H3
**328**	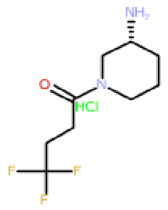	n.a.	1941189-57-6	Z1818332938	bmse011768	InChI=1S/C9H15F3N2O.ClH/c10-9(11,12)4-3-8(15)14-5-1-2-7(13)6-14;/h7H,1-6,13H2;1H/t7-;/m1./s1
**329**	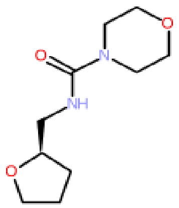	n.a.	1941215-86-6	Z2064898127	bmse011636	InChI=1S/C10H18N2O3/c13-10(12-3-6-14-7-4-12)11-8-9-2-1-5-15-9/h9H,1-8H2,(H,11,13)/t9-/m1/s1
**330**	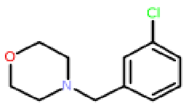	n.a.	196792-37-7	Z2856434856	bmse011514	InChI=1S/C11H14ClNO/c12-11-3-1-2-10(8-11)9-13-4-6-14-7-5-13/h1-3,8H,4-7,9H2

Suppl Table 2: Details of the 768 compounds from the DSI-Poised fragment Library (DSI-PL)

Sl. No.	Structures	No. in Manuscript	CAS Registry Number	"ID"	^{1}H NMR Data Repository BMRB ID	ALATIS InChI
**331**	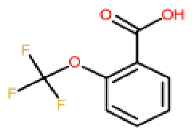	n.a.	1979-29-9	Z2856434778	bmse011758	InChI=1S/C8H5F3O3/c9-8(10,11)14-6-4-2-1-3-5(6)7(12)13/h1-4H,(H,12,13)
**332**	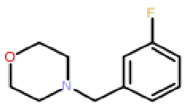	n.a.	198879-71-9	Z2856434791	bmse011505	InChI=1S/C11H14FNO/c12-11-3-1-2-10(8-11)9-13-4-6-14-7-5-13/h1-3,8H,4-7,9H2
**333**	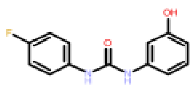	n.a.	199585-00-7	Z55290386	bmse011278	InChI=1S/C13H11FN2O2/c14-9-4-6-10(7-5-9)15-13(18)16-11-2-1-3-12(17)8-11/h1-8,17H,(H2,15,16,18)
**334**	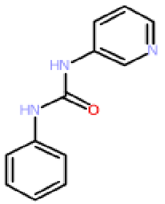	n.a.	2000-55-7	Z44592329	bmse011032	InChI=1S/C12H11N3O/c16-12(14-10-5-2-1-3-6-10)15-11-7-4-8-13-9-11/h1-9H,(H2,14,15,16)
**335**	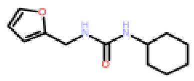	n.a.	200058-87-3	Z44584192	bmse011674	InChI=1S/C12H18N2O2/c15-12(13-9-11-7-4-8-16-11)14-10-5-2-1-3-6-10/h4,7-8,10H,1-3,5-6,9H2,(H2,13,14,15)

Suppl Table 2: Details of the 768 compounds from the DSI-Poised fragment Library (DSI-PL)

Sl. No.	Structures	No. in Manuscript	CAS Registry Number	"ID"	^{1}H NMR Data Repository BMRB ID	ALATIS InChI
**336**	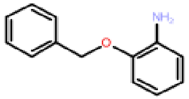	n.a.	20012-63-9	Z336089202	bmse011065	InChI=1S/C13H13NO/c14-12-8-4-5-9-13(12)15-10-11-6-2-1-3-7-11/h1-9H,10,14H2
**337**	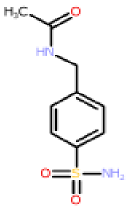	n.a.	2015-14-7	Z30932204	bmse011379	InChI=1S/C9H12N2O3S/c1-7(12)11-6-8-2-4-9(5-3-8)15(10,13)14/h2-5H,6H2,1H3,(H,11,12)(H2,10,13,14)
**338**	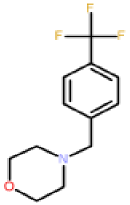	n.a.	201682-13-5	Z2856434871	bmse011493	InChI=1S/C12H14F3NO/c13-12(14,15)11-3-1-10(2-4-11)9-16-5-7-17-8-6-16/h1-4H,5-9H2
**339**	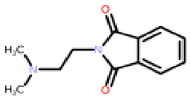	n.a.	20320-52-9	Z2856434895	bmse011378	InChI=1S/C12H14N2O2/c1-13(2)7-8-14-11(15)9-5-3-4-6-10(9)12(14)16/h3-6H,7-8H2,1-2H3
**340**	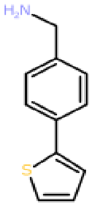	n.a.	203436-48-0	Z2856434805	bmse011477	InChI=1S/C11H11NS/c12-8-9-3-5-10(6-4-9)11-2-1-7-13-11/h1-7H,8,12H2

Suppl Table 2: Details of the 768 compounds from the DSI-Poised fragment Library (DSI-PL)

Sl. No.	Structures	No. in Manuscript	CAS Registry Number	"ID"	^{1}H NMR Data Repository BMRB ID	ALATIS InChI
**341**	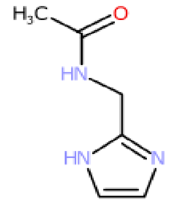	n.a.	203664-03-3	Z1203191681	bmse011167	InChI=1S/C6H9N3O/c1-5(10)9-4-6-7-2-3-8-6/h2-3H,4H2,1H3,(H,7,8)(H,9,10)
**342**	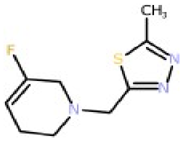	n.a.	2093752-67-9	Z1619978933	bmse011617	InChI=1S/C9H12FN3S/c1-7-11-12-9(14-7)6-13-4-2-3-8(10)5-13/h3H,2,4-6H2,1H3
**343**	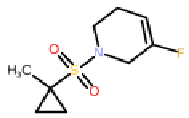	n.a.	2093848-33-8	Z2277255954	bmse011614	InChI=1S/C9H14FNO2S/c1-9(4-5-9)14(12,13)11-6-2-3-8(10)7-11/h3H,2,4-7H2,1H3
**344**	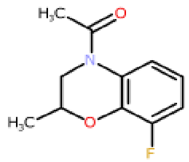	n.a.	2093849-83-1	Z1730522163	bmse011168	InChI=1S/C11H12FNO2/c1-7-6-13(8(2)14)10-5-3-4-9(12)11(10)15-7/h3-5,7H,6H2,1-2H3/t7-/m1/s1
**345**	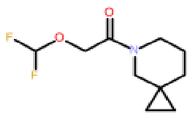	n.a.	2093871-60-2	Z2074076908	bmse011615	InChI=1S/C10H15F2NO2/c11-9(12)15-6-8(14)13-5-1-2-10(7-13)3-4-10/h9H,1-7H2

Suppl Table 2: Details of the 768 compounds from the DSI-Poised fragment Library (DSI-PL)

Sl. No.	Structures	No. in Manuscript	CAS Registry Number	"ID"	^{1}H NMR Data Repository BMRB ID	ALATIS InChI
**346**	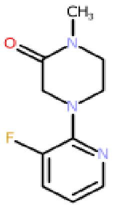	n.a.	2093905-71-4	Z1373445602	bmse011479	InChI=1S/C10H12FN3O/c1-13-5-6-14(7-9(13)15)10-8(11)3-2-4-12-10/h2-4H,5-7H2,1H3
**347**	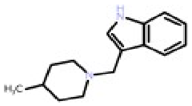	n.a.	21000-95-3	Z2856434912	bmse011720	InChI=1S/C15H20N2/c1-12-6-8-17(9-7-12)11-13-10-16-15-5-3-2-4-14(13)15/h2-5,10,12,16H,6-9,11H2,1H3
**348**	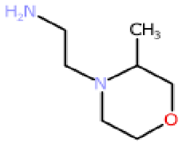	n.a.	2101221-33-2	Z1263811695	bmse011537	InChI=1S/C7H16N2O/c1-7-6-10-5-4-9(7)3-2-8/h7H,2-6,8H2,1H3/t7-/m0/s1
**349**	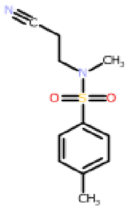	n.a.	21230-34-2	Z45515569	bmse011359	InChI=1S/C11H14N2O2S/c1-10-4-6-11(7-5-10)16(14,15)13(2)9-3-8-12/h4-7H,3,9H2,1-2H3
**350**	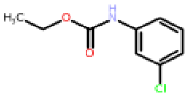	n.a.	2150-89-2	Z31697001	bmse011645	InChI=1S/C9H10ClNO2/c1-2-13-9(12)11-8-5-3-4-7(10)6-8/h3-6H,2H2,1H3,(H,11,12)

Suppl Table 2: Details of the 768 compounds from the DSI-Poised fragment Library (DSI-PL)

Sl. No.	Structures	No. in Manuscript	CAS Registry Number	"ID"	^{1}H NMR Data Repository BMRB ID	ALATIS InChI
**351**	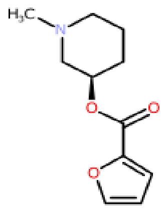	n.a.	2155840-31-4	Z2940170964	bmse011750	InChI=1S/C11H15NO3/c1-12-6-2-4-9(8-12)15-11(13)10-5-3-7-14-10/h3,5,7,9H,2,4,6,8H2,1H3/t9-/m1/s1
**352**	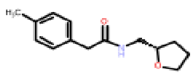	n.a.	2155840-32-5	Z2064898339	bmse011465	InChI=1S/C14H19NO2/c1-11-4-6-12(7-5-11)9-14(16)15-10-13-3-2-8-17-13/h4-7,13H,2-3,8-10H2,1H3,(H,15,16)/t13-/m0/s1
**353**	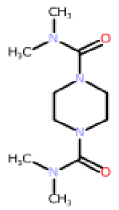	n.a.	21579-91-9	Z57475068	bmse011387	InChI=1S/C10H20N4O2/c1-11(2)9(15)13-5-7-14(8-6-13)10(16)12(3)4/h5-8H2,1-4H3
**354**	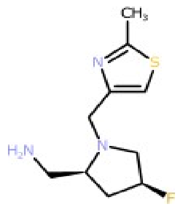	n.a.	2165541-85-3	Z2444997446	bmse011752	InChI=1S/C10H16FN3S/c1-7-13-9(6-15-7)5-14-4-8(11)2-10(14)3-12/h6,8,10H,2-5,12H2,1H3/t8-,10-/m0/s1
**355**	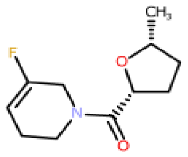	n.a.	2167674-93-1	Z2272040604	bmse011622	InChI=1S/C11H16FNO2/c1-8-4-5-10(15-8)11(14)13-6-2-3-9(12)7-13/h3,8,10H,2,4-7H2,1H3/t8-,10-/m1/s1

Suppl Table 2: Details of the 768 compounds from the DSI-Poised fragment Library (DSI-PL)

Sl. No.	Structures	No. in Manuscript	CAS Registry Number	"ID"	^{1}H NMR Data Repository BMRB ID	ALATIS InChI
**356**	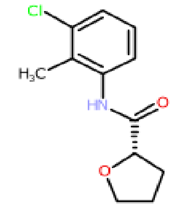	n.a.	2176362-38-0	Z1545312521	bmse011651	InChI=1S/C12H14ClNO2/c1-8-9(13)4-2-5-10(8)14-12(15)11-6-3-7-16-11/h2,4-5,11H,3,6-7H2,1H3,(H,14,15)/t11-/m0/s1
**357**	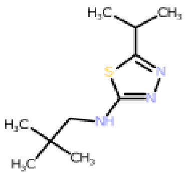	n.a.	218795-46-1	Z1461982627	bmse011688	InChI=1S/C10H19N3S/c1-7(2)8-12-13-9(14-8)11-6-10(3,4)5/h7H,6H2,1-5H3,(H,11,13)
**358**	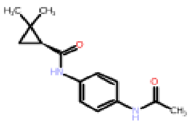	n.a.	2193725-87-8	Z1505719304	bmse011338	InChI=1S/C14H18N2O2/c1-9(17)15-10-4-6-11(7-5-10)16-13(18)12-8-14(12,2)3/h4-7,12H,8H2,1-3H3,(H,15,17)(H,16,18)/t12-/m1/s1
**359**	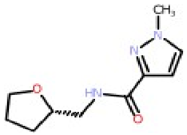	n.a.	2196041-45-7	Z2643472210	bmse011742	InChI=1S/C10H15N3O2/c1-13-5-4-9(12-13)10(14)11-7-8-3-2-6-15-8/h4-5,8H,2-3,6-7H2,1H3,(H,11,14)/t8-/m0/s1
**360**	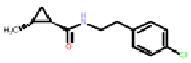	n.a.	2223091-39-0	Z1802166390	bmse011317	InChI=1S/C13H16ClNO/c1-9-8-12(9)13(16)15-7-6-10-2-4-11(14)5-3-10/h2-5,9,12H,6-8H2,1H3,(H,15,16)/t9-,12-/m1/s1

Suppl Table 2: Details of the 768 compounds from the DSI-Poised fragment Library (DSI-PL)

Sl. No.	Structures	No. in Manuscript	CAS Registry Number	"ID"	1H NMR Data Repository BMRB ID	ALATIS InChI
**361**	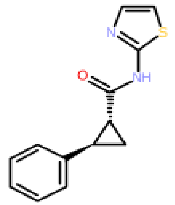	n.a.	2223103-00-0	Z2064107709	bmse011675	InChI=1S/C13H12N2OS/c16-12(15-13-14-6-7-17-13)11-8-10(11)9-4-2-1-3-5-9/h1-7,10-11H,8H2,(H,14,15,16)/t10-,11+/m0/s1
**362**	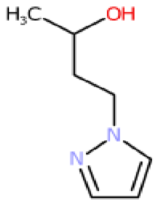	n.a.	2227878-17-1	Z1613492358	bmse011519	InChI=1S/C7H12N2O/c1-7(10)3-6-9-5-2-4-8-9/h2,4-5,7,10H,3,6H2,1H3/t7-/m0/s1
**363**	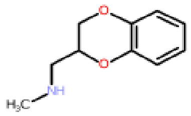	n.a.	2242-31-1	Z1891773476	bmse011441	InChI=1S/C10H13NO2/c1-11-6-8-7-12-9-4-2-3-5-10(9)13-8/h2-5,8,11H,6-7H2,1H3/t8-/m1/s1
**364**	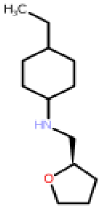	n.a.	2271775-80-3	Z2856434866	bmse011641	InChI=1S/C13H25NO/c1-2-11-5-7-12(8-6-11)14-10-13-4-3-9-15-13/h11-14H,2-10H2,1H3/t11-,12-,13-/m1/s1
**365**	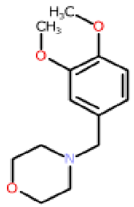	n.a.	22792-30-9	Z2856434878	bmse011678	InChI=1S/C13H19NO3/c1-15-12-4-3-11(9-13(12)16-2)10-14-5-7-17-8-6-14/h3-4,9H,5-8,10H2,1-2H3

Suppl Table 2: Details of the 768 compounds from the DSI-Poised fragment Library (DSI-PL)

Sl. No.	Structures	No. in Manuscript	CAS Registry Number	"ID"	^{1}H NMR Data Repository BMRB ID	ALATIS InChI
**366**	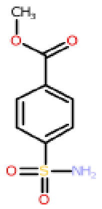	n.a.	22808-73-7	Z18197050	bmse011366	InChI=1S/C8H9NO4S/c1-13-8(10)6-2-4-7(5-3-6)14(9,11)12/h2-5H,1H3,(H2,9,11,12)
**367**	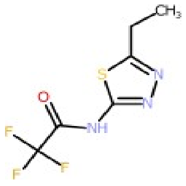	n.a.	22926-50-7	Z275156196	bmse011356	InChI=1S/C6H6F3N3OS/c1-2-3-11-12-5(14-3)10-4(13)6(7,8)9/h2H2,1H3,(H,10,12,13)
**368**	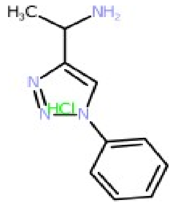	n.a.	2322927-70-6	Z2527301677	bmse011405	InChI=1S/C10H12N4.ClH/c1-8(11)10-7-14(13-12-10)9-5-3-2-4-6-9;/h2-8H,11H2,1H3;1H/t8-;/m0./s1
**369**	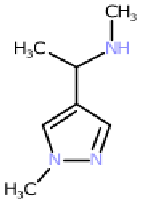	n.a.	2322929-21-3	Z1437171658	bmse011524	InChI=1S/C7H13N3/c1-6(8-2)7-4-9-10(3)5-7/h4-6,8H,1-3H3/t6-/m0/s1
**370**	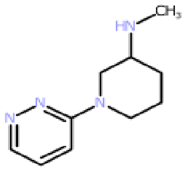	n.a.	2322929-33-7	Z1139246057	bmse011536	InChI=1S/C10H16N4/c1-11-9-4-3-7-14(8-9)10-5-2-6-12-13-10/h2,5-6,9,11H,3-4,7-8H2,1H3/t9-/m0/s1

Suppl Table 2: Details of the 768 compounds from the DSI-Poised fragment Library (DSI-PL)

Sl. No.	Structures	No. in Manuscript	CAS Registry Number	"ID"	^{1}H NMR Data Repository BMRB ID	ALATIS InChI
**371**	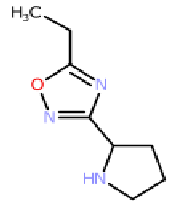	n.a.	2323067-86-1	Z1350579414	bmse011539	InChI=1S/C8H13N3O/c1-2-7-10-8(11-12-7)6-4-3-5-9-6/h6,9H,2-5H2,1H3/t6-/m1/s1
**372**	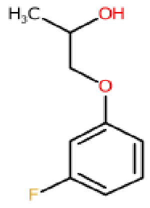	n.a.	2366-93-0	Z1250132544	bmse011534	InChI=1S/C9H11FO2/c1-7(11)6-12-9-4-2-3-8(10)5-9/h2-5,7,11H,6H2,1H3/t7-/m0/s1
**373**	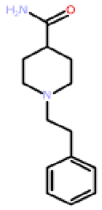	n.a.	23793-55-7	Z2856434897	bmse011061	InChI=1S/C14H20N2O/c15-14(17)13-7-10-16(11-8-13)9-6-12-4-2-1-3-5-12/h1-5,13H,6-11H2,(H2,15,17)
**374**	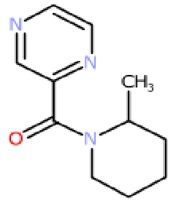	n.a.	2381174-54-3	Z32968340	bmse011613	InChI=1S/C11H15N3O/c1-9-4-2-3-7-14(9)11(15)10-8-12-5-6-13-10/h5-6,8-9H,2-4,7H2,1H3/t9-/m0/s1
**375**	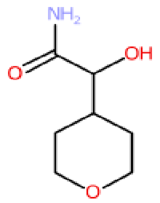	n.a.	2382445-52-3	Z1312590981	bmse011427	InChI=1S/C7H13NO3/c8-7(10)6(9)5-1-3-11-4-2-5/h5-6,9H,1-4H2,(H2,8,10)/t6-/m1/s1

Suppl Table 2: Details of the 768 compounds from the DSI-Poised fragment Library (DSI-PL)

Sl. No.	Structures	No. in Manuscript	CAS Registry Number	"ID"	^{1}H NMR Data Repository BMRB ID	ALATIS InChI
**376**	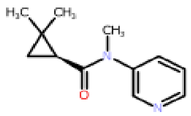	n.a.	2384436-47-7	Z1506050651	bmse011484	InChI=1S/C12H16N2O/c1-12(2)7-10(12)11(15)14(3)9-5-4-6-13-8-9/h4-6,8,10H,7H2,1-3H3/t10-/m1/s1
**377**	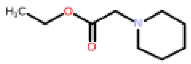	n.a.	23853-10-3	Z2856434773	bmse011055	InChI=1S/C9H17NO2/c1-2-12-9(11)8-10-6-4-3-5-7-10/h2-8H2,1H3
**378**	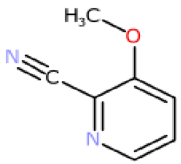	n.a.	24059-89-0	Z1955122823	bmse011154	InChI=1S/C7H6N2O/c1-10-7-3-2-4-9-6(7)5-8/h2-4H,1H3
**379**	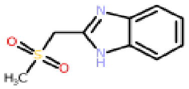	n.a.	24092-75-9	Z126932614	bmse011012	InChI=1S/C9H10N2O2S/c1-14(12,13)6-9-10-7-4-2-3-5-8(7)11-9/h2-5H,6H2,1H3,(H,10,11)
**380**	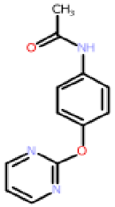	n.a.	24550-10-5	Z54571979	bmse011261	InChI=1S/C12H11N3O2/c1-9(16)15-10-3-5-11(6-4-10)17-12-13-7-2-8-14-12/h2-8H,1H3,(H,15,16)

Suppl Table 2: Details of the 768 compounds from the DSI-Poised fragment Library (DSI-PL)

Sl. No.	Structures	No. in Manuscript	CAS Registry Number	"ID"	^{1}H NMR Data Repository BMRB ID	ALATIS InChI
**381**	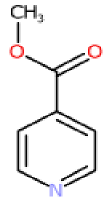	n.a.	2459-09-8	Z19684186	bmse011470	InChI=1S/C7H7NO2/c1-10-7(9)6-2-4-8-5-3-6/h2-5H,1H3
**382**	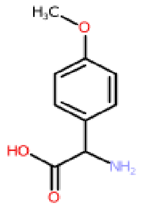	n.a.	24593-49-5	Z56755722	bmse011480	InChI=1S/C9H11NO3/c1-13-7-4-2-6(3-5-7)8(10)9(11)12/h2-5,8H,10H2,1H3,(H,11,12)/t8-/m1/s1
**383**	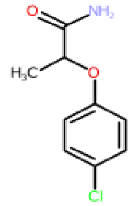	n.a.	24889-00-7	Z19727416	bmse011074	InChI=1S/C9H10ClNO2/c1-6(9(11)12)13-8-4-2-7(10)3-5-8/h2-6H,1H3,(H2,11,12)/t6-/m0/s1
**384**	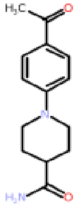	n.a.	250713-72-5	Z126933926	bmse011024	InChI=1S/C14H18N2O2/c1-10(17)11-2-4-13(5-3-11)16-8-6-12(7-9-16)14(15)18/h2-5,12H,6-9H2,1H3,(H2,15,18)
**385**	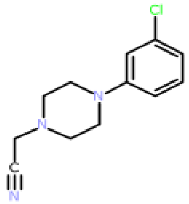	n.a.	25178-91-0	Z2856434806	bmse011163	InChI=1S/C12H14ClN3/c13-11-2-1-3-12(10-11)16-8-6-15(5-4-14)7-9-16/h1-3,10H,5-9H2

Suppl Table 2: Details of the 768 compounds from the DSI-Poised fragment Library (DSI-PL)

Sl. No.	Structures	No. in Manuscript	CAS Registry Number	"ID"	^{1}H NMR Data Repository BMRB ID	ALATIS InChI
**386**	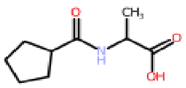	n.a.	252678-52-7	Z1238477790	bmse011253	InChI=1S/C9H15NO3/c1-6(9(12)13)10-8(11)7-4-2-3-5-7/h6-7H,2-5H2,1H3,(H,10,11)(H,12,13)/t6-/m1/s1
**387**	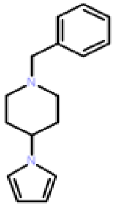	n.a.	254115-91-8	Z2856434898	bmse011494	InChI=1S/C16H20N2/c1-2-6-15(7-3-1)14-17-12-8-16(9-13-17)18-10-4-5-11-18/h1-7,10-11,16H,8-9,12-14H2
**388**	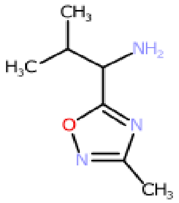	n.a.	254744-73-5	Z1267773566	bmse011533	InChI=1S/C7H13N3O/c1-4(2)6(8)7-9-5(3)10-11-7/h4,6H,8H2,1-3H3/t6-/m0/s1
**389**	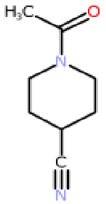	n.a.	25503-91-7	Z952656810	bmse011661	InChI=1S/C8H12N2O/c1-7(11)10-4-2-8(6-9)3-5-10/h8H,2-5H2,1H3
**390**	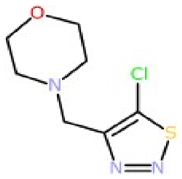	n.a.	256508-44-8	Z2856434925	bmse011247	nChI=1S/C7H10ClN3OS/c8-7-6(9-10-13-7)5-11-1-3-12-4-2-11/h1-5H

Suppl Table 2: Details of the 768 compounds from the DSI-Poised fragment Library (DSI-PL)

Sl. No.	Structures	No. in Manuscript	CAS Registry Number	"ID"	^{1}H NMR Data Repository BMRB ID	ALATIS InChI
391	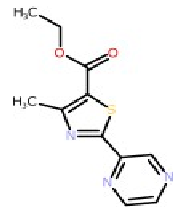	n.a.	256529-18-7	Z735557654	bmse011708	InChI=1S/C11H11N3O2S/c1-3-16-11(15)9-7(2)14-10(17-9)8-6-12-4-5-13-8/h4-6H,3H2,1-2H3
392	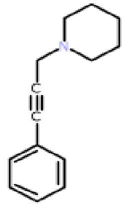	n.a.	2568-57-2	Z57257264	bmse011044	InChI=1S/C14H17N/c1-3-8-14(9-4-1)10-7-13-15-11-5-2-6-12-15/h1,3-4,8-9H,2,5-6,11-13H2
393	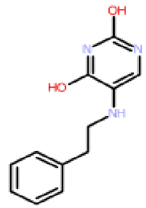	n.a.	25912-34-9	Z57744604	bmse011352	InChI=1S/C12H13N3O2/c16-11-10(8-14-12(17)15-11)13-7-6-9-4-2-1-3-5-9/h1-5,8,13H,6-7H2,(H2,14,15,16,17)
394	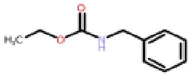	n.a.	2621-78-5	Z31478129	bmse011632	InChI=1S/C10H13NO2/c1-2-13-10(12)11-8-9-6-4-3-5-7-9/h3-7H,2,8H2,1H3,(H,11,12)
395	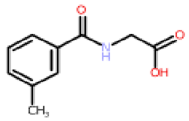	n.a.	27115-49-7	Z56827661	bmse011384	InChI=1S/C10H11NO3/c1-7-3-2-4-8(5-7)10(14)11-6-9(12)13/h2-5H,6H2,1H3,(H,11,14)(H,12,13)

Suppl Table 2: Details of the 768 compounds from the DSI-Poised fragment Library (DSI-PL)

Sl. No.	Structures	No. in Manuscript	CAS Registry Number	"ID"	1H NMR Data Repository BMRB ID	ALATIS InChI
**396**	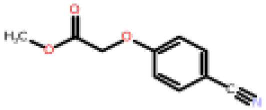	n.a.	272792-14-0	Z19234337	bmse011144	InChI=1S/C10H9NO3/c1-13-10(12)7-14-9-4-2-8(6-11)3-5-9/h2-5H,7H2,1H3
**397**	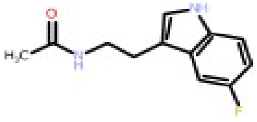	n.a.	2806-01-1	Z1220452176	bmse011339	InChI=1S/C12H13FN2O/c1-8(16)14-5-4-9-7-15-12-3-2-10(13)6-11(9)12/h2-3,6-7,15H,4-5H2,1H3,(H,14,16)
**398**	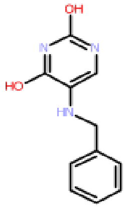	n.a.	28485-19-0	Z111666026	bmse011228	InChI=1S/C11H11N3O2/c15-10-9(7-13-11(16)14-10)12-6-8-4-2-1-3-5-8/h1-5,7,12H,6H2,(H2,13,14,15,16)
**399**	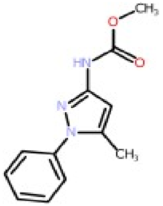	n.a.	287176-87-8	Z1581680287	bmse011706	InChI=1S/C12H13N3O2/c1-9-8-11(13-12(16)17-2)14-15(9)10-6-4-3-5-7-10/h3-8H,1-2H3,(H,13,14,16)
**400**	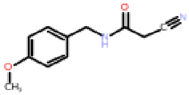	n.a.	288154-72-3	Z190662888	bmse011016	InChI=1S/C11H12N2O2/c1-15-10-4-2-9(3-5-10)8-13-11(14)6-7-12/h2-5H,6,8H2,1H3,(H,13,14)

Suppl Table 2: Details of the 768 compounds from the DSI-Poised fragment Library (DSI-PL)

Sl. No.	Structures	No. in Manuscript	CAS Registry Number	"ID"	^{1}H NMR Data Repository BMRB ID	ALATIS InChI
**401**	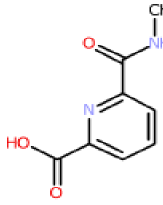	n.a.	288616-99-9	Z1816233707	bmse011110	InChI=1S/C8H8N2O3/c1-9-7(11)5-3-2-4-6(10-5)8(12)13/h2-4H,1H3,(H,9,11)(H,12,13)
**402**	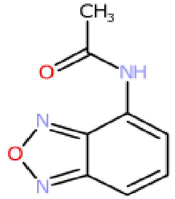	n.a.	289650-01-7	Z2027158783	bmse011694	InChI=1S/C8H7N3O2/c1-5(12)9-6-3-2-4-7-8(6)11-13-10-7/h2-4H,1H3,(H,9,12)
**403**	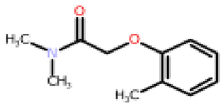	n.a.	29239-56-3	Z19733482	bmse011237	InChI=1S/C11H15NO2/c1-9-6-4-5-7-10(9)14-8-11(13)12(2)3/h4-7H,8H2,1-3H3
**404**	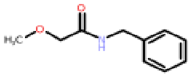	n.a.	2945-05-3	Z31478538	bmse011582	InChI=1S/C10H13NO2/c1-13-8-10(12)11-7-9-5-3-2-4-6-9/h2-6H,7-8H2,1H3,(H,11,12)
**405**	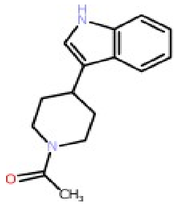	n.a.	30030-83-2	Z223388508	bmse011631	InChI=1S/C15H18N2O/c1-11(18)17-8-6-12(7-9-17)14-10-16-15-5-3-2-4-13(14)15/h2-5,10,12,16H,6-9H2,1H3

Suppl Table 2: Details of the 768 compounds from the DSI-Poised fragment Library (DSI-PL)

Sl. No.	Structures	No. in Manuscript	CAS Registry Number	"ID"	^{1}H NMR Data Repository BMRB ID	ALATIS InChI
**406**	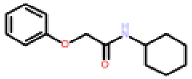	n.a.	303092-41-3	Z19735904	bmse011064	InChI=1S/C14H19NO2/c16-14(15-12-7-3-1-4-8-12)11-17-13-9-5-2-6-10-13/h2,5-6,9-10,12H,1,3-4,7-8,11H2,(H,15,16)
**407**	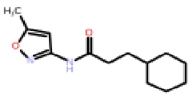	n.a.	303132-67-4	Z86949053	bmse011717	InChI=1S/C13H20N2O2/c1-10-9-12(15-17-10)14-13(16)8-7-11-5-3-2-4-6-11/h9,11H,2-8H2,1H3,(H,14,15,16)
**408**	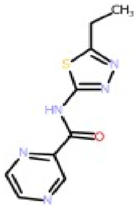	n.a.	303138-15-0	Z26824727	bmse011014	InChI=1S/C9H9N5OS/c1-2-7-13-14-9(16-7)12-8(15)6-5-10-3-4-11-6/h3-5H,2H2,1H3,(H,12,14,15)
**409**	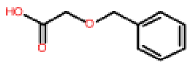	n.a.	30379-55-6	Z2856434906	bmse011765	InChI=1S/C9H10O3/c10-9(11)7-12-6-8-4-2-1-3-5-8/h1-5H,6-7H2,(H,10,11)
**410**	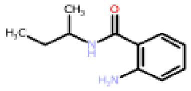	n.a.	30391-87-8	Z56040660	bmse011035	InChI=1S/C11H16N2O/c1-3-8(2)13-11(14)9-6-4-5-7-10(9)12/h4-8H,3,12H2,1-2H3,(H,13,14)/t8-/m1/s1

Suppl Table 2: Details of the 768 compounds from the DSI-Poised fragment Library (DSI-PL)

Sl. No.	Structures	No. in Manuscript	CAS Registry Number	"ID"	^{1}H NMR Data Repository BMRB ID	ALATIS InChI
**411**	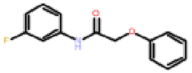	n.a.	303989-18-6	Z19735192	bmse011677	InChI=1S/C14H12FNO2/c15-11-5-4-6-12(9-11)16-14(17)10-18-13-7-2-1-3-8-13/h1-9H,10H2,(H,16,17)
**412**	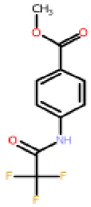	n.a.	304646-56-8	Z56767614	bmse011373	InChI=1S/C10H8F3NO3/c1-17-8(15)6-2-4-7(5-3-6)14-9(16)10(11,12)13/h2-5H,1H3,(H,14,16)
**413**	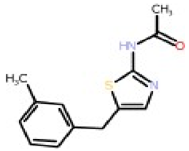	n.a.	304895-11-2	Z57080305	bmse011039	InChI=1S/C13H14N2OS/c1-9-4-3-5-11(6-9)7-12-8-14-13(17-12)15-10(2)16/h3-6,8H,7H2,1-2H3,(H,14,15,16)
**414**	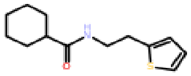	n.a.	308287-68-5	Z29191465	bmse011580	InChI=1S/C13H19NOS/c15-13(11-5-2-1-3-6-11)14-9-8-12-7-4-10-16-12/h4,7,10-11H,1-3,5-6,8-9H2,(H,14,15)
**415**	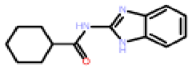	n.a.	312525-49-8	Z27666218	n.a.	n.a.

Suppl Table 2: Details of the 768 compounds from the DSI-Poised fragment Library (DSI-PL)

Sl. No.	Structures	No. in Manuscript	CAS Registry Number	"ID"	1H NMR Data Repository BMRB ID	ALATIS InChI
**416**	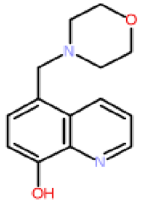	n.a.	312611-90-8	Z2856434798	bmse011422	InChI=1S/C14H16N2O2/c17-13-4-3-11(10-16-6-8-18-9-7-16)12-2-1-5-15-14(12)13/h1-5,17H,6-10H2
**417**	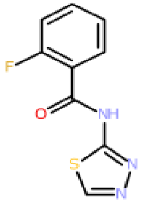	n.a.	313368-15-9	Z86417414	bmse011241	InChI=1S/C9H6FN3OS/c10-7-4-2-1-3-6(7)8(14)12-9-13-11-5-15-9/h1-5H,(H,12,13,14)
**418**	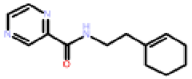	n.a.	313385-77-2	Z69091635	bmse011319	InChI=1S/C13H17N3O/c17-13(12-10-14-8-9-15-12)16-7-6-11-4-2-1-3-5-11/h4,8-10H,1-3,5-7H2,(H,16,17)
**419**	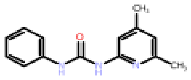	n.a.	313386-33-3	Z1693429442	bmse011175	InChI=1S/C14H15N3O/c1-10-8-11(2)15-13(9-10)17-14(18)16-12-6-4-3-5-7-12/h3-9H,1-2H3,(H2,15,16,17,18)
**420**	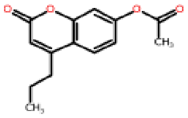	n.a.	313470-98-3	Z29634868	bmse011375	InChI=1S/C14H14O4/c1-3-4-10-7-14(16)18-13-8-11(17-9(2)15)5-6-12(10)13/h5-8H,3-4H2,1-2H3

Suppl Table 2: Details of the 768 compounds from the DSI-Poised fragment Library (DSI-PL)

Sl. No.	Structures	No. in Manuscript	CAS Registry Number	"ID"	^{1}H NMR Data Repository BMRB ID	ALATIS InChI
**421**	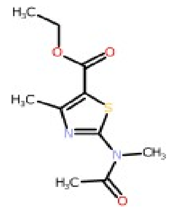	n.a.	3161-69-1	Z1230032143	bmse011291	InChI=1S/C10H14N2O3S/c1-5-15-9(14)8-6(2)11-10(16-8)12(4)7(3)13/h5H2,1-4H3
**422**	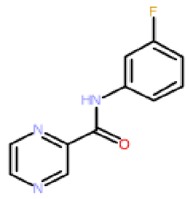	n.a.	320582-40-9	Z28143241	bmse011071	InChI=1S/C11H8FN3O/c12-8-2-1-3-9(6-8)15-11(16)10-7-13-4-5-14-10/h1-7H,(H,15,16)
**423**	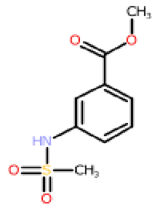	n.a.	32087-05-1	Z24758179	bmse011710	InChI=1S/C9H11NO4S/c1-14-9(11)7-4-3-5-8(6-7)10-15(2,12)13/h3-6,10H,1-2H3
**424**	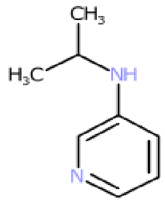	n.a.	32405-76-8	Z1259341037	bmse011267	InChI=1S/C8H12N2/c1-7(2)10-8-4-3-5-9-6-8/h3-7,10H,1-2H3
**425**	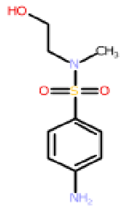	n.a.	328072-15-7	Z1673618163	bmse011296	InChI=1S/C9H14N2O3S/c1-11(6-7-12)15(13,14)9-4-2-8(10)3-5-9/h2-5,12H,6-7,10H2,1H3

Suppl Table 2: Details of the 768 compounds from the DSI-Poised fragment Library (DSI-PL)

Sl. No.	Structures	No. in Manuscript	CAS Registry Number	"ID"	^{1}H NMR Data Repository BMRB ID	ALATIS InChI
**426**	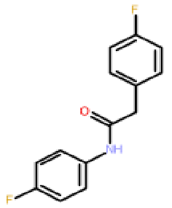	n.a.	328278-71-3	Z27805986	bmse011673	InChI=1S/C14H11F2NO/c15-11-3-1-10(2-4-11)9-14(18)17-13-7-5-12(16)6-8-13/h1-8H,9H2,(H,17,18)
**427**	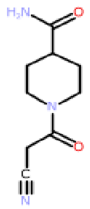	n.a.	328384-63-0	Z57627041	bmse011063	InChI=1S/C9H13N3O2/c10-4-1-8(13)12-5-2-7(3-6-12)9(11)14/h7H,1-3,5-6H2,(H2,11,14)
**428**	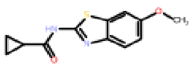	n.a.	32895-11-7	Z27678561	bmse011080	InChI=1S/C12H12N2O2S/c1-16-8-4-5-9-10(6-8)17-12(13-9)14-11(15)7-2-3-7/h4-7H,2-3H2,1H3,(H,13,14,15)
**429**	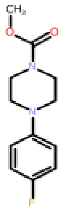	n.a.	329042-42-4	Z192955056	bmse011681	InChI=1S/C12H15FN2O2/c1-17-12(16)15-8-6-14(7-9-15)11-4-2-10(13)3-5-11/h2-5H,6-9H2,1H3
**430**	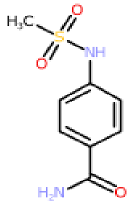	n.a.	329043-17-6	Z45656995	bmse011214	InChI=1S/C8H10N2O3S/c1-14(12,13)10-7-4-2-6(3-5-7)8(9)11/h2-5,10H,1H3,(H2,9,11)

Suppl Table 2: Details of the 768 compounds from the DSI-Poised fragment Library (DSI-PL)

Sl. No.	Structures	No. in Manuscript	CAS Registry Number	"ID"	^{1}H NMR Data Repository BMRB ID	ALATIS InChI
**431**	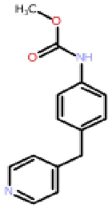	n.a.	329921-01-9	Z57190020	bmse011358	InChI=1S/C14H14N2O2/c1-18-14(17)16-13-4-2-11(3-5-13)10-12-6-8-15-9-7-12/h2-9H,10H2,1H3,(H,16,17)
**432**	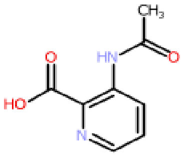	n.a.	3303-18-2	Z1815155460	bmse011109	InChI=1S/C8H8N2O3/c1-5(11)10-6-3-2-4-9-7(6)8(12)13/h2-4H,1H3,(H,10,11)(H,12,13)
**433**	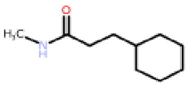	n.a.	330855-40-8	Z32016974	bmse011082	InChI=1S/C10H19NO/c1-11-10(12)8-7-9-5-3-2-4-6-9/h9H,2-8H2,1H3,(H,11,12)
**434**	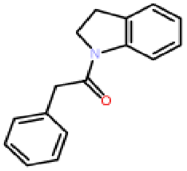	n.a.	331240-55-2	Z30624464	bmse011031	InChI=1S/C16H15NO/c18-16(12-13-6-2-1-3-7-13)17-11-10-14-8-4-5-9-15(14)17/h1-9H,10-12H2
**435**	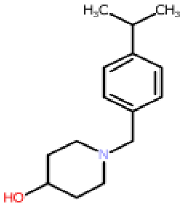	n.a.	331855-73-3	Z2856434874	bmse011313	InChI=1S/C15H23NO/c1-12(2)14-5-3-13(4-6-14)11-16-9-7-15(17)8-10-16/h3-6,12,15,17H,7-11H2,1-2H3

Suppl Table 2: Details of the 768 compounds from the DSI-Poised fragment Library (DSI-PL)

Sl. No.	Structures	No. in Manuscript	CAS Registry Number	"ID"	^{1}H NMR Data Repository BMRB ID	ALATIS InChI
**436**	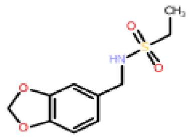	n.a.	332025-91-9	Z53860899	bmse011670	InChI=1S/C10H13NO4S/c1-2-16(12,13)11-6-8-3-4-9-10(5-8)15-7-14-9/h3-5,11H,2,6-7H2,1H3
**437**	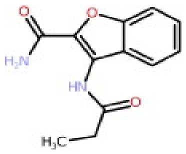	n.a.	332374-98-8	Z108545814	bmse011357	InChI=1S/C12H12N2O3/c1-2-9(15)14-10-7-5-3-4-6-8(7)17-11(10)12(13)16/h3-6H,2H2,1H3,(H2,13,16)(H,14,15)
**438**	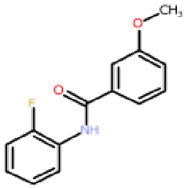	n.a.	333349-76-1	Z28290384	bmse011015	InChI=1S/C14H12FNO2/c1-18-11-6-4-5-10(9-11)14(17)16-13-8-3-2-7-12(13)15/h2-9H,1H3,(H,16,17)
**439**	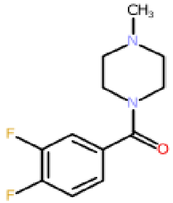	n.a.	333742-29-3	Z2856434829	bmse011662	InChI=1S/C12H14F2N2O/c1-15-4-6-16(7-5-15)12(17)9-2-3-10(13)11(14)8-9/h2-3,8H,4-7H2,1H3
**440**	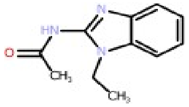	n.a.	333771-27-0	Z68639747	bmse011740	InChI=1S/C11H13N3O/c1-3-14-10-7-5-4-6-9(10)13-11(14)12-8(2)15/h4-7H,3H2,1-2H3,(H,12,13,15)

Suppl Table 2: Details of the 768 compounds from the DSI-Poised fragment Library (DSI-PL)

Sl. No.	Structures	No. in Manuscript	CAS Registry Number	"ID"	^{1}H NMR Data Repository BMRB ID	ALATIS InChI
**441**	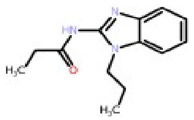	n.a.	333771-28-1	Z29077827	bmse011321	InChI=1S/C13H17N3O/c1-3-9-16-11-8-6-5-7-10(11)14-13(16)15-12(17)4-2/h5-8H,3-4,9H2,1-2H3,(H,14,15,17)
**442**	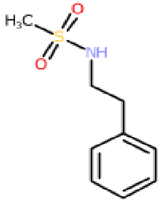	n.a.	33893-36-6	Z45617795	bmse011655	InChI=1S/C9H13NO2S/c1-13(11,12)10-8-7-9-5-3-2-4-6-9/h2-6,10H,7-8H2,1H3
**443**	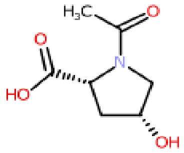	n.a.	33996-33-7	Z1824511473	bmse011468	InChI=1S/C7H11NO4/c1-4(9)8-3-5(10)2-6(8)7(11)12/h5-6,10H,2-3H2,1H3,(H,11,12)/t5-,6-/m1/s1
**444**	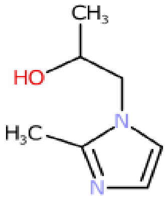	n.a.	34091-34-4	Z1362754425	bmse011540	InChI=1S/C7H12N2O/c1-6(10)5-9-4-3-8-7(9)2/h3-4,6,10H,5H2,1-2H3/t6-/m0/s1
**445**	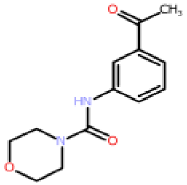	n.a.	341021-30-5	Z274555794	bmse011388	InChI=1S/C13H16N2O3/c1-10(16)11-3-2-4-12(9-11)14-13(17)15-5-7-18-8-6-15/h2-4,9H,5-8H2,1H3,(H,14,17)

Suppl Table 2: Details of the 768 compounds from the DSI-Poised fragment Library (DSI-PL)

Sl. No.	Structures	No. in Manuscript	CAS Registry Number	"ID"	1H NMR Data Repository BMRB ID	ALATIS InChI
**446**	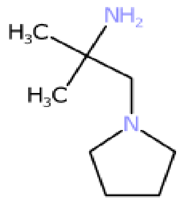	n.a.	34155-39-0	Z1741966630	bmse011609	InChI=1S/C8H18N2/c1-8(2,9)7-10-5-3-4-6-10/h3-7,9H2,1-2H3
**447**	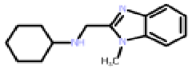	n.a.	342385-30-2	Z2856434855	bmse011381	InChI=1S/C15H21N3/c1-18-14-10-6-5-9-13(14)17-15(18)11-16-12-7-3-2-4-8-12/h5-6,9-10,12,16H,2-4,7-8,11H2,1H3
**448**	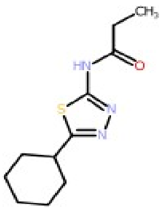	n.a.	342594-15-4	Z165063492	bmse011361	InChI=1S/C11H17N3OS/c1-2-9(15)12-11-14-13-10(16-11)8-6-4-3-5-7-8/h8H,2-7H2,1H3,(H,12,14,15)
**449**	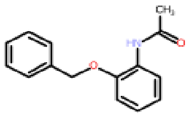	n.a.	34288-19-2	Z26365442	bmse011025	InChI=1S/C15H15NO2/c1-12(17)16-14-9-5-6-10-15(14)18-11-13-7-3-2-4-8-13/h2-10H,11H2,1H3,(H,16,17)
**450**	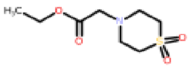	n.a.	343334-01-0	Z2856434920	bmse011252	InChI=1S/C8H15NO4S/c1-2-13-8(10)7-9-3-5-14(11,12)6-4-9/h2-7H2,1H3

Suppl Table 2: Details of the 768 compounds from the DSI-Poised fragment Library (DSI-PL)

Sl. No.	Structures	No. in Manuscript	CAS Registry Number	"ID"	^{1}H NMR Data Repository BMRB ID	ALATIS InChI
451	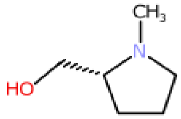	n.a.	#REF!	Z1002568728	bmse011475	InChI=1S/C6H13NO/c1-7-4-2-3-6(7)5-8/h6,8H,2-5H2,1H3/t6-/m1/s1
452	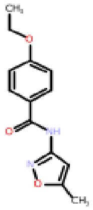	n.a.	346720-95-4	Z86948938	bmse011088	InChI=1S/C13H14N2O3/c1-3-17-11-6-4-10(5-7-11)13(16)14-12-8-9(2)18-15-12/h4-8H,3H2,1-2H3,(H,14,15,16)
453	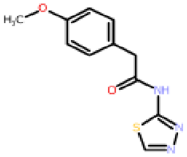	n.a.	346727-10-4	Z86416929	bmse011659	InChI=1S/C11H11N3O2S/c1-16-9-4-2-8(3-5-9)6-10(15)13-11-14-12-7-17-11/h2-5,7H,6H2,1H3,(H,13,14,15)
454	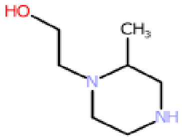	n.a.	3477-42-7	Z1741794237	bmse011550	InChI=1S/C7H16N2O/c1-7-6-8-2-3-9(7)4-5-10/h7-8,10H,2-6H2,1H3/t7-/m0/s1
455	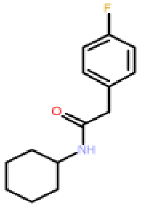	n.a.	348156-13-8	Z31484539	bmse011068	InChI=1S/C14H18FNO/c15-12-8-6-11(7-9-12)10-14(17)16-13-4-2-1-3-5-13/h6-9,13H,1-5,10H2,(H,16,17)

Suppl Table 2: Details of the 768 compounds from the DSI-Poised fragment Library (DSI-PL)

Sl. No.	Structures	No. in Manuscript	CAS Registry Number	"ID"	1H NMR Data Repository BMRB ID	ALATIS InChI
**456**	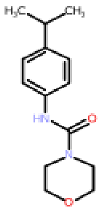	n.a.	349139-26-0	Z208334100	bmse011196	InChI=1S/C14H20N2O2/c1-11(2)12-3-5-13(6-4-12)15-14(17)16-7-9-18-10-8-16/h3-6,11H,7-10H2,1-2H3,(H,15,17)
**457**	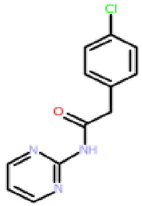	n.a.	349419-74-5	Z203581214	bmse011020	InChI=1S/C12H10ClN3O/c13-10-4-2-9(3-5-10)8-11(17)16-12-14-6-1-7-15-12/h1-7H,8H2,(H,14,15,16,17)
**458**	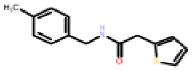	n.a.	349429-04-5	Z28429411	bmse011057	InChI=1S/C14H15NOS/c1-11-4-6-12(7-5-11)10-15-14(16)9-13-3-2-8-17-13/h2-8H,9-10H2,1H3,(H,15,16)
**459**	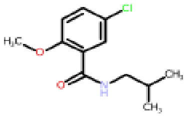	n.a.	349434-28-2	Z32386228	bmse011345	InChI=1S/C12H16ClNO2/c1-8(2)7-14-12(15)10-6-9(13)4-5-11(10)16-3/h4-6,8H,7H2,1-3H3,(H,14,15)
**460**	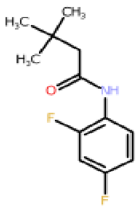	n.a.	349437-14-5	Z30802768	bmse011324	InChI=1S/C12H15F2NO/c1-12(2,3)7-11(16)15-10-5-4-8(13)6-9(10)14/h4-6H,7H2,1-3H3,(H,15,16)

Suppl Table 2: Details of the 768 compounds from the DSI-Poised fragment Library (DSI-PL)

Sl. No.	Structures	No. in Manuscript	CAS Registry Number	"ID"	^{1}H NMR Data Repository BMRB ID	ALATIS InChI
**461**	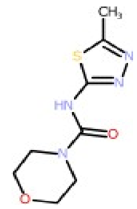	n.a.	349442-33-7	Z410633222	bmse011198	InChI=1S/C8H12N4O2S/c1-6-10-11-7(15-6)9-8(13)12-2-4-14-5-3-12/h2-5H2,1H3,(H,9,11,13)
**462**	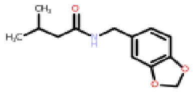	n.a.	349540-63-2	Z27695365	bmse011703	InChI=1S/C13H17NO3/c1-9(2)5-13(15)14-7-10-3-4-11-12(6-10)17-8-16-11/h3-4,6,9H,5,7-8H2,1-2H3,(H,14,15)
**463**	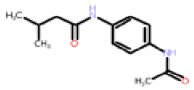	n.a.	349574-58-9	Z27653940	bmse011434	InChI=1S/C13H18N2O2/c1-9(2)8-13(17)15-12-6-4-11(5-7-12)14-10(3)16/h4-7,9H,8H2,1-3H3,(H,14,16)(H,15,17)
**464**	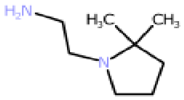	n.a.	35018-17-8	Z1741815708	bmse011574	InChI=1S/C8H18N2/c1-8(2)4-3-6-10(8)7-5-9/h3-7,9H2,1-2H3
**465**	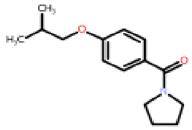	n.a.	350989-16-1	Z31480458	bmse011668	InChI=1S/C15H21NO2/c1-12(2)11-18-14-7-5-13(6-8-14)15(17)16-9-3-4-10-16/h5-8,12H,3-4,9-11H2,1-2H3

Suppl Table 2: Details of the 768 compounds from the DSI-Poised fragment Library (DSI-PL)

Sl. No.	Structures	No. in Manuscript	CAS Registry Number	"ID"	^{1}H NMR Data Repository BMRB ID	ALATIS InChI
**466**	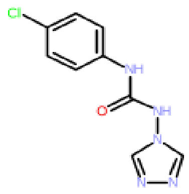	n.a.	351225-32-6	Z56259166	bmse011716	InChI=1S/C9H8ClN5O/c10-7-1-3-8(4-2-7)13-9(16)14-15-5-11-12-6-15/h1-6H,(H2,13,14,16)
**467**	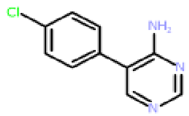	n.a.	35202-25-6	Z1262398388	bmse011273	InChI=1S/C10H8ClN3/c11-8-3-1-7(2-4-8)9-5-13-6-14-10(9)12/h1-6H,(H2,12,13,14)
**468**	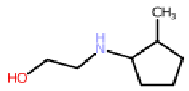	n.a.	35265-10-2	Z1396419547	bmse011559	InChI=1S/C8H17NO/c1-7-3-2-4-8(7)9-5-6-10/h7-10H,2-6H2,1H3/t7-,8-/m0/s1
**469**	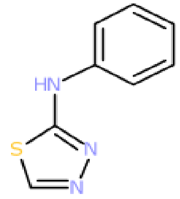	n.a.	3530-62-9	Z56347187	bmse011022	InChI=1S/C8H7N3S/c1-2-4-7(5-3-1)10-8-11-9-6-12-8/h1-6H,(H,10,11)
**470**	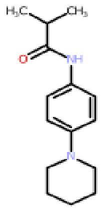	n.a.	354768-54-0	Z30595739	bmse011443	InChI=1S/C15H22N2O/c1-12(2)15(18)16-13-6-8-14(9-7-13)17-10-4-3-5-11-17/h6-9,12H,3-5,10-11H2,1-2H3,(H,16,18)

Suppl Table 2: Details of the 768 compounds from the DSI-Poised fragment Library (DSI-PL)

Sl. No.	Structures	No. in Manuscript	CAS Registry Number	"ID"	^{1}H NMR Data Repository BMRB ID	ALATIS InChI
**471**	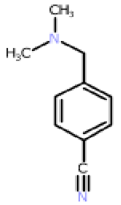	n.a.	35525-86-1	Z2856434815	bmse011567	InChI=1S/C10H12N2/c1-12(2)8-10-5-3-9(7-11)4-6-10/h3-6H,8H2,1-2H3
**472**	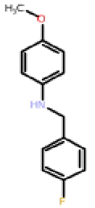	n.a.	356531-43-6	Z57328997	bmse011587	InChI=1S/C14H14FNO/c1-17-14-8-6-13(7-9-14)16-10-11-2-4-12(15)5-3-11/h2-9,16H,10H2,1H3
**473**	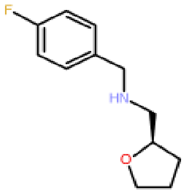	n.a.	356531-65-2	Z2856434824	bmse011743	InChI=1S/C12H16FNO/c13-11-5-3-10(4-6-11)8-14-9-12-2-1-7-15-12/h3-6,12,14H,1-2,7-9H2/t12-/m1/s1
**474**	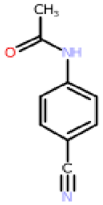	n.a.	35704-19-9	Z30612220	bmse011455	InChI=1S/C9H8N2O/c1-7(12)11-9-4-2-8(6-10)3-5-9/h2-5H,1H3,(H,11,12)
**475**	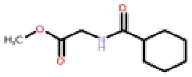	n.a.	35960-89-5	Z18618496	bmse011618	InChI=1S/C10H17NO3/c1-14-9(12)7-11-10(13)8-5-3-2-4-6-8/h8H,2-7H2,1H3,(H,11,13)

Suppl Table 2: Details of the 768 compounds from the DSI-Poised fragment Library (DSI-PL)

Sl. No.	Structures	No. in Manuscript	CAS Registry Number	"ID"	^{1}H NMR Data Repository BMRB ID	ALATIS InChI
**476**	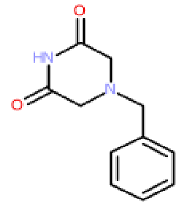	n.a.	35975-17-8	Z1157654117	bmse011767	InChI=1S/C11H12N2O2/c14-10-7-13(8-11(15)12-10)6-9-4-2-1-3-5-9/h1-5H,6-8H2,(H,12,14,15)
**477**	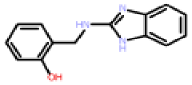	n.a.	364599-60-0	Z1587220559	bmse011372	InChI=1S/C14H13N3O/c18-13-8-4-1-5-10(13)9-15-14-16-11-6-2-3-7-12(11)17-14/h1-8,18H,9H2,(H2,15,16,17)
**478**	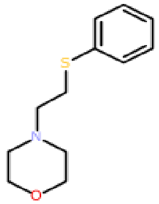	n.a.	364739-42-4	Z2856434865	bmse011647	InChI=1S/C12H17NOS/c1-2-4-12(5-3-1)15-11-8-13-6-9-14-10-7-13/h1-5H,6-11H2
**479**	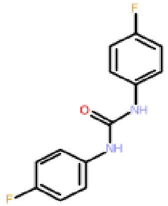	n.a.	370-22-9	Z44586758	bmse011036	InChI=1S/C13H10F2N2O/c14-9-1-5-11(6-2-9)16-13(18)17-12-7-3-10(15)4-8-12/h1-8H,(H2,16,17,18)
**480**	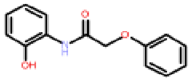	n.a.	3743-69-9	Z68195082	bmse011442	InChI=1S/C14H13NO3/c16-13-9-5-4-8-12(13)15-14(17)10-18-11-6-2-1-3-7-11/h1-9,16H,10H2,(H,15,17)

Suppl Table 2: Details of the 768 compounds from the DSI-Poised fragment Library (DSI-PL)

Sl. No.	Structures	No. in Manuscript	CAS Registry Number	"ID"	^{1}H NMR Data Repository BMRB ID	ALATIS InChI
**481**	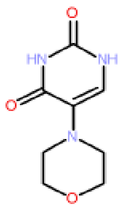	n.a.	37454-52-7	Z57282999	bmse011058	InChI=1S/C8H11N3O3/c12-7-6(5-9-8(13)10-7)11-1-3-14-4-2-11/h5H,1-4H2,(H2,9,10,12,13)
**482**	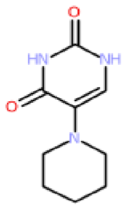	n.a.	37454-53-8	Z277468432	bmse011060	InChI=1S/C9H13N3O2/c13-8-7(6-10-9(14)11-8)12-4-2-1-3-5-12/h6H,1-5H2,(H2,10,11,13,14)
**483**	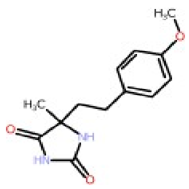	n.a.	374562-58-0	Z56772132	bmse011374	InChI=1S/C13H16N2O3/c1-13(11(16)14-12(17)15-13)8-7-9-3-5-10(18-2)6-4-9/h3-6H,7-8H2,1-2H3,(H2,14,15,16,17)/t13-/m1/s1
**484**	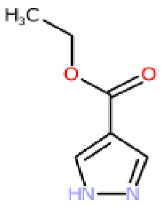	n.a.	37622-90-5	Z1124201124	bmse011396	InChI=1S/C6H8N2O2/c1-2-10-6(9)5-3-7-8-4-5/h3-4H,2H2,1H3,(H,7,8)
**485**	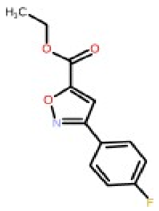	n.a.	377052-00-1	Z2106600670	n.a.	n.a.

Suppl Table 2: Details of the 768 compounds from the DSI-Poised fragment Library (DSI-PL)

Sl. No.	Structures	No. in Manuscript	CAS Registry Number	"ID"	^{1}H NMR Data Repository BMRB ID	ALATIS InChI
**486**	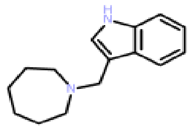	n.a.	378201-57-1	Z2856434848	bmse011368	InChI=1S/C15H20N2/c1-2-6-10-17(9-5-1)12-13-11-16-15-8-4-3-7-14(13)15/h3-4,7-8,11,16H,1-2,5-6,9-10,12H2
**487**	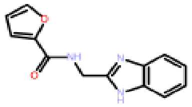	n.a.	378208-16-3	Z26781964	bmse011047	InChI=1S/C13H11N3O2/c17-13(11-6-3-7-18-11)14-8-12-15-9-4-1-2-5-10(9)16-12/h1-7H,8H2,(H,14,17)(H,15,16)
**488**	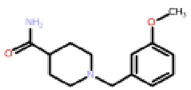	n.a.	380424-20-4	Z2856434854	bmse011299	InChI=1S/C14H20N2O2/c1-18-13-4-2-3-11(9-13)10-16-7-5-12(6-8-16)14(15)17/h2-4,9,12H,5-8,10H2,1H3,(H2,15,17)
**489**	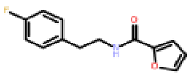	n.a.	380469-52-3	Z26769872	bmse011383	InChI=1S/C13H12FNO2/c14-11-5-3-10(4-6-11)7-8-15-13(16)12-2-1-9-17-12/h1-6,9H,7-8H2,(H,15,16)
**490**	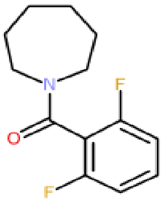	n.a.	385380-70-1	Z54226095	bmse011303	InChI=1S/C13H15F2NO/c14-10-6-5-7-11(15)12(10)13(17)16-8-3-1-2-4-9-16/h5-7H,1-4,8-9H2

Suppl Table 2: Details of the 768 compounds from the DSI-Poised fragment Library (DSI-PL)

Sl. No.	Structures	No. in Manuscript	CAS Registry Number	"ID"	^{1}H NMR Data Repository BMRB ID	ALATIS InChI
**491**	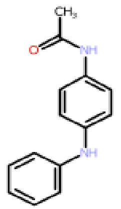	n.a.	38674-90-7	Z68404778	bmse011323	InChI=1S/C14H14N2O/c1-11(17)15-13-7-9-14(10-8-13)16-12-5-3-2-4-6-12/h2-10,16H,1H3,(H,15,17)
**492**	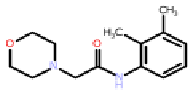	n.a.	389138-76-5	Z2856434834	bmse011336	InChI=1S/C14H20N2O2/c1-11-4-3-5-13(12(11)2)15-14(17)10-16-6-8-18-9-7-16/h3-5H,6-10H2,1-2H3,(H,15,17)
**493**	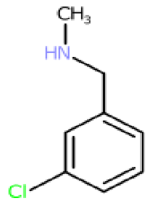	n.a.	39191-07-6	Z2856434770	bmse011579	InChI=1S/C8H10ClN/c1-10-6-7-3-2-4-8(9)5-7/h2-5,10H,6H2,1H3
**494**	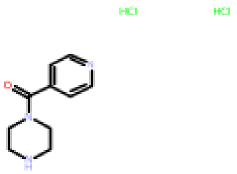	n.a.	39640-05-6	Z126932654	bmse011013	InChI=1S/C10H13N3O.2ClH/c14-10(9-1-3-11-4-2-9)13-7-5-12-6-8-13;;/h1-4,12H,5-8H2;2*1H
**495**	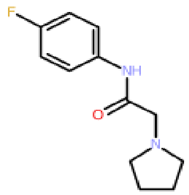	n.a.	397880-93-2	Z2856434942	bmse011421	InChI=1S/C12H15FN2O/c13-10-3-5-11(6-4-10)14-12(16)9-15-7-1-2-8-15/h3-6H,1-2,7-9H2,(H,14,16)

Suppl Table 2: Details of the 768 compounds from the DSI-Poised fragment Library (DSI-PL)

Sl. No.	Structures	No. in Manuscript	CAS Registry Number	"ID"	^{1}H NMR Data Repository BMRB ID	ALATIS InChI
**496**	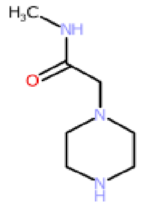	n.a.	39890-41-0	Z85934875	bmse011023	InChI=1S/C7H15N3O/c1-8-7(11)6-10-4-2-9-3-5-10/h9H,2-6H2,1H3,(H,8,11)
**497**	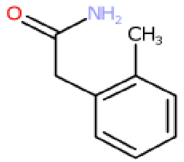	n.a.	40089-14-3	Z52214433	bmse011277	InChI=1S/C9H11NO/c1-7-4-2-3-5-8(7)6-9(10)11/h2-5H,6H2,1H3,(H2,10,11)
**498**	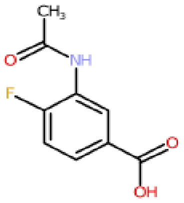	n.a.	401-37-6	Z364328788	bmse011745	InChI=1S/C9H8FNO3/c1-5(12)11-8-4-6(9(13)14)2-3-7(8)10/h2-4H,1H3,(H,11,12)(H,13,14)
**499**	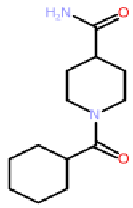	n.a.	401589-79-5	Z32399948	bmse011070	InChI=1S/C13H22N2O2/c14-12(16)10-6-8-15(9-7-10)13(17)11-4-2-1-3-5-11/h10-11H,1-9H2,(H2,14,16)
**500**	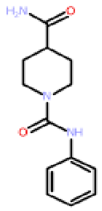	n.a.	401589-80-8	Z33545544	bmse011446	InChI=1S/C13H17N3O2/c14-12(17)10-6-8-16(9-7-10)13(18)15-11-4-2-1-3-5-11/h1-5,10H,6-9H2,(H2,14,17)(H,15,18)

Suppl Table 2: Details of the 768 compounds from the DSI-Poised fragment Library (DSI-PL)

Sl. No.	Structures	No. in Manuscript	CAS Registry Number	"ID"	1H NMR Data Repository BMRB ID	ALATIS InChI
**501**	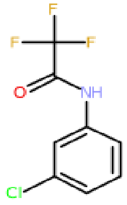	n.a.	40410-54-6	Z111810692	bmse011302	InChI=1S/C8H5ClF3NO/c9-5-2-1-3-6(4-5)13-7(14)8(10,11)12/h1-4H,(H,13,14)
**502**	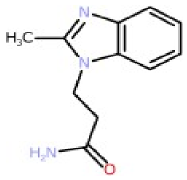	n.a.	40508-01-8	Z53116498	bmse011428	InChI=1S/C11H13N3O/c1-8-13-9-4-2-3-5-10(9)14(8)7-6-11(12)15/h2-5H,6-7H2,1H3,(H2,12,15)
**503**	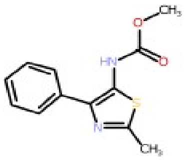	n.a.	41260-96-2	Z979145504	bmse011276	InChI=1S/C12H12N2O2S/c1-8-13-10(9-6-4-3-5-7-9)11(17-8)14-12(15)16-2/h3-7H,1-2H3,(H,14,15)
**504**	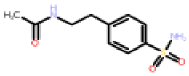	n.a.	41472-49-5	Z30857828	bmse011147	InChI=1S/C10H14N2O3S/c1-8(13)12-7-6-9-2-4-10(5-3-9)16(11,14)15/h2-5H,6-7H2,1H3,(H,12,13)(H2,11,14,15)
**505**	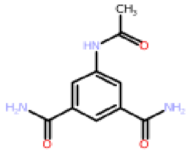	n.a.	414907-43-0	Z33486130	bmse011176	InChI=1S/C10H11N3O3/c1-5(14)13-8-3-6(9(11)15)2-7(4-8)10(12)16/h2-4H,1H3,(H2,11,15)(H2,12,16)(H,13,14)

Suppl Table 2: Details of the 768 compounds from the DSI-Poised fragment Library (DSI-PL)

Sl. No.	Structures	No. in Manuscript	CAS Registry Number	"ID"	^{1}H NMR Data Repository BMRB ID	ALATIS InChI
**506**	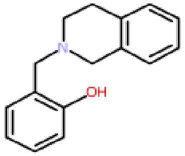	n.a.	415928-50-6	Z2856434881	bmse011709	InChI=1S/C16H17NO/c18-16-8-4-3-7-15(16)12-17-10-9-13-5-1-2-6-14(13)11-17/h1-8,18H,9-12H2
**507**	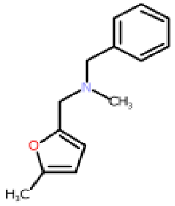	n.a.	415930-18-6	Z2856434896	bmse011486	InChI=1S/C14H17NO/c1-12-8-9-14(16-12)11-15(2)10-13-6-4-3-5-7-13/h3-9H,10-11H2,1-2H3
**508**	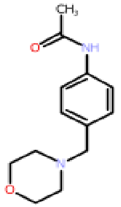	n.a.	415933-46-9	Z2856434875	bmse011192	InChI=1S/C13H18N2O2/c1-11(16)14-13-4-2-12(3-5-13)10-15-6-8-17-9-7-15/h2-5H,6-10H2,1H3,(H,14,16)
**509**	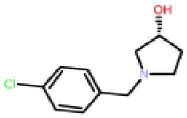	n.a.	415946-61-1	Z2856434858	bmse011628	InChI=1S/C11H14ClNO/c12-10-3-1-9(2-4-10)7-13-6-5-11(14)8-13/h1-4,11,14H,5-8H2/t11-/m1/s1
**510**	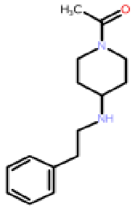	n.a.	415948-37-7	Z2856434883	bmse011657	InChI=1S/C15H22N2O/c1-13(18)17-11-8-15(9-12-17)16-10-7-14-5-3-2-4-6-14/h2-6,15-16H,7-12H2,1H3

Suppl Table 2: Details of the 768 compounds from the DSI-Poised fragment Library (DSI-PL)

Sl. No.	Structures	No. in Manuscript	CAS Registry Number	"ID"	^{1}H NMR Data Repository BMRB ID	ALATIS InChI
**511**	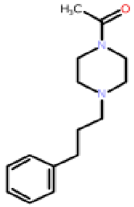	n.a.	415955-32-7	Z2856434884	bmse011663	InChI=1S/C15H22N2O/c1-14(18)17-12-10-16(11-13-17)9-5-8-15-6-3-2-4-7-15/h2-4,6-7H,5,8-13H2,1H3
**512**	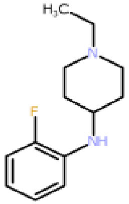	n.a.	416868-42-3	Z2856434868	bmse011672	InChI=1S/C13H19FN2/c1-2-16-9-7-11(8-10-16)15-13-6-4-3-5-12(13)14/h3-6,11,15H,2,7-10H2,1H3
**513**	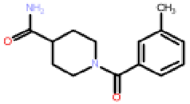	n.a.	418777-87-4	Z32400357	bmse011311	InChI=1S/C14H18N2O2/c1-10-3-2-4-12(9-10)14(18)16-7-5-11(6-8-16)13(15)17/h2-4,9,11H,5-8H2,1H3,(H2,15,17)
**514**	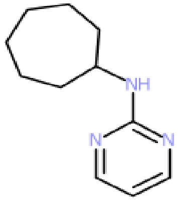	n.a.	419557-01-0	Z31244728	bmse011211	InChI=1S/C11H17N3/c1-2-4-7-10(6-3-1)14-11-12-8-5-9-13-11/h5,8-10H,1-4,6-7H2,(H,12,13,14)
**515**	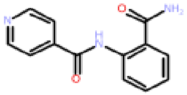	n.a.	419557-85-0	Z91797745	bmse011043	InChI=1S/C13H11N3O2/c14-12(17)10-3-1-2-4-11(10)16-13(18)9-5-7-15-8-6-9/h1-8H,(H2,14,17)(H,16,18)

Suppl Table 2: Details of the 768 compounds from the DSI-Poised fragment Library (DSI-PL)

Sl. No.	Structures	No. in Manuscript	CAS Registry Number	"ID"	^{1}H NMR Data Repository BMRB ID	ALATIS InChI
**516**	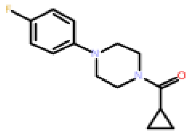	n.a.	419560-24-0	Z30620520	bmse011656	InChI=1S/C14H17FN2O/c15-12-3-5-13(6-4-12)16-7-9-17(10-8-16)14(18)11-1-2-11/h3-6,11H,1-2,7-10H2
**517**	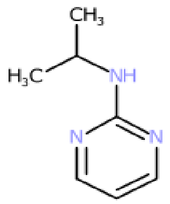	n.a.	4214-72-6	Z31190928	bmse011591	InChI=1S/C7H11N3/c1-6(2)10-7-8-4-3-5-9-7/h3-6H,1-2H3,(H,8,9,10)
**518**	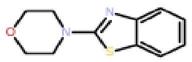	n.a.	4225-26-7	Z56767623	bmse011051	InChI=1S/C11H12N2OS/c1-2-4-10-9(3-1)12-11(15-10)13-5-7-14-8-6-13/h1-4H,5-8H2
**519**	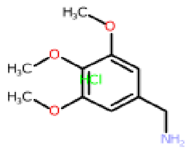	n.a.	42365-69-5	Z1741959530	bmse011445	InChI=1S/C10H15NO3.ClH/c1-12-8-4-7(6-11)5-9(13-2)10(8)14-3;/h4-5H,6,11H2,1-3H3;1H
**520**	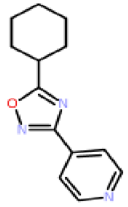	n.a.	423725-92-2	Z57715447	bmse011329	InChI=1S/C13H15N3O/c1-2-4-11(5-3-1)13-15-12(16-17-13)10-6-8-14-9-7-10/h6-9,11H,1-5H2

Suppl Table 2: Details of the 768 compounds from the DSI-Poised fragment Library (DSI-PL)

Sl. No.	Structures	No. in Manuscript	CAS Registry Number	"ID"	1H NMR Data Repository BMRB ID	ALATIS InChI
**521**	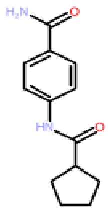	n.a.	424808-39-9	Z30242133	bmse011003	InChI=1S/C13H16N2O2/c14-12(16)9-5-7-11(8-6-9)15-13(17)10-3-1-2-4-10/h5-8,10H,1-4H2,(H2,14,16)(H,15,17)
**522**	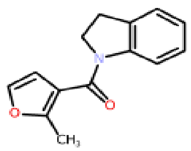	n.a.	424818-17-7	Z89307993	bmse011315	InChI=1S/C14H13NO2/c1-10-12(7-9-17-10)14(16)15-8-6-11-4-2-3-5-13(11)15/h2-5,7,9H,6,8H2,1H3
**523**	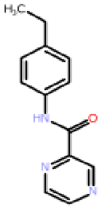	n.a.	424818-79-1	Z30917949	bmse011004	InChI=1S/C13H13N3O/c1-2-10-3-5-11(6-4-10)16-13(17)12-9-14-7-8-15-12/h3-9H,2H2,1H3,(H,16,17)
**524**	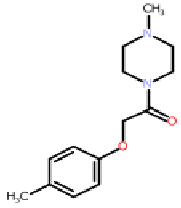	n.a.	426235-15-6	Z2856434836	bmse011658	InChI=1S/C14H20N2O2/c1-12-3-5-13(6-4-12)18-11-14(17)16-9-7-15(2)8-10-16/h3-6H,7-11H2,1-2H3
**525**	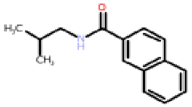	n.a.	429627-93-0	Z32385991	bmse011327	InChI=1S/C15H17NO/c1-11(2)10-16-15(17)14-8-7-12-5-3-4-6-13(12)9-14/h3-9,11H,10H2,1-2H3,(H,16,17)

Suppl Table 2: Details of the 768 compounds from the DSI-Poised fragment Library (DSI-PL)

Sl. No.	Structures	No. in Manuscript	CAS Registry Number	"ID"	^{1}H NMR Data Repository BMRB ID	ALATIS InChI
**526**	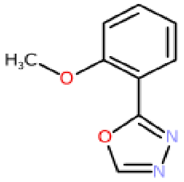	n.a.	42966-95-0	Z1359419878	bmse011395	InChI=1S/C9H8N2O2/c1-12-8-5-3-2-4-7(8)9-11-10-6-13-9/h2-6H,1H3
**527**	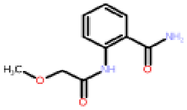	n.a.	430465-88-6	Z57260539	bmse011348	InChI=1S/C10H12N2O3/c1-15-6-9(13)12-8-5-3-2-4-7(8)10(11)14/h2-5H,6H2,1H3,(H2,11,14)(H,12,13)
**528**	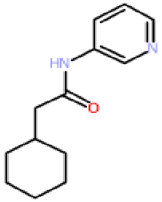	n.a.	431907-04-9	Z31792168	bmse011312	InChI=1S/C13H18N2O/c16-13(9-11-5-2-1-3-6-11)15-12-7-4-8-14-10-12/h4,7-8,10-11H,1-3,5-6,9H2,(H,15,16)
**529**	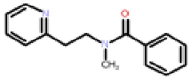	n.a.	431940-21-5	Z68277692	bmse011667	InChI=1S/C15H16N2O/c1-17(12-10-14-9-5-6-11-16-14)15(18)13-7-3-2-4-8-13/h2-9,11H,10,12H2,1H3
**530**	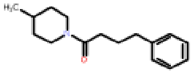	n.a.	432494-63-8	Z419884046	bmse011664	InChI=1S/C16H23NO/c1-14-10-12-17(13-11-14)16(18)9-5-8-15-6-3-2-4-7-15/h2-4,6-7,14H,5,8-13H2,1H3

Suppl Table 2: Details of the 768 compounds from the DSI-Poised fragment Library (DSI-PL)

Sl. No.	Structures	No. in Manuscript	CAS Registry Number	"ID"	1H NMR Data Repository BMRB ID	ALATIS InChI
**531**	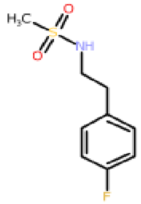	n.a.	432529-74-3	Z45705015	bmse011165	InChI=1S/C9H12FNO2S/c1-14(12,13)11-7-6-8-2-4-9(10)5-3-8/h2-5,11H,6-7H2,1H3
**532**	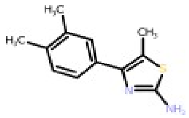	n.a.	438215-91-9	Z48847633	bmse011440	InChI=1S/C12H14N2S/c1-7-4-5-10(6-8(7)2)11-9(3)15-12(13)14-11/h4-6H,1-3H3,(H2,13,14)
**533**	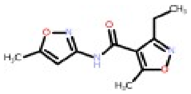	n.a.	438224-38-5	Z117233350	bmse011712	InChI=1S/C11H13N3O3/c1-4-8-10(7(3)17-13-8)11(15)12-9-5-6(2)16-14-9/h5H,4H2,1-3H3,(H,12,14,15)
**534**	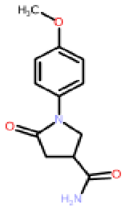	n.a.	443638-17-3	Z57324924	bmse011766	InChI=1S/C12H14N2O3/c1-17-10-4-2-9(3-5-10)14-7-8(12(13)16)6-11(14)15/h2-5,8H,6-7H2,1H3,(H2,13,16)/t8-/m1/s1
**535**	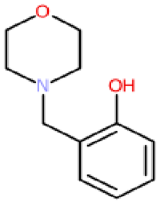	n.a.	4438-01-1	Z2856434887	bmse011362	InChI=1S/C11H15NO2/c13-11-4-2-1-3-10(11)9-12-5-7-14-8-6-12/h1-4,13H,5-9H2

Suppl Table 2: Details of the 768 compounds from the DSI-Poised fragment Library (DSI-PL)

Sl. No.	Structures	No. in Manuscript	CAS Registry Number	"ID"	1H NMR Data Repository BMRB ID	ALATIS InChI
**536**	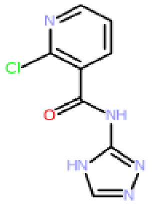	n.a.	444938-07-2	Z275154304	bmse011714	InChI=1S/C8H6ClN5O/c9-6-5(2-1-3-10-6)7(15)13-8-11-4-12-14-8/h1-4H,(H2,11,12,13,14,15)
**537**	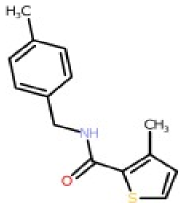	n.a.	445007-73-8	Z28429425	bmse011695	InChI=1S/C14H15NOS/c1-10-3-5-12(6-4-10)9-15-14(16)13-11(2)7-8-17-13/h3-8H,9H2,1-2H3,(H,15,16)
**538**	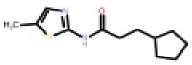	n.a.	445232-08-6	Z29325421	bmse011722	InChI=1S/C12H18N2OS/c1-9-8-13-12(16-9)14-11(15)7-6-10-4-2-3-5-10/h8,10H,2-7H2,1H3,(H,13,14,15)
**539**	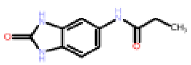	n.a.	445410-25-3	Z26794351	bmse011216	InChI=1S/C10H11N3O2/c1-2-9(14)11-6-3-4-7-8(5-6)13-10(15)12-7/h3-5H,2H2,1H3,(H,11,14)(H2,12,13,15)
**540**	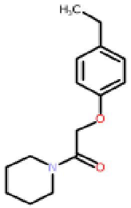	n.a.	448226-41-3	Z18839017	bmse011652	InChI=1S/C15H21NO2/c1-2-13-6-8-14(9-7-13)18-12-15(17)16-10-4-3-5-11-16/h6-9H,2-5,10-12H2,1H3

Suppl Table 2: Details of the 768 compounds from the DSI-Poised fragment Library (DSI-PL)

Sl. No.	Structures	No. in Manuscript	CAS Registry Number	"ID"	^{1}H NMR Data Repository BMRB ID	ALATIS InChI
**541**	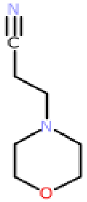	n.a.	4542-47-6	Z2856434767	bmse011738	InChI=1S/C7H12N2O/c8-2-1-3-9-4-6-10-7-5-9/h1,3-7H2
**542**	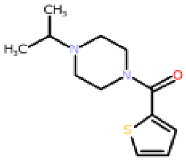	n.a.	461409-27-8	Z2856434826	bmse011702	InChI=1S/C12H18N2OS/c1-10(2)13-5-7-14(8-6-13)12(15)11-4-3-9-16-11/h3-4,9-10H,5-8H2,1-2H3
**543**	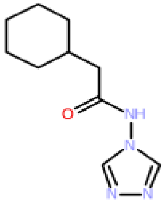	n.a.	462622-06-6	Z57261895	bmse011306	InChI=1S/C10H16N4O/c15-10(13-14-7-11-12-8-14)6-9-4-2-1-3-5-9/h7-9H,1-6H2,(H,13,15)
**544**	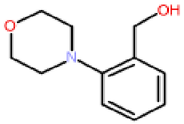	n.a.	465514-33-4	Z235361235	bmse011458	InChI=1S/C11H15NO2/c13-9-10-3-1-2-4-11(10)12-5-7-14-8-6-12/h1-4,13H,5-9H2
**545**	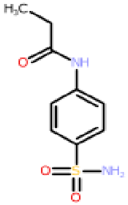	n.a.	4708-37-6	Z27682767	bmse011242	InChI=1S/C9H12N2O3S/c1-2-9(12)11-7-3-5-8(6-4-7)15(10,13)14/h3-6H,2H2,1H3,(H,11,12)(H2,10,13,14)

Suppl Table 2: Details of the 768 compounds from the DSI-Poised fragment Library (DSI-PL)

Sl. No.	Structures	No. in Manuscript	CAS Registry Number	"ID"	^{1}H NMR Data Repository BMRB ID	ALATIS InChI
**546**	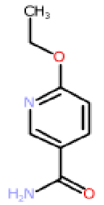	n.a.	473693-84-4	Z458894644	bmse011195	InChI=1S/C8H10N2O2/c1-2-12-7-4-3-6(5-10-7)8(9)11/h3-5H,2H2,1H3,(H2,9,11)
**547**	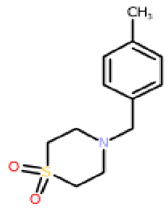	n.a.	477858-35-8	Z2856434929	bmse011318	InChI=1S/C12H17NO2S/c1-11-2-4-12(5-3-11)10-13-6-8-16(14,15)9-7-13/h2-5H,6-10H2,1H3
**548**	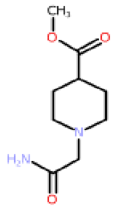	n.a.	477862-08-1	Z2856434804	bmse011293	InChI=1S/C9H16N2O3/c1-14-9(13)7-2-4-11(5-3-7)6-8(10)12/h7H,2-6H2,1H3,(H2,10,12)
**549**	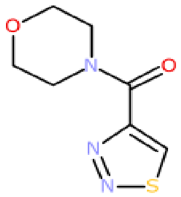	n.a.	478261-73-3	Z741055844	bmse011342	InChI=1S/C7H9N3O2S/c11-7(6-5-13-9-8-6)10-1-3-12-4-2-10/h5H,1-4H2
**550**	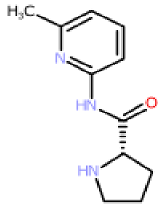	n.a.	478912-51-5	Z1267881672	bmse011284	InChI=1S/C11H15N3O/c1-8-4-2-6-10(13-8)14-11(15)9-5-3-7-12-9/h2,4,6,9,12H,3,5,7H2,1H3,(H,13,14,15)/t9-/m0/s1

Suppl Table 2: Details of the 768 compounds from the DSI-Poised fragment Library (DSI-PL)

Sl. No.	Structures	No. in Manuscript	CAS Registry Number	"ID"	^{1}H NMR Data Repository BMRB ID	ALATIS InChI
**551**	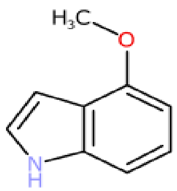	n.a.	4837-90-5	Z1429867185	bmse011151	InChI=1S/C9H9NO/c1-11-9-4-2-3-8-7(9)5-6-10-8/h2-6,10H,1H3
**552**	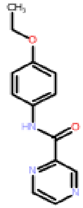	n.a.	484038-99-5	Z27808049	bmse011007	InChI=1S/C13H13N3O2/c1-2-18-11-5-3-10(4-6-11)16-13(17)12-9-14-7-8-15-12/h3-9H,2H2,1H3,(H,16,17)
**553**	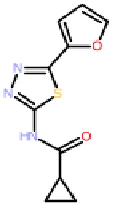	n.a.	484658-74-4	Z165141116	bmse011149	InChI=1S/C10H9N3O2S/c14-8(6-3-4-6)11-10-13-12-9(16-10)7-2-1-5-15-7/h1-2,5-6H,3-4H2,(H,11,13,14)
**554**	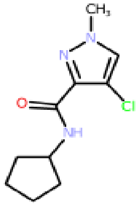	n.a.	489406-95-3	Z275151340	bmse011686	InChI=1S/C10H14ClN3O/c1-14-6-8(11)9(13-14)10(15)12-7-4-2-3-5-7/h6-7H,2-5H2,1H3,(H,12,15)
**555**	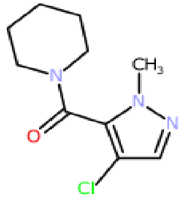	n.a.	491828-56-9	Z275179946	bmse011726	InChI=1S/C10H14ClN3O/c1-13-9(8(11)7-12-13)10(15)14-5-3-2-4-6-14/h7H,2-6H2,1H3

Suppl Table 2: Details of the 768 compounds from the DSI-Poised fragment Library (DSI-PL)

Sl. No.	Structures	No. in Manuscript	CAS Registry Number	"ID"	^{1}H NMR Data Repository BMRB ID	ALATIS InChI
**556**	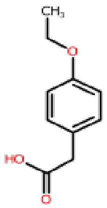	n.a.	4919-33-9	Z2856434918	bmse011437	InChI=1S/C10H12O3/c1-2-13-9-5-3-8(4-6-9)7-10(11)12/h3-6H,2,7H2,1H3,(H,11,12)
**557**	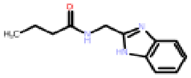	n.a.	497230-46-3	Z26781952	bmse011009	InChI=1S/C12H15N3O/c1-2-5-12(16)13-8-11-14-9-6-3-4-7-10(9)15-11/h3-4,6-7H,2,5,8H2,1H3,(H,13,16)(H,14,15)
**558**	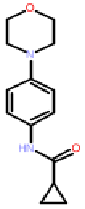	n.a.	499112-31-1	Z30485868	bmse011734	InChI=1S/C14H18N2O2/c17-14(11-1-2-11)15-12-3-5-13(6-4-12)16-7-9-18-10-8-16/h3-6,11H,1-2,7-10H2,(H,15,17)
**559**	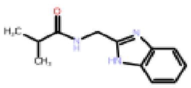	n.a.	500269-41-0	Z26781943	bmse011429	InChI=1S/C12H15N3O/c1-8(2)12(16)13-7-11-14-9-5-3-4-6-10(9)15-11/h3-6,8H,7H2,1-2H3,(H,13,16)(H,14,15)
**560**	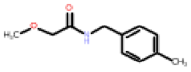	n.a.	503563-82-4	Z33297786	bmse011581	InChI=1S/C11H15NO2/c1-9-3-5-10(6-4-9)7-12-11(13)8-14-2/h3-6H,7-8H2,1-2H3,(H,12,13)

Suppl Table 2: Details of the 768 compounds from the DSI-Poised fragment Library (DSI-PL)

Sl. No.	Structures	No. in Manuscript	CAS Registry Number	"ID"	^{1}H NMR Data Repository BMRB ID	ALATIS InChI
**561**	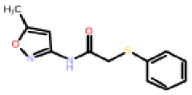	n.a.	505088-70-0	Z19750454	bmse011034	InChI=1S/C12H12N2O2S/c1-9-7-11(14-16-9)13-12(15)8-17-10-5-3-2-4-6-10/h2-7H,8H2,1H3,(H,13,14,15)
**562**	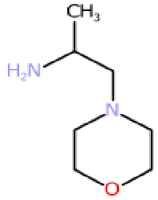	n.a.	50998-05-5	Z818732104	bmse011549	InChI=1S/C7H16N2O/c1-7(8)6-9-2-4-10-5-3-9/h7H,2-6,8H2,1H3/t7-/m0/s1
**563**	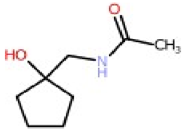	n.a.	51004-22-9	Z1203329531	bmse011129	InChI=1S/C8H15NO2/c1-7(10)9-6-8(11)4-2-3-5-8/h11H,2-6H2,1H3,(H,9,10)
**564**	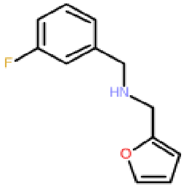	n.a.	510723-71-4	Z2737076969	bmse011689	InChI=1S/C12H12FNO/c13-11-4-1-3-10(7-11)8-14-9-12-5-2-6-15-12/h1-7,14H,8-9H2
**565**	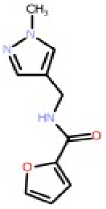	n.a.	514843-64-2	Z275179758	bmse011724	InChI=1S/C10H11N3O2/c1-13-7-8(6-12-13)5-11-10(14)9-3-2-4-15-9/h2-4,6-7H,5H2,1H3,(H,11,14)

Suppl Table 2: Details of the 768 compounds from the DSI-Poised fragment Library (DSI-PL)

Sl. No.	Structures	No. in Manuscript	CAS Registry Number	"ID"	^{1}H NMR Data Repository BMRB ID	ALATIS InChI
**566**	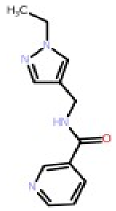	n.a.	514856-39-4	Z275181224	bmse011626	InChI=1S/C12H14N4O/c1-2-16-9-10(7-15-16)6-14-12(17)11-4-3-5-13-8-11/h3-5,7-9H,2,6H2,1H3,(H,14,17)
**567**	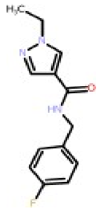	n.a.	515831-20-6	Z1203107138	bmse011699	InChI=1S/C13H14FN3O/c1-2-17-9-11(8-16-17)13(18)15-7-10-3-5-12(14)6-4-10/h3-6,8-9H,2,7H2,1H3,(H,15,18)
**568**	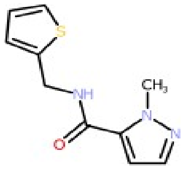	n.a.	515847-82-2	Z915492990	bmse011683	InChI=1S/C10H11N3OS/c1-13-9(4-5-12-13)10(14)11-7-8-3-2-6-15-8/h2-6H,7H2,1H3,(H,11,14)
**569**	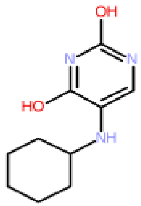	n.a.	5177-53-7	Z57283017	bmse011639	InChI=1S/C10H15N3O2/c14-9-8(6-11-10(15)13-9)12-7-4-2-1-3-5-7/h6-7,12H,1-5H2,(H2,11,13,14,15)
**570**	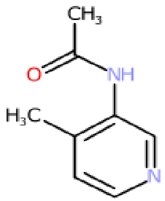	n.a.	52090-68-3	Z1129283193	bmse011158	InChI=1S/C8H10N2O/c1-6-3-4-9-5-8(6)10-7(2)11/h3-5H,1-2H3,(H,10,11)

Suppl Table 2: Details of the 768 compounds from the DSI-Poised fragment Library (DSI-PL)

Sl. No.	Structures	No. in Manuscript	CAS Registry Number	"ID"	1H NMR Data Repository BMRB ID	ALATIS InChI
**571**	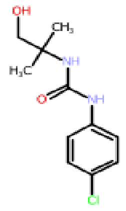	n.a.	522660-63-5	Z123970702	bmse011320	InChI=1S/C11H15ClN2O2/c1-11(2,7-15)14-10(16)13-9-5-3-8(12)4-6-9/h3-6,15H,7H2,1-2H3,(H2,13,14,16)
**572**	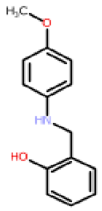	n.a.	52537-88-9	Z56943440	bmse011439	InChI=1S/C14H15NO2/c1-17-13-8-6-12(7-9-13)15-10-11-4-2-3-5-14(11)16/h2-9,15-16H,10H2,1H3
**573**	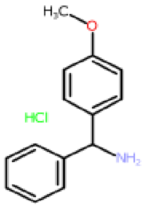	n.a.	5267-46-9	Z2574937229	bmse011751	InChI=1S/C14H15NO.ClH/c1-16-13-9-7-12(8-10-13)14(15)11-5-3-2-4-6-11;/h2-10,14H,15H2,1H3;1H/t14-;/m1./s1
**574**	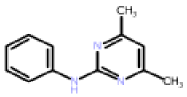	n.a.	53112-28-0	Z285675722	bmse011367	InChI=1S/C12H13N3/c1-9-8-10(2)14-12(13-9)15-11-6-4-3-5-7-11/h3-8H,1-2H3,(H,13,14,15)
**575**	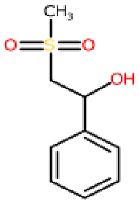	n.a.	5324-56-1	Z822382694	bmse011260	InChI=1S/C9H12O3S/c1-13(11,12)7-9(10)8-5-3-2-4-6-8/h2-6,9-10H,7H2,1H3/t9-/m0/s1

Suppl Table 2: Details of the 768 compounds from the DSI-Poised fragment Library (DSI-PL)

Sl. No.	Structures	No. in Manuscript	CAS Registry Number	"ID"	^{1}H NMR Data Repository BMRB ID	ALATIS InChI
**576**	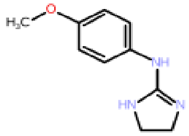	n.a.	53976-95-7	Z57186564	bmse011100	InChI=1S/C10H13N3O/c1-14-9-4-2-8(3-5-9)13-10-11-6-7-12-10/h2-5H,6-7H2,1H3,(H2,11,12,13)
**577**	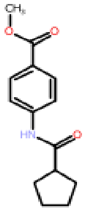	n.a.	540531-66-6	Z28226359	bmse011066	InChI=1S/C14H17NO3/c1-18-14(17)11-6-8-12(9-7-11)15-13(16)10-4-2-3-5-10/h6-10H,2-5H2,1H3,(H,15,16)
**578**	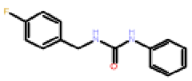	n.a.	541545-68-0	Z44609285	bmse011292	InChI=1S/C14H13FN2O/c15-12-8-6-11(7-9-12)10-16-14(18)17-13-4-2-1-3-5-13/h1-9H,10H2,(H2,16,17,18)
**579**	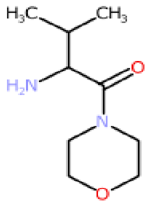	n.a.	54164-04-4	Z927400026	bmse011596	InChI=1S/C9H18N2O2/c1-7(2)8(10)9(12)11-3-5-13-6-4-11/h7-8H,3-6,10H2,1-2H3/t8-/m0/s1
**580**	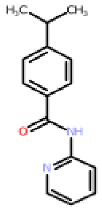	n.a.	543706-26-9	Z85525355	bmse011365	InChI=1S/C15H16N2O/c1-11(2)12-6-8-13(9-7-12)15(18)17-14-5-3-4-10-16-14/h3-11H,1-2H3,(H,16,17,18)

Suppl Table 2: Details of the 768 compounds from the DSI-Poised fragment Library (DSI-PL)

Sl. No.	Structures	No. in Manuscript	CAS Registry Number	"ID"	^{1}H NMR Data Repository BMRB ID	ALATIS InChI
**581**	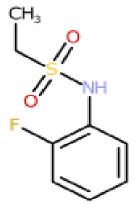	n.a.	544662-80-8	Z53825177	bmse011408	InChI=1S/C8H10FNO2S/c1-2-13(11,12)10-8-6-4-3-5-7(8)9/h3-6,10H,2H2,1H3
**582**	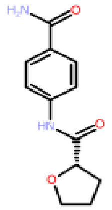	n.a.	544668-49-7	Z1545313172	bmse011346	InChI=1S/C12H14N2O3/c13-11(15)8-3-5-9(6-4-8)14-12(16)10-2-1-7-17-10/h3-6,10H,1-2,7H2,(H2,13,15)(H,14,16)/t10-/m0/s1
**583**	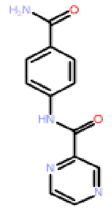	n.a.	544670-58-8	Z30242120	bmse011017	InChI=1S/C12H10N4O2/c13-11(17)8-1-3-9(4-2-8)16-12(18)10-7-14-5-6-15-10/h1-7H,(H2,13,17)(H,16,18)
**584**	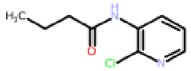	n.a.	546070-44-4	Z240654968	bmse011665	InChI=1S/C9H11ClN2O/c1-2-4-8(13)12-7-5-3-6-11-9(7)10/h3,5-6H,2,4H2,1H3,(H,12,13)
**585**	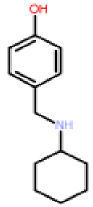	n.a.	5461-17-6	Z2241127906	bmse011246	InChI=1S/C13H19NO/c15-13-8-6-11(7-9-13)10-14-12-4-2-1-3-5-12/h6-9,12,14-15H,1-5,10H2

Suppl Table 2: Details of the 768 compounds from the DSI-Poised fragment Library (DSI-PL)

Sl. No.	Structures	No. in Manuscript	CAS Registry Number	"ID"	^{1}H NMR Data Repository BMRB ID	ALATIS InChI
**586**	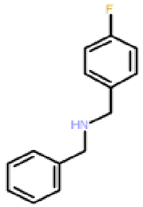	n.a.	55096-88-3	Z2856434783	bmse011753	InChI=1S/C14H14FN/c15-14-8-6-13(7-9-14)11-16-10-12-4-2-1-3-5-12/h1-9,16H,10-11H2
**587**	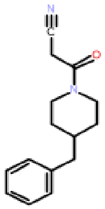	n.a.	551907-33-6	Z1688493887	bmse011691	InChI=1S/C15H18N2O/c16-9-6-15(18)17-10-7-14(8-11-17)12-13-4-2-1-3-5-13/h1-5,14H,6-8,10-12H2
**588**	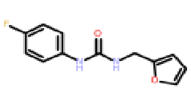	n.a.	551931-68-1	Z44584202	bmse011026	InChI=1S/C12H11FN2O2/c13-9-3-5-10(6-4-9)15-12(16)14-8-11-2-1-7-17-11/h1-7H,8H2,(H2,14,15,16)
**589**	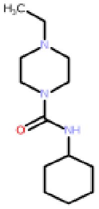	n.a.	553657-03-7	Z2856434830	bmse011671	InChI=1S/C13H25N3O/c1-2-15-8-10-16(11-9-15)13(17)14-12-6-4-3-5-7-12/h12H,2-11H2,1H3,(H,14,17)
**590**	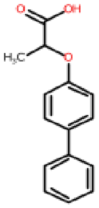	n.a.	5555-13-5	Z111782404	bmse011073	InChI=1S/C15H14O3/c1-11(15(16)17)18-14-9-7-13(8-10-14)12-5-3-2-4-6-12/h2-11H,1H3,(H,16,17)/t11-/m0/s1

Suppl Table 2: Details of the 768 compounds from the DSI-Poised fragment Library (DSI-PL)

Sl. No.	Structures	No. in Manuscript	CAS Registry Number	"ID"	^{1}H NMR Data Repository BMRB ID	ALATIS InChI
**591**	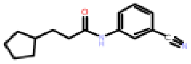	n.a.	556805-44-8	Z26548228	bmse011081	InChI=1S/C15H18N2O/c16-11-13-6-3-7-14(10-13)17-15(18)9-8-12-4-1-2-5-12/h3,6-7,10,12H,1-2,4-5,8-9H2,(H,17,18)
**592**	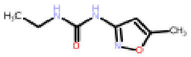	n.a.	55807-55-1	Z56880342	bmse011089	InChI=1S/C7H11N3O2/c1-3-8-7(11)9-6-4-5(2)12-10-6/h4H,3H2,1-2H3,(H2,8,9,10,11)
**593**	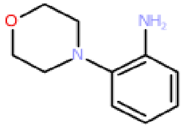	n.a.	5585-33-1	Z56785490	bmse011376	InChI=1S/C10H14N2O/c11-9-3-1-2-4-10(9)12-5-7-13-8-6-12/h1-4H,5-8,11H2
**594**	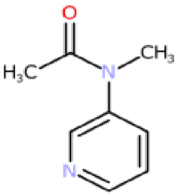	n.a.	55899-01-9	Z1203252645	bmse011598	InChI=1S/C8H10N2O/c1-7(11)10(2)8-4-3-5-9-6-8/h3-6H,1-2H3
**595**	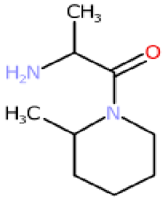	n.a.	56414-90-5	Z927412236	bmse011488	InChI=1S/C9H18N2O/c1-7-5-3-4-6-11(7)9(12)8(2)10/h7-8H,3-6,10H2,1-2H3/t7-,8-/m0/s1

Suppl Table 2: Details of the 768 compounds from the DSI-Poised fragment Library (DSI-PL)

Sl. No.	Structures	No. in Manuscript	CAS Registry Number	"ID"	1H NMR Data Repository BMRB ID	ALATIS InChI
596	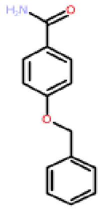	n.a.	56442-43-4	Z26312102	bmse011205	InChI=1S/C14H13NO2/c15-14(16)12-6-8-13(9-7-12)17-10-11-4-2-1-3-5-11/h1-9H,10H2,(H2,15,16)
597	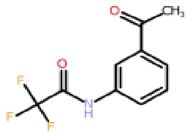	n.a.	56915-87-8	Z57111868	bmse011190	InChI=1S/C10H8F3NO2/c1-6(15)7-3-2-4-8(5-7)14-9(16)10(11,12)13/h2-5H,1H3,(H,14,16)
598	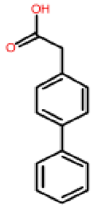	n.a.	5728-52-9	Z2856434779	bmse011053	InChI=1S/C14H12O2/c15-14(16)10-11-6-8-13(9-7-11)12-4-2-1-3-5-12/h1-9H,10H2,(H,15,16)
599	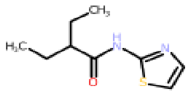	n.a.	574011-44-2	Z28173817	bmse011322	InChI=1S/C9H14N2OS/c1-3-7(4-2)8(12)11-9-10-5-6-13-9/h5-7H,3-4H2,1-2H3,(H,10,11,12)
600	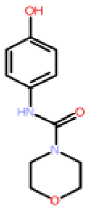	n.a.	57726-21-3	Z274554872	bmse011390	InChI=1S/C11H14N2O3/c14-10-3-1-9(2-4-10)12-11(15)13-5-7-16-8-6-13/h1-4,14H,5-8H2,(H,12,15)

Suppl Table 2: Details of the 768 compounds from the DSI-Poised fragment Library (DSI-PL)

Sl. No.	Structures	No. in Manuscript	CAS Registry Number	"ID"	^{1}H NMR Data Repository BMRB ID	ALATIS InChI
601	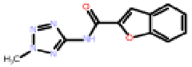	n.a.	577980-81-5	Z57292378	bmse011697	InChI=1S/C11H9N5O2/c1-16-14-11(13-15-16)12-10(17)9-6-7-4-2-3-5-8(7)18-9/h2-6H,1H3,(H,12,14,17)
602	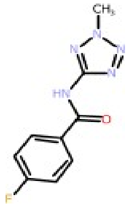	n.a.	578746-24-4	Z57292433	bmse011732	InChI=1S/C9H8FN5O/c1-15-13-9(12-14-15)11-8(16)6-2-4-7(10)5-3-6/h2-5H,1H3,(H,11,13,16)
603	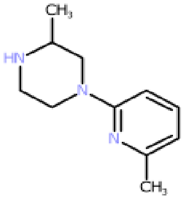	n.a.	582325-37-9	Z1267885772	bmse011521	InChI=1S/C11H17N3/c1-9-4-3-5-11(13-9)14-7-6-12-10(2)8-14/h3-5,10,12H,6-8H2,1-2H3/t10-/m1/s1
604	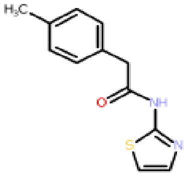	n.a.	587843-69-4	Z90713255	bmse011042	InChI=1S/C12H12N2OS/c1-9-2-4-10(5-3-9)8-11(15)14-12-13-6-7-16-12/h2-7H,8H2,1H3,(H,13,14,15)
605	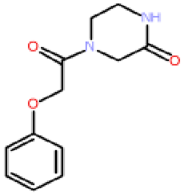	n.a.	587850-66-6	Z31602870	bmse011351	InChI=1S/C12H14N2O3/c15-11-8-14(7-6-13-11)12(16)9-17-10-4-2-1-3-5-10/h1-5H,6-9H2,(H,13,15)

Suppl Table 2: Details of the 768 compounds from the DSI-Poised fragment Library (DSI-PL)

Sl. No.	Structures	No. in Manuscript	CAS Registry Number	"ID"	1H NMR Data Repository BMRB ID	ALATIS InChI
**606**	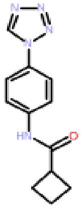	n.a.	606096-31-5	Z26552420	bmse011444	InChI=1S/C12H13N5O/c18-12(9-2-1-3-9)14-10-4-6-11(7-5-10)17-8-13-15-16-17/h4-9H,1-3H2,(H,14,18)
**607**	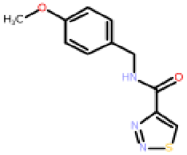	n.a.	606102-02-7	Z741218268	bmse011134	InChI=1S/C11H11N3O2S/c1-16-9-4-2-8(3-5-9)6-12-11(15)10-7-17-14-13-10/h2-5,7H,6H2,1H3,(H,12,15)
**608**	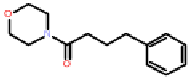	n.a.	61123-44-2	Z419995480	bmse011504	InChI=1S/C14H19NO2/c16-14(15-9-11-17-12-10-15)8-4-7-13-5-2-1-3-6-13/h1-3,5-6H,4,7-12H2
**609**	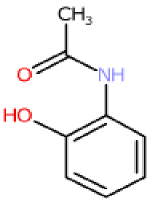	n.a.	614-80-2	Z57040482	bmse011002	InChI=1S/C8H9NO2/c1-6(10)9-7-4-2-3-5-8(7)11/h2-5,11H,1H3,(H,9,10)
**610**	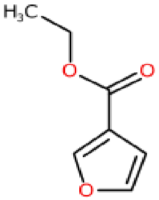	n.a.	614-98-2	Z293079056	bmse011642	InChI=1S/C7H8O3/c1-2-10-7(8)6-3-4-9-5-6/h3-5H,2H2,1H3

Suppl Table 2: Details of the 768 compounds from the DSI-Poised fragment Library (DSI-PL)

Sl. No.	Structures	No. in Manuscript	CAS Registry Number	"ID"	^{1}H NMR Data Repository BMRB ID	ALATIS InChI
**611**	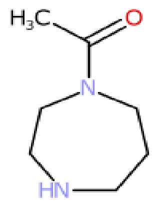	n.a.	61903-11-5	Z168883358	bmse011561	InChI=1S/C7H14N2O/c1-7(10)9-5-2-3-8-4-6-9/h8H,2-6H2,1H3
**612**	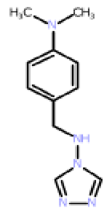	n.a.	6213-03-2	Z57258487	bmse011733	InChI=1S/C11H15N5/c1-15(2)11-5-3-10(4-6-11)7-14-16-8-12-13-9-16/h3-6,8-9,14H,7H2,1-2H3
**613**	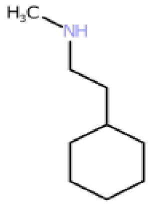	n.a.	62141-38-2	Z2856434901	bmse011576	InChI=1S/C9H19N/c1-10-8-7-9-5-3-2-4-6-9/h9-10H,2-8H2,1H3
**614**	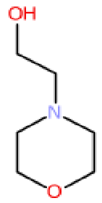	n.a.	622-40-2	Z2856434843	bmse011532	InChI=1S/C6H13NO2/c8-4-1-7-2-5-9-6-3-7/h8H,1-6H2
**615**	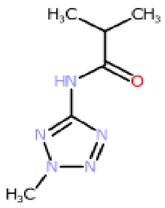	n.a.	62400-57-1	Z57292369	bmse011739	InChI=1S/C6H11N5O/c1-4(2)5(12)7-6-8-10-11(3)9-6/h4H,1-3H3,(H,7,9,12)

Suppl Table 2: Details of the 768 compounds from the DSI-Poised fragment Library (DSI-PL)

Sl. No.	Structures	No. in Manuscript	CAS Registry Number	"ID"	^{1}H NMR Data Repository BMRB ID	ALATIS InChI
**616**	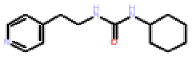	n.a.	626222-65-9	Z111507846	bmse011191	InChI=1S/C14H21N3O/c18-14(17-13-4-2-1-3-5-13)16-11-8-12-6-9-15-10-7-12/h6-7,9-10,13H,1-5,8,11H2,(H2,16,17,18)
**617**	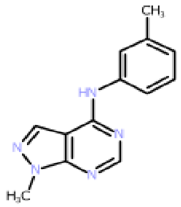	n.a.	6289-08-3	Z56983806	bmse011289	InChI=1S/C13H13N5/c1-9-4-3-5-10(6-9)17-12-11-7-16-18(2)13(11)15-8-14-12/h3-8H,1-2H3,(H,14,15,17)
**618**	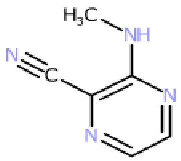	n.a.	63352-06-7	Z1896597864	bmse011138	InChI=1S/C6H6N4/c1-8-6-5(4-7)9-2-3-10-6/h2-3H,1H3,(H,8,10)
**619**	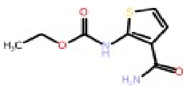	n.a.	63895-76-1	Z64450924	bmse011730	InChI=1S/C8H10N2O3S/c1-2-13-8(12)10-7-5(6(9)11)3-4-14-7/h3-4H,2H2,1H3,(H2,9,11)(H,10,12)
**620**	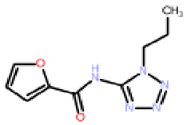	n.a.	639046-93-8	Z57446103	bmse011041	InChI=1S/C9H11N5O2/c1-2-5-14-9(11-12-13-14)10-8(15)7-4-3-6-16-7/h3-4,6H,2,5H2,1H3,(H,10,11,13,15)

Suppl Table 2: Details of the 768 compounds from the DSI-Poised fragment Library (DSI-PL)

Sl. No.	Structures	No. in Manuscript	CAS Registry Number	"ID"	^{1}H NMR Data Repository BMRB ID	ALATIS InChI
**621**	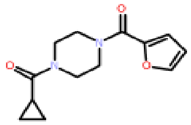	n.a.	6391-74-8	Z32327641	bmse011335	InChI=1S/C13H16N2O3/c16-12(10-3-4-10)14-5-7-15(8-6-14)13(17)11-2-1-9-18-11/h1-2,9-10H,3-8H2
**622**	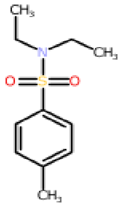	n.a.	649-15-0	Z45516134	bmse011413	InChI=1S/C11H17NO2S/c1-4-12(5-2)15(13,14)11-8-6-10(3)7-9-11/h6-9H,4-5H2,1-3H3
**623**	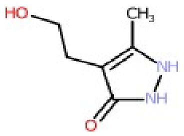	n.a.	65287-96-9	Z1259155959	bmse011263	InChI=1S/C6H10N2O2/c1-4-5(2-3-9)6(10)8-7-4/h9H,2-3H2,1H3,(H2,7,8,10)
**624**	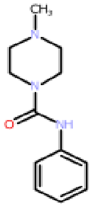	n.a.	65766-72-5	Z2856434890	bmse011340	InChI=1S/C12H17N3O/c1-14-7-9-15(10-8-14)12(16)13-11-5-3-2-4-6-11/h2-6H,7-10H2,1H3,(H,13,16)
**625**	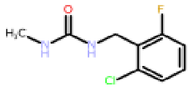	n.a.	660849-84-3	Z933840894	bmse011295	InChI=1S/C9H10ClFN2O/c1-12-9(14)13-5-6-7(10)3-2-4-8(6)11/h2-4H,5H2,1H3,(H2,12,13,14)

Suppl Table 2: Details of the 768 compounds from the DSI-Poised fragment Library (DSI-PL)

Sl. No.	Structures	No. in Manuscript	CAS Registry Number	"ID"	^{1}H NMR Data Repository BMRB ID	ALATIS InChI
**626**	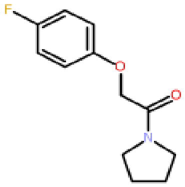	n.a.	667881-38-1	Z19735067	bmse011076	InChI=1S/C12H14FNO2/c13-10-3-5-11(6-4-10)16-9-12(15)14-7-1-2-8-14/h3-6H,1-2,7-9H2
**627**	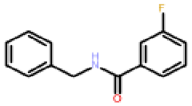	n.a.	671-01-2	Z27749656	bmse011316	InChI=1S/C14H12FNO/c15-13-8-4-7-12(9-13)14(17)16-10-11-5-2-1-3-6-11/h1-9H,10H2,(H,16,17)
**628**	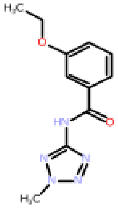	n.a.	673453-78-6	Z57292400	bmse011723	InChI=1S/C11H13N5O2/c1-3-18-9-6-4-5-8(7-9)10(17)12-11-13-15-16(2)14-11/h4-7H,3H2,1-2H3,(H,12,14,17)
**629**	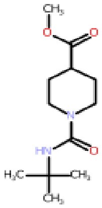	n.a.	675121-22-9	Z44567722	bmse011297	InChI=1S/C12H22N2O3/c1-12(2,3)13-11(16)14-7-5-9(6-8-14)10(15)17-4/h9H,5-8H2,1-4H3,(H,13,16)
**630**	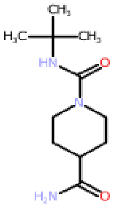	n.a.	676146-84-2	Z44603686	bmse011682	InChI=1S/C11H21N3O2/c1-11(2,3)13-10(16)14-6-4-8(5-7-14)9(12)15/h8H,4-7H2,1-3H3,(H2,12,15)(H,13,16)

Suppl Table 2: Details of the 768 compounds from the DSI-Poised fragment Library (DSI-PL)

Sl. No.	Structures	No. in Manuscript	CAS Registry Number	"ID"	^{1}H NMR Data Repository BMRB ID	ALATIS InChI
**631**	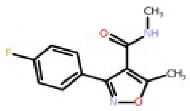	n.a.	67764-99-2	Z363104204	bmse011583	InChI=1S/C12H11FN2O2/c1-7-10(12(16)14-2)11(15-17-7)8-3-5-9(13)6-4-8/h3-6H,1-2H3,(H,14,16)
**632**	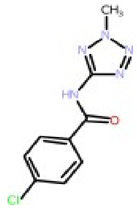	n.a.	679824-05-6	Z57292434	bmse011741	InChI=1S/C9H8ClN5O/c1-15-13-9(12-14-15)11-8(16)6-2-4-7(10)5-3-6/h2-5H,1H3,(H,11,13,16)
**633**	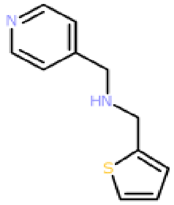	n.a.	680185-81-3	Z2856434816	bmse011761	InChI=1S/C11H12N2S/c1-2-11(14-7-1)9-13-8-10-3-5-12-6-4-10/h1-7,13H,8-9H2
**634**	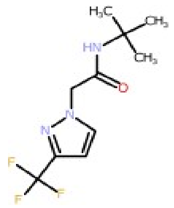	n.a.	680203-49-0	Z55009317	bmse011482	InChI=1S/C10H14F3N3O/c1-9(2,3)14-8(17)6-16-5-4-7(15-16)10(11,12)13/h4-5H,6H2,1-3H3,(H,14,17)
**635**	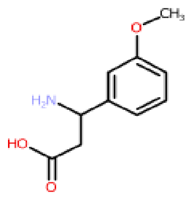	n.a.	68208-19-5	Z335451386	bmse011067	InChI=1S/C10H13NO3/c1-14-8-4-2-3-7(5-8)9(11)6-10(12)13/h2-5,9H,6,11H2,1H3,(H,12,13)/t9-/m0/s1

Suppl Table 2: Details of the 768 compounds from the DSI-Poised fragment Library (DSI-PL)

Sl. No.	Structures	No. in Manuscript	CAS Registry Number	"ID"	1H NMR Data Repository BMRB ID	ALATIS InChI
**636**	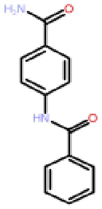	n.a.	68688-78-8	Z30242076	bmse011393	InChI=1S/C14H12N2O2/c15-13(17)10-6-8-12(9-7-10)16-14(18)11-4-2-1-3-5-11/h1-9H,(H2,15,17)(H,16,18)
**637**	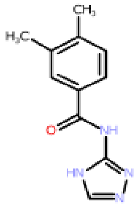	n.a.	690981-54-5	Z2856434827	bmse011179	InChI=1S/C11H12N4O/c1-7-3-4-9(5-8(7)2)10(16)14-11-12-6-13-15-11/h3-6H,1-2H3,(H2,12,13,14,15,16)
**638**	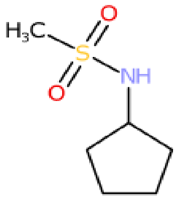	n.a.	69200-54-0	Z53825479	bmse011188	InChI=1S/C6H13NO2S/c1-10(8,9)7-6-4-2-3-5-6/h6-7H,2-5H2,1H3
**639**	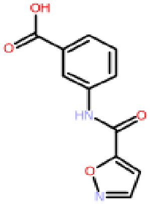	n.a.	693776-70-4	Z2856434931	bmse011637	InChI=1S/C11H8N2O4/c14-10(9-4-5-12-17-9)13-8-3-1-2-7(6-8)11(15)16/h1-6H,(H,13,14)(H,15,16)
**640**	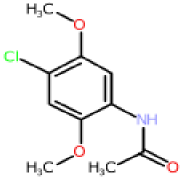	n.a.	6938-75-6	Z26968795	bmse011078	InChI=1S/C10H12ClNO3/c1-6(13)12-8-5-9(14-2)7(11)4-10(8)15-3/h4-5H,1-3H3,(H,12,13)

Suppl Table 2: Details of the 768 compounds from the DSI-Poised fragment Library (DSI-PL)

Sl. No.	Structures	No. in Manuscript	CAS Registry Number	"ID"	^{1}H NMR Data Repository BMRB ID	ALATIS InChI
**641**	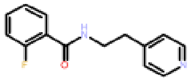	n.a.	696629-69-3	Z1310876699	bmse011330	InChI=1S/C14H13FN2O/c15-13-4-2-1-3-12(13)14(18)17-10-7-11-5-8-16-9-6-11/h1-6,8-9H,7,10H2,(H,17,18)
**642**	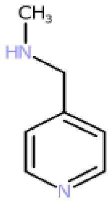	n.a.	6971-44-4	Z2856434786	bmse011735	InChI=1S/C7H10N2/c1-8-6-7-2-4-9-5-3-7/h2-5,8H,6H2,1H3
**643**	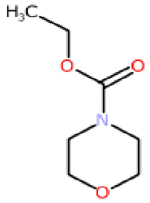	n.a.	6976-49-4	Z31721097	bmse011538	InChI=1S/C7H13NO3/c1-2-11-7(9)8-3-5-10-6-4-8/h2-6H2,1H3
**644**	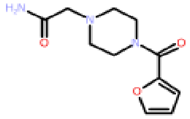	n.a.	697770-37-9	Z2856434793	bmse011181	InChI=1S/C11H15N3O3/c12-10(15)8-13-3-5-14(6-4-13)11(16)9-2-1-7-17-9/h1-2,7H,3-6,8H2,(H2,12,15)
**645**	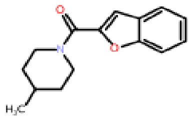	n.a.	701224-39-7	Z32665176	bmse011334	InChI=1S/C15H17NO2/c1-11-6-8-16(9-7-11)15(17)14-10-12-4-2-3-5-13(12)18-14/h2-5,10-11H,6-9H2,1H3

Suppl Table 2: Details of the 768 compounds from the DSI-Poised fragment Library (DSI-PL)

Sl. No.	Structures	No. in Manuscript	CAS Registry Number	"ID"	^{1}H NMR Data Repository BMRB ID	ALATIS InChI
**646**	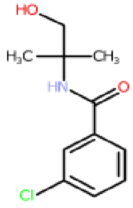	n.a.	701923-86-6	Z68299550	bmse011308	InChI=1S/C11H14ClNO2/c1-11(2,7-14)13-10(15)8-4-3-5-9(12)6-8/h3-6,14H,7H2,1-2H3,(H,13,15)
**647**	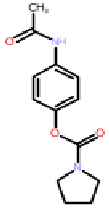	n.a.	701973-29-7	Z274555566	bmse011389	InChI=1S/C13H16N2O3/c1-10(16)14-11-4-6-12(7-5-11)18-13(17)15-8-2-3-9-15/h4-7H,2-3,8-9H2,1H3,(H,14,16)
**648**	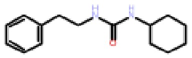	n.a.	70243-17-3	Z44585920	bmse011380	InChI=1S/C15H22N2O/c18-15(17-14-9-5-2-6-10-14)16-12-11-13-7-3-1-4-8-13/h1,3-4,7-8,14H,2,5-6,9-12H2,(H2,16,17,18)
**649**	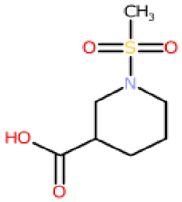	n.a.	702670-29-9	Z133632670	bmse011457	InChI=1S/C7H13NO4S/c1-13(11,12)8-4-2-3-6(5-8)7(9)10/h6H,2-5H2,1H3,(H,9,10)/t6-/m0/s1
**650**	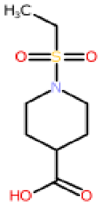	n.a.	702670-32-4	Z53825020	bmse011452	InChI=1S/C8H15NO4S/c1-2-14(12,13)9-5-3-7(4-6-9)8(10)11/h7H,2-6H2,1H3,(H,10,11)

Suppl Table 2: Details of the 768 compounds from the DSI-Poised fragment Library (DSI-PL)

Sl. No.	Structures	No. in Manuscript	CAS Registry Number	"ID"	^{1}H NMR Data Repository BMRB ID	ALATIS InChI
**651**	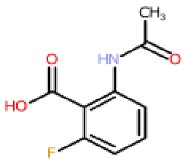	n.a.	70413-91-1	Z1229798311	bmse011265	InChI=1S/C9H8FNO3/c1-5(12)11-7-4-2-3-6(10)8(7)9(13)14/h2-4H,1H3,(H,11,12)(H,13,14)
**652**	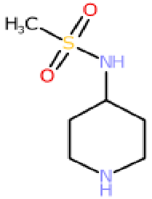	n.a.	70724-72-0	Z212045094	bmse011453	InChI=1S/C6H14N2O2S/c1-11(9,10)8-6-2-4-7-5-3-6/h6-8H,2-5H2,1H3
**653**	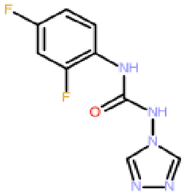	n.a.	708216-82-4	Z56259192	bmse011633	InChI=1S/C9H7F2N5O/c10-6-1-2-8(7(11)3-6)14-9(17)15-16-4-12-13-5-16/h1-5H,(H2,14,15,17)
**654**	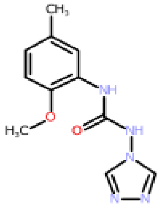	n.a.	708236-68-4	Z2234920345	bmse011731	InChI=1S/C11H13N5O2/c1-8-3-4-10(18-2)9(5-8)14-11(17)15-16-6-12-13-7-16/h3-7H,1-2H3,(H2,14,15,17)
**655**	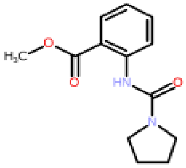	n.a.	708241-16-1	Z281077318	bmse011075	InChI=1S/C13H16N2O3/c1-18-12(16)10-6-2-3-7-11(10)14-13(17)15-8-4-5-9-15/h2-3,6-7H,4-5,8-9H2,1H3,(H,14,17)

Suppl Table 2: Details of the 768 compounds from the DSI-Poised fragment Library (DSI-PL)

Sl. No.	Structures	No. in Manuscript	CAS Registry Number	"ID"	1H NMR Data Repository BMRB ID	ALATIS InChI
**656**	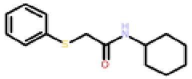	n.a.	71433-02-8	Z19751622	bmse011696	InChI=1S/C14H19NOS/c16-14(15-12-7-3-1-4-8-12)11-17-13-9-5-2-6-10-13/h2,5-6,9-10,12H,1,3-4,7-8,11H2,(H,15,16)
**657**	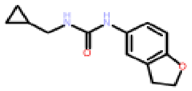	n.a.	717098-24-3	Z413792090	bmse011634	InChI=1S/C13H16N2O2/c16-13(14-8-9-1-2-9)15-11-3-4-12-10(7-11)5-6-17-12/h3-4,7,9H,1-2,5-6,8H2,(H2,14,15,16)
**658**	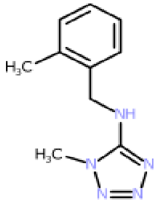	n.a.	717830-48-3	Z57778470	bmse011298	InChI=1S/C10H13N5/c1-8-5-3-4-6-9(8)7-11-10-12-13-14-15(10)2/h3-6H,7H2,1-2H3,(H,11,12,14)
**659**	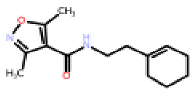	n.a.	717857-92-6	Z69092635	bmse011304	InChI=1S/C14H20N2O2/c1-10-13(11(2)18-16-10)14(17)15-9-8-12-6-4-3-5-7-12/h6H,3-5,7-9H2,1-2H3,(H,15,17)
**660**	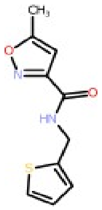	n.a.	717857-97-1	Z29692148	bmse011086	InChI=1S/C10H10N2O2S/c1-7-5-9(12-14-7)10(13)11-6-8-3-2-4-15-8/h2-5H,6H2,1H3,(H,11,13)

Suppl Table 2: Details of the 768 compounds from the DSI-Poised fragment Library (DSI-PL)

Sl. No.	Structures	No. in Manuscript	CAS Registry Number	"ID"	^{1}H NMR Data Repository BMRB ID	ALATIS InChI
**661**	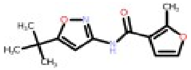	n.a.	717872-08-7	Z1086293874	bmse011685	InChI=1S/C13H16N2O3/c1-8-9(5-6-17-8)12(16)14-11-7-10(18-15-11)13(2,3)4/h5-7H,1-4H3,(H,14,15,16)
**662**	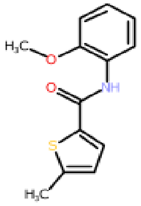	n.a.	717873-31-9	Z27797417	bmse011046	InChI=1S/C13H13NO2S/c1-9-7-8-12(17-9)13(15)14-10-5-3-4-6-11(10)16-2/h3-8H,1-2H3,(H,14,15)
**663**	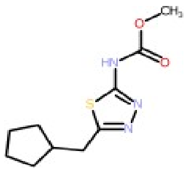	n.a.	717873-65-9	Z1119505742	bmse011355	InChI=1S/C10H15N3O2S/c1-15-10(14)11-9-13-12-8(16-9)6-7-4-2-3-5-7/h7H,2-6H2,1H3,(H,11,13,14)
**664**	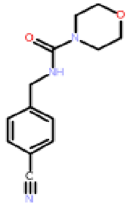	n.a.	720663-80-9	Z509756472	bmse011183	InChI=1S/C13H15N3O2/c14-9-11-1-3-12(4-2-11)10-15-13(17)16-5-7-18-8-6-16/h1-4H,5-8,10H2,(H,15,17)
**665**	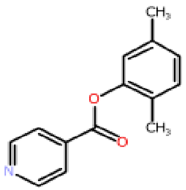	n.a.	720670-03-1	Z30891796	bmse011294	InChI=1S/C14H13NO2/c1-10-3-4-11(2)13(9-10)17-14(16)12-5-7-15-8-6-12/h3-9H,1-2H3

Suppl Table 2: Details of the 768 compounds from the DSI-Poised fragment Library (DSI-PL)

Sl. No.	Structures	No. in Manuscript	CAS Registry Number	"ID"	1H NMR Data Repository BMRB ID	ALATIS InChI
**666**	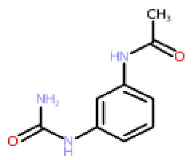	n.a.	720670-20-2	Z198195770	bmse011684	InChI=1S/C9H11N3O2/c1-6(13)11-7-3-2-4-8(5-7)12-9(10)14/h2-5H,1H3,(H,11,13)(H3,10,12,14)
**667**	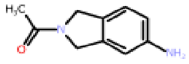	n.a.	722444-62-4	Z1354416068	bmse011394	InChI=1S/C10H12N2O/c1-7(13)12-5-8-2-3-10(11)4-9(8)6-12/h2-4H,5-6,11H2,1H3
**668**	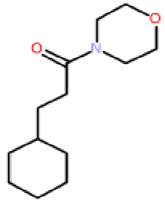	n.a.	72299-30-0	Z31721798	bmse011090	InChI=1S/C13H23NO2/c15-13(14-8-10-16-11-9-14)7-6-12-4-2-1-3-5-12/h12H,1-11H2
**669**	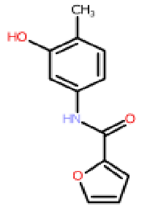	n.a.	723257-51-0	Z1430613384	bmse011307	InChI=1S/C12H11NO3/c1-8-4-5-9(7-10(8)14)13-12(15)11-3-2-6-16-11/h2-7,14H,1H3,(H,13,15)
**670**	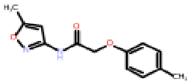	n.a.	723260-99-9	Z19731563	bmse011045	InChI=1S/C13H14N2O3/c1-9-3-5-11(6-4-9)17-8-13(16)14-12-7-10(2)18-15-12/h3-7H,8H2,1-2H3,(H,14,15,16)

Suppl Table 2: Details of the 768 compounds from the DSI-Poised fragment Library (DSI-PL)

Sl. No.	Structures	No. in Manuscript	CAS Registry Number	"ID"	1H NMR Data Repository BMRB ID	ALATIS InChI
**671**	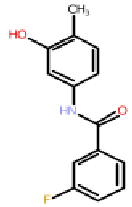	n.a.	723261-32-3	Z1430613393	bmse011309	InChI=1S/C14H12FNO2/c1-9-5-6-12(8-13(9)17)16-14(18)10-3-2-4-11(15)7-10/h2-8,17H,1H3,(H,16,18)
**672**	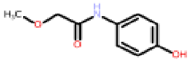	n.a.	723757-41-3	Z943693514	bmse011285	InChI=1S/C9H11NO3/c1-13-6-9(12)10-7-2-4-8(11)5-3-7/h2-5,11H,6H2,1H3,(H,10,12)
**673**	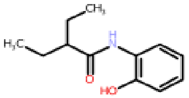	n.a.	723757-74-2	Z1331830630	bmse011728	InChI=1S/C12H17NO2/c1-3-9(4-2)12(15)13-10-7-5-6-8-11(10)14/h5-9,14H,3-4H2,1-2H3,(H,13,15)
**674**	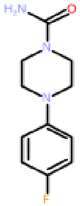	n.a.	724455-68-9	Z198194394	bmse011693	InChI=1S/C11H14FN3O/c12-9-1-3-10(4-2-9)14-5-7-15(8-6-14)11(13)16/h1-4H,5-8H2,(H2,13,16)
**675**	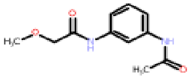	n.a.	724734-86-5	Z31735562	bmse011207	InChI=1S/C11H14N2O3/c1-8(14)12-9-4-3-5-10(6-9)13-11(15)7-16-2/h3-6H,7H2,1-2H3,(H,12,14)(H,13,15)

Suppl Table 2: Details of the 768 compounds from the DSI-Poised fragment Library (DSI-PL)

Sl. No.	Structures	No. in Manuscript	CAS Registry Number	"ID"	^{1}H NMR Data Repository BMRB ID	ALATIS InChI
**676**	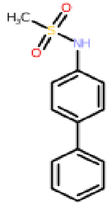	n.a.	724743-56-0	Z45641455	bmse011687	InChI=1S/C13H13NO2S/c1-17(15,16)14-13-9-7-12(8-10-13)11-5-3-2-4-6-11/h2-10,14H,1H3
**677**	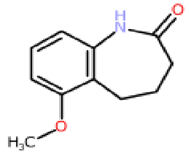	n.a.	72503-43-6	Z1507502062	bmse011409	InChI=1S/C11H13NO2/c1-14-10-6-3-5-9-8(10)4-2-7-11(13)12-9/h3,5-6H,2,4,7H2,1H3,(H,12,13)
**678**	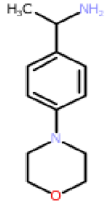	n.a.	728024-36-0	Z1259341012	bmse011266	InChI=1S/C12H18N2O/c1-10(13)11-2-4-12(5-3-11)14-6-8-15-9-7-14/h2-5,10H,6-9,13H2,1H3/t10-/m0/s1
**679**	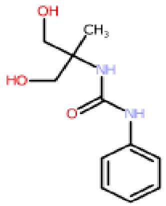	n.a.	74548-62-2	Z57472297	bmse011623	InChI=1S/C11H16N2O3/c1-11(7-14,8-15)13-10(16)12-9-5-3-2-4-6-9/h2-6,14-15H,7-8H2,1H3,(H2,12,13,16)
**680**	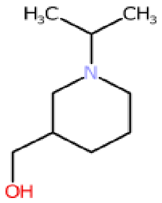	n.a.	752970-45-9	Z103740620	bmse011573	InChI=1S/C9H19NO/c1-8(2)10-5-3-4-9(6-10)7-11/h8-9,11H,3-7H2,1-2H3/t9-/m0/s1

Suppl Table 2: Details of the 768 compounds from the DSI-Poised fragment Library (DSI-PL)

Sl. No.	Structures	No. in Manuscript	CAS Registry Number	"ID"	^{1}H NMR Data Repository BMRB ID	ALATIS InChI
**681**	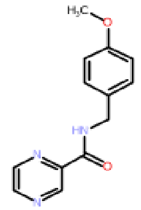	n.a.	75908-56-4	Z30871350	bmse011079	InChI=1S/C13H13N3O2/c1-18-11-4-2-10(3-5-11)8-16-13(17)12-9-14-6-7-15-12/h2-7,9H,8H2,1H3,(H,16,17)
**682**	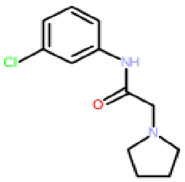	n.a.	75997-68-1	Z2735692823	bmse011011	InChI=1S/C12H15ClN2O/c13-10-4-3-5-11(8-10)14-12(16)9-15-6-1-2-7-15/h3-5,8H,1-2,6-7,9H2,(H,14,16)
**683**	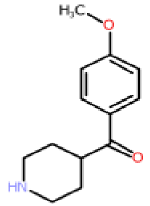	n.a.	76362-12-4	Z1741973467	bmse011018	InChI=1S/C13H17NO2/c1-16-12-4-2-10(3-5-12)13(15)11-6-8-14-9-7-11/h2-5,11,14H,6-9H2,1H3
**684**	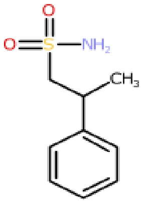	n.a.	76653-16-2	Z1407673036	bmse011400	InChI=1S/C9H13NO2S/c1-8(7-13(10,11)12)9-5-3-2-4-6-9/h2-6,8H,7H2,1H3,(H2,10,11,12)/t8-/m0/s1
**685**	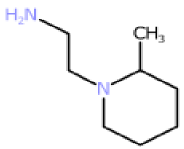	n.a.	768-08-1	Z1245580425	bmse011606	InChI=1S/C8H18N2/c1-8-4-2-3-6-10(8)7-5-9/h8H,2-7,9H2,1H3/t8-/m0/s1

Suppl Table 2: Details of the 768 compounds from the DSI-Poised fragment Library (DSI-PL)

Sl. No.	Structures	No. in Manuscript	CAS Registry Number	"ID"	^{1}H NMR Data Repository BMRB ID	ALATIS InChI
**686**	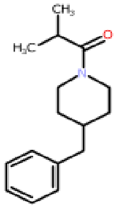	n.a.	77251-48-0	Z30162334	bmse011660	InChI=1S/C16H23NO/c1-13(2)16(18)17-10-8-15(9-11-17)12-14-6-4-3-5-7-14/h3-7,13,15H,8-12H2,1-2H3
**687**	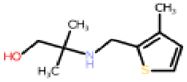	n.a.	774193-68-9	Z2856434821	bmse011700	InChI=1S/C10H17NOS/c1-8-4-5-13-9(8)6-11-10(2,3)7-12/h4-5,11-12H,6-7H2,1-3H3
**688**	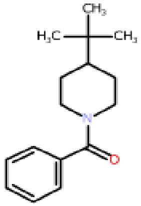	n.a.	787-50-8	Z1217741507	bmse011654	InChI=1S/C16H23NO/c1-16(2,3)14-9-11-17(12-10-14)15(18)13-7-5-4-6-8-13/h4-8,14H,9-12H2,1-3H3
**689**	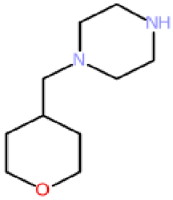	n.a.	787518-60-9	Z1267773591	bmse011553	InChI=1S/C10H20N2O/c1-7-13-8-2-10(1)9-12-5-3-11-4-6-12/h10-11H,1-9H2
**690**	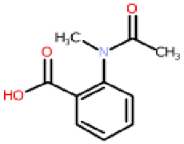	n.a.	78944-67-9	Z223688272	bmse011456	InChI=1S/C10H11NO3/c1-7(12)11(2)9-6-4-3-5-8(9)10(13)14/h3-6H,1-2H3,(H,13,14)

Suppl Table 2: Details of the 768 compounds from the DSI-Poised fragment Library (DSI-PL)

Sl. No.	Structures	No. in Manuscript	CAS Registry Number	"ID"	^{1}H NMR Data Repository BMRB ID	ALATIS InChI
**691**	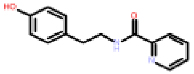	n.a.	791805-78-2	Z730649594	bmse011669	InChI=1S/C14H14N2O2/c17-12-6-4-11(5-7-12)8-10-16-14(18)13-3-1-2-9-15-13/h1-7,9,17H,8,10H2,(H,16,18)
**692**	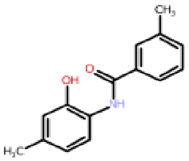	n.a.	791831-63-5	Z33452549	bmse011029	InChI=1S/C15H15NO2/c1-10-4-3-5-12(8-10)15(18)16-13-7-6-11(2)9-14(13)17/h3-9,17H,1-2H3,(H,16,18)
**693**	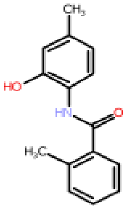	n.a.	791840-46-5	Z33452282	bmse011069	InChI=1S/C15H15NO2/c1-10-7-8-13(14(17)9-10)16-15(18)12-6-4-3-5-11(12)2/h3-9,17H,1-2H3,(H,16,18)
**694**	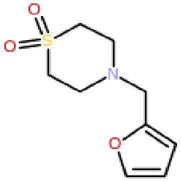	n.a.	79206-94-3	Z2856434926	bmse011640	InChI=1S/C9H13NO3S/c11-14(12)6-3-10(4-7-14)8-9-2-1-5-13-9/h1-2,5H,3-4,6-8H2
**695**	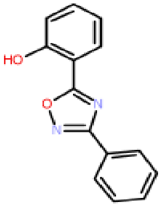	n.a.	79349-24-9	Z276351322	n.a.	n.a.

Suppl Table 2: Details of the 768 compounds from the DSI-Poised fragment Library (DSI-PL)

Sl. No.	Structures	No. in Manuscript	CAS Registry Number	"ID"	^{1}H NMR Data Repository BMRB ID	ALATIS InChI
**696**	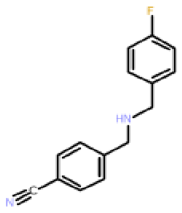	n.a.	797769-58-5	Z2856434851	bmse011653	InChI=1S/C15H13FN2/c16-15-7-5-14(6-8-15)11-18-10-13-3-1-12(9-17)2-4-13/h1-8,18H,10-11H2
**697**	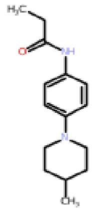	n.a.	797777-96-9	Z57344995	bmse011364	InChI=1S/C15H22N2O/c1-3-15(18)16-13-4-6-14(7-5-13)17-10-8-12(2)9-11-17/h4-7,12H,3,8-11H2,1-2H3,(H,16,18)
**698**	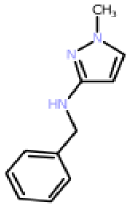	n.a.	81574-05-2	Z1401333862	bmse011526	InChI=1S/C11H13N3/c1-14-8-7-11(13-14)12-9-10-5-3-2-4-6-10/h2-8H,9H2,1H3,(H,12,13)
**699**	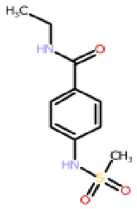	n.a.	824974-96-1	Z57614330	bmse011729	InChI=1S/C10H14N2O3S/c1-3-11-10(13)8-4-6-9(7-5-8)12-16(2,14)15/h4-7,12H,3H2,1-2H3,(H,11,13)
**700**	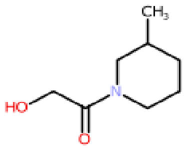	n.a.	82554-10-7	Z228585534	bmse011566	InChI=1S/C8H15NO2/c1-7-3-2-4-9(5-7)8(11)6-10/h7,10H,2-6H2,1H3/t7-/m0/s1

Suppl Table 2: Details of the 768 compounds from the DSI-Poised fragment Library (DSI-PL)

Sl. No.	Structures	No. in Manuscript	CAS Registry Number	"ID"	^{1}H NMR Data Repository BMRB ID	ALATIS InChI
**701**	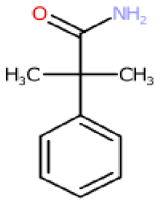	n.a.	826-54-0	Z270979606	bmse011161	InChI=1S/C10H13NO/c1-10(2,9(11)12)8-6-4-3-5-7-8/h3-7H,1-2H3,(H2,11,12)
**702**	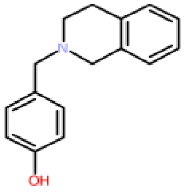	n.a.	82965-56-8	Z2856434812	bmse011625	InChI=1S/C16H17NO/c18-16-7-5-13(6-8-16)11-17-10-9-14-3-1-2-4-15(14)12-17/h1-8,18H,9-12H2
**703**	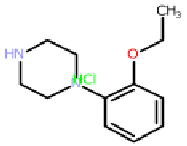	n.a.	83081-75-8	Z425387594	bmse011092	InChI=1S/C12H18N2O.ClH/c1-2-15-12-6-4-3-5-11(12)14-9-7-13-8-10-14;/h3-6,13H,2,7-10H2,1H3;1H
**704**	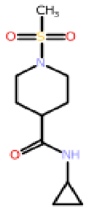	n.a.	832119-05-8	Z227998000	bmse011354	InChI=1S/C10H18N2O3S/c1-16(14,15)12-6-4-8(5-7-12)10(13)11-9-2-3-9/h8-9H,2-7H2,1H3,(H,11,13)
**705**	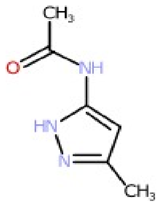	n.a.	83725-05-7	Z239136710	bmse011171	InChI=1S/C6H9N3O/c1-4-3-6(9-8-4)7-5(2)10/h3H,1-2H3,(H2,7,8,9,10)

Suppl Table 2: Details of the 768 compounds from the DSI-Poised fragment Library (DSI-PL)

Sl. No.	Structures	No. in Manuscript	CAS Registry Number	"ID"	^{1}H NMR Data Repository BMRB ID	ALATIS InChI
**706**	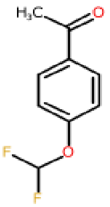	n.a.	83882-67-1	Z55671900	bmse011529	InChI=1S/C9H8F2O2/c1-6(12)7-2-4-8(5-3-7)13-9(10)11/h2-5,9H,1H3
**707**	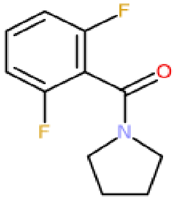	n.a.	84044-77-9	Z54226006	bmse011231	InChI=1S/C11H11F2NO/c12-8-4-3-5-9(13)10(8)11(15)14-6-1-2-7-14/h3-5H,1-2,6-7H2
**708**	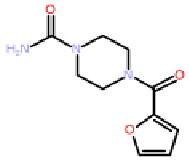	n.a.	84655-78-7	Z198194396	bmse011690	InChI=1S/C10H13N3O3/c11-10(15)13-5-3-12(4-6-13)9(14)8-2-1-7-16-8/h1-2,7H,3-6H2,(H2,11,15)
**709**	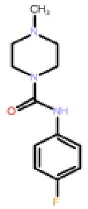	n.a.	84882-87-1	Z2856434944	bmse011056	InChI=1S/C12H16FN3O/c1-15-6-8-16(9-7-15)12(17)14-11-4-2-10(13)3-5-11/h2-5H,6-9H2,1H3,(H,14,17)
**710**	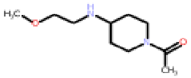	n.a.	849021-41-6	Z2856434813	bmse011648	InChI=1S/C10H20N2O2/c1-9(13)12-6-3-10(4-7-12)11-5-8-14-2/h10-11H,3-8H2,1-2H3

Suppl Table 2: Details of the 768 compounds from the DSI-Poised fragment Library (DSI-PL)

Sl. No.	Structures	No. in Manuscript	CAS Registry Number	"ID"	^{1}H NMR Data Repository BMRB ID	ALATIS InChI
**711**	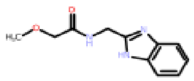	n.a.	850921-03-8	Z111529496	bmse011227	InChI=1S/C11H13N3O2/c1-16-7-11(15)12-6-10-13-8-4-2-3-5-9(8)14-10/h2-5H,6-7H2,1H3,(H,12,15)(H,13,14)
**712**	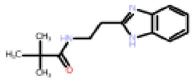	n.a.	850923-38-5	Z111716368	bmse011010	InChI=1S/C14H19N3O/c1-14(2,3)13(18)15-9-8-12-16-10-6-4-5-7-11(10)17-12/h4-7H,8-9H2,1-3H3,(H,15,18)(H,16,17)
**713**	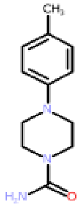	n.a.	85474-81-3	Z1650868495	bmse011332	InChI=1S/C12H17N3O/c1-10-2-4-11(5-3-10)14-6-8-15(9-7-14)12(13)16/h2-5H,6-9H2,1H3,(H2,13,16)
**714**	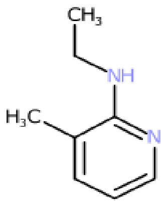	n.a.	856836-17-4	Z1992316287	bmse011564	InChI=1S/C8H12N2/c1-3-9-8-7(2)5-4-6-10-8/h4-6H,3H2,1-2H3,(H,9,10)
**715**	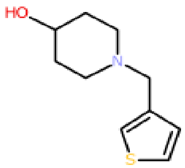	n.a.	864388-84-1	Z2856434899	bmse011274	InChI=1S/C10H15NOS/c12-10-1-4-11(5-2-10)7-9-3-6-13-8-9/h3,6,8,10,12H,1-2,4-5,7H2

Suppl Table 2: Details of the 768 compounds from the DSI-Poised fragment Library (DSI-PL)

Sl. No.	Structures	No. in Manuscript	CAS Registry Number	"ID"	^{1}H NMR Data Repository BMRB ID	ALATIS InChI
716	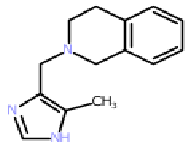	n.a.	864416-90-0	Z2856434879	bmse011705	InChI=1S/C14H17N3/c1-11-14(16-10-15-11)9-17-7-6-12-4-2-3-5-13(12)8-17/h2-5,10H,6-9H2,1H3,(H,15,16)
717	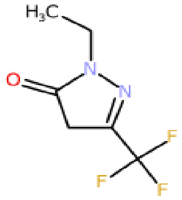	n.a.	866472-53-9	Z1268152398	bmse011038	InChI=1S/C6H7F3N2O/c1-2-11-5(12)3-4(10-11)6(7,8)9/h2-3H2,1H3
718	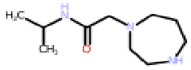	n.a.	87055-39-8	Z1262254278	bmse011603	InChI=1S/C10H21N3O/c1-9(2)12-10(14)8-13-6-3-4-11-5-7-13/h9,11H,3-8H2,1-2H3,(H,12,14)
719	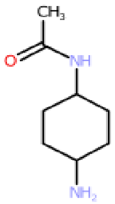	n.a.	873537-23-6	Z1787158625	bmse011462	InChI=1S/C8H16N2O/c1-6(11)10-8-4-2-7(9)3-5-8/h7-8H,2-5,9H2,1H3,(H,10,11)/t7-,8-
720	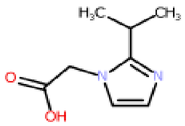	n.a.	876717-08-7	Z2446040567	bmse011162	InChI=1S/C8H12N2O2/c1-6(2)8-9-3-4-10(8)5-7(11)12/h3-4,6H,5H2,1-2H3,(H,11,12)

Suppl Table 2: Details of the 768 compounds from the DSI-Poised fragment Library (DSI-PL)

Sl. No.	Structures	No. in Manuscript	CAS Registry Number	"ID"	^{1}H NMR Data Repository BMRB ID	ALATIS InChI
**721**	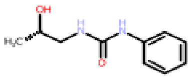	n.a.	87919-24-2	Z1563512128	bmse011369	InChI=1S/C10H14N2O2/c1-8(13)7-11-10(14)12-9-5-3-2-4-6-9/h2-6,8,13H,7H2,1H3,(H2,11,12,14)/t8-/m0/s1
**722**	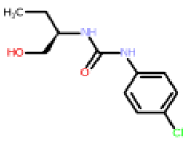	n.a.	87919-30-0	Z1593306637	bmse011370	InChI=1S/C11H15ClN2O2/c1-2-9(7-15)13-11(16)14-10-5-3-8(12)4-6-10/h3-6,9,15H,2,7H2,1H3,(H2,13,14,16)/t9-/m1/s1
**723**	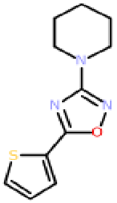	n.a.	883013-61-4	Z1623890017	bmse011646	InChI=1S/C11H13N3OS/c1-2-6-14(7-3-1)11-12-10(15-13-11)9-5-4-8-16-9/h4-5,8H,1-3,6-7H2
**724**	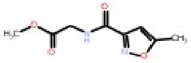	n.a.	883043-92-3	Z375990520	bmse011649	InChI=1S/C8H10N2O4/c1-5-3-6(10-14-5)8(12)9-4-7(11)13-2/h3H,4H2,1-2H3,(H,9,12)
**725**	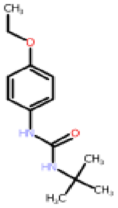	n.a.	883095-34-9	Z44586802	bmse011343	InChI=1S/C13H20N2O2/c1-5-17-11-8-6-10(7-9-11)14-12(16)15-13(2,3)4/h6-9H,5H2,1-4H3,(H2,14,15,16)

Suppl Table 2: Details of the 768 compounds from the DSI-Poised fragment Library (DSI-PL)

Sl. No.	Structures	No. in Manuscript	CAS Registry Number	"ID"	^{1}H NMR Data Repository BMRB ID	ALATIS InChI
**726**	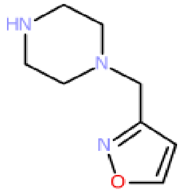	n.a.	885952-54-5	Z1263714198	bmse011610	InChI=1S/C8H13N3O/c1-6-12-10-8(1)7-11-4-2-9-3-5-11/h1,6,9H,2-5,7H2
**727**	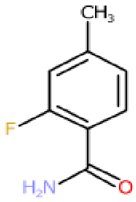	n.a.	886761-61-1	Z1343633025	bmse011399	InChI=1S/C8H8FNO/c1-5-2-3-6(8(10)11)7(9)4-5/h2-4H,1H3,(H2,10,11)
**728**	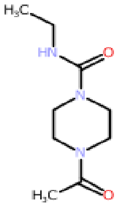	n.a.	887577-54-0	Z44590919	bmse011174	InChI=1S/C9H17N3O2/c1-3-10-9(14)12-6-4-11(5-7-12)8(2)13/h3-7H2,1-2H3,(H,10,14)
**729**	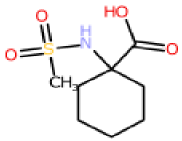	n.a.	887842-05-9	Z133622412	bmse011197	InChI=1S/C8H15NO4S/c1-14(12,13)9-8(7(10)11)5-3-2-4-6-8/h9H,2-6H2,1H3,(H,10,11)
**730**	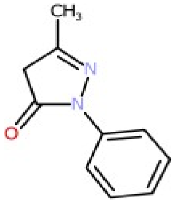	n.a.	89-25-8	Z50145861	bmse011258	InChI=1S/C10H10N2O/c1-8-7-10(13)12(11-8)9-5-3-2-4-6-9/h2-6H,7H2,1H3

Suppl Table 2: Details of the 768 compounds from the DSI-Poised fragment Library (DSI-PL)

Sl. No.	Structures	No. in Manuscript	CAS Registry Number	"ID"	^{1}H NMR Data Repository BMRB ID	ALATIS InChI
**731**	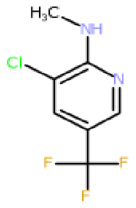	n.a.	89810-01-5	Z54628157	bmse011747	InChI=1S/C7H6ClF3N2/c1-12-6-5(8)2-4(3-13-6)7(9,10)11/h2-3H,1H3,(H,12,13)
**732**	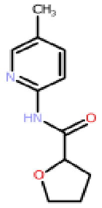	n.a.	899020-94-1	Z71580604	bmse011530	InChI=1S/C11H14N2O2/c1-8-4-5-10(12-7-8)13-11(14)9-3-2-6-15-9/h4-5,7,9H,2-3,6H2,1H3,(H,12,13,14)/t9-/m1/s1
**733**	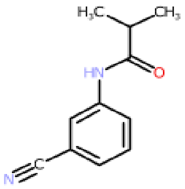	n.a.	900710-59-0	Z26548083	bmse011019	InChI=1S/C11H12N2O/c1-8(2)11(14)13-10-5-3-4-9(6-10)7-12/h3-6,8H,1-2H3,(H,13,14)
**734**	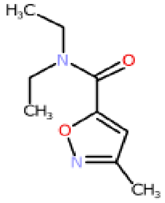	n.a.	902623-58-9	Z135439900	bmse011595	InChI=1S/C9H14N2O2/c1-4-11(5-2)9(12)8-6-7(3)10-13-8/h6H,4-5H2,1-3H3
**735**	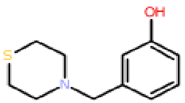	n.a.	90287-65-3	Z2856434857	bmse011707	InChI=1S/C11H15NOS/c13-11-3-1-2-10(8-11)9-12-4-6-14-7-5-12/h1-3,8,13H,4-7,9H2

Suppl Table 2: Details of the 768 compounds from the DSI-Poised fragment Library (DSI-PL)

Sl. No.	Structures	No. in Manuscript	CAS Registry Number	"ID"	^{1}H NMR Data Repository BMRB ID	ALATIS InChI
**736**	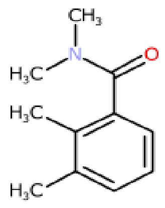	n.a.	90525-99-8	Z32014663	bmse011593	InChI=1S/C11H15NO/c1-8-6-5-7-10(9(8)2)11(13)12(3)4/h5-7H,1-4H3
**737**	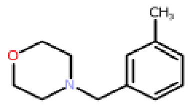	n.a.	90754-64-6	Z2856434862	bmse011571	InChI=1S/C12H17NO/c1-11-3-2-4-12(9-11)10-13-5-7-14-8-6-13/h2-4,9H,5-8,10H2,1H3
**738**	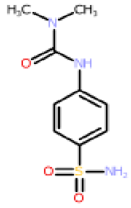	n.a.	91328-92-6	Z123856654	bmse011027	InChI=1S/C9H13N3O3S/c1-12(2)9(13)11-7-3-5-8(6-4-7)16(10,14)15/h3-6H,1-2H3,(H,11,13)(H2,10,14,15)
**739**	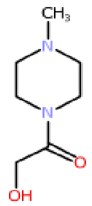	n.a.	91406-27-8	Z228585842	bmse011280	InChI=1S/C7H14N2O2/c1-8-2-4-9(5-3-8)7(11)6-10/h10H,2-6H2,1H3
**740**	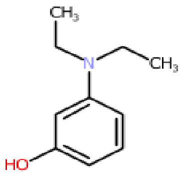	n.a.	91-68-9	Z2856434814	bmse011268	InChI=1S/C10H15NO/c1-3-11(4-2)9-6-5-7-10(12)8-9/h5-8,12H,3-4H2,1-2H3

Suppl Table 2: Details of the 768 compounds from the DSI-Poised fragment Library (DSI-PL)

Sl. No.	Structures	No. in Manuscript	CAS Registry Number	"ID"	^{1}H NMR Data Repository BMRB ID	ALATIS InChI
**741**	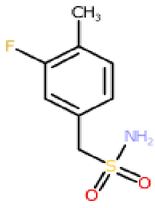	n.a.	919354-68-0	Z1703168683	bmse011098	InChI=1S/C8H10FNO2S/c1-6-2-3-7(4-8(6)9)5-13(10,11)12/h2-4H,5H2,1H3,(H2,10,11,12)
**742**	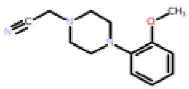	n.a.	92043-13-5	Z2856434807	bmse011314	InChI=1S/C13H17N3O/c1-17-13-5-3-2-4-12(13)16-10-8-15(7-6-14)9-11-16/h2-5H,7-11H2,1H3
**743**	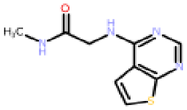	n.a.	923187-09-1	Z52584368	bmse011105	InChI=1S/C9H10N4OS/c1-10-7(14)4-11-8-6-2-3-15-9(6)13-5-12-8/h2-3,5H,4H2,1H3,(H,10,14)(H,11,12,13)
**744**	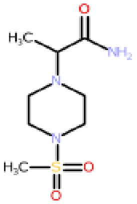	n.a.	923681-46-3	Z134785326	bmse011209	InChI=1S/C8H17N3O3S/c1-7(8(9)12)10-3-5-11(6-4-10)15(2,13)14/h7H,3-6H2,1-2H3,(H2,9,12)/t7-/m1/s1
**745**	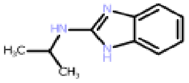	n.a.	924872-04-8	Z2467208649	bmse011588	InChI=1S/C10H13N3/c1-7(2)11-10-12-8-5-3-4-6-9(8)13-10/h3-7H,1-2H3,(H2,11,12,13)

Suppl Table 2: Details of the 768 compounds from the DSI-Poised fragment Library (DSI-PL)

Sl. No.	Structures	No. in Manuscript	CAS Registry Number	"ID"	^{1}H NMR Data Repository BMRB ID	ALATIS InChI
**746**	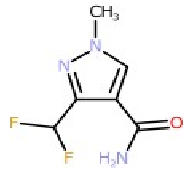	n.a.	925689-10-7	Z1515654336	bmse011424	InChI=1S/C6H7F2N3O/c1-11-2-3(6(9)12)4(10-11)5(7)8/h2,5H,1H3,(H2,9,12)
**747**	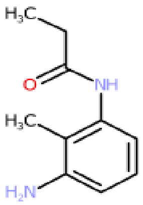	n.a.	926235-23-6	Z235361315	bmse011459	InChI=1S/C10H14N2O/c1-3-10(13)12-9-6-4-5-8(11)7(9)2/h4-6H,3,11H2,1-2H3,(H,12,13)
**748**	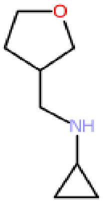	n.a.	926239-80-7	Z818727262	bmse011600	InChI=1S/C8H15NO/c1-2-8(1)9-5-7-3-4-10-6-7/h7-9H,1-6H2/t7-/m1/s1
**749**	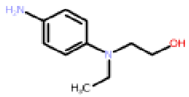	n.a.	92-65-9	Z752989138	bmse011495	InChI=1S/C10H16N2O/c1-2-12(7-8-13)10-5-3-9(11)4-6-10/h3-6,13H,2,7-8,11H2,1H3
**750**	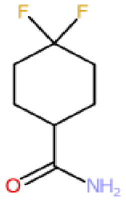	n.a.	927209-98-1	Z1526748660	bmse011153	InChI=1S/C7H11F2NO/c8-7(9)3-1-5(2-4-7)6(10)11/h5H,1-4H2,(H2,10,11)

Suppl Table 2: Details of the 768 compounds from the DSI-Poised fragment Library (DSI-PL)

Sl. No.	Structures	No. in Manuscript	CAS Registry Number	"ID"	^{1}H NMR Data Repository BMRB ID	ALATIS InChI
**751**	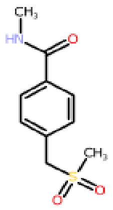	n.a.	930939-62-1	Z30272045	bmse011049	InChI=1S/C10H13NO3S/c1-11-10(12)9-5-3-8(4-6-9)7-15(2,13)14/h3-6H,7H2,1-2H3,(H,11,12)
**752**	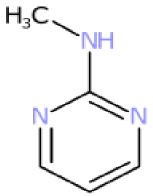	n.a.	931-61-3	Z54628578	bmse011193	InChI=1S/C5H7N3/c1-6-5-7-3-2-4-8-5/h2-4H,1H3,(H,6,7,8)
**753**	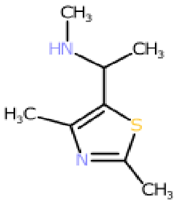	n.a.	933718-01-5	Z1266823232	bmse011757	InChI=1S/C8H14N2S/c1-5(9-4)8-6(2)10-7(3)11-8/h5,9H,1-4H3/t5-/m0/s1
**754**	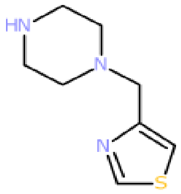	n.a.	933737-25-8	Z812517112	bmse011466	InChI=1S/C8H13N3S/c1-3-11(4-2-9-1)5-8-6-12-7-10-8/h6-7,9H,1-5H2
**755**	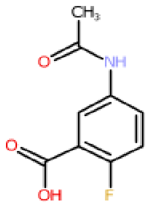	n.a.	939909-22-5	Z364321922	bmse011262	InChI=1S/C9H8FNO3/c1-5(12)11-6-2-3-8(10)7(4-6)9(13)14/h2-4H,1H3,(H,11,12)(H,13,14)

Suppl Table 2: Details of the 768 compounds from the DSI-Poised fragment Library (DSI-PL)

Sl. No.	Structures	No. in Manuscript	CAS Registry Number	"ID"	^{1}H NMR Data Repository BMRB ID	ALATIS InChI
**756**	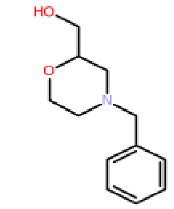	n.a.	943442-96-4	Z1259086950	bmse011736	InChI=1S/C12H17NO2/c14-10-12-9-13(6-7-15-12)8-11-4-2-1-3-5-11/h1-5,12,14H,6-10H2/t12-/m0/s1
**757**	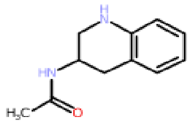	n.a.	945-80-2	Z1492796719	bmse011407	InChI=1S/C11H14N2O/c1-8(14)13-10-6-9-4-2-3-5-11(9)12-7-10/h2-5,10,12H,6-7H2,1H3,(H,13,14)/t10-/m0/s1
**758**	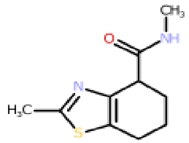	n.a.	95203-31-9	Z1904249627	bmse011121	InChI=1S/C10H14N2OS/c1-6-12-9-7(10(13)11-2)4-3-5-8(9)14-6/h7H,3-5H2,1-2H3,(H,11,13)/t7-/m1/s1
**759**	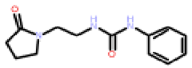	n.a.	95356-60-8	Z2472938267	bmse011184	InChI=1S/C13H17N3O2/c17-12-7-4-9-16(12)10-8-14-13(18)15-11-5-2-1-3-6-11/h1-3,5-6H,4,7-10H2,(H2,14,15,18)
**760**	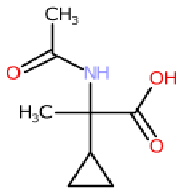	n.a.	95525-82-9	Z1259162160	bmse011264	InChI=1S/C8H13NO3/c1-5(10)9-8(2,7(11)12)6-3-4-6/h6H,3-4H2,1-2H3,(H,9,10)(H,11,12)/t8-/m1/s1

Suppl Table 2: Details of the 768 compounds from the DSI-Poised fragment Library (DSI-PL)

Sl. No.	Structures	No. in Manuscript	CAS Registry Number	"ID"	^{1}H NMR Data Repository BMRB ID	ALATIS InChI
**761**	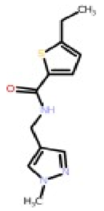	n.a.	955901-04-9	Z804566442	bmse011627	InChI=1S/C12H15N3OS/c1-3-10-4-5-11(17-10)12(16)13-6-9-7-14-15(2)8-9/h4-5,7-8H,3,6H2,1-2H3,(H,13,16)
**762**	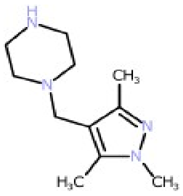	n.a.	957514-00-0	Z1263820300	bmse011279	InChI=1S/C11H20N4/c1-9-11(10(2)14(3)13-9)8-15-6-4-12-5-7-15/h12H,4-8H2,1-3H3
**763**	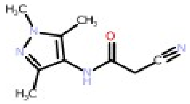	n.a.	957942-27-7	Z1688504114	bmse011676	InChI=1S/C9H12N4O/c1-6-9(7(2)13(3)12-6)11-8(14)4-5-10/h4H2,1-3H3,(H,11,14)
**764**	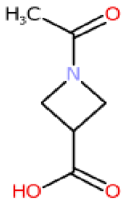	n.a.	97628-91-6	Z1868430535	bmse011467	InChI=1S/C6H9NO3/c1-4(8)7-2-5(3-7)6(9)10/h5H,2-3H2,1H3,(H,9,10)
**765**	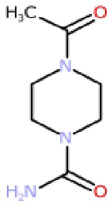	n.a.	98337-79-2	Z90122368	bmse011021	InChI=1S/C7H13N3O2/c1-6(11)9-2-4-10(5-3-9)7(8)12/h2-5H2,1H3,(H2,8,12)

Suppl Table 2: Details of the 768 compounds from the DSI-Poised fragment Library (DSI-PL)

Sl. No.	Structures	No. in Manuscript	CAS Registry Number	"ID"	^{1}H NMR Data Repository BMRB ID	ALATIS InChI
**766**	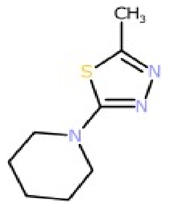	n.a.	98427-81-7	Z1251207602	bmse011513	InChI=1S/C8H13N3S/c1-7-9-10-8(12-7)11-5-3-2-4-6-11/h2-6H2,1H3
**767**	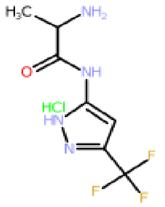	n.a.	n.a.	Z1622626423	bmse011103	InChI=1S/C7H9F3N4O.ClH/c1-3(11)6(15)12-5-2-4(13-14-5)7(8,9)10;/h2-3H,11H2,1H3,(H2,12,13,14,15);1H/t3-;/m0./s1
**768**	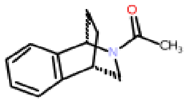	n.a.	n.a.	Z2017861827	bmse011133	InChI=1S/C13H15NO/c1-9(15)14-8-10-6-7-13(14)12-5-3-2-4-11(10)12/h2-5,10,13H,6-8H2,1H3/t10-,13-/m1/s1
