# Supplementary material for: Exploring the Druggability of Conserved RNA Regulatory Elements in the SARS‐CoV‐2 Genome
Source: Angew Chem Int Ed Engl. 2021 Aug 3;60(35):19191–200. doi: 10.1002/anie.202103693 (PMC8426693; doi:10.1002/anie.202103693)
Supplement: Supplementary file 3 — Supporting Information [file ANIE-60-19191-s001.pdf]

Suppl Table 2: Details of the 768 compounds from the DSI-Poised fragment Library (DSI-PL)

Sl. No.	Structures	No. in Manuscript	CAS Registry Number	"ID"	^{1}H NMR Data Repository BMRB ID	ALATIS InChI
**1**	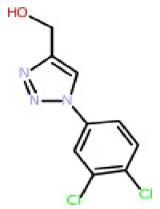	1	338419-11-7	Z1891776064	bmse011464	InChI=1S/C9H7Cl2N3O/c10-8-2-1-7(3-9(8)11)14-4-6(5-15)12-13-14/h1-4,15H,5H2
**2**	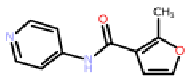	2	35498-44-3	Z136583524	bmse011087	InChI=1S/C11H10N2O2/c1-8-10(4-7-15-8)11(14)13-9-2-5-12-6-3-9/h2-7H,1H3,(H,12,13,14)
**3**	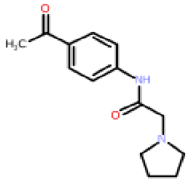	3	406930-20-9	Z2856434916	bmse011371	InChI=1S/C14H18N2O2/c1-11(17)12-4-6-13(7-5-12)15-14(18)10-16-8-2-3-9-16/h4-7H,2-3,8-10H2,1H3,(H,15,18)
**4**	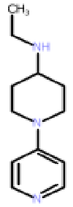	4	1094514-08-5	Z2065616520	bmse011605	InChI=1S/C12H19N3/c1-2-14-11-5-9-15(10-6-11)12-3-7-13-8-4-12/h3-4,7-8,11,14H,2,5-6,9-10H2,1H3
**5**	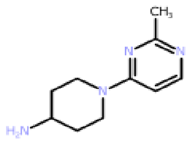	5	1329748-51-7	Z1333043417	bmse011525	InChI=1S/C10H16N4/c1-8-12-5-2-10(13-8)14-6-3-9(11)4-7-14/h2,5,9H,3-4,6-7,11H2,1H3

Suppl Table 2: Details of the 768 compounds from the DSI-Poised fragment Library (DSI-PL)

Sl. No.	Structures	No. in Manuscript	CAS Registry Number	"ID"	^{1}H NMR Data Repository BMRB ID	ALATIS InChI
**6**	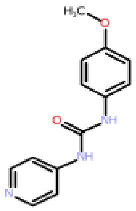	6	20949-48-8	Z321318226	bmse011713	InChI=1S/C13H13N3O2/c1-18-12-4-2-10(3-5-12)15-13(17)16-11-6-8-14-9-7-11/h2-9H,1H3,(H2,14,15,16,17)
**7**	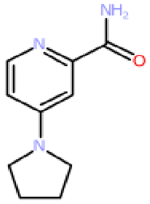	7	1001041-22-0	Z145119960	bmse011137	InChI=1S/C10H13N3O/c11-10(14)9-7-8(3-4-12-9)13-5-1-2-6-13/h3-4,7H,1-2,5-6H2,(H2,11,14)
**8**	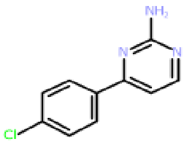	8	133256-51-6	Z363993198	bmse011744	InChI=1S/C10H8ClN3/c11-8-3-1-7(2-4-8)9-5-6-13-10(12)14-9/h1-6H,(H2,12,13,14)
**9**	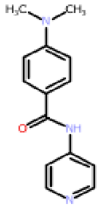	9	113204-31-2	Z85517292	bmse011180	InChI=1S/C14H15N3O/c1-17(2)13-5-3-11(4-6-13)14(18)16-12-7-9-15-10-8-12/h3-10H,1-2H3,(H,15,16,18)
**10**	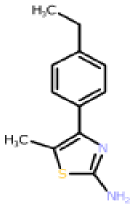	10	438218-98-5	Z48847639	bmse011448	InChI=1S/C12H14N2S/c1-3-9-4-6-10(7-5-9)11-8(2)15-12(13)14-11/h4-7H,3H2,1-2H3,(H2,13,14)

Suppl Table 2: Details of the 768 compounds from the DSI-Poised fragment Library (DSI-PL)

Sl. No.	Structures	No. in Manuscript	CAS Registry Number	"ID"	^{1}H NMR Data Repository BMRB ID	ALATIS InChI
**11**	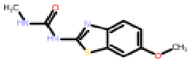	11	28956-38-9	Z371866204	bmse011187	InChI=1S/C10H11N3O2S/c1-11-9(14)13-10-12-7-4-3-6(15-2)5-8(7)16-10/h3-5H,1-2H3,(H2,11,12,13,14)
**12**	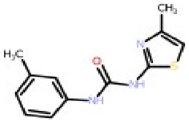	12	663217-65-0	Z44602971	bmse011185	InChI=1S/C12H13N3OS/c1-8-4-3-5-10(6-8)14-11(16)15-12-13-9(2)7-17-12/h3-7H,1-2H3,(H2,13,14,15,16)
**13**	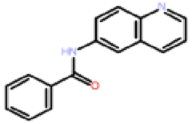	13	219645-89-3	Z453319206	bmse011146	InChI=1S/C16H12N2O/c19-16(12-5-2-1-3-6-12)18-14-8-9-15-13(11-14)7-4-10-17-15/h1-11H,(H,18,19)
**14**	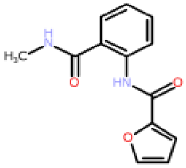	14	84141-29-7	Z30272547	bmse011189	InChI=1S/C13H12N2O3/c1-14-12(16)9-5-2-3-6-10(9)15-13(17)11-7-4-8-18-11/h2-8H,1H3,(H,14,16)(H,15,17)
**15**	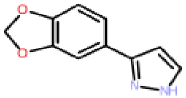	15	141791-06-2	Z2204875953	bmse011337	InChI=1S/C10H8N2O2/c1-2-9-10(14-6-13-9)5-7(1)8-3-4-11-12-8/h1-5H,6H2,(H,11,12)

Suppl Table 2: Details of the 768 compounds from the DSI-Poised fragment Library (DSI-PL)

Sl. No.	Structures	No. in Manuscript	CAS Registry Number	"ID"	^{1}H NMR Data Repository BMRB ID	ALATIS InChI
**16**	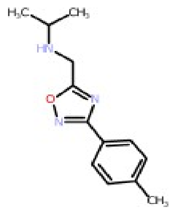	16	708284-23-5	Z2856434849	bmse011650	InChI=1S/C13H17N3O/c1-9(2)14-8-12-15-13(16-17-12)11-6-4-10(3)5-7-11/h4-7,9,14H,8H2,1-3H3
**17**	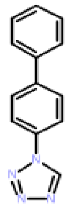	17	63472-38-8	Z57479285	bmse011344	nChI=1S/C13H10N4/c1-2-4-11(5-3-1)12-6-8-13(9-7-12)17-10-14-15-16-17/h1-10
**18**	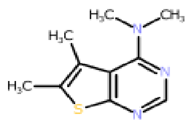	18	690684-98-1	Z31222641	bmse011491	InChI=1S/C10H13N3S/c1-6-7(2)14-10-8(6)9(13(3)4)11-5-12-10/h5H,1-4H3
**19**	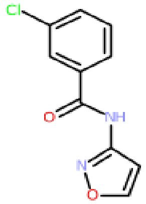	19	777880-26-9	Z86622311	bmse011719	InChI=1S/C10H7ClN2O2/c11-8-3-1-2-7(6-8)10(14)12-9-4-5-15-13-9/h1-6H,(H,12,13,14)
**20**	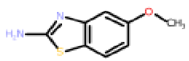	20	54346-87-1	Z1954800564	bmse011353	InChI=1S/C8H8N2OS/c1-11-5-2-3-7-6(4-5)10-8(9)12-7/h2-4H,1H3,(H2,9,10)

Suppl Table 2: Details of the 768 compounds from the DSI-Poised fragment Library (DSI-PL)

Sl. No.	Structures	No. in Manuscript	CAS Registry Number	"ID"	^{1}H NMR Data Repository BMRB ID	ALATIS InChI
**21**	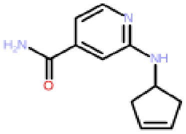	21	1445660-57-0	Z1416571195	bmse011416	InChI=1S/C11H13N3O/c12-11(15)8-5-6-13-10(7-8)14-9-3-1-2-4-9/h1-2,5-7,9H,3-4H2,(H2,12,15)(H,13,14)
**22**	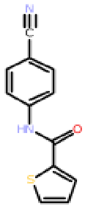	22	137272-71-0	Z30612387	bmse011033	InChI=1S/C12H8N2OS/c13-8-9-3-5-10(6-4-9)14-12(15)11-2-1-7-16-11/h1-7H,(H,14,15)
**23**	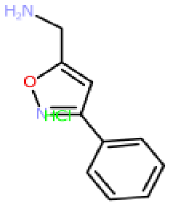	23	13608-55-4	Z425449682	bmse011093	InChI=1S/C10H10N2O.ClH/c11-7-9-6-10(12-13-9)8-4-2-1-3-5-8;/h1-6H,7,11H2;1H
**24**	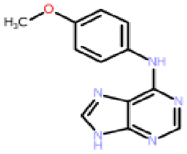	24	6296-91-9	Z276545932	bmse011059	InChI=1S/C12H11N5O/c1-18-9-4-2-8(3-5-9)17-12-10-11(14-6-13-10)15-7-16-12/h2-7H,1H3,(H2,13,14,15,16,17)
**25**	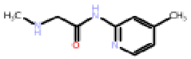	25	1019383-78-8	Z927746322	bmse011463	InChI=1S/C9H13N3O/c1-7-3-4-11-8(5-7)12-9(13)6-10-2/h3-5,10H,6H2,1-2H3,(H,11,12,13)

Suppl Table 2: Details of the 768 compounds from the DSI-Poised fragment Library (DSI-PL)

Sl. No.	Structures	No. in Manuscript	CAS Registry Number	"ID"	1H NMR Data Repository BMRB ID	ALATIS InChI
**26**	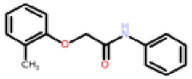	26	22560-44-7	Z19733979	bmse011328	InChI=1S/C15H15NO2/c1-12-7-5-6-10-14(12)18-11-15(17)16-13-8-3-2-4-9-13/h2-10H,11H2,1H3,(H,16,17)
**27**	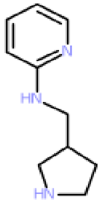	27	1251925-43-5	Z1262327505	bmse011522	InChI=1S/C10H15N3/c1-2-5-12-10(3-1)13-8-9-4-6-11-7-9/h1-3,5,9,11H,4,6-8H2,(H,12,13)/t9-/m0/s1
**28**	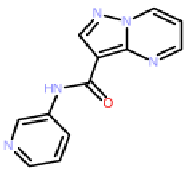	28	717831-80-6	Z296054478	bmse011360	InChI=1S/C12H9N5O/c18-12(16-9-3-1-4-13-7-9)10-8-15-17-6-2-5-14-11(10)17/h1-8H,(H,16,18)
**29**	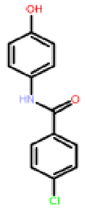	29	19207-92-2	Z56346825	bmse011001	InChI=1S/C13H10ClNO2/c14-10-3-1-9(2-4-10)13(17)15-11-5-7-12(16)8-6-11/h1-8,16H,(H,15,17)
**30**	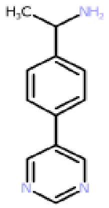	30	1250290-30-2	Z1696822287	bmse011426	InChI=1S/C12H13N3/c1-9(13)10-2-4-11(5-3-10)12-6-14-8-15-7-12/h2-9H,13H2,1H3/t9-/m0/s1

Suppl Table 2: Details of the 768 compounds from the DSI-Poised fragment Library (DSI-PL)

Sl. No.	Structures	No. in Manuscript	CAS Registry Number	"ID"	^{1}H NMR Data Repository BMRB ID	ALATIS InChI
**31**	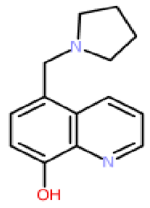	31	41455-82-7	Z2856434902	bmse011447	InChI=1S/C14H16N2O/c17-13-6-5-11(10-16-8-1-2-9-16)12-4-3-7-15-14(12)13/h3-7,17H,1-2,8-10H2
**32**	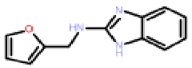	32	727392-72-5	Z57299526	bmse011727	InChI=1S/C12H11N3O/c1-2-6-11-10(5-1)14-12(15-11)13-8-9-4-3-7-16-9/h1-7H,8H2,(H2,13,14,15)
**33**	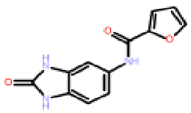	33	339207-13-5	Z26794338	bmse011698	InChI=1S/C12H9N3O3/c16-11(10-2-1-5-18-10)13-7-3-4-8-9(6-7)15-12(17)14-8/h1-6H,(H,13,16)(H2,14,15,17)
**34**	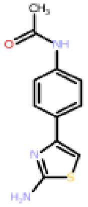	34	21674-96-4	Z48847594	bmse011438	InChI=1S/C11H11N3OS/c1-7(15)13-9-4-2-8(3-5-9)10-6-16-11(12)14-10/h2-6H,1H3,(H2,12,14)(H,13,15)
**35**	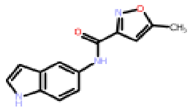	35	708987-60-4	Z1190787729	bmse011725	InChI=1S/C13H11N3O2/c1-8-6-12(16-18-8)13(17)15-10-2-3-11-9(7-10)4-5-14-11/h2-7,14H,1H3,(H,15,17)

Suppl Table 2: Details of the 768 compounds from the DSI-Poised fragment Library (DSI-PL)

Sl. No.	Structures	No. in Manuscript	CAS Registry Number	"ID"	^{1}H NMR Data Repository BMRB ID	ALATIS InChI
**36**	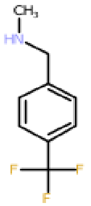	36	90390-11-7	Z2856434809	bmse011474	InChI=1S/C9H10F3N/c1-13-6-7-2-4-8(5-3-7)9(10,11)12/h2-5,13H,6H2,1H3
**37**	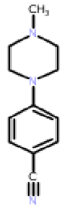	37	34334-28-6	Z2856434840	bmse011037	InChI=1S/C12H15N3/c1-14-6-8-15(9-7-14)12-4-2-11(10-13)3-5-12/h2-5H,6-9H2,1H3
**38**	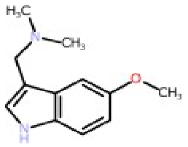	38	16620-52-3	Z2856434938	bmse011469	InChI=1S/C12H16N2O/c1-14(2)8-9-7-13-12-5-4-10(15-3)6-11(9)12/h4-7,13H,8H2,1-3H3
**39**	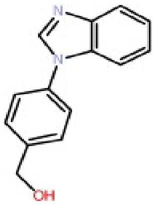	39	451485-67-9	Z235449082	bmse011062	InChI=1S/C14H12N2O/c17-9-11-5-7-12(8-6-11)16-10-15-13-3-1-2-4-14(13)16/h1-8,10,17H,9H2
**40**	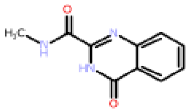	40	53115-17-6	Z332370018	bmse011072	InChI=1S/C10H9N3O2/c1-11-10(15)8-12-7-5-3-2-4-6(7)9(14)13-8/h2-5H,1H3,(H,11,15)(H,12,13,14)

Suppl Table 2: Details of the 768 compounds from the DSI-Poised fragment Library (DSI-PL)

Sl. No.	Structures	No. in Manuscript	CAS Registry Number	"ID"	^{1}H NMR Data Repository BMRB ID	ALATIS InChI
**41**	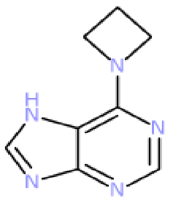	41	1379186-26-1	Z373221060	bmse011127	InChI=1S/C8H9N5/c1-2-13(3-1)8-6-7(10-4-9-6)11-5-12-8/h4-5H,1-3H2,(H,9,10,11,12)
**42**	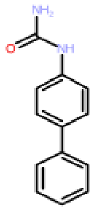	42	13262-48-1	Z198195774	bmse011251	InChI=1S/C13H12N2O/c14-13(16)15-12-8-6-11(7-9-12)10-4-2-1-3-5-10/h1-9H,(H3,14,15,16)
**43**	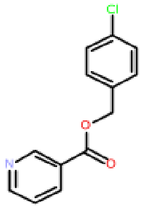	43	100398-39-8	Z57515803	bmse011305	InChI=1S/C13H10ClNO2/c14-12-5-3-10(4-6-12)9-17-13(16)11-2-1-7-15-8-11/h1-8H,9H2
**44**	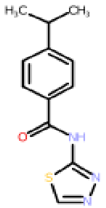	44	544690-34-8	Z86416893	bmse011331	InChI=1S/C12H13N3OS/c1-8(2)9-3-5-10(6-4-9)11(16)14-12-15-13-7-17-12/h3-8H,1-2H3,(H,14,15,16)
**45**	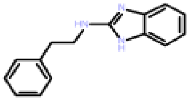	45	727392-70-3	Z57299529	bmse011629	InChI=1S/C15H15N3/c1-2-6-12(7-3-1)10-11-16-15-17-13-8-4-5-9-14(13)18-15/h1-9H,10-11H2,(H2,16,17,18)

Suppl Table 2: Details of the 768 compounds from the DSI-Poised fragment Library (DSI-PL)

Sl. No.	Structures	No. in Manuscript	CAS Registry Number	"ID"	^{1}H NMR Data Repository BMRB ID	ALATIS InChI
**46**	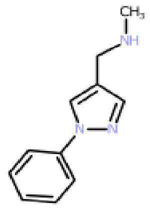	46	868552-05-0	Z102768020	bmse011449	InChI=1S/C11H13N3/c1-12-7-10-8-13-14(9-10)11-5-3-2-4-6-11/h2-6,8-9,12H,7H2,1H3
**47**	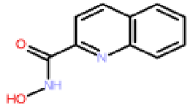	47	37137-42-1	Z59181945	bmse011401	InChI=1S/C10H8N2O2/c13-10(12-14)9-6-5-7-3-1-2-4-8(7)11-9/h1-6,14H,(H,12,13)
**48**	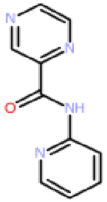	48	484039-13-6	Z85525386	bmse011215	InChI=1S/C10H8N4O/c15-10(8-7-11-5-6-12-8)14-9-3-1-2-4-13-9/h1-7H,(H,13,14,15)
**49**	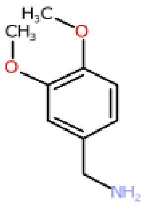	49	5763-61-1	Z2856434917	bmse011473	InChI=1S/C9H13NO2/c1-11-8-4-3-7(6-10)5-9(8)12-2/h3-5H,6,10H2,1-2H3
**50**	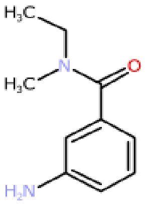	50	1094911-27-9	Z2092370954	bmse011131	InChI=1S/C10H14N2O/c1-3-12(2)10(13)8-5-4-6-9(11)7-8/h4-7H,3,11H2,1-2H3

Suppl Table 2: Details of the 768 compounds from the DSI-Poised fragment Library (DSI-PL)

Sl. No.	Structures	No. in Manuscript	CAS Registry Number	"ID"	^{1}H NMR Data Repository BMRB ID	ALATIS InChI
**51**	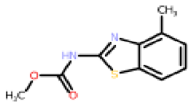	51	717868-63-8	Z192981502	bmse011679	InChI=1S/C10H10N2O2S/c1-6-4-3-5-7-8(6)11-9(15-7)12-10(13)14-2/h3-5H,1-2H3,(H,11,12,13)
**52**	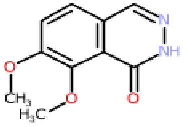	52	4821-89-0	Z102895082	bmse011050	InChI=1S/C10H10N2O3/c1-14-7-4-3-6-5-11-12-10(13)8(6)9(7)15-2/h3-5H,1-2H3,(H,12,13)
**53**	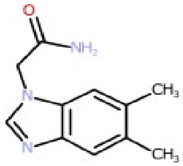	53	953847-76-2	Z26251905	bmse011083	InChI=1S/C11H13N3O/c1-7-3-9-10(4-8(7)2)14(6-13-9)5-11(12)15/h3-4,6H,5H2,1-2H3,(H2,12,15)
**54**	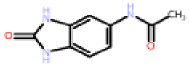	54	91085-68-6	Z26794305	bmse011177	InChI=1S/C9H9N3O2/c1-5(13)10-6-2-3-7-8(4-6)12-9(14)11-7/h2-4H,1H3,(H,10,13)(H2,11,12,14)
**55**	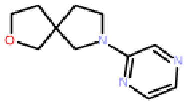	55	1607251-73-9	Z1401276297	bmse011101	InChI=1S/C11H15N3O/c1-5-14(10-7-12-3-4-13-10)8-11(1)2-6-15-9-11/h3-4,7H,1-2,5-6,8-9H2/t11-/m0/s1

Suppl Table 2: Details of the 768 compounds from the DSI-Poised fragment Library (DSI-PL)

Sl. No.	Structures	No. in Manuscript	CAS Registry Number	"ID"	^{1}H NMR Data Repository BMRB ID	ALATIS InChI
**56**	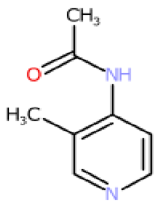	56	104915-66-4	Z1148747945	bmse011159	InChI=1S/C8H10N2O/c1-6-5-9-4-3-8(6)10-7(2)11/h3-5H,1-2H3,(H,9,10,11)
**57**	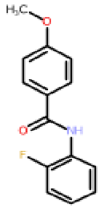	57	143925-52-4	Z28290321	bmse011232	InChI=1S/C14H12FNO2/c1-18-11-8-6-10(7-9-11)14(17)16-13-5-3-2-4-12(13)15/h2-9H,1H3,(H,16,17)
**58**	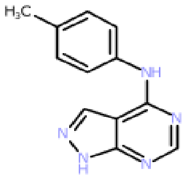	58	369396-26-9	Z57101343	bmse011290	InChI=1S/C12H11N5/c1-8-2-4-9(5-3-8)16-11-10-6-15-17-12(10)14-7-13-11/h2-7H,1H3,(H2,13,14,15,16,17)
**59**	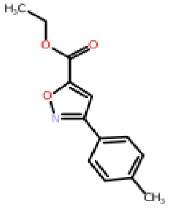	59	377062-62-9	Z793500562	bmse011326	InChI=1S/C13H13NO3/c1-3-16-13(15)12-8-11(14-17-12)10-6-4-9(2)5-7-10/h4-8H,3H2,1-2H3
**60**	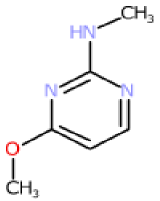	60	66131-71-3	Z281802060	bmse011406	InChI=1S/C6H9N3O/c1-7-6-8-4-3-5(9-6)10-2/h3-4H,1-2H3,(H,7,8,9)

Suppl Table 2: Details of the 768 compounds from the DSI-Poised fragment Library (DSI-PL)

Sl. No.	Structures	No. in Manuscript	CAS Registry Number	"ID"	^{1}H NMR Data Repository BMRB ID	ALATIS InChI
**61**	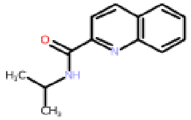	61	586982-18-5	Z31385861	bmse011586	InChI=1S/C13H14N2O/c1-9(2)14-13(16)12-8-7-10-5-3-4-6-11(10)15-12/h3-9H,1-2H3,(H,14,16)
**62**	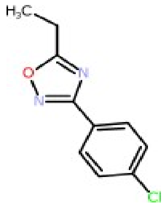	62	196301-93-6	Z57792053	bmse011692	InChI=1S/C10H9ClN2O/c1-2-9-12-10(13-14-9)7-3-5-8(11)6-4-7/h3-6H,2H2,1H3
**63**	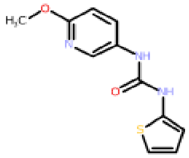	63	719269-39-3	Z1152242726	bmse011711	InChI=1S/C11H11N3O2S/c1-16-9-5-4-8(7-12-9)13-11(15)14-10-3-2-6-17-10/h2-7H,1H3,(H2,13,14,15)
**64**	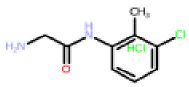	64	1052545-13-7	Z85956652	bmse011008	InChI=1S/C9H11ClN2O.ClH/c1-6-7(10)3-2-4-8(6)12-9(13)5-11;/h2-4H,5,11H2,1H3,(H,12,13);1H
**65**	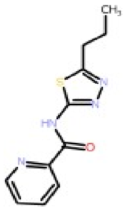	65	691382-96-4	Z52425517	bmse011130	InChI=1S/C11H12N4OS/c1-2-5-9-14-15-11(17-9)13-10(16)8-6-3-4-7-12-8/h3-4,6-7H,2,5H2,1H3,(H,13,15,16)

Suppl Table 2: Details of the 768 compounds from the DSI-Poised fragment Library (DSI-PL)

Sl. No.	Structures	No. in Manuscript	CAS Registry Number	"ID"	^{1}H NMR Data Repository BMRB ID	ALATIS InChI
**66**	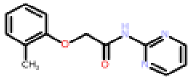	66	723737-15-3	Z53834613	bmse011182	InChI=1S/C13H13N3O2/c1-10-5-2-3-6-11(10)18-9-12(17)16-13-14-7-4-8-15-13/h2-8H,9H2,1H3,(H,14,15,16,17)
**67**	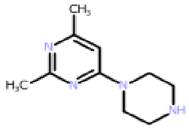	67	887686-65-9	Z274575916	bmse011270	InChI=1S/C10H16N4/c1-8-7-10(13-9(2)12-8)14-5-3-11-4-6-14/h7,11H,3-6H2,1-2H3
**68**	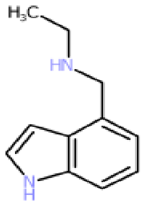	68	342412-30-0	Z1137725943	bmse011556	InChI=1S/C11H14N2/c1-2-12-8-9-4-3-5-11-10(9)6-7-13-11/h3-7,12-13H,2,8H2,1H3
**69**	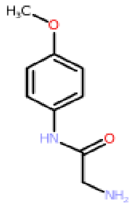	69	148627-63-8	Z1449748885	bmse011624	InChI=1S/C9H12N2O2/c1-13-8-4-2-7(3-5-8)11-9(12)6-10/h2-5H,6,10H2,1H3,(H,11,12)
**70**	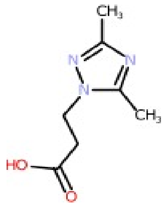	n.a.	842971-05-5	Z274553586	bmse011095	InChI=1S/C7H11N3O2/c1-5-8-6(2)10(9-5)4-3-7(11)12/h3-4H2,1-2H3,(H,11,12)

Suppl Table 2: Details of the 768 compounds from the DSI-Poised fragment Library (DSI-PL)

Sl. No.	Structures	No. in Manuscript	CAS Registry Number	"ID"	^{1}H NMR Data Repository BMRB ID	ALATIS InChI
**71**	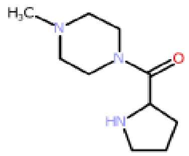	n.a.	100158-68-7	Z241832786	bmse011528	InChI=1S/C10H19N3O/c1-12-5-7-13(8-6-12)10(14)9-3-2-4-11-9/h9,11H,2-8H2,1H3/t9-/m1/s1
**72**	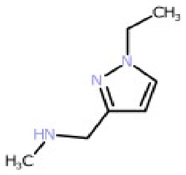	n.a.	1002651-65-1	Z2856434888	bmse011472	InChI=1S/C7H13N3/c1-3-10-5-4-7(9-10)6-8-2/h4-5,8H,3,6H2,1-2H3
**73**	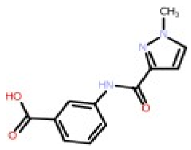	n.a.	1002651-89-9	Z2856434892	bmse011746	InChI=1S/C12H11N3O3/c1-15-6-5-10(14-15)11(16)13-9-4-2-3-8(7-9)12(17)18/h2-7H,1H3,(H,13,16)(H,17,18)
**74**	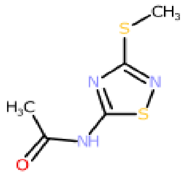	n.a.	100517-04-2	Z26804467	bmse011432	InChI=1S/C5H7N3OS2/c1-3(9)6-4-7-5(10-2)8-11-4/h1-2H3,(H,6,7,8,9)
**75**	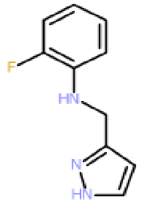	n.a.	1006438-26-1	Z1545196403	bmse011560	InChI=1S/C10H10FN3/c11-9-3-1-2-4-10(9)12-7-8-5-6-13-14-8/h1-6,12H,7H2,(H,13,14)

Suppl Table 2: Details of the 768 compounds from the DSI-Poised fragment Library (DSI-PL)

Sl. No.	Structures	No. in Manuscript	CAS Registry Number	"ID"	^{1}H NMR Data Repository BMRB ID	ALATIS InChI
**76**	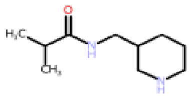	n.a.	1016685-94-1	Z1262327459	bmse011604	InChI=1S/C10H20N2O/c1-8(2)10(13)12-7-9-4-3-5-11-6-9/h8-9,11H,3-7H2,1-2H3,(H,12,13)/t9-/m0/s1
**77**	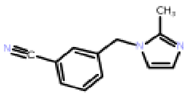	n.a.	1016716-83-8	Z319545618	bmse011254	InChI=1S/C12H11N3/c1-10-14-5-6-15(10)9-12-4-2-3-11(7-12)8-13/h2-7H,9H2,1H3
**78**	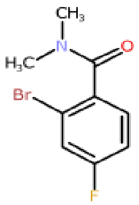	n.a.	1016749-54-4	Z100642432	bmse011257	InChI=1S/C9H9BrFNO/c1-12(2)9(13)7-4-3-6(11)5-8(7)10/h3-5H,1-2H3
**79**	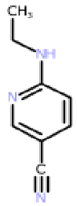	n.a.	1016813-34-5	Z219104216	bmse011084	InChI=1S/C8H9N3/c1-2-10-8-4-3-7(5-9)6-11-8/h3-4,6H,2H2,1H3,(H,10,11)
**80**	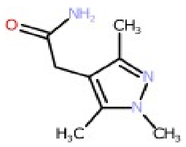	n.a.	1017502-16-7	Z287484230	bmse011244	InChI=1S/C8H13N3O/c1-5-7(4-8(9)12)6(2)11(3)10-5/h4H2,1-3H3,(H2,9,12)

Suppl Table 2: Details of the 768 compounds from the DSI-Poised fragment Library (DSI-PL)

Sl. No.	Structures	No. in Manuscript	CAS Registry Number	"ID"	^{1}H NMR Data Repository BMRB ID	ALATIS InChI
**81**	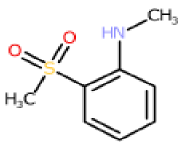	n.a.	10224-69-8	Z285782452	bmse011392	InChI=1S/C8H11NO2S/c1-9-7-5-3-4-6-8(7)12(2,10)11/h3-6,9H,1-2H3
**82**	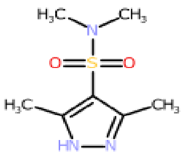	n.a.	1025067-61-1	Z94597856	bmse011194	InChI=1S/C7H13N3O2S/c1-5-7(6(2)9 8-5)13(11,12)10(3)4/h1-4H3,(H,8,9)
**83**	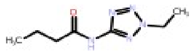	n.a.	1025922-90-0	Z57475877	bmse011310	InChI=1S/C7H13N5O/c1-3-5-6(13)8-7-9-11-12(4-2)10-7/h3-5H2,1-2H3,(H,8,10,13)
**84**	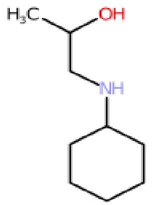	n.a.	103-00-4	Z90526999	bmse011272	InChI=1S/C9H19NO/c1-8(11)7-10-9-5-3-2-4-6-9/h8-11H,2-7H2,1H3/t8-/m0/s1
**85**	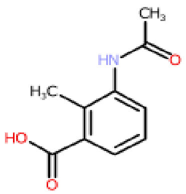	n.a.	103204-68-8	Z364368134	bmse011107	InChI=1S/C10H11NO3/c1-6-8(10(13)14)4-3-5-9(6)11-7(2)12/h3-5H,1-2H3,(H,11,12)(H,13,14)

Suppl Table 2: Details of the 768 compounds from the DSI-Poised fragment Library (DSI-PL)

Sl. No.	Structures	No. in Manuscript	CAS Registry Number	"ID"	^{1}H NMR Data Repository BMRB ID	ALATIS InChI
**86**	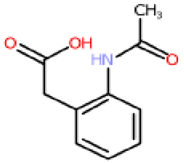	n.a.	103205-34-1	Z1444783243	bmse011411	InChI=1S/C10H11NO3/c1-7(12)11-9-5-3-2-4-8(9)6-10(13)14/h2-5H,6H2,1H3,(H,11,12)(H,13,14)
**87**	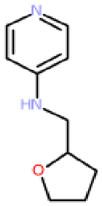	n.a.	1036514-53-0	Z1267882044	bmse011541	InChI=1S/C10H14N2O/c1-2-10(13-7-1)8-12-9-3-5-11-6-4-9/h3-6,10H,1-2,7-8H2,(H,11,12)/t10-/m1/s1
**88**	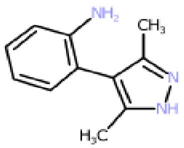	n.a.	103858-98-6	Z1270312110	bmse011386	InChI=1S/C11H13N3/c1-7-11(8(2)14-13-7)9-5-3-4-6-10(9)12/h3-6H,12H2,1-2H3,(H,13,14)
**89**	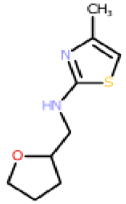	n.a.	1039996-30-9	Z48852953	bmse011223	InChI=1S/C9H14N2OS/c1-7-6-13-9(11-7)10-5-8-3-2-4-12-8/h6,8H,2-5H2,1H3,(H,10,11)/t8-/m1/s1
**90**	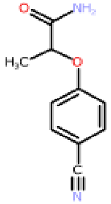	n.a.	1040041-89-1	Z19739650	bmse011221	InChI=1S/C10H10N2O2/c1-7(10(12)13)14-9-4-2-8(6-11)3-5-9/h2-5,7H,1H3,(H2,12,13)/t7-/m0/s1

Suppl Table 2: Details of the 768 compounds from the DSI-Poised fragment Library (DSI-PL)

Sl. No.	Structures	No. in Manuscript	CAS Registry Number	"ID"	1H NMR Data Repository BMRB ID	ALATIS InChI
**91**	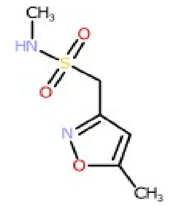	n.a.	1042787-05-2	Z763030030	bmse011201	InChI=1S/C6H10N2O3S/c1-5-3-6(8-11-5)4-12(9,10)7-2/h3,7H,4H2,1-2H3
**92**	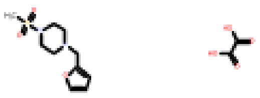	n.a.	1047978-76-6	Z2241115980	bmse011220	InChI=1S/C10H16N2O3S.C2H2O4/c1-16(13,14)12-6-4-11(5-7-12)9-10-3-2-8-15-10;3-1(4)2(5)6/h2-3,8H,4-7,9H2,1H3;(H,3,4)(H,5,6)
**93**	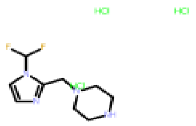	n.a.	1049726-54-6	Z2444672448	bmse011450	InChI=1S/C9H14F2N4.3ClH/c10-9(11)15-6-3-13-8(15)7-14-4-1-12-2-5-14;;;/h3,6,9,12H,1-2,4-5,7H2;3*1H
**94**	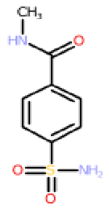	n.a.	10518-85-1	Z165170770	bmse011212	InChI=1S/C8H10N2O3S/c1-10-8(11)6-2-4-7(5-3-6)14(9,12)13/h2-5H,1H3,(H,10,11)(H2,9,12,13)
**95**	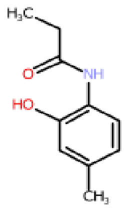	n.a.	105296-19-3	Z33452106	bmse011202	InChI=1S/C10H13NO2/c1-3-10(13)11-8-5-4-7(2)6-9(8)12/h4-6,12H,3H2,1-2H3,(H,11,13)

Suppl Table 2: Details of the 768 compounds from the DSI-Poised fragment Library (DSI-PL)

Sl. No.	Structures	No. in Manuscript	CAS Registry Number	"ID"	^{1}H NMR Data Repository BMRB ID	ALATIS InChI
**96**	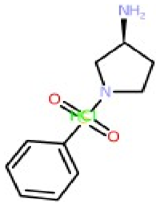	n.a.	1056998-45-8	Z2443429438	bmse011132	InChI=1S/C10H14N2O2S.ClH/c11-9-6-7-12(8-9)15(13,14)10-4-2-1-3-5-10;/h1-5,9H,6-8,11H2;1H/t9-;/m0./s1
**97**	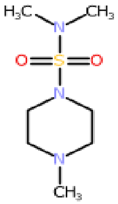	n.a.	105871-27-0	Z2735592898	bmse011704	InChI=1S/C7H17N3O2S/c1-8(2)13(11,12)10-6-4-9(3)5-7-10/h4-7H2,1-3H3
**98**	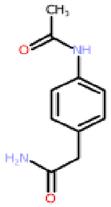	n.a.	1060283-53-5	Z87615031	bmse011281	InChI=1S/C10H12N2O2/c1-7(13)12-9-4-2-8(3-5-9)6-10(11)14/h2-5H,6H2,1H3,(H2,11,14)(H,12,13)
**99**	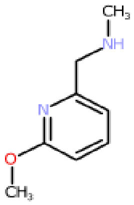	n.a.	1060806-94-1	Z1603775714	bmse011748	InChI=1S/C8H12N2O/c1-9-6-7-4-3-5-8(10-7)11-2/h3-5,9H,6H2,1-2H3
**100**	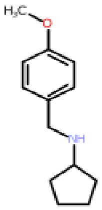	n.a.	108157-23-9	Z2856434940	bmse011715	InChI=1S/C13H19NO/c1-15-13-8-6-11(7-9-13)10-14-12-4-2-3-5-12/h6-9,12,14H,2-5,10H2,1H3

Suppl Table 2: Details of the 768 compounds from the DSI-Poised fragment Library (DSI-PL)

Sl. No.	Structures	No. in Manuscript	CAS Registry Number	"ID"	1H NMR Data Repository BMRB ID	ALATIS InChI
**101**	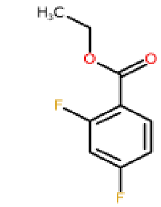	n.a.	108928-00-3	Z53836105	bmse011644	InChI=1S/C9H8F2O2/c1-2-13-9(12)7-4-3-6(10)5-8(7)11/h3-5H,2H2,1H3
**102**	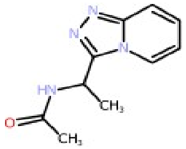	n.a.	1090718-29-8	Z131516158	bmse011239	InChI=1S/C10H12N4O/c1-7(11-8(2)15)10-13-12-9-5-3-4-6-14(9)10/h3-7H,1-2H3,(H,11,15)/t7-/m0/s1
**103**	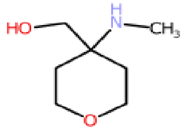	n.a.	1094072-05-5	Z1169060901	bmse011557	InChI=1S/C7H15NO2/c1-8-7(6-9)2-4-10-5-3-7/h8-9H,2-6H2,1H3
**104**	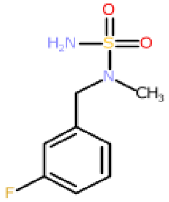	n.a.	1094315-37-3	Z300245038	bmse011222	InChI=1S/C8H11FN2O2S/c1-11(14(10,12)13)6-7-3-2-4-8(9)5-7/h2-5H,6H2,1H3,(H2,10,12,13)
**105**	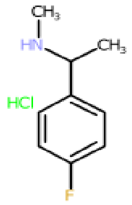	n.a.	1095514-42-3	Z166605480	bmse011451	InChI=1S/C9H12FN.ClH/c1-7(11-2)8-3-5-9(10)6-4-8;/h3-7,11H,1-2H3;1H/t7-;/m0./s1

Suppl Table 2: Details of the 768 compounds from the DSI-Poised fragment Library (DSI-PL)

Sl. No.	Structures	No. in Manuscript	CAS Registry Number	"ID"	^{1}H NMR Data Repository BMRB ID	ALATIS InChI
**106**	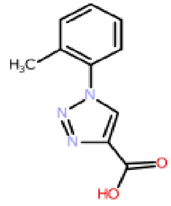	n.a.	1099631-80-7	Z2327226104	bmse011142	InChI=1S/C10H9N3O2/c1-7-4-2-3-5-9(7)13-6-8(10(14)15)11-12-13/h2-6H,1H3,(H,14,15)
**107**	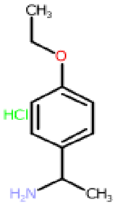	n.a.	1108684-07-6	Z210803634	bmse011126	InChI=1S/C10H15NO.ClH/c1-3-12-10-6-4-9(5-7-10)8(2)11;/h4-8H,3,11H2,1-2H3;1H/t8-;/m0./s1
**108**	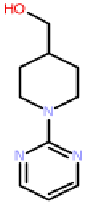	n.a.	111247-61-1	Z645232558	bmse011666	InChI=1S/C10H15N3O/c14-8-9-2-6-13(7-3-9)10-11-4-1-5-12-10/h1,4-5,9,14H,2-3,6-8H2
**109**	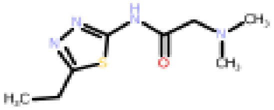	n.a.	111750-46-0	Z2856434846	bmse011363	InChI=1S/C8H14N4OS/c1-4-7-10-11-8(14-7)9-6(13)5-12(2)3/h4-5H2,1-3H3,(H,9,11,13)
**110**	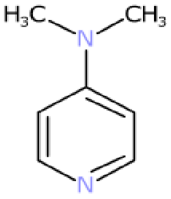	n.a.	1122-58-3	Z2856434762	bmse011259	InChI=1S/C7H10N2/c1-9(2)7-3-5-8-6-4-7/h3-6H,1-2H3

Suppl Table 2: Details of the 768 compounds from the DSI-Poised fragment Library (DSI-PL)

Sl. No.	Structures	No. in Manuscript	CAS Registry Number	"ID"	^{1}H NMR Data Repository BMRB ID	ALATIS InChI
**111**	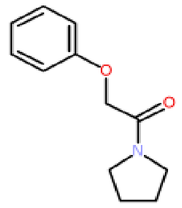	n.a.	112283-41-7	Z19735981	bmse011701	InChI=1S/C12H15NO2/c14-12(13-8-4-5-9-13)10-15-11-6-2-1-3-7-11/h1-3,6-7H,4-5,8-10H2
**112**	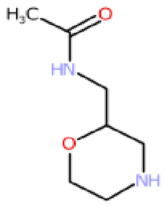	n.a.	112913-95-8	Z1269638430	bmse011461	InChI=1S/C7H14N2O2/c1-6(10)9-5-7-4-8-2-3-11-7/h7-8H,2-5H2,1H3,(H,9,10)/t7-/m0/s1
**113**	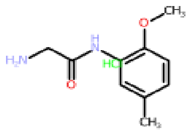	n.a.	1147208-19-2	Z235341991	bmse011152	InChI=1S/C10H14N2O2.ClH/c1-7-3-4-9(14-2)8(5-7)12-10(13)6-11;/h3-5H,6,11H2,1-2H3,(H,12,13);1H
**114**	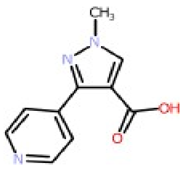	n.a.	1152510-62-7	Z815264062	bmse011206	InChI=1S/C10H9N3O2/c1-13-6-8(10(14)15)9(12-13)7-2-4-11-5-3-7/h2-6H,1H3,(H,14,15)
**115**	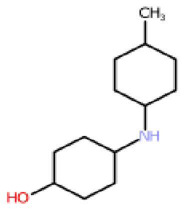	n.a.	1152964-08-3	Z2241963319	bmse011271	InChI=1S/C13H25NO/c1-10-2-4-11(5-3-10)14-12-6-8-13(15)9-7-12/h10-15H,2-9H2,1H3/t10-,11-,12-,13-

Suppl Table 2: Details of the 768 compounds from the DSI-Poised fragment Library (DSI-PL)

Sl. No.	Structures	No. in Manuscript	CAS Registry Number	"ID"	1H NMR Data Repository BMRB ID	ALATIS InChI
**116**	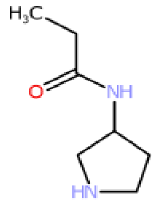	n.a.	1154969-50-2	Z1650040241	bmse011531	InChI=1S/C7H14N2O/c1-2-7(10)9-6-3-4-8-5-6/h6,8H,2-5H2,1H3,(H,9,10)/t6-/m0/s1
**117**	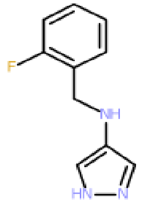	n.a.	1156177-23-9	Z1454310449	bmse011116	InChI=1S/C10H10FN3/c11-10-4-2-1-3-8(10)5-12-9-6-13-14-7-9/h1-4,6-7,12H,5H2,(H,13,14)
**118**	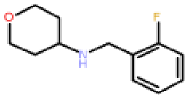	n.a.	1157009-10-3	Z823455846	bmse011737	InChI=1S/C12H16FNO/c13-12-4-2-1-3-10(12)9-14-11-5-7-15-8-6-11/h1-4,11,14H,5-9H2
**119**	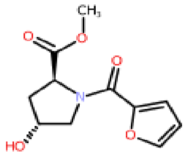	n.a.	1164125-09-0	Z1614545742	bmse011301	InChI=1S/C11H13NO5/c1-16-11(15)8-5-7(13)6-12(8)10(14)9-3-2-4-17-9/h2-4,7-8,13H,5-6H2,1H3/t7-,8+/m1/s1
**120**	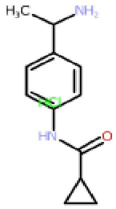	n.a.	1170962-96-5	Z1891773393	bmse011091	InChI=1S/C12H16N2O.ClH/c1-8(13)9-4-6-11(7-5-9)14-12(15)10-2-3-10;/h4-8,10H,2-3,13H2,1H3,(H,14,15);1H/t8-;/m0./s1

Suppl Table 2: Details of the 768 compounds from the DSI-Poised fragment Library (DSI-PL)

Sl. No.	Structures	No. in Manuscript	CAS Registry Number	"ID"	^{1}H NMR Data Repository BMRB ID	ALATIS InChI
121	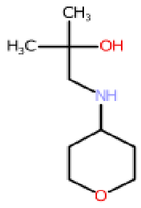	n.a.	1178195-37-3	Z1103351268	bmse011592	InChI=1S/C9H19NO2/c1-9(2,11)7-10-8-3-5-12-6-4-8/h8,10-11H,3-7H2,1-2H3
122	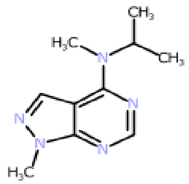	n.a.	117864-83-2	Z328695024	bmse011240	InChI=1S/C10H15N5/c1-7(2)14(3)9-8-5-13-15(4)10(8)12-6-11-9/h5-7H,1-4H3
123	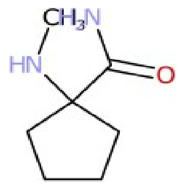	n.a.	1179878-43-3	Z1348559502	bmse011412	InChI=1S/C7H14N2O/c1-9-7(6(8)10)4-2-3-5-7/h9H,2-5H2,1H3,(H2,8,10)
124	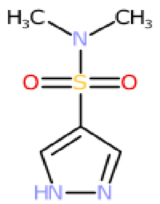	n.a.	1179901-00-8	Z805551440	bmse011106	InChI=1S/C5H9N3O2S/c1-8(2)11(9,10)5-3-6-7-4-5/h3-4H,1-2H3,(H,6,7)
125	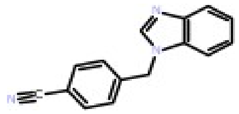	n.a.	118001-91-5	Z26333434	bmse011048	InChI=1S/C15H11N3/c16-9-12-5-7-13(8-6-12)10-18-11-17-14-3-1-2-4-15(14)18/h1-8,11H,10H2

Suppl Table 2: Details of the 768 compounds from the DSI-Poised fragment Library (DSI-PL)

Sl. No.	Structures	No. in Manuscript	CAS Registry Number	"ID"	1H NMR Data Repository BMRB ID	ALATIS InChI
**126**	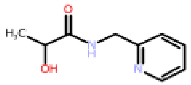	n.a.	118201-19-7	Z1551688335	bmse011120	InChI=1S/C9H12N2O2/c1-7(12)9(13)11-6-8-4-2-3-5-10-8/h2-5,7,12H,6H2,1H3,(H,11,13)/t7-/m0/s1
**127**	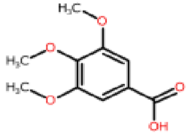	n.a.	118-41-2	Z2734782702	bmse011052	InChI=1S/C10H12O5/c1-13-7-4-6(10(11)12)5-8(14-2)9(7)15-3/h4-5H,1-3H3,(H,11,12)
**128**	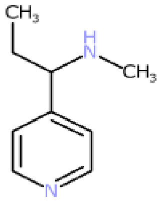	n.a.	1184131-82-5	Z1250132788	bmse011551	InChI=1S/C9H14N2/c1-3-9(10-2)8-4-6-11-7-5-8/h4-7,9-10H,3H2,1-2H3/t9-/m0/s1
**129**	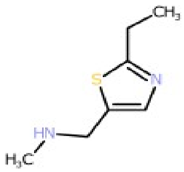	n.a.	1184195-56-9	Z1267773633	bmse011602	InChI=1S/C7H12N2S/c1-3-7-9-5-6(10-7)4-8-2/h5,8H,3-4H2,1-2H3
**130**	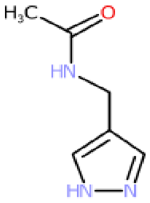	n.a.	1184541-34-1	Z383325512	bmse011164	InChI=1S/C6H9N3O/c1-5(10)7-2-6-3-8-9-4-6/h3-4H,2H2,1H3,(H,7,10)(H,8,9)

Suppl Table 2: Details of the 768 compounds from the DSI-Poised fragment Library (DSI-PL)

Sl. No.	Structures	No. in Manuscript	CAS Registry Number	"ID"	^{1}H NMR Data Repository BMRB ID	ALATIS InChI
**131**	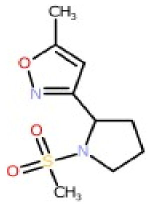	n.a.	1185124-04-2	Z319891284	bmse011102	InChI=1S/C9H14N2O3S/c1-7-6-8(10-14-7)9-4-3-5-11(9)15(2,12)13/h6,9H,3-5H2,1-2H3/t9-/m1/s1
**132**	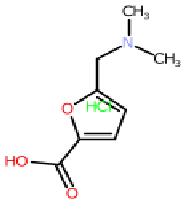	n.a.	1185300-64-4	Z359352902	bmse011173	InChI=1S/C8H11NO3.ClH/c1-9(2)5-6-3-4-7(12-6)8(10)11;/h3-4H,5H2,1-2H3,(H,10,11);1H
**133**	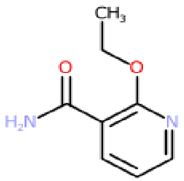	n.a.	119646-51-4	Z272156568	bmse011225	InChI=1S/C8H10N2O2/c1-2-12-8-6(7(9)11)4-3-5-10-8/h3-5H,2H2,1H3,(H2,9,11)
**134**	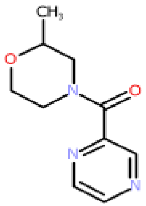	n.a.	1197773-69-5	Z212851096	bmse011489	InChI=1S/C10H13N3O2/c1-8-7-13(4-5-15-8)10(14)9-6-11-2-3-12-9/h2-3,6,8H,4-5,7H2,1H3/t8-/m1/s1
**135**	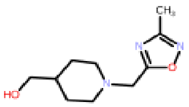	n.a.	1197809-26-9	Z416877194	bmse011496	InChI=1S/C10H17N3O2/c1-8-11-10(15-12-8)6-13-4-2-9(7-14)3-5-13/h9,14H,2-7H2,1H3

Suppl Table 2: Details of the 768 compounds from the DSI-Poised fragment Library (DSI-PL)

Sl. No.	Structures	No. in Manuscript	CAS Registry Number	"ID"	^{1}H NMR Data Repository BMRB ID	ALATIS InChI
**136**	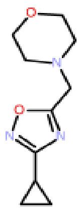	n.a.	1197900-26-7	Z295848548	bmse011490	InChI=1S/C10H15N3O2/c1-2-8(1)10-11-9(15-12-10)7-13-3-5-14-6-4-13/h8H,1-7H2
**137**	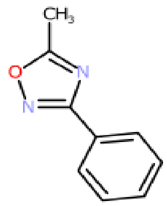	n.a.	1198-98-7	Z57791725	bmse011643	InChI=1S/C9H8N2O/c1-7-10-9(11-12-7)8-5-3-2-4-6-8/h2-6H,1H3
**138**	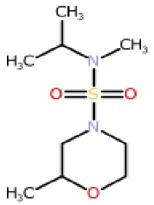	n.a.	1208453-10-4	Z416341642	bmse011492	InChI=1S/C9H20N2O3S/c1-8(2)10(4)15(12,13)11-5-6-14-9(3)7-11/h8-9H,5-7H2,1-4H3/t9-/m1/s1
**139**	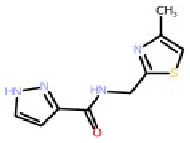	n.a.	1209647-58-4	Z466628048	bmse011208	InChI=1S/C9H10N4OS/c1-6-5-15-8(12-6)4-10-9(14)7-2-3-11-13-7/h2-3,5H,4H2,1H3,(H,10,14)(H,11,13)
**140**	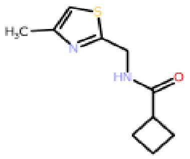	n.a.	1210016-75-3	Z212122838	bmse011420	InChI=1S/C10H14N2OS/c1-7-6-14-9(12-7)5-11-10(13)8-3-2-4-8/h6,8H,2-5H2,1H3,(H,11,13)

Suppl Table 2: Details of the 768 compounds from the DSI-Poised fragment Library (DSI-PL)

Sl. No.	Structures	No. in Manuscript	CAS Registry Number	"ID"	1H NMR Data Repository BMRB ID	ALATIS InChI
**141**	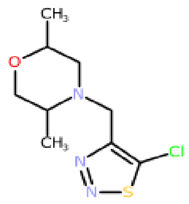	n.a.	1210287-28-7	Z354992234	bmse011516	InChI=1S/C9H14ClN3OS/c1-6-5-14-7(2)3-13(6)4-8-9(10)15-12-11-8/h6-7H,3-5H2,1-2H3/t6-,7+/m0/s1
**142**	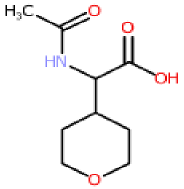	n.a.	1219171-26-2	Z1891772663	bmse011155	InChI=1S/C9H15NO4/c1-6(11)10-8(9(12)13)7-2-4-14-5-3-7/h7-8H,2-5H2,1H3,(H,10,11)(H,12,13)/t8-/m0/s1
**143**	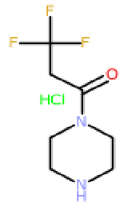	n.a.	1221722-69-5	Z1262246195	bmse011269	InChI=1S/C7H11F3N2O.ClH/c8-7(9,10)5-6(13)12-3-1-11-2-4-12;/h11H,1-5H2;1H
**144**	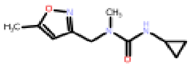	n.a.	1223010-53-4	Z369936976	bmse011501	InChI=1S/C10H15N3O2/c1-7-5-9(12-15-7)6-13(2)10(14)11-8-3-4-8/h5,8H,3-4,6H2,1-2H3,(H,11,14)
**145**	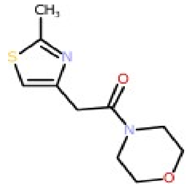	n.a.	1223036-54-1	Z31720228	bmse011497	InChI=1S/C10H14N2O2S/c1-8-11-9(7-15-8)6-10(13)12-2-4-14-5-3-12/h7H,2-6H2,1H3

Suppl Table 2: Details of the 768 compounds from the DSI-Poised fragment Library (DSI-PL)

Sl. No.	Structures	No. in Manuscript	CAS Registry Number	"ID"	1H NMR Data Repository BMRB ID	ALATIS InChI
**146**	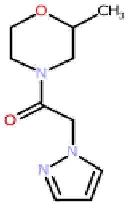	n.a.	1223167-92-7	Z217038356	bmse011210	InChI=1S/C10H15N3O2/c1-9-7-12(5-6-15-9)10(14)8-13-4-2-3-11-13/h2-4,9H,5-8H2,1H3/t9-/m1/s1
**147**	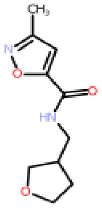	n.a.	1223482-48-1	Z384361454	bmse011030	InChI=1S/C10H14N2O3/c1-7-4-9(15-12-7)10(13)11-5-8-2-3-14-6-8/h4,8H,2-3,5-6H2,1H3,(H,11,13)/t8-/m1/s1
**148**	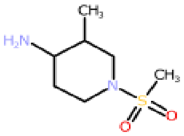	n.a.	1228453-26-6	Z1346370629	bmse011612	InChI=1S/C7H16N2O2S/c1-6-5-9(12(2,10)11)4-3-7(6)8/h6-7H,3-5,8H2,1-2H3/t6-,7+/m1/s1
**149**	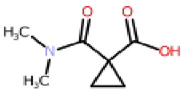	n.a.	1229625-39-1	Z1874937335	bmse011113	InChI=1S/C7H11NO3/c1-8(2)5(9)7(3-4-7)6(10)11/h3-4H2,1-2H3,(H,10,11)
**150**	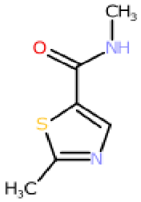	n.a.	1235439-10-7	Z969560582	bmse011218	InChI=1S/C6H8N2OS/c1-4-8-3-5(10-4)6(9)7-2/h3H,1-2H3,(H,7,9)

Suppl Table 2: Details of the 768 compounds from the DSI-Poised fragment Library (DSI-PL)

Sl. No.	Structures	No. in Manuscript	CAS Registry Number	"ID"	1H NMR Data Repository BMRB ID	ALATIS InChI
**151**	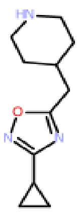	n.a.	1239851-15-0	Z2442270563	bmse011575	InChI=1S/C11H17N3O/c1-2-9(1)11-13-10(15-14-11)7-8-3-5-12-6-4-8/h8-9,12H,1-7H2
**152**	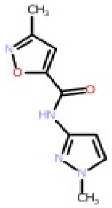	n.a.	1241174-55-9	Z240297434	bmse011217	InChI=1S/C9H10N4O2/c1-6-5-7(15-12-6)9(14)10-8-3-4-13(2)11-8/h3-5H,1-2H3,(H,10,11,14)
**153**	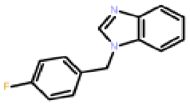	n.a.	124443-67-0	Z26333448	bmse011005	InChI=1S/C14H11FN2/c15-12-7-5-11(6-8-12)9-17-10-16-13-3-1-2-4-14(13)17/h1-8,10H,9H2
**154**	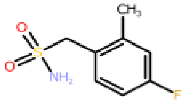	n.a.	1247342-88-6	Z1849009686	bmse011112	InChI=1S/C8H10FNO2S/c1-6-4-8(9)3-2-7(6)5-13(10,11)12/h2-4H,5H2,1H3,(H2,10,11,12)
**155**	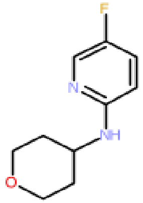	n.a.	1248980-68-8	Z1172243962	bmse011250	InChI=1S/C10H13FN2O/c11-8-1-2-10(12-7-8)13-9-3-5-14-6-4-9/h1-2,7,9H,3-6H2,(H,12,13)

Suppl Table 2: Details of the 768 compounds from the DSI-Poised fragment Library (DSI-PL)

Sl. No.	Structures	No. in Manuscript	CAS Registry Number	"ID"	^{1}H NMR Data Repository BMRB ID	ALATIS InChI
**156**	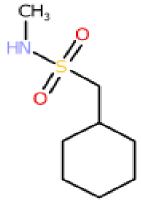	n.a.	1249194-40-8	Z1003207278	bmse011487	InChI=1S/C8H17NO2S/c1-9-12(10,11)7-8-5-3-2-4-6-8/h8-9H,2-7H2,1H3
**157**	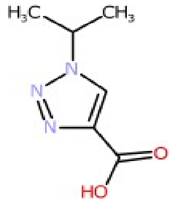	n.a.	1249780-66-2	Z2510259379	bmse011156	InChI=1S/C6H9N3O2/c1-4(2)9-3-5(6(10)11)7-8-9/h3-4H,1-2H3,(H,10,11)
**158**	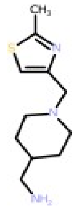	n.a.	1249955-95-0	Z1259335913	bmse011548	InChI=1S/C11H19N3S/c1-9-13-11(8-15-9)7-14-4-2-10(6-12)3-5-14/h8,10H,2-7,12H2,1H3
**159**	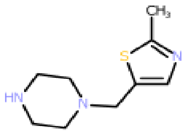	n.a.	1250118-20-7	Z1171217421	bmse011523	InChI=1S/C9H15N3S/c1-8-11-6-9(13-8)7-12-4-2-10-3-5-12/h6,10H,2-5,7H2,1H3
**160**	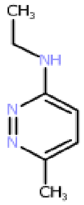	n.a.	1250628-49-9	Z1267773786	bmse011283	InChI=1S/C7H11N3/c1-3-8-7-5-4-6(2)9-10-7/h4-5H,3H2,1-2H3,(H,8,10)

Suppl Table 2: Details of the 768 compounds from the DSI-Poised fragment Library (DSI-PL)

Sl. No.	Structures	No. in Manuscript	CAS Registry Number	"ID"	^{1}H NMR Data Repository BMRB ID	ALATIS InChI
**161**	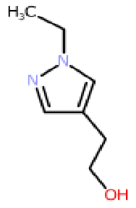	n.a.	1251131-12-0	Z1674937530	bmse011552	InChI=1S/C7H12N2O/c1-2-9-6-7(3-4-<br>10)5-8-9/h5-6,10H,2-4H2,1H3
**162**	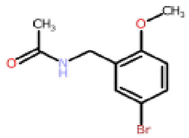	n.a.	1252130-18-9	Z955369596	bmse011230	InChI=1S/C10H12BrNO2/c1-7(13)12-<br>6-8-5-9(11)3-4-10(8)14-2/h3-<br>5H,6H2,1-2H3,(H,12,13)
**163**	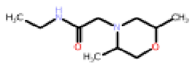	n.a.	1252134-18-1	Z355728146	bmse011500	InChI=1S/C10H20N2O2/c1-4-11-<br>10(13)6-12-5-9(3)14-7-8(12)2/h8-<br>9H,4-7H2,1-3H3,(H,11,13)/t8-<br>,9+/m0/s1
**164**	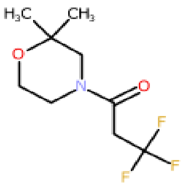	n.a.	1252136-02-9	Z394039592	bmse011234	InChI=1S/C9H14F3NO2/c1-8(2)6-<br>13(3-4-15-8)7(14)5-9(10,11)12/h3-<br>6H2,1-2H3
**165**	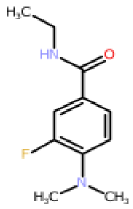	n.a.	1252274-64-8	Z907784200	bmse011236	InChI=1S/C11H15FN2O/c1-4-13-<br>11(15)8-5-6-10(14(2)3)9(12)7-8/h5-<br>7H,4H2,1-3H3,(H,13,15)

Suppl Table 2: Details of the 768 compounds from the DSI-Poised fragment Library (DSI-PL)

Sl. No.	Structures	No. in Manuscript	CAS Registry Number	"ID"	^{1}H NMR Data Repository BMRB ID	ALATIS InChI
**166**	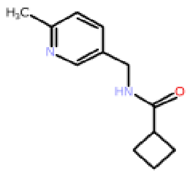	n.a.	1252297-64-5	Z437516460	bmse011235	InChI=1S/C12H16N2O/c1-9-5-6-10(7-13-9)8-14-12(15)11-3-2-4-11/h5-7,11H,2-4,8H2,1H3,(H,14,15)
**167**	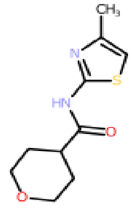	n.a.	1252372-19-2	Z73240835	bmse011238	InChI=1S/C10H14N2O2S/c1-7-6-15-10(11-7)12-9(13)8-2-4-14-5-3-8/h6,8H,2-5H2,1H3,(H,11,12,13)
**168**	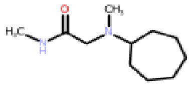	n.a.	1252443-78-9	Z204776284	bmse011498	InChI=1S/C11H22N2O/c1-12-11(14)9-13(2)10-7-5-3-4-6-8-10/h10H,3-9H2,1-2H3,(H,12,14)
**169**	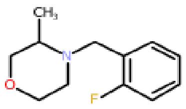	n.a.	1252499-28-7	Z369042042	bmse011499	InChI=1S/C12H16FNO/c1-10-9-15-7-6-14(10)8-11-4-2-3-5-12(11)13/h2-5,10H,6-9H2,1H3/t10-/m0/s1
**170**	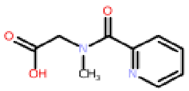	n.a.	125686-77-3	Z1171978788	bmse011249	InChI=1S/C9H10N2O3/c1-11(6-8(12)13)9(14)7-4-2-3-5-10-7/h2-5H,6H2,1H3,(H,12,13)

Suppl Table 2: Details of the 768 compounds from the DSI-Poised fragment Library (DSI-PL)

Sl. No.	Structures	No. in Manuscript	CAS Registry Number	"ID"	^{1}H NMR Data Repository BMRB ID	ALATIS InChI
**171**	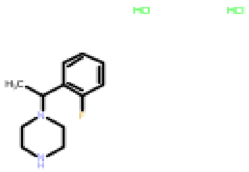	n.a.	1258649-78-3	Z2466069494	bmse011282	InChI=1S/C12H17FN2.2ClH/c1-10(15-8-6-14-7-9-15)11-4-2-3-5-12(11)13;;/h2-5,10,14H,6-9H2,1H3;2*1H/t10-;;/m0../s1
**172**	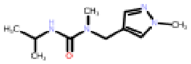	n.a.	1258705-03-1	Z183344018	bmse011515	InChI=1S/C10H18N4O/c1-8(2)12-10(15)13(3)6-9-5-11-14(4)7-9/h5,7-8H,6H2,1-4H3,(H,12,15)
**173**	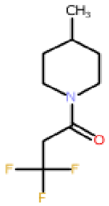	n.a.	1258720-49-8	Z316425948	bmse011503	InChI=1S/C9H14F3NO/c1-7-2-4-13(5-3-7)8(14)6-9(10,11)12/h7H,2-6H2,1H3
**174**	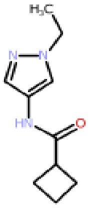	n.a.	1259209-34-1	Z373768900	bmse011511	InChI=1S/C10H15N3O/c1-2-13-7-9(6-11-13)12-10(14)8-4-3-5-8/h6-8H,2-5H2,1H3,(H,12,14)
**175**	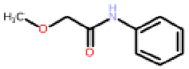	n.a.	126191-21-7	Z31504642	bmse011585	InChI=1S/C9H11NO2/c1-12-7-9(11)10-8-5-3-2-4-6-8/h2-6H,7H2,1H3,(H,10,11)

Suppl Table 2: Details of the 768 compounds from the DSI-Poised fragment Library (DSI-PL)

Sl. No.	Structures	No. in Manuscript	CAS Registry Number	"ID"	1H NMR Data Repository BMRB ID	ALATIS InChI
**176**	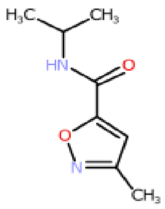	n.a.	126243-11-6	Z135394292	bmse011226	InChI=1S/C8H12N2O2/c1-5(2)9-8(11)7-4-6(3)10-12-7/h4-5H,1-3H3,(H,9,11)
**177**	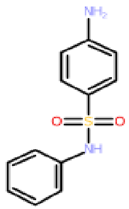	n.a.	127-77-5	Z56923284	bmse011436	InChI=1S/C12H12N2O2S/c13-10-6-8-12(9-7-10)17(15,16)14-11-4-2-1-3-5-11/h1-9,14H,13H2
**178**	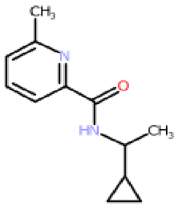	n.a.	1280825-17-3	Z287256168	bmse011597	InChI=1S/C12H16N2O/c1-8-4-3-5-11(13-8)12(15)14-9(2)10-6-7-10/h3-5,9-10H,6-7H2,1-2H3,(H,14,15)/t9-/m1/s1
**179**	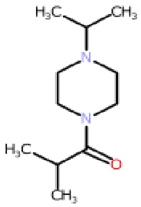	n.a.	1280837-87-7	Z106307058	bmse011601	InChI=1S/C11H22N2O/c1-9(2)11(14)13-7-5-12(6-8-13)10(3)4/h9-10H,5-8H2,1-4H3
**180**	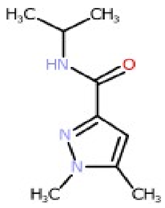	n.a.	1280844-52-1	Z733898630	bmse011599	InChI=1S/C9H15N3O/c1-6(2)10-9(13)8-5-7(3)12(4)11-8/h5-6H,1-4H3,(H,10,13)

Suppl Table 2: Details of the 768 compounds from the DSI-Poised fragment Library (DSI-PL)

Sl. No.	Structures	No. in Manuscript	CAS Registry Number	"ID"	1H NMR Data Repository BMRB ID	ALATIS InChI
**181**	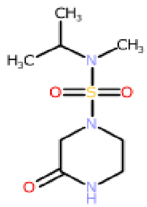	n.a.	1280851-40-2	Z431807512	bmse011233	InChI=1S/C8H17N3O3S/c1-7(2)10(3)15(13,14)11-5-4-9-8(12)6-11/h7H,4-6H2,1-3H3,(H,9,12)
**182**	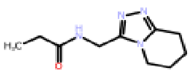	n.a.	1280931-26-1	Z438067480	bmse011243	InChI=1S/C10H16N4O/c1-2-10(15)11-7-9-13-12-8-5-3-4-6-14(8)9/h2-7H2,1H3,(H,11,15)
**183**	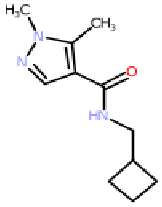	n.a.	1281013-68-0	Z396380540	bmse011248	InChI=1S/C11H17N3O/c1-8-10(7-13-14(8)2)11(15)12-6-9-4-3-5-9/h7,9H,3-6H2,1-2H3,(H,12,15)
**184**	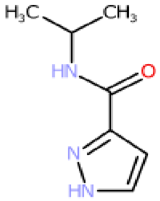	n.a.	1281118-68-0	Z381729066	bmse011224	InChI=1S/C7H11N3O/c1-5(2)9-7(11)6-3-4-8-10-6/h3-5H,1-2H3,(H,8,10)(H,9,11)
**185**	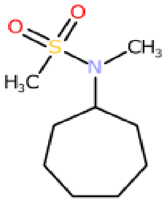	n.a.	1281160-88-0	Z216450634	bmse011507	InChI=1S/C9H19NO2S/c1-10(13(2,11)12)9-7-5-3-4-6-8-9/h9H,3-8H2,1-2H3

Suppl Table 2: Details of the 768 compounds from the DSI-Poised fragment Library (DSI-PL)

Sl. No.	Structures	No. in Manuscript	CAS Registry Number	"ID"	1H NMR Data Repository BMRB ID	ALATIS InChI
186	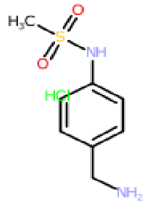	n.a.	128263-66-1	Z426041412	bmse011094	InChI=1S/C8H12N2O2S.ClH/c1-13(11,12)10-8-4-2-7(6-9)3-5-8;/h2-5,10H,6,9H2,1H3;1H
187	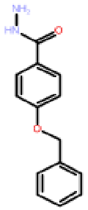	n.a.	128958-65-6	Z58982727	bmse011454	InChI=1S/C14H14N2O2/c15-16-14(17)12-6-8-13(9-7-12)18-10-11-4-2-1-3-5-11/h1-9H,10,15H2,(H,16,17)
188	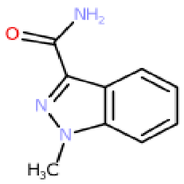	n.a.	129137-93-5	Z2697514548	bmse011754	InChI=1S/C9H9N3O/c1-12-7-5-3-2-4-6(7)8(11-12)9(10)13/h2-5H,1H3,(H2,10,13)
189	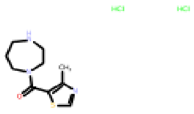	n.a.	1306606-53-0	Z803153598	bmse011172	InChI=1S/C10H15N3OS.2ClH/c1-8-9(15-7-12-8)10(14)13-5-2-3-11-4-6-13;;/h7,11H,2-6H2,1H3;2*1H
190	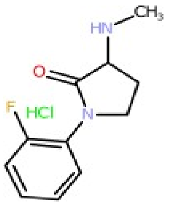	n.a.	1311313-52-6	Z1186029914	bmse011763	InChI=1S/C11H13FN2O.ClH/c1-13-9-6-7-14(11(9)15)10-5-3-2-4-8(10)12;/h2-5,9,13H,6-7H2,1H3;1H/t9-;/m0./s1

Suppl Table 2: Details of the 768 compounds from the DSI-Poised fragment Library (DSI-PL)

Sl. No.	Structures	No. in Manuscript	CAS Registry Number	"ID"	^{1}H NMR Data Repository BMRB ID	ALATIS InChI
**191**	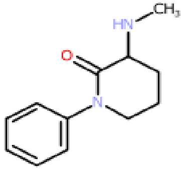	n.a.	1311313-94-6	Z1212984951	bmse011286	InChI=1S/C12H16N2O/c1-13-11-8-5-9-14(12(11)15)10-6-3-2-4-7-10/h2-4,6-7,11,13H,5,8-9H2,1H3/t11-/m0/s1
**192**	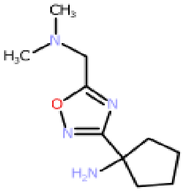	n.a.	1311318-30-5	Z1259339735	bmse011518	InChI=1S/C10H18N4O/c1-14(2)7-8-12-9(13-15-8)10(11)5-3-4-6-10/h3-7,11H2,1-2H3
**193**	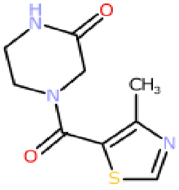	n.a.	1311779-47-1	Z422344882	bmse011096	InChI=1S/C9H11N3O2S/c1-6-8(15-5-11-6)9(14)12-3-2-10-7(13)4-12/h5H,2-4H2,1H3,(H,10,13)
**194**	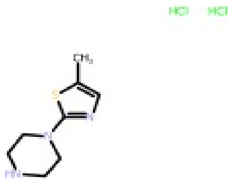	n.a.	1315368-08-1	Z1741975423	bmse011287	InChI=1S/C8H13N3S.2ClH/c1-7-6-10-8(12-7)11-4-2-9-3-5-11;;/h6,9H,2-5H2,1H3;2*1H
**195**	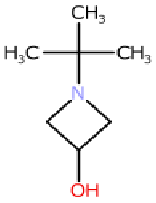	n.a.	13156-04-2	Z2856434900	bmse011510	InChI=1S/C7H15NO/c1-7(2,3)8-4-6(9)5-8/h6,9H,4-5H2,1-3H3

Suppl Table 2: Details of the 768 compounds from the DSI-Poised fragment Library (DSI-PL)

Sl. No.	Structures	No. in Manuscript	CAS Registry Number	"ID"	^{1}H NMR Data Repository BMRB ID	ALATIS InChI
**196**	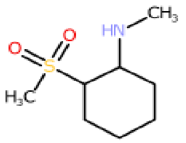	n.a.	131946-93-5	Z1259161657	bmse011520	InChI=1S/C8H17NO2S/c1-9-7-5-3-4-6-8(7)12(2,10)11/h7-9H,3-6H2,1-2H3/t7-,8-/m0/s1
**197**	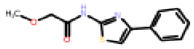	n.a.	132214-37-0	Z62645406	bmse011325	InChI=1S/C12H12N2O2S/c1-16-7-11(15)14-12-13-10(8-17-12)9-5-3-2-4-6-9/h2-6,8H,7H2,1H3,(H,13,14,15)
**198**	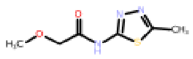	n.a.	132214-40-5	Z263785508	bmse011203	InChI=1S/C6H9N3O2S/c1-4-8-9-6(12-4)7-5(10)3-11-2/h3H2,1-2H3,(H,7,9,10)
**199**	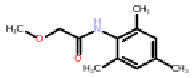	n.a.	133058-08-9	Z57260516	bmse011085	InChI=1S/C12H17NO2/c1-8-5-9(2)12(10(3)6-8)13-11(14)7-15-4/h5-6H,7H2,1-4H3,(H,13,14)
**200**	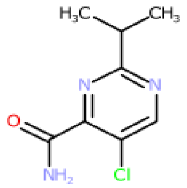	n.a.	1333872-58-4	Z906021418	bmse011255	InChI=1S/C8H10ClN3O/c1-4(2)8-11-3-5(9)6(12-8)7(10)13/h3-4H,1-2H3,(H2,10,13)

Suppl Table 2: Details of the 768 compounds from the DSI-Poised fragment Library (DSI-PL)

Sl. No.	Structures	No. in Manuscript	CAS Registry Number	"ID"	1H NMR Data Repository BMRB ID	ALATIS InChI
**201**	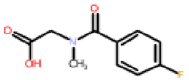	n.a.	133604-67-8	Z85895198	bmse011385	InChI=1S/C10H10FNO3/c1-12(6-9(13)14)10(15)7-2-4-8(11)5-3-7/h2-5H,6H2,1H3,(H,13,14)
**202**	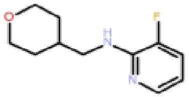	n.a.	1338988-79-6	Z1373430305	bmse011590	InChI=1S/C11H15FN2O/c12-10-2-1-5-13-11(10)14-8-9-3-6-15-7-4-9/h1-2,5,9H,3-4,6-8H2,(H,13,14)
**203**	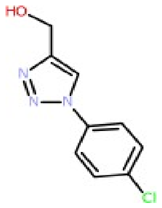	n.a.	133902-66-6	Z1374778753	bmse011397	InChI=1S/C9H8ClN3O/c10-7-1-3-9(4-2-7)13-5-8(6-14)11-12-13/h1-5,14H,6H2
**204**	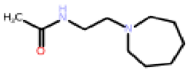	n.a.	1340208-96-9	Z169226638	bmse011569	InChI=1S/C10H20N2O/c1-10(13)11-6-9-12-7-4-2-3-5-8-12/h2-9H2,1H3,(H,11,13)
**205**	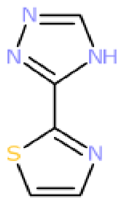	n.a.	1340241-97-5	Z2027049478	bmse011124	InChI=1S/C5H4N4S/c1-2-10-5(6-1)4-7-3-8-9-4/h1-3H,(H,7,8,9)

Suppl Table 2: Details of the 768 compounds from the DSI-Poised fragment Library (DSI-PL)

Sl. No.	Structures	No. in Manuscript	CAS Registry Number	"ID"	^{1}H NMR Data Repository BMRB ID	ALATIS InChI
206	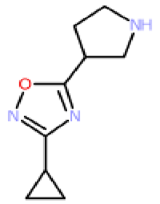	n.a.	1341019-84-8	Z2177153697	bmse011721	InChI=1S/C9H13N3O/c1-2-6(1)8-11-9(13-12-8)7-3-4-10-5-7/h6-7,10H,1-5H2/t7-/m0/s1
207	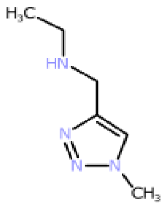	n.a.	1341638-33-2	Z1271660837	bmse011607	InChI=1S/C6H12N4/c1-3-7-4-6-5-10(2)9-8-6/h5,7H,3-4H2,1-2H3
208	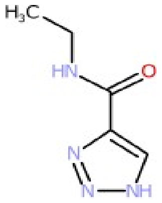	n.a.	1342762-99-5	Z755044716	bmse011417	InChI=1S/C5H8N4O/c1-2-6-5(10)4-3-7-9-8-4/h3H,2H2,1H3,(H,6,10)(H,7,8,9)
209	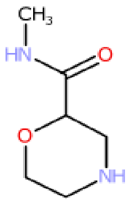	n.a.	135072-21-8	Z1636723439	bmse011460	InChI=1S/C6H12N2O2/c1-7-6(9)5-4-8-2-3-10-5/h5,8H,2-4H2,1H3,(H,7,9)/t5-/m1/s1
210	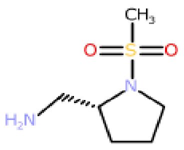	n.a.	1353897-54-7	Z1713595338	bmse011478	InChI=1S/C6H14N2O2S/c1-11(9,10)8-4-2-3-6(8)5-7/h6H,2-5,7H2,1H3/t6-/m1/s1

Suppl Table 2: Details of the 768 compounds from the DSI-Poised fragment Library (DSI-PL)

Sl. No.	Structures	No. in Manuscript	CAS Registry Number	"ID"	1H NMR Data Repository BMRB ID	ALATIS InChI
**211**	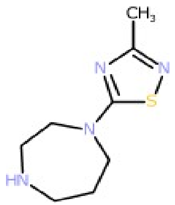	n.a.	1354961-32-2	Z1578665941	bmse011611	InChI=1S/C8H14N4S/c1-7-10-8(13-11-7)12-5-2-3-9-4-6-12/h9H,2-6H2,1H3
**212**	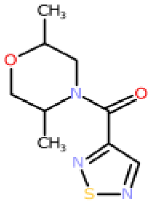	n.a.	1355694-70-0	Z768399682	bmse011506	InChI=1S/C9H13N3O2S/c1-6-5-14-7(2)4-12(6)9(13)8-3-10-15-11-8/h3,6-7H,4-5H2,1-2H3/t6-,7+/m0/s1
**213**	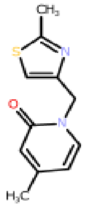	n.a.	1355889-65-4	Z1134990241	bmse011275	InChI=1S/C11H12N2OS/c1-8-3-4-13(11(14)5-8)6-10-7-15-9(2)12-10/h3-5,7H,6H2,1-2H3
**214**	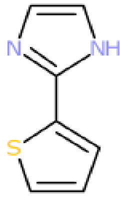	n.a.	136103-77-0	Z744754722	bmse011199	InChI=1S/C7H6N2S/c1-2-6(10-5-1)7-8-3-4-9-7/h1-5H,(H,8,9)
**215**	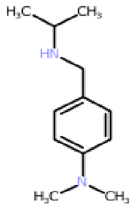	n.a.	137379-64-7	Z2856434894	bmse011509	InChI=1S/C12H20N2/c1-10(2)13-9-11-5-7-12(8-6-11)14(3)4/h5-8,10,13H,9H2,1-4H3

Suppl Table 2: Details of the 768 compounds from the DSI-Poised fragment Library (DSI-PL)

Sl. No.	Structures	No. in Manuscript	CAS Registry Number	"ID"	1H NMR Data Repository BMRB ID	ALATIS InChI
**216**	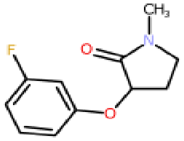	n.a.	1375168-80-1	Z1217960891	bmse011150	InChI=1S/C11H12FNO2/c1-13-6-5-10(11(13)14)15-9-4-2-3-8(12)7-9/h2-4,7,10H,5-6H2,1H3/t10-/m0/s1
**217**	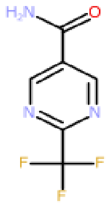	n.a.	1378340-42-1	Z1745658474	bmse011349	InChI=1S/C6H4F3N3O/c7-6(8,9)5-11-1-3(2-12-5)4(10)13/h1-2H,(H2,10,13)
**218**	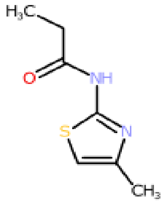	n.a.	13808-59-8	Z30820160	bmse011077	InChI=1S/C7H10N2OS/c1-3-6(10)9-7-8-5(2)4-11-7/h4H,3H2,1-2H3,(H,8,9,10)
**219**	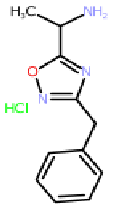	n.a.	1384428-19-6	Z2442288995	bmse011398	InChI=1S/C11H13N3O.ClH/c1-8(12)11-13-10(14-15-11)7-9-5-3-2-4-6-9;/h2-6,8H,7,12H2,1H3;1H/t8-;/m0./s1
**220**	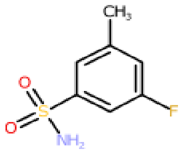	n.a.	1384429-42-8	Z1324080698	bmse011423	InChI=1S/C7H8FNO2S/c1-5-2-6(8)4-7(3-5)12(9,10)11/h2-4H,1H3,(H2,9,10,11)

Suppl Table 2: Details of the 768 compounds from the DSI-Poised fragment Library (DSI-PL)

Sl. No.	Structures	No. in Manuscript	CAS Registry Number	"ID"	1H NMR Data Repository BMRB ID	ALATIS InChI
**221**	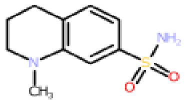	n.a.	1384429-63-3	Z1367324110	bmse011402	InChI=1S/C10H14N2O2S/c1-12-6-2-3-8-4-5-9(7-10(8)12)15(11,13)14/h4-5,7H,2-3,6H2,1H3,(H2,11,13,14)
**222**	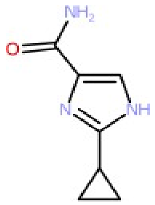	n.a.	1384430-46-9	Z1407672867	bmse011117	InChI=1S/C7H9N3O/c8-6(11)5-3-9-7(10-5)4-1-2-4/h3-4H,1-2H2,(H2,8,11)(H,9,10)
**223**	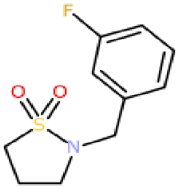	n.a.	1388691-56-2	Z729352906	bmse011562	InChI=1S/C10H12FNO2S/c11-10-4-1-3-9(7-10)8-12-5-2-6-15(12,13)14/h1,3-4,7H,2,5-6,8H2
**224**	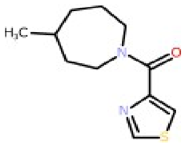	n.a.	1389617-98-4	Z1101755952	bmse011563	InChI=1S/C11H16N2OS/c1-9-3-2-5-13(6-4-9)11(14)10-7-15-8-12-10/h7-9H,2-6H2,1H3/t9-/m0/s1
**225**	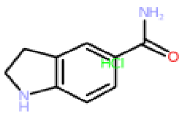	n.a.	1394041-29-2	Z2434225559	bmse011425	InChI=1S/C9H10N2O.ClH/c10-9(12)7-1-2-8-6(5-7)3-4-11-8;/h1-2,5,11H,3-4H2,(H2,10,12);1H

Suppl Table 2: Details of the 768 compounds from the DSI-Poised fragment Library (DSI-PL)

Sl. No.	Structures	No. in Manuscript	CAS Registry Number	"ID"	^{1}H NMR Data Repository BMRB ID	ALATIS InChI
226	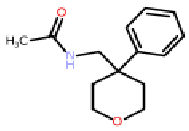	n.a.	14006-33-8	Z384468096	bmse011186	InChI=1S/C14H19NO2/c1-12(16)15-11-14(7-9-17-10-8-14)13-5-3-2-4-6-13/h2-6H,7-11H2,1H3,(H,15,16)
227	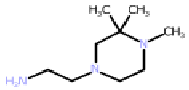	n.a.	1408418-82-5	Z1992316315	bmse011565	InChI=1S/C9H21N3/c1-9(2)8-12(5-4-10)7-6-11(9)3/h4-8,10H2,1-3H3
228	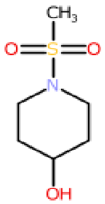	n.a.	141482-19-1	Z1247413608	bmse011391	InChI=1S/C6H13NO3S/c1-11(9,10)7-4-2-6(8)3-5-7/h6,8H,2-5H2,1H3
229	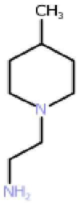	n.a.	14156-95-7	Z1245580461	bmse011546	InChI=1S/C8H18N2/c1-8-2-5-10(6-3-8)7-4-9/h8H,2-7,9H2,1H3
230	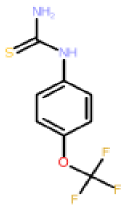	n.a.	142229-74-1	Z1741960769	bmse011755	InChI=1S/C8H7F3N2OS/c9-8(10,11)14-6-3-1-5(2-4-6)13-7(12)15/h1-4H,(H3,12,13,15)

Suppl Table 2: Details of the 768 compounds from the DSI-Poised fragment Library (DSI-PL)

Sl. No.	Structures	No. in Manuscript	CAS Registry Number	"ID"	^{1}H NMR Data Repository BMRB ID	ALATIS InChI
**231**	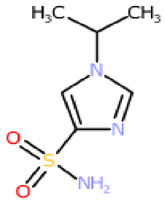	n.a.	1423029-76-8	Z1509882419	bmse011410	InChI=1S/C6H11N3O2S/c1-5(2)9-3-6(8-4-9)12(7,10)11/h3-5H,1-2H3,(H2,7,10,11)
**232**	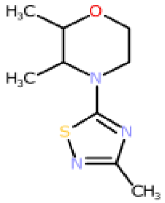	n.a.	1423709-57-2	Z1439422127	bmse011512	InChI=1S/C9H15N3OS/c1-6-7(2)13-5-4-12(6)9-10-8(3)11-14-9/h6-7H,4-5H2,1-3H3/t6-,7+/m0/s1
**233**	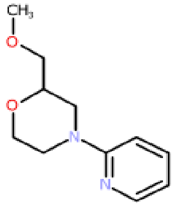	n.a.	1424464-95-8	Z1272480091	bmse011594	InChI=1S/C11H16N2O2/c1-14-9-10-8-13(6-7-15-10)11-4-2-3-5-12-11/h2-5,10H,6-9H2,1H3/t10-/m0/s1
**234**	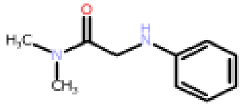	n.a.	14307-89-2	Z104584152	bmse011256	InChI=1S/C10H14N2O/c1-12(2)10(13)8-11-9-6-4-3-5-7-9/h3-7,11H,8H2,1-2H3
**235**	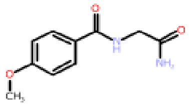	n.a.	143153-70-2	Z33546965	bmse011638	InChI=1S/C10H12N2O3/c1-15-8-4-2-7(3-5-8)10(14)12-6-9(11)13/h2-5H,6H2,1H3,(H2,11,13)(H,12,14)

Suppl Table 2: Details of the 768 compounds from the DSI-Poised fragment Library (DSI-PL)

Sl. No.	Structures	No. in Manuscript	CAS Registry Number	"ID"	^{1}H NMR Data Repository BMRB ID	ALATIS InChI
236	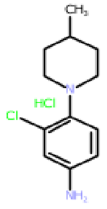	n.a.	1431965-45-5	Z57345491	bmse011760	InChI=1S/C12H17ClN2.ClH/c1-9-4-6-15(7-5-9)12-3-2-10(14)8-11(12)13;/h2-3,8-9H,4-7,14H2,1H3;1H
237	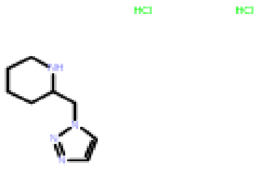	n.a.	1432679-74-7	Z1891776952	bmse011471	InChI=1S/C8H14N4.2ClH/c1-2-4-9-8(3-1)7-12-6-5-10-11-12;;/h5-6,8-9H,1-4,7H2;2*1H/t8-;;/m1../s1
238	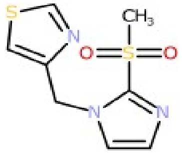	n.a.	1436099-85-2	Z1328968520	bmse011403	InChI=1S/C8H9N3O2S2/c1-15(12,13)8-9-2-3-11(8)4-7-5-14-6-10-7/h2-3,5-6H,4H2,1H3
239	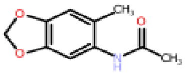	n.a.	1443980-28-6	Z1635496816	bmse011418	InChI=1S/C10H11NO3/c1-6-3-9-10(14-5-13-9)4-8(6)11-7(2)12/h3-4H,5H2,1-2H3,(H,11,12)
240	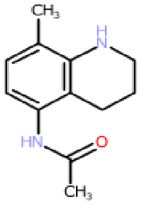	n.a.	1443980-33-3	Z1650168321	bmse011419	InChI=1S/C12H16N2O/c1-8-5-6-11(14-9(2)15)10-4-3-7-13-12(8)10/h5-6,13H,3-4,7H2,1-2H3,(H,14,15)

Suppl Table 2: Details of the 768 compounds from the DSI-Poised fragment Library (DSI-PL)

Sl. No.	Structures	No. in Manuscript	CAS Registry Number	"ID"	^{1}H NMR Data Repository BMRB ID	ALATIS InChI
**241**	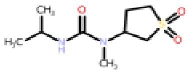	n.a.	1444157-22-5	Z445856640	bmse011288	InChI=1S/C9H18N2O3S/c1-7(2)10-9(12)11(3)8-4-5-15(13,14)6-8/h7-8H,4-6H2,1-3H3,(H,10,12)/t8-/m0/s1
**242**	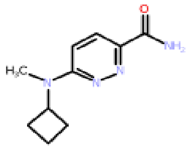	n.a.	1444314-07-1	Z1349163663	bmse011414	InChI=1S/C10H14N4O/c1-14(7-3-2-4-7)9-6-5-8(10(11)15)12-13-9/h5-7H,2-4H2,1H3,(H2,11,15)
**243**	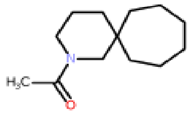	n.a.	1444594-73-3	Z1275599911	bmse011517	InChI=1S/C13H23NO/c1-12(15)14-10-6-9-13(11-14)7-4-2-3-5-8-13/h2-11H2,1H3
**244**	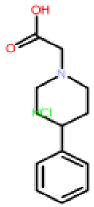	n.a.	1445133-23-2	Z287148438	bmse011415	InChI=1S/C13H17NO2.ClH/c15-13(16)10-14-8-6-12(7-9-14)11-4-2-1-3-5-11;/h1-5,12H,6-10H2,(H,15,16);1H
**245**	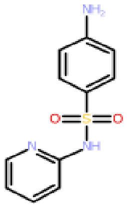	n.a.	144-83-2	Z271004858	bmse011054	InChI=1S/C11H11N3O2S/c12-9-4-6-10(7-5-9)17(15,16)14-11-3-1-2-8-13-11/h1-8H,12H2,(H,13,14)

Suppl Table 2: Details of the 768 compounds from the DSI-Poised fragment Library (DSI-PL)

Sl. No.	Structures	No. in Manuscript	CAS Registry Number	"ID"	^{1}H NMR Data Repository BMRB ID	ALATIS InChI
**246**	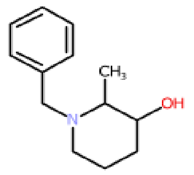	n.a.	1462379-90-3	Z2217052426	bmse011485	InChI=1S/C13H19NO/c1-11-13(15)8-5-9-14(11)10-12-6-3-2-4-7-12/h2-4,6-7,11,13,15H,5,8-10H2,1H3/t11-,13-/m0/s1
**247**	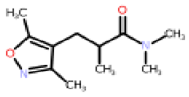	n.a.	1465302-15-1	Z1246465616	bmse011543	InChI=1S/C11H18N2O2/c1-7(11(14)13(4)5)6-10-8(2)12-15-9(10)3/h7H,6H2,1-5H3/t7-/m0/s1
**248**	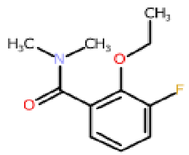	n.a.	1465358-31-9	Z1273312142	bmse011544	InChI=1S/C11H14FNO2/c1-4-15-10-8(11(14)13(2)3)6-5-7-9(10)12/h5-7H,4H2,1-3H3
**249**	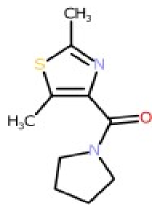	n.a.	1465360-84-2	Z1497321453	bmse011545	InChI=1S/C10H14N2OS/c1-7-9(11-8(2)14-7)10(13)12-5-3-4-6-12/h3-6H2,1-2H3
**250**	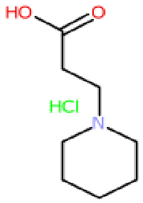	n.a.	14788-15-9	Z53115945	bmse011200	InChI=1S/C8H15NO2.ClH/c10-8(11)4-7-9-5-2-1-3-6-9;/h1-7H2,(H,10,11);1H

Suppl Table 2: Details of the 768 compounds from the DSI-Poised fragment Library (DSI-PL)

Sl. No.	Structures	No. in Manuscript	CAS Registry Number	"ID"	1H NMR Data Repository BMRB ID	ALATIS InChI
**251**	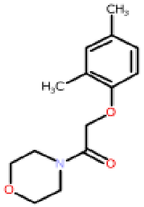	n.a.	148183-90-8	Z17497990	bmse011347	InChI=1S/C14H19NO3/c1-11-3-4-13(12(2)9-11)18-10-14(16)15-5-7-17-8-6-15/h3-4,9H,5-8,10H2,1-2H3
**252**	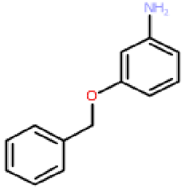	n.a.	1484-26-0	Z933326822	bmse011148	InChI=1S/C13H13NO/c14-12-7-4-8-13(9-12)15-10-11-5-2-1-3-6-11/h1-9H,10,14H2
**253**	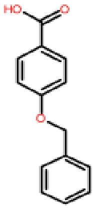	n.a.	1486-51-7	Z2856434845	bmse011433	InChI=1S/C14H12O3/c15-14(16)12-6-8-13(9-7-12)17-10-11-4-2-1-3-5-11/h1-9H,10H2,(H,15,16)
**254**	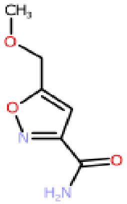	n.a.	1491320-49-0	Z1273141646	bmse011141	InChI=1S/C6H8N2O3/c1-10-3-4-2-5(6(7)9)8-11-4/h2H,3H2,1H3,(H2,7,9)
**255**	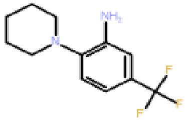	n.a.	1496-40-8	Z56792776	bmse011377	InChI=1S/C12H15F3N2/c13-12(14,15)9-4-5-11(10(16)8-9)17-6-2-1-3-7-17/h4-5,8H,1-3,6-7,16H2

Suppl Table 2: Details of the 768 compounds from the DSI-Poised fragment Library (DSI-PL)

Sl. No.	Structures	No. in Manuscript	CAS Registry Number	"ID"	^{1}H NMR Data Repository BMRB ID	ALATIS InChI
**256**	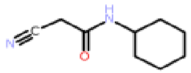	n.a.	15029-38-6	Z56813508	bmse011382	InChI=1S/C9H14N2O/c10-7-6-9(12)11-8-4-2-1-3-5-8/h8H,1-6H2,(H,11,12)
**257**	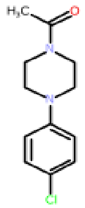	n.a.	150557-99-6	Z275165822	bmse011140	InChI=1S/C12H15ClN2O/c1-10(16)14-6-8-15(9-7-14)12-4-2-11(13)3-5-12/h2-5H,6-9H2,1H3
**258**	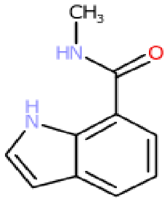	n.a.	1519477-17-8	Z1273312153	bmse011136	InChI=1S/C10H10N2O/c1-11-10(13)8-4-2-3-7-5-6-12-9(7)8/h2-6,12H,1H3,(H,11,13)
**259**	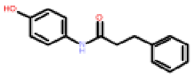	n.a.	152189-77-0	Z69118333	bmse011333	InChI=1S/C15H15NO2/c17-14-9-7-13(8-10-14)16-15(18)11-6-12-4-2-1-3-5-12/h1-5,7-10,17H,6,11H2,(H,16,18)
**260**	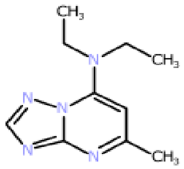	n.a.	15421-84-8	Z56791867	bmse011006	InChI=1S/C10H15N5/c1-4-14(5-2)9-6-8(3)13-10-11-7-12-15(9)10/h6-7H,4-5H2,1-3H3

Suppl Table 2: Details of the 768 compounds from the DSI-Poised fragment Library (DSI-PL)

Sl. No.	Structures	No. in Manuscript	CAS Registry Number	"ID"	1H NMR Data Repository BMRB ID	ALATIS InChI
261	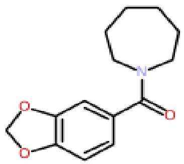	n.a.	154235-79-7	Z31432226	bmse011527	InChI=1S/C14H17NO3/c16-14(15-7-3-1-2-4-8-15)11-5-6-12-13(9-11)18-10-17-12/h5-6,9H,1-4,7-8,10H2
262	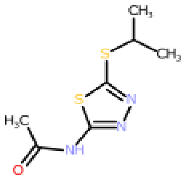	n.a.	154347-50-9	Z1134240160	bmse011178	InChI=1S/C7H11N3OS2/c1-4(2)12-7-10-9-6(13-7)8-5(3)11/h4H,1-3H3,(H,8,9,11)
263	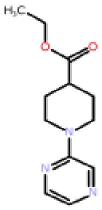	n.a.	154348-18-2	Z31217395	bmse011508	InChI=1S/C12H17N3O2/c1-2-17-12(16)10-3-7-15(8-4-10)11-9-13-5-6-14-11/h5-6,9-10H,2-4,7-8H2,1H3
264	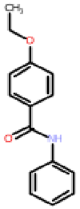	n.a.	15437-13-5	Z27782760	bmse011718	InChI=1S/C15H15NO2/c1-2-18-14-10-8-12(9-11-14)15(17)16-13-6-4-3-5-7-13/h3-11H,2H2,1H3,(H,16,17)
265	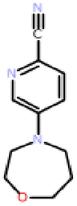	n.a.	1556733-66-4	Z1348371854	bmse011111	InChI=1S/C11H13N3O/c12-8-10-2-3-11(9-13-10)14-4-1-6-15-7-5-14/h2-3,9H,1,4-7H2

Suppl Table 2: Details of the 768 compounds from the DSI-Poised fragment Library (DSI-PL)

Sl. No.	Structures	No. in Manuscript	CAS Registry Number	"ID"	^{1}H NMR Data Repository BMRB ID	ALATIS InChI
**266**	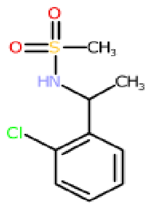	n.a.	1568201-21-7	Z133729708	bmse011430	InChI=1S/C9H12ClNO2S/c1-7(11-14(2,12)13)8-5-3-4-6-9(8)10/h3-7,11H,1-2H3/t7-/m1/s1
**267**	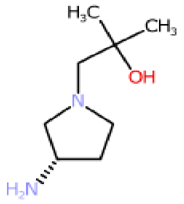	n.a.	1568206-90-5	Z2429435052	bmse011481	InChI=1S/C8H18N2O/c1-8(2,11)6-10-4-3-7(9)5-10/h7,11H,3-6,9H2,1-2H3/t7-/m0/s1
**268**	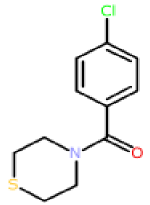	n.a.	158077-85-1	Z437584380	bmse011204	InChI=1S/C11H12ClNOS/c12-10-3-1-9(2-4-10)11(14)13-5-7-15-8-6-13/h1-4H,5-8H2
**269**	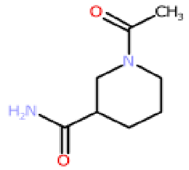	n.a.	15827-99-3	Z220816104	bmse011245	InChI=1S/C8H14N2O2/c1-6(11)10-4-2-3-7(5-10)8(9)12/h7H,2-5H2,1H3,(H2,9,12)/t7-/m0/s1
**270**	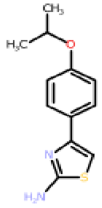	n.a.	15850-31-4	Z969560308	bmse011219	InChI=1S/C12H14N2OS/c1-8(2)15-10-5-3-9(4-6-10)11-7-16-12(13)14-11/h3-8H,1-2H3,(H2,13,14)

Suppl Table 2: Details of the 768 compounds from the DSI-Poised fragment Library (DSI-PL)

Sl. No.	Structures	No. in Manuscript	CAS Registry Number	"ID"	1H NMR Data Repository BMRB ID	ALATIS InChI
271	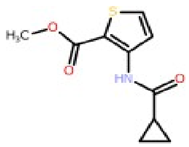	n.a.	158806-51-0	Z109092588	bmse011040	InChI=1S/C10H11NO3S/c1-14-10(13)8-7(4-5-15-8)11-9(12)6-2-3-6/h4-6H,2-3H2,1H3,(H,11,12)
272	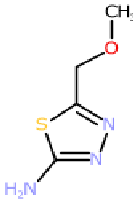	n.a.	15884-86-3	Z57478994	bmse011749	InChI=1S/C4H7N3OS/c1-8-2-3-6-7-4(5)9-3/h2H2,1H3,(H2,5,7)
273	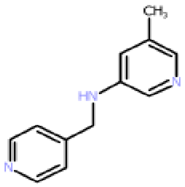	n.a.	1592679-07-6	Z1478435544	bmse011764	InChI=1S/C12H13N3/c1-10-6-12(9-14-7-10)15-8-11-2-4-13-5-3-11/h2-7,9,15H,8H2,1H3
274	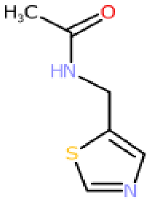	n.a.	1597981-65-1	Z1667545918	bmse011589	InChI=1S/C6H8N2OS/c1-5(9)8-3-6-2-7-4-10-6/h2,4H,3H2,1H3,(H,8,9)
275	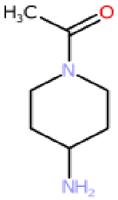	n.a.	160357-94-8	Z90664455	bmse011135	InChI=1S/C7H14N2O/c1-6(10)9-4-2-7(8)3-5-9/h7H,2-5,8H2,1H3

Suppl Table 2: Details of the 768 compounds from the DSI-Poised fragment Library (DSI-PL)

Sl. No.	Structures	No. in Manuscript	CAS Registry Number	"ID"	^{1}H NMR Data Repository BMRB ID	ALATIS InChI
**276**	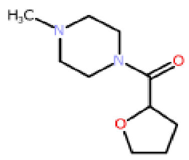	n.a.	1604302-01-3	Z31432964	bmse011756	InChI=1S/C10H18N2O2/c1-11-4-6-12(7-5-11)10(13)9-3-2-8-14-9/h9H,2-8H2,1H3/t9-/m1/s1
**277**	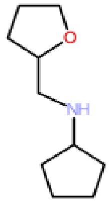	n.a.	1604372-25-9	Z90504169	bmse011535	InChI=1S/C10H19NO/c1-2-5-9(4-1)11-8-10-6-3-7-12-10/h9-11H,1-8H2/t10-/m1/s1
**278**	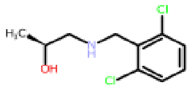	n.a.	1604441-87-3	Z2856434909	bmse011341	InChI=1S/C10H13Cl2NO/c1-7(14)5-13-6-8-9(11)3-2-4-10(8)12/h2-4,7,13-14H,5-6H2,1H3/t7-/m0/s1
**279**	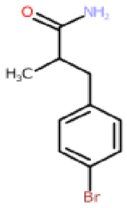	n.a.	1607316-72-2	Z1551999220	bmse011099	InChI=1S/C10H12BrNO/c1-7(10(12)13)6-8-2-4-9(11)5-3-8/h2-5,7H,6H2,1H3,(H2,12,13)/t7-/m1/s1
**280**	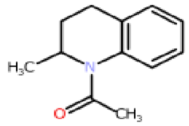	n.a.	16078-42-5	Z28870646	bmse011578	InChI=1S/C12H15NO/c1-9-7-8-11-5-3-4-6-12(11)13(9)10(2)14/h3-6,9H,7-8H2,1-2H3/t9-/m1/s1

Suppl Table 2: Details of the 768 compounds from the DSI-Poised fragment Library (DSI-PL)

Sl. No.	Structures	No. in Manuscript	CAS Registry Number	"ID"	^{1}H NMR Data Repository BMRB ID	ALATIS InChI
281	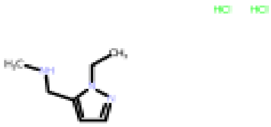	n.a.	1645405-95-3	Z2856434839	bmse011115	InChI=1S/C7H13N3.2ClH/c1-3-10-7(6-8-2)4-5-9-10;;/h4-5,8H,3,6H2,1-2H3;2*1H
282	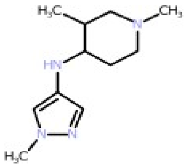	n.a.	1645500-53-3	Z1694504496	bmse011558	InChI=1S/C11H20N4/c1-9-7-14(2)5-4-11(9)13-10-6-12-15(3)8-10/h6,8-9,11,13H,4-5,7H2,1-3H3/t9-,11-/m0/s1
283	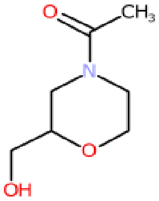	n.a.	1664380-98-6	Z1454840342	bmse011619	InChI=1S/C7H13NO3/c1-6(10)8-2-3-11-7(4-8)5-9/h7,9H,2-5H2,1H3/t7-/m0/s1
284	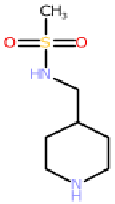	n.a.	166815-15-2	Z1741966151	bmse011125	InChI=1S/C7H16N2O2S/c1-12(10,11)9-6-7-2-4-8-5-3-7/h7-9H,2-6H2,1H3
285	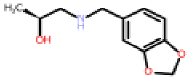	n.a.	1690164-82-9	Z2856434941	bmse011300	InChI=1S/C11H15NO3/c1-8(13)5-12-6-9-2-3-10-11(4-9)15-7-14-10/h2-4,8,12-13H,5-7H2,1H3/t8-/m0/s1

Suppl Table 2: Details of the 768 compounds from the DSI-Poised fragment Library (DSI-PL)

Sl. No.	Structures	No. in Manuscript	CAS Registry Number	"ID"	^{1}H NMR Data Repository BMRB ID	ALATIS InChI
**286**	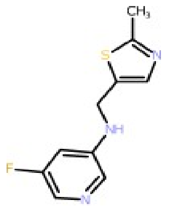	n.a.	1699685-98-7	Z1983897532	bmse011621	InChI=1S/C10H10FN3S/c1-7-13-5-10(15-7)6-14-9-2-8(11)3-12-4-9/h2-5,14H,6H2,1H3
**287**	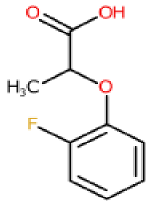	n.a.	17088-71-0	Z65532537	bmse011157	InChI=1S/C9H9FO3/c1-6(9(11)12)13-8-5-3-2-4-7(8)10/h2-6H,1H3,(H,11,12)/t6-/m0/s1
**288**	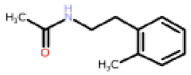	n.a.	171880-36-7	Z52314092	bmse011502	InChI=1S/C11H15NO/c1-9-5-3-4-6-11(9)7-8-12-10(2)13/h3-6H,7-8H2,1-2H3,(H,12,13)
**289**	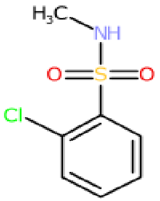	n.a.	17260-67-2	Z45527714	bmse011028	InChI=1S/C7H8ClNO2S/c1-9-12(10,11)7-5-3-2-4-6(7)8/h2-5,9H,1H3
**290**	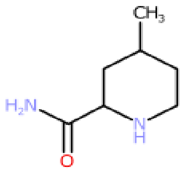	n.a.	172703-83-2	Z1741785925	bmse011104	InChI=1S/C7H14N2O/c1-5-2-3-9-6(4-5)7(8)10/h5-6,9H,2-4H2,1H3,(H2,8,10)/t5-,6-/m1/s1

Suppl Table 2: Details of the 768 compounds from the DSI-Poised fragment Library (DSI-PL)

Sl. No.	Structures	No. in Manuscript	CAS Registry Number	"ID"	^{1}H NMR Data Repository BMRB ID	ALATIS InChI
**291**	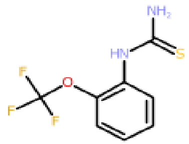	n.a.	175205-24-0	Z291279160	bmse011143	InChI=1S/C8H7F3N2OS/c9-8(10,11)14-6-4-2-1-3-5(6)13-7(12)15/h1-4H,(H3,12,13,15)
**292**	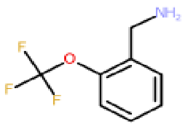	n.a.	175205-64-8	Z2856434776	bmse011584	InChI=1S/C8H8F3NO/c9-8(10,11)13-7-4-2-1-3-6(7)5-12/h1-4H,5,12H2
**293**	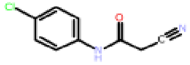	n.a.	17722-17-7	Z56837087	bmse011431	InChI=1S/C9H7ClN2O/c10-7-1-3-8(4-2-7)12-9(13)5-6-11/h1-4H,5H2,(H,12,13)
**294**	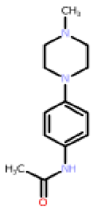	n.a.	17761-86-3	Z2856434903	bmse011145	InChI=1S/C13H19N3O/c1-11(17)14-12-3-5-13(6-4-12)16-9-7-15(2)8-10-16/h3-6H,7-10H2,1-2H3,(H,14,17)
**295**	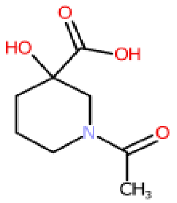	n.a.	1779359-88-4	Z2293643386	bmse011635	InChI=1S/C8H13NO4/c1-6(10)9-4-2-3-8(13,5-9)7(11)12/h13H,2-5H2,1H3,(H,11,12)/t8-/m0/s1

Suppl Table 2: Details of the 768 compounds from the DSI-Poised fragment Library (DSI-PL)

Sl. No.	Structures	No. in Manuscript	CAS Registry Number	"ID"	^{1}H NMR Data Repository BMRB ID	ALATIS InChI
**296**	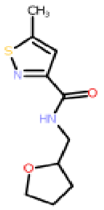	n.a.	1788976-17-9	Z1881545321	bmse011570	InChI=1S/C10H14N2O2S/c1-7-5-9(12-15-7)10(13)11-6-8-3-2-4-14-8/h5,8H,2-4,6H2,1H3,(H,11,13)/t8-/m1/s1
**297**	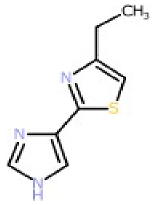	n.a.	1791353-66-6	Z1530301542	bmse011114	InChI=1S/C8H9N3S/c1-2-6-4-12-8(11-6)7-3-9-5-10-7/h3-5H,2H2,1H3,(H,9,10)
**298**	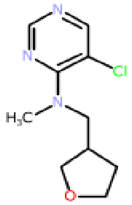	n.a.	1797334-69-0	Z1787627869	bmse011554	InChI=1S/C10H14ClN3O/c1-14(5-8-2-3-15-6-8)10-9(11)4-12-7-13-10/h4,7-8H,2-3,5-6H2,1H3/t8-/m1/s1
**299**	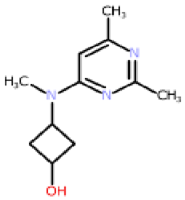	n.a.	1797358-96-3	Z1675346324	bmse011108	InChI=1S/C11H17N3O/c1-7-4-11(13-8(2)12-7)14(3)9-5-10(15)6-9/h4,9-10,15H,5-6H2,1-3H3/t9-,10-
**300**	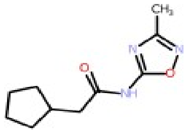	n.a.	1797931-68-0	Z729726784	bmse011229	InChI=1S/C10H15N3O2/c1-7-11-10(15-13-7)12-9(14)6-8-4-2-3-5-8/h8H,2-6H2,1H3,(H,11,12,13,14)

Suppl Table 2: Details of the 768 compounds from the DSI-Poised fragment Library (DSI-PL)

Sl. No.	Structures	No. in Manuscript	CAS Registry Number	"ID"	^{1}H NMR Data Repository BMRB ID	ALATIS InChI
**301**	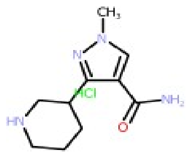	n.a.	1803589-18-5	Z2377835233	bmse011119	InChI=1S/C10H16N4O.ClH/c1-14-6-8(10(11)15)9(13-14)7-3-2-4-12-5-7;/h6-7,12H,2-5H2,1H3,(H2,11,15);1H/t7-;/m0./s1
**302**	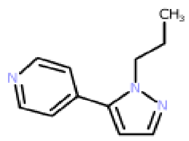	n.a.	1803589-88-9	Z1381484542	bmse011759	InChI=1S/C11H13N3/c1-2-9-14-11(5-8-13-14)10-3-6-12-7-4-10/h3-8H,2,9H2,1H3
**303**	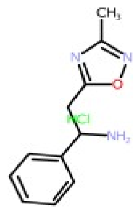	n.a.	1803590-55-7	Z2442269480	bmse011476	InChI=1S/C11H13N3O.ClH/c1-8-13-11(15-14-8)7-10(12)9-5-3-2-4-6-9;/h2-6,10H,7,12H2,1H3;1H/t10-;/m0./s1
**304**	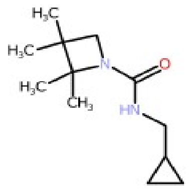	n.a.	1808326-78-4	Z1328078283	bmse011118	InChI=1S/C12H22N2O/c1-11(2)8-14(12(11,3)4)10(15)13-7-9-5-6-9/h9H,5-8H2,1-4H3,(H,13,15)
**305**	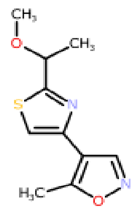	n.a.	1808426-16-5	Z1945710531	bmse011568	InChI=1S/C10H12N2O2S/c1-6-8(4-11-14-6)9-5-15-10(12-9)7(2)13-3/h4-5,7H,1-3H3/t7-/m0/s1

Suppl Table 2: Details of the 768 compounds from the DSI-Poised fragment Library (DSI-PL)

Sl. No.	Structures	No. in Manuscript	CAS Registry Number	"ID"	^{1}H NMR Data Repository BMRB ID	ALATIS InChI
**306**	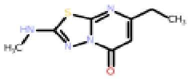	n.a.	1808817-91-5	Z1896598013	bmse011123	InChI=1S/C8H10N4OS/c1-3-5-4-6(13)12-8(10-5)14-7(9-2)11-12/h4H,3H2,1-2H3,(H,9,11)
**307**	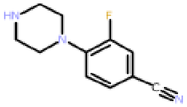	n.a.	182181-38-0	Z939944666	bmse011213	InChI=1S/C11H12FN3/c12-10-7-9(8-13)1-2-11(10)15-5-3-14-4-6-15/h1-2,7,14H,3-6H2
**308**	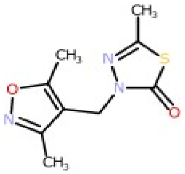	n.a.	1825462-54-1	Z1899842917	bmse011572	InChI=1S/C9H11N3O2S/c1-5-8(6(2)14-11-5)4-12-9(13)15-7(3)10-12/h4H2,1-3H3
**309**	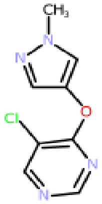	n.a.	1825505-53-0	Z1918536193	bmse011577	InChI=1S/C8H7ClN4O/c1-13-4-6(2-12-13)14-8-7(9)3-10-5-11-8/h2-5H,1H3
**310**	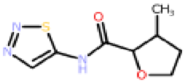	n.a.	1825622-15-8	Z1827602749	bmse011128	InChI=1S/C8H11N3O2S/c1-5-2-3-13-7(5)8(12)10-6-4-9-11-14-6/h4-5,7H,2-3H2,1H3,(H,10,12)/t5-,7+/m0/s1

Suppl Table 2: Details of the 768 compounds from the DSI-Poised fragment Library (DSI-PL)

Sl. No.	Structures	No. in Manuscript	CAS Registry Number	"ID"	^{1}H NMR Data Repository BMRB ID	ALATIS InChI
**311**	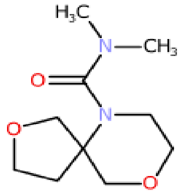	n.a.	1825684-74-9	Z1998104358	bmse011608	InChI=1S/C10H18N2O3/c1-11(2)9(13)12-4-6-15-8-10(12)3-5-14-7-10/h3-8H2,1-2H3/t10-/m1/s1
**312**	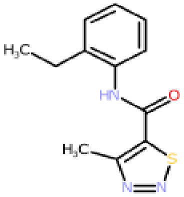	n.a.	183306-35-6	Z89385775	bmse011350	InChI=1S/C12H13N3OS/c1-3-9-6-4-5-7-10(9)13-12(16)11-8(2)14-15-17-11/h4-7H,3H2,1-2H3,(H,13,16)
**313**	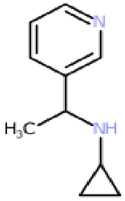	n.a.	183609-12-3	Z1263529721	bmse011542	InChI=1S/C10H14N2/c1-8(12-10-4-5-10)9-3-2-6-11-7-9/h2-3,6-8,10,12H,4-5H2,1H3/t8-/m1/s1
**314**	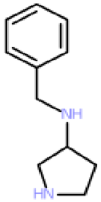	n.a.	184107-56-0	Z1827898537	bmse011555	InChI=1S/C11H16N2/c1-2-4-10(5-3-1)8-13-11-6-7-12-9-11/h1-5,11-13H,6-9H2/t11-/m1/s1
**315**	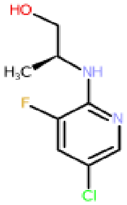	n.a.	1841274-96-1	Z1432018343	bmse011169	InChI=1S/C8H10ClFN2O/c1-5(4-13)12-8-7(10)2-6(9)3-11-8/h2-3,5,13H,4H2,1H3,(H,11,12)/t5-/m0/s1

Suppl Table 2: Details of the 768 compounds from the DSI-Poised fragment Library (DSI-PL)

Sl. No.	Structures	No. in Manuscript	CAS Registry Number	"ID"	1H NMR Data Repository BMRB ID	ALATIS InChI
**316**	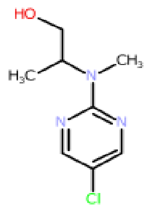	n.a.	1845036-67-0	Z1546887028	bmse011547	InChI=1S/C8H12ClN3O/c1-6(5-13)12(2)8-10-3-7(9)4-11-8/h3-4,6,13H,5H2,1-2H3/t6-/m1/s1
**317**	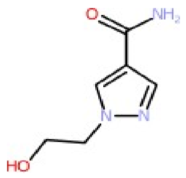	n.a.	1849283-80-2	Z1562205518	bmse011160	InChI=1S/C6H9N3O2/c7-6(11)5-3-8-9(4-5)1-2-10/h3-4,10H,1-2H2,(H2,7,11)
**318**	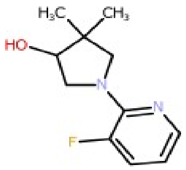	n.a.	1853078-72-4	Z1929757385	bmse011620	InChI=1S/C11H15FN2O/c1-11(2)7-14(6-9(11)15)10-8(12)4-3-5-13-10/h3-5,9,15H,6-7H2,1-2H3/t9-/m0/s1
**319**	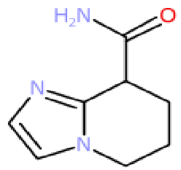	n.a.	185797-36-8	Z1324853681	bmse011404	InChI=1S/C8H11N3O/c9-7(12)6-2-1-4-11-5-3-10-8(6)11/h3,5-6H,1-2,4H2,(H2,9,12)/t6-/m0/s1
**320**	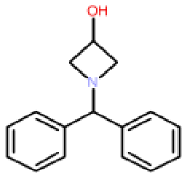	n.a.	18621-17-5	Z1741964527	bmse011097	InChI=1S/C16H17NO/c18-15-11-17(12-15)16(13-7-3-1-4-8-13)14-9-5-2-6-10-14/h1-10,15-16,18H,11-12H2

Suppl Table 2: Details of the 768 compounds from the DSI-Poised fragment Library (DSI-PL)

Sl. No.	Structures	No. in Manuscript	CAS Registry Number	"ID"	1H NMR Data Repository BMRB ID	ALATIS InChI
**321**	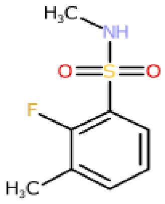	n.a.	1864268-87-0	Z1639162606	bmse011166	InChI=1S/C8H10FNO2S/c1-6-4-3-5-7(8(6)9)13(11,12)10-2/h3-5,10H,1-2H3
**322**	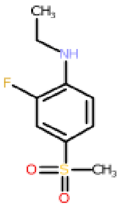	n.a.	1864906-89-7	Z2142244288	bmse011170	InChI=1S/C9H12FNO2S/c1-3-11-9-5-4-7(6-8(9)10)14(2,12)13/h4-6,11H,3H2,1-2H3
**323**	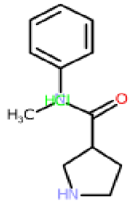	n.a.	1867579-24-5	Z2447286438	bmse011122	InChI=1S/C12H16N2O.ClH/c1-14(11-5-3-2-4-6-11)12(15)10-7-8-13-9-10;/h2-6,10,13H,7-9H2,1H3;1H/t10-;/m0./s1
**324**	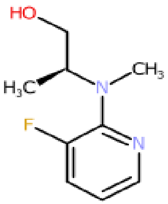	n.a.	1867629-67-1	Z2017168803	bmse011616	InChI=1S/C9H13FN2O/c1-7(6-13)12(2)9-8(10)4-3-5-11-9/h3-5,7,13H,6H2,1-2H3/t7-/m0/s1
**325**	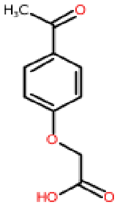	n.a.	1878-81-5	Z2856434937	bmse011435	InChI=1S/C10H10O4/c1-7(11)8-2-4-9(5-3-8)14-6-10(12)13/h2-5H,6H2,1H3,(H,12,13)

Suppl Table 2: Details of the 768 compounds from the DSI-Poised fragment Library (DSI-PL)

Sl. No.	Structures	No. in Manuscript	CAS Registry Number	"ID"	^{1}H NMR Data Repository BMRB ID	ALATIS InChI
**326**	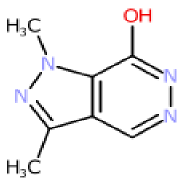	n.a.	1909337-15-0	Z2301438417	bmse011139	InChI=1S/C7H8N4O/c1-4-5-3-8-9-7(12)6(5)11(2)10-4/h3H,1-2H3,(H,9,12)
**327**	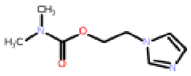	n.a.	191539-21-6	Z1722836661	bmse011630	InChI=1S/C8H13N3O2/c1-10(2)8(12)13-6-5-11-4-3-9-7-11/h3-4,7H,5-6H2,1-2H3
**328**	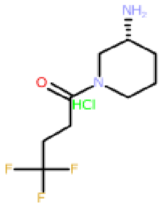	n.a.	1941189-57-6	Z1818332938	bmse011768	InChI=1S/C9H15F3N2O.ClH/c10-9(11,12)4-3-8(15)14-5-1-2-7(13)6-14;/h7H,1-6,13H2;1H/t7-;/m1./s1
**329**	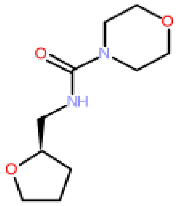	n.a.	1941215-86-6	Z2064898127	bmse011636	InChI=1S/C10H18N2O3/c13-10(12-3-6-14-7-4-12)11-8-9-2-1-5-15-9/h9H,1-8H2,(H,11,13)/t9-/m1/s1
**330**	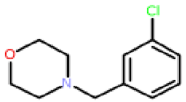	n.a.	196792-37-7	Z2856434856	bmse011514	InChI=1S/C11H14ClNO/c12-11-3-1-2-10(8-11)9-13-4-6-14-7-5-13/h1-3,8H,4-7,9H2

Suppl Table 2: Details of the 768 compounds from the DSI-Poised fragment Library (DSI-PL)

Sl. No.	Structures	No. in Manuscript	CAS Registry Number	"ID"	1H NMR Data Repository BMRB ID	ALATIS InChI
**331**	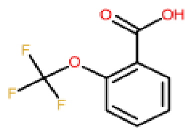	n.a.	1979-29-9	Z2856434778	bmse011758	InChI=1S/C8H5F3O3/c9-8(10,11)14-6-4-2-1-3-5(6)7(12)13/h1-4H,(H,12,13)
**332**	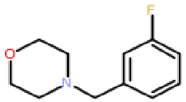	n.a.	198879-71-9	Z2856434791	bmse011505	InChI=1S/C11H14FNO/c12-11-3-1-2-10(8-11)9-13-4-6-14-7-5-13/h1-3,8H,4-7,9H2
**333**	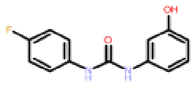	n.a.	199585-00-7	Z55290386	bmse011278	InChI=1S/C13H11FN2O2/c14-9-4-6-10(7-5-9)15-13(18)16-11-2-1-3-12(17)8-11/h1-8,17H,(H2,15,16,18)
**334**	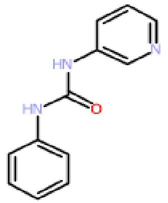	n.a.	2000-55-7	Z44592329	bmse011032	InChI=1S/C12H11N3O/c16-12(14-10-5-2-1-3-6-10)15-11-7-4-8-13-9-11/h1-9H,(H2,14,15,16)
**335**	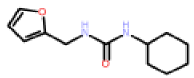	n.a.	200058-87-3	Z44584192	bmse011674	InChI=1S/C12H18N2O2/c15-12(13-9-11-7-4-8-16-11)14-10-5-2-1-3-6-10/h4,7-8,10H,1-3,5-6,9H2,(H2,13,14,15)

Suppl Table 2: Details of the 768 compounds from the DSI-Poised fragment Library (DSI-PL)

Sl. No.	Structures	No. in Manuscript	CAS Registry Number	"ID"	^{1}H NMR Data Repository BMRB ID	ALATIS InChI
**336**	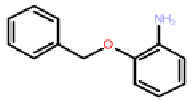	n.a.	20012-63-9	Z336089202	bmse011065	InChI=1S/C13H13NO/c14-12-8-4-5-9-13(12)15-10-11-6-2-1-3-7-11/h1-9H,10,14H2
**337**	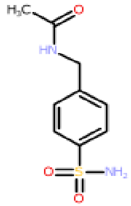	n.a.	2015-14-7	Z30932204	bmse011379	InChI=1S/C9H12N2O3S/c1-7(12)11-6-8-2-4-9(5-3-8)15(10,13)14/h2-5H,6H2,1H3,(H,11,12)(H2,10,13,14)
**338**	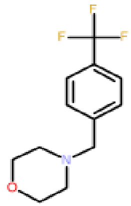	n.a.	201682-13-5	Z2856434871	bmse011493	InChI=1S/C12H14F3NO/c13-12(14,15)11-3-1-10(2-4-11)9-16-5-7-17-8-6-16/h1-4H,5-9H2
**339**	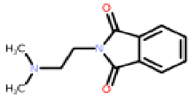	n.a.	20320-52-9	Z2856434895	bmse011378	InChI=1S/C12H14N2O2/c1-13(2)7-8-14-11(15)9-5-3-4-6-10(9)12(14)16/h3-6H,7-8H2,1-2H3
**340**	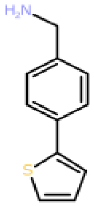	n.a.	203436-48-0	Z2856434805	bmse011477	InChI=1S/C11H11NS/c12-8-9-3-5-10(6-4-9)11-2-1-7-13-11/h1-7H,8,12H2

Suppl Table 2: Details of the 768 compounds from the DSI-Poised fragment Library (DSI-PL)

Sl. No.	Structures	No. in Manuscript	CAS Registry Number	"ID"	^{1}H NMR Data Repository BMRB ID	ALATIS InChI
**341**	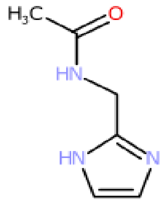	n.a.	203664-03-3	Z1203191681	bmse011167	InChI=1S/C6H9N3O/c1-5(10)9-4-6-7-2-3-8-6/h2-3H,4H2,1H3,(H,7,8)(H,9,10)
**342**	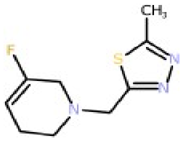	n.a.	2093752-67-9	Z1619978933	bmse011617	InChI=1S/C9H12FN3S/c1-7-11-12-9(14-7)6-13-4-2-3-8(10)5-13/h3H,2,4-6H2,1H3
**343**	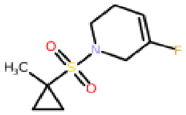	n.a.	2093848-33-8	Z2277255954	bmse011614	InChI=1S/C9H14FNO2S/c1-9(4-5-9)14(12,13)11-6-2-3-8(10)7-11/h3H,2,4-7H2,1H3
**344**	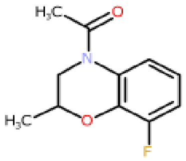	n.a.	2093849-83-1	Z1730522163	bmse011168	InChI=1S/C11H12FNO2/c1-7-6-13(8(2)14)10-5-3-4-9(12)11(10)15-7/h3-5,7H,6H2,1-2H3/t7-/m1/s1
**345**	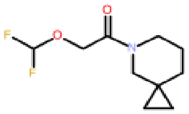	n.a.	2093871-60-2	Z2074076908	bmse011615	InChI=1S/C10H15F2NO2/c11-9(12)15-6-8(14)13-5-1-2-10(7-13)3-4-10/h9H,1-7H2

Suppl Table 2: Details of the 768 compounds from the DSI-Poised fragment Library (DSI-PL)

Sl. No.	Structures	No. in Manuscript	CAS Registry Number	"ID"	^{1}H NMR Data Repository BMRB ID	ALATIS InChI
**346**	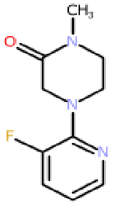	n.a.	2093905-71-4	Z1373445602	bmse011479	InChI=1S/C10H12FN3O/c1-13-5-6-14(7-9(13)15)10-8(11)3-2-4-12-10/h2-4H,5-7H2,1H3
**347**	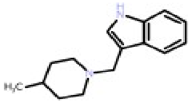	n.a.	21000-95-3	Z2856434912	bmse011720	InChI=1S/C15H20N2/c1-12-6-8-17(9-7-12)11-13-10-16-15-5-3-2-4-14(13)15/h2-5,10,12,16H,6-9,11H2,1H3
**348**	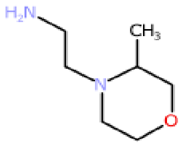	n.a.	2101221-33-2	Z1263811695	bmse011537	InChI=1S/C7H16N2O/c1-7-6-10-5-4-9(7)3-2-8/h7H,2-6,8H2,1H3/t7-/m0/s1
**349**	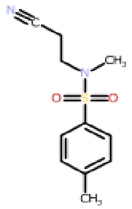	n.a.	21230-34-2	Z45515569	bmse011359	InChI=1S/C11H14N2O2S/c1-10-4-6-11(7-5-10)16(14,15)13(2)9-3-8-12/h4-7H,3,9H2,1-2H3
**350**	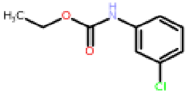	n.a.	2150-89-2	Z31697001	bmse011645	InChI=1S/C9H10ClNO2/c1-2-13-9(12)11-8-5-3-4-7(10)6-8/h3-6H,2H2,1H3,(H,11,12)

Suppl Table 2: Details of the 768 compounds from the DSI-Poised fragment Library (DSI-PL)

Sl. No.	Structures	No. in Manuscript	CAS Registry Number	"ID"	^{1}H NMR Data Repository BMRB ID	ALATIS InChI
**351**	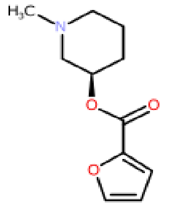	n.a.	2155840-31-4	Z2940170964	bmse011750	InChI=1S/C11H15NO3/c1-12-6-2-4-9(8-12)15-11(13)10-5-3-7-14-10/h3,5,7,9H,2,4,6,8H2,1H3/t9-/m1/s1
**352**	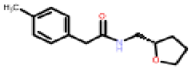	n.a.	2155840-32-5	Z2064898339	bmse011465	InChI=1S/C14H19NO2/c1-11-4-6-12(7-5-11)9-14(16)15-10-13-3-2-8-17-13/h4-7,13H,2-3,8-10H2,1H3,(H,15,16)/t13-/m0/s1
**353**	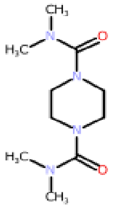	n.a.	21579-91-9	Z57475068	bmse011387	InChI=1S/C10H20N4O2/c1-11(2)9(15)13-5-7-14(8-6-13)10(16)12(3)4/h5-8H2,1-4H3
**354**	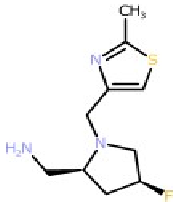	n.a.	2165541-85-3	Z2444997446	bmse011752	InChI=1S/C10H16FN3S/c1-7-13-9(6-15-7)5-14-4-8(11)2-10(14)3-12/h6,8,10H,2-5,12H2,1H3/t8-,10-/m0/s1
**355**	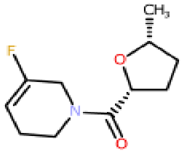	n.a.	2167674-93-1	Z2272040604	bmse011622	InChI=1S/C11H16FNO2/c1-8-4-5-10(15-8)11(14)13-6-2-3-9(12)7-13/h3,8,10H,2,4-7H2,1H3/t8-,10-/m1/s1

Suppl Table 2: Details of the 768 compounds from the DSI-Poised fragment Library (DSI-PL)

Sl. No.	Structures	No. in Manuscript	CAS Registry Number	"ID"	^{1}H NMR Data Repository BMRB ID	ALATIS InChI
**356**	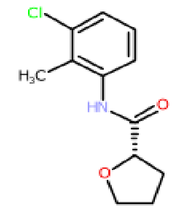	n.a.	2176362-38-0	Z1545312521	bmse011651	InChI=1S/C12H14ClNO2/c1-8-9(13)4-2-5-10(8)14-12(15)11-6-3-7-16-11/h2,4-5,11H,3,6-7H2,1H3,(H,14,15)/t11-/m0/s1
**357**	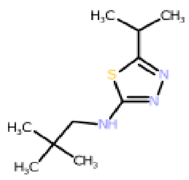	n.a.	218795-46-1	Z1461982627	bmse011688	InChI=1S/C10H19N3S/c1-7(2)8-12-13-9(14-8)11-6-10(3,4)5/h7H,6H2,1-5H3,(H,11,13)
**358**	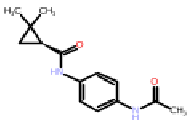	n.a.	2193725-87-8	Z1505719304	bmse011338	InChI=1S/C14H18N2O2/c1-9(17)15-10-4-6-11(7-5-10)16-13(18)12-8-14(12,2)3/h4-7,12H,8H2,1-3H3,(H,15,17)(H,16,18)/t12-/m1/s1
**359**	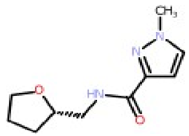	n.a.	2196041-45-7	Z2643472210	bmse011742	InChI=1S/C10H15N3O2/c1-13-5-4-9(12-13)10(14)11-7-8-3-2-6-15-8/h4-5,8H,2-3,6-7H2,1H3,(H,11,14)/t8-/m0/s1
**360**	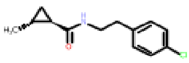	n.a.	2223091-39-0	Z1802166390	bmse011317	InChI=1S/C13H16ClNO/c1-9-8-12(9)13(16)15-7-6-10-2-4-11(14)5-3-10/h2-5,9,12H,6-8H2,1H3,(H,15,16)/t9-,12-/m1/s1

Suppl Table 2: Details of the 768 compounds from the DSI-Poised fragment Library (DSI-PL)

Sl. No.	Structures	No. in Manuscript	CAS Registry Number	"ID"	1H NMR Data Repository BMRB ID	ALATIS InChI
**361**	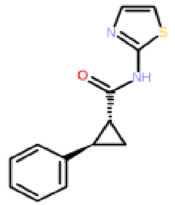	n.a.	2223103-00-0	Z2064107709	bmse011675	InChI=1S/C13H12N2OS/c16-12(15-13-14-6-7-17-13)11-8-10(11)9-4-2-1-3-5-9/h1-7,10-11H,8H2,(H,14,15,16)/t10-,11+/m0/s1
**362**	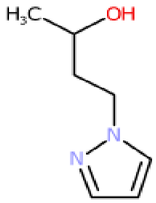	n.a.	2227878-17-1	Z1613492358	bmse011519	InChI=1S/C7H12N2O/c1-7(10)3-6-9-5-2-4-8-9/h2,4-5,7,10H,3,6H2,1H3/t7-/m0/s1
**363**	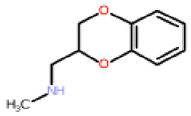	n.a.	2242-31-1	Z1891773476	bmse011441	InChI=1S/C10H13NO2/c1-11-6-8-7-12-9-4-2-3-5-10(9)13-8/h2-5,8,11H,6-7H2,1H3/t8-/m1/s1
**364**	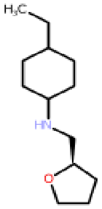	n.a.	2271775-80-3	Z2856434866	bmse011641	InChI=1S/C13H25NO/c1-2-11-5-7-12(8-6-11)14-10-13-4-3-9-15-13/h11-14H,2-10H2,1H3/t11-,12-,13-/m1/s1
**365**	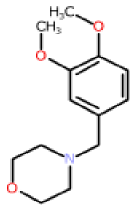	n.a.	22792-30-9	Z2856434878	bmse011678	InChI=1S/C13H19NO3/c1-15-12-4-3-11(9-13(12)16-2)10-14-5-7-17-8-6-14/h3-4,9H,5-8,10H2,1-2H3

Suppl Table 2: Details of the 768 compounds from the DSI-Poised fragment Library (DSI-PL)

Sl. No.	Structures	No. in Manuscript	CAS Registry Number	"ID"	1H NMR Data Repository BMRB ID	ALATIS InChI
**366**	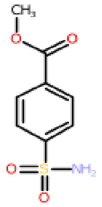	n.a.	22808-73-7	Z18197050	bmse011366	InChI=1S/C8H9NO4S/c1-13-8(10)6-2-4-7(5-3-6)14(9,11)12/h2-5H,1H3,(H2,9,11,12)
**367**	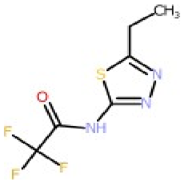	n.a.	22926-50-7	Z275156196	bmse011356	InChI=1S/C6H6F3N3OS/c1-2-3-11-12-5(14-3)10-4(13)6(7,8)9/h2H2,1H3,(H,10,12,13)
**368**	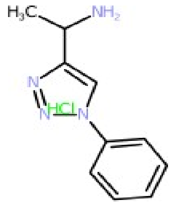	n.a.	2322927-70-6	Z2527301677	bmse011405	InChI=1S/C10H12N4.ClH/c1-8(11)10-7-14(13-12-10)9-5-3-2-4-6-9;/h2-8H,11H2,1H3;1H/t8-;/m0./s1
**369**	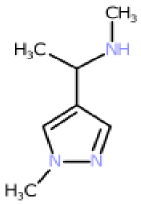	n.a.	2322929-21-3	Z1437171658	bmse011524	InChI=1S/C7H13N3/c1-6(8-2)7-4-9-10(3)5-7/h4-6,8H,1-3H3/t6-/m0/s1
**370**	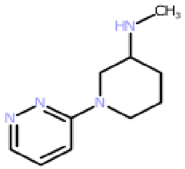	n.a.	2322929-33-7	Z1139246057	bmse011536	InChI=1S/C10H16N4/c1-11-9-4-3-7-14(8-9)10-5-2-6-12-13-10/h2,5-6,9,11H,3-4,7-8H2,1H3/t9-/m0/s1

Suppl Table 2: Details of the 768 compounds from the DSI-Poised fragment Library (DSI-PL)

Sl. No.	Structures	No. in Manuscript	CAS Registry Number	"ID"	^{1}H NMR Data Repository BMRB ID	ALATIS InChI
371	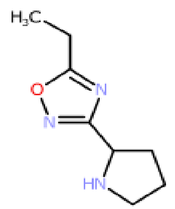	n.a.	2323067-86-1	Z1350579414	bmse011539	InChI=1S/C8H13N3O/c1-2-7-10-8(11-12-7)6-4-3-5-9-6/h6,9H,2-5H2,1H3/t6-/m1/s1
372	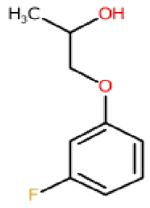	n.a.	2366-93-0	Z1250132544	bmse011534	InChI=1S/C9H11FO2/c1-7(11)6-12-9-4-2-3-8(10)5-9/h2-5,7,11H,6H2,1H3/t7-/m0/s1
373	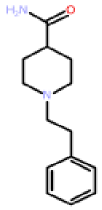	n.a.	23793-55-7	Z2856434897	bmse011061	InChI=1S/C14H20N2O/c15-14(17)13-7-10-16(11-8-13)9-6-12-4-2-1-3-5-12/h1-5,13H,6-11H2,(H2,15,17)
374	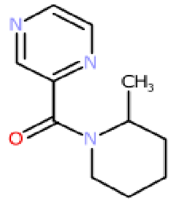	n.a.	2381174-54-3	Z32968340	bmse011613	InChI=1S/C11H15N3O/c1-9-4-2-3-7-14(9)11(15)10-8-12-5-6-13-10/h5-6,8-9H,2-4,7H2,1H3/t9-/m0/s1
375	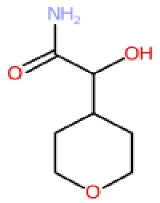	n.a.	2382445-52-3	Z1312590981	bmse011427	InChI=1S/C7H13NO3/c8-7(10)6(9)5-1-3-11-4-2-5/h5-6,9H,1-4H2,(H2,8,10)/t6-/m1/s1

Suppl Table 2: Details of the 768 compounds from the DSI-Poised fragment Library (DSI-PL)

Sl. No.	Structures	No. in Manuscript	CAS Registry Number	"ID"	^{1}H NMR Data Repository BMRB ID	ALATIS InChI
**376**	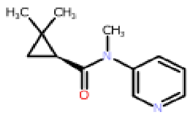	n.a.	2384436-47-7	Z1506050651	bmse011484	InChI=1S/C12H16N2O/c1-12(2)7-10(12)11(15)14(3)9-5-4-6-13-8-9/h4-6,8,10H,7H2,1-3H3/t10-/m1/s1
**377**	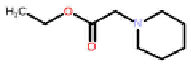	n.a.	23853-10-3	Z2856434773	bmse011055	InChI=1S/C9H17NO2/c1-2-12-9(11)8-10-6-4-3-5-7-10/h2-8H2,1H3
**378**	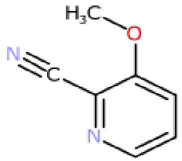	n.a.	24059-89-0	Z1955122823	bmse011154	InChI=1S/C7H6N2O/c1-10-7-3-2-4-9-6(7)5-8/h2-4H,1H3
**379**	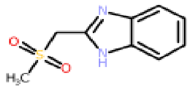	n.a.	24092-75-9	Z126932614	bmse011012	InChI=1S/C9H10N2O2S/c1-14(12,13)6-9-10-7-4-2-3-5-8(7)11-9/h2-5H,6H2,1H3,(H,10,11)
**380**	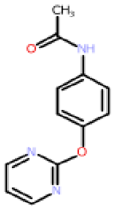	n.a.	24550-10-5	Z54571979	bmse011261	InChI=1S/C12H11N3O2/c1-9(16)15-10-3-5-11(6-4-10)17-12-13-7-2-8-14-12/h2-8H,1H3,(H,15,16)

Suppl Table 2: Details of the 768 compounds from the DSI-Poised fragment Library (DSI-PL)

Sl. No.	Structures	No. in Manuscript	CAS Registry Number	"ID"	^{1}H NMR Data Repository BMRB ID	ALATIS InChI
**381**	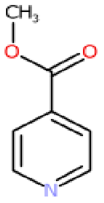	n.a.	2459-09-8	Z19684186	bmse011470	InChI=1S/C7H7NO2/c1-10-7(9)6-2-4-8-5-3-6/h2-5H,1H3
**382**	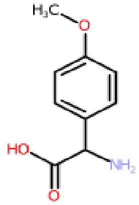	n.a.	24593-49-5	Z56755722	bmse011480	InChI=1S/C9H11NO3/c1-13-7-4-2-6(3-5-7)8(10)9(11)12/h2-5,8H,10H2,1H3,(H,11,12)/t8-/m1/s1
**383**	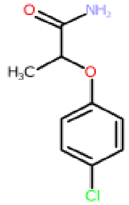	n.a.	24889-00-7	Z19727416	bmse011074	InChI=1S/C9H10ClNO2/c1-6(9(11)12)13-8-4-2-7(10)3-5-8/h2-6H,1H3,(H2,11,12)/t6-/m0/s1
**384**	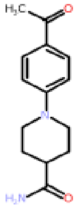	n.a.	250713-72-5	Z126933926	bmse011024	InChI=1S/C14H18N2O2/c1-10(17)11-2-4-13(5-3-11)16-8-6-12(7-9-16)14(15)18/h2-5,12H,6-9H2,1H3,(H2,15,18)
**385**	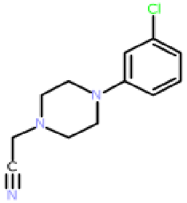	n.a.	25178-91-0	Z2856434806	bmse011163	InChI=1S/C12H14ClN3/c13-11-2-1-3-12(10-11)16-8-6-15(5-4-14)7-9-16/h1-3,10H,5-9H2

Suppl Table 2: Details of the 768 compounds from the DSI-Poised fragment Library (DSI-PL)

Sl. No.	Structures	No. in Manuscript	CAS Registry Number	"ID"	^{1}H NMR Data Repository BMRB ID	ALATIS InChI
386	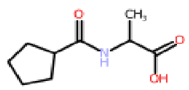	n.a.	252678-52-7	Z1238477790	bmse011253	InChI=1S/C9H15NO3/c1-6(9(12)13)10-8(11)7-4-2-3-5-7/h6-7H,2-5H2,1H3,(H,10,11)(H,12,13)/t6-/m1/s1
387	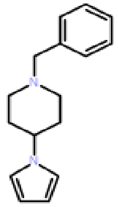	n.a.	254115-91-8	Z2856434898	bmse011494	InChI=1S/C16H20N2/c1-2-6-15(7-3-1)14-17-12-8-16(9-13-17)18-10-4-5-11-18/h1-7,10-11,16H,8-9,12-14H2
388	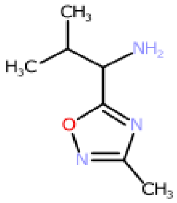	n.a.	254744-73-5	Z1267773566	bmse011533	InChI=1S/C7H13N3O/c1-4(2)6(8)7-9-5(3)10-11-7/h4,6H,8H2,1-3H3/t6-/m0/s1
389	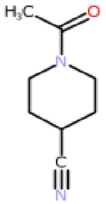	n.a.	25503-91-7	Z952656810	bmse011661	InChI=1S/C8H12N2O/c1-7(11)10-4-2-8(6-9)3-5-10/h8H,2-5H2,1H3
390	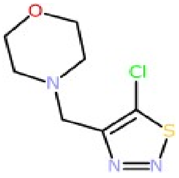	n.a.	256508-44-8	Z2856434925	bmse011247	nChI=1S/C7H10ClN3OS/c8-7-6(9-10-13-7)5-11-1-3-12-4-2-11/h1-5H

Suppl Table 2: Details of the 768 compounds from the DSI-Poised fragment Library (DSI-PL)

Sl. No.	Structures	No. in Manuscript	CAS Registry Number	"ID"	^{1}H NMR Data Repository BMRB ID	ALATIS InChI
**391**	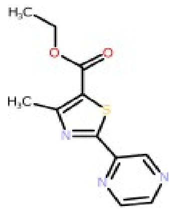	n.a.	256529-18-7	Z735557654	bmse011708	InChI=1S/C11H11N3O2S/c1-3-16-11(15)9-7(2)14-10(17-9)8-6-12-4-5-13-8/h4-6H,3H2,1-2H3
**392**	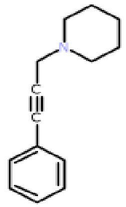	n.a.	2568-57-2	Z57257264	bmse011044	InChI=1S/C14H17N/c1-3-8-14(9-4-1)10-7-13-15-11-5-2-6-12-15/h1,3-4,8-9H,2,5-6,11-13H2
**393**	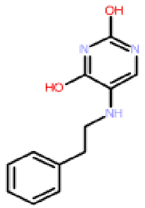	n.a.	25912-34-9	Z57744604	bmse011352	InChI=1S/C12H13N3O2/c16-11-10(8-14-12(17)15-11)13-7-6-9-4-2-1-3-5-9/h1-5,8,13H,6-7H2,(H2,14,15,16,17)
**394**	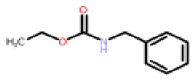	n.a.	2621-78-5	Z31478129	bmse011632	InChI=1S/C10H13NO2/c1-2-13-10(12)11-8-9-6-4-3-5-7-9/h3-7H,2,8H2,1H3,(H,11,12)
**395**	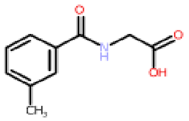	n.a.	27115-49-7	Z56827661	bmse011384	InChI=1S/C10H11NO3/c1-7-3-2-4-8(5-7)10(14)11-6-9(12)13/h2-5H,6H2,1H3,(H,11,14)(H,12,13)

Suppl Table 2: Details of the 768 compounds from the DSI-Poised fragment Library (DSI-PL)

Sl. No.	Structures	No. in Manuscript	CAS Registry Number	"ID"	^{1}H NMR Data Repository BMRB ID	ALATIS InChI
**396**	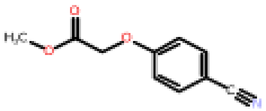	n.a.	272792-14-0	Z19234337	bmse011144	InChI=1S/C10H9NO3/c1-13-10(12)7-14-9-4-2-8(6-11)3-5-9/h2-5H,7H2,1H3
**397**	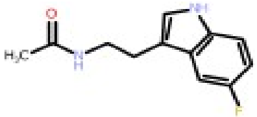	n.a.	2806-01-1	Z1220452176	bmse011339	InChI=1S/C12H13FN2O/c1-8(16)14-5-4-9-7-15-12-3-2-10(13)6-11(9)12/h2-3,6-7,15H,4-5H2,1H3,(H,14,16)
**398**	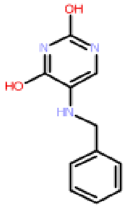	n.a.	28485-19-0	Z111666026	bmse011228	InChI=1S/C11H11N3O2/c15-10-9(7-13-11(16)14-10)12-6-8-4-2-1-3-5-8/h1-5,7,12H,6H2,(H2,13,14,15,16)
**399**	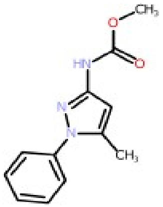	n.a.	287176-87-8	Z1581680287	bmse011706	InChI=1S/C12H13N3O2/c1-9-8-11(13-12(16)17-2)14-15(9)10-6-4-3-5-7-10/h3-8H,1-2H3,(H,13,14,16)
**400**	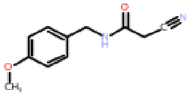	n.a.	288154-72-3	Z190662888	bmse011016	InChI=1S/C11H12N2O2/c1-15-10-4-2-9(3-5-10)8-13-11(14)6-7-12/h2-5H,6,8H2,1H3,(H,13,14)

Suppl Table 2: Details of the 768 compounds from the DSI-Poised fragment Library (DSI-PL)

Sl. No.	Structures	No. in Manuscript	CAS Registry Number	"ID"	1H NMR Data Repository BMRB ID	ALATIS InChI
**401**	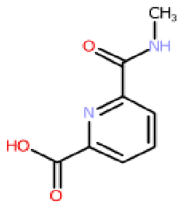	n.a.	288616-99-9	Z1816233707	bmse011110	InChI=1S/C8H8N2O3/c1-9-7(11)5-3-2-4-6(10-5)8(12)13/h2-4H,1H3,(H,9,11)(H,12,13)
**402**	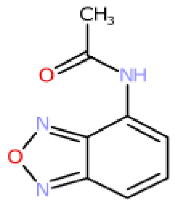	n.a.	289650-01-7	Z2027158783	bmse011694	InChI=1S/C8H7N3O2/c1-5(12)9-6-3-2-4-7-8(6)11-13-10-7/h2-4H,1H3,(H,9,12)
**403**	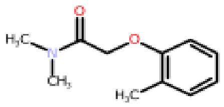	n.a.	29239-56-3	Z19733482	bmse011237	InChI=1S/C11H15NO2/c1-9-6-4-5-7-10(9)14-8-11(13)12(2)3/h4-7H,8H2,1-3H3
**404**	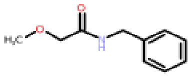	n.a.	2945-05-3	Z31478538	bmse011582	InChI=1S/C10H13NO2/c1-13-8-10(12)11-7-9-5-3-2-4-6-9/h2-6H,7-8H2,1H3,(H,11,12)
**405**	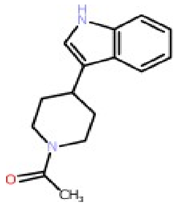	n.a.	30030-83-2	Z223388508	bmse011631	InChI=1S/C15H18N2O/c1-11(18)17-8-6-12(7-9-17)14-10-16-15-5-3-2-4-13(14)15/h2-5,10,12,16H,6-9H2,1H3

Suppl Table 2: Details of the 768 compounds from the DSI-Poised fragment Library (DSI-PL)

Sl. No.	Structures	No. in Manuscript	CAS Registry Number	"ID"	^{1}H NMR Data Repository BMRB ID	ALATIS InChI
**406**	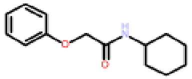	n.a.	303092-41-3	Z19735904	bmse011064	InChI=1S/C14H19NO2/c16-14(15-12-7-3-1-4-8-12)11-17-13-9-5-2-6-10-13/h2,5-6,9-10,12H,1,3-4,7-8,11H2,(H,15,16)
**407**	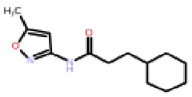	n.a.	303132-67-4	Z86949053	bmse011717	InChI=1S/C13H20N2O2/c1-10-9-12(15-17-10)14-13(16)8-7-11-5-3-2-4-6-11/h9,11H,2-8H2,1H3,(H,14,15,16)
**408**	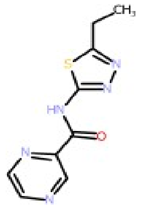	n.a.	303138-15-0	Z26824727	bmse011014	InChI=1S/C9H9N5OS/c1-2-7-13-14-9(16-7)12-8(15)6-5-10-3-4-11-6/h3-5H,2H2,1H3,(H,12,14,15)
**409**	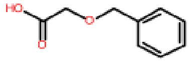	n.a.	30379-55-6	Z2856434906	bmse011765	InChI=1S/C9H10O3/c10-9(11)7-12-6-8-4-2-1-3-5-8/h1-5H,6-7H2,(H,10,11)
**410**	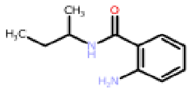	n.a.	30391-87-8	Z56040660	bmse011035	InChI=1S/C11H16N2O/c1-3-8(2)13-11(14)9-6-4-5-7-10(9)12/h4-8H,3,12H2,1-2H3,(H,13,14)/t8-/m1/s1

Suppl Table 2: Details of the 768 compounds from the DSI-Poised fragment Library (DSI-PL)

Sl. No.	Structures	No. in Manuscript	CAS Registry Number	"ID"	^{1}H NMR Data Repository BMRB ID	ALATIS InChI
**411**	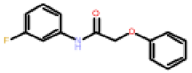	n.a.	303989-18-6	Z19735192	bmse011677	InChI=1S/C14H12FNO2/c15-11-5-4-6-12(9-11)16-14(17)10-18-13-7-2-1-3-8-13/h1-9H,10H2,(H,16,17)
**412**	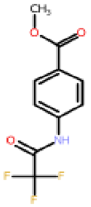	n.a.	304646-56-8	Z56767614	bmse011373	InChI=1S/C10H8F3NO3/c1-17-8(15)6-2-4-7(5-3-6)14-9(16)10(11,12)13/h2-5H,1H3,(H,14,16)
**413**	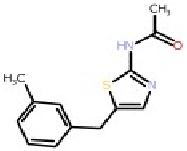	n.a.	304895-11-2	Z57080305	bmse011039	InChI=1S/C13H14N2OS/c1-9-4-3-5-11(6-9)7-12-8-14-13(17-12)15-10(2)16/h3-6,8H,7H2,1-2H3,(H,14,15,16)
**414**	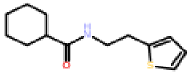	n.a.	308287-68-5	Z29191465	bmse011580	InChI=1S/C13H19NOS/c15-13(11-5-2-1-3-6-11)14-9-8-12-7-4-10-16-12/h4,7,10-11H,1-3,5-6,8-9H2,(H,14,15)
**415**	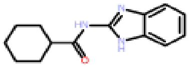	n.a.	312525-49-8	Z27666218	n.a.	n.a.

Suppl Table 2: Details of the 768 compounds from the DSI-Poised fragment Library (DSI-PL)

Sl. No.	Structures	No. in Manuscript	CAS Registry Number	"ID"	^{1}H NMR Data Repository BMRB ID	ALATIS InChI
**416**	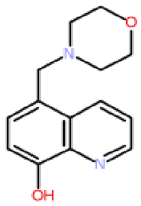	n.a.	312611-90-8	Z2856434798	bmse011422	InChI=1S/C14H16N2O2/c17-13-4-3-11(10-16-6-8-18-9-7-16)12-2-1-5-15-14(12)13/h1-5,17H,6-10H2
**417**	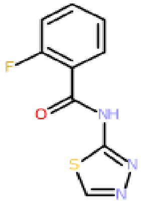	n.a.	313368-15-9	Z86417414	bmse011241	InChI=1S/C9H6FN3OS/c10-7-4-2-1-3-6(7)8(14)12-9-13-11-5-15-9/h1-5H,(H,12,13,14)
**418**	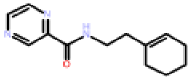	n.a.	313385-77-2	Z69091635	bmse011319	InChI=1S/C13H17N3O/c17-13(12-10-14-8-9-15-12)16-7-6-11-4-2-1-3-5-11/h4,8-10H,1-3,5-7H2,(H,16,17)
**419**	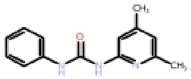	n.a.	313386-33-3	Z1693429442	bmse011175	InChI=1S/C14H15N3O/c1-10-8-11(2)15-13(9-10)17-14(18)16-12-6-4-3-5-7-12/h3-9H,1-2H3,(H2,15,16,17,18)
**420**	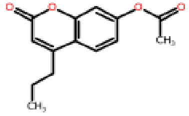	n.a.	313470-98-3	Z29634868	bmse011375	InChI=1S/C14H14O4/c1-3-4-10-7-14(16)18-13-8-11(17-9(2)15)5-6-12(10)13/h5-8H,3-4H2,1-2H3

Suppl Table 2: Details of the 768 compounds from the DSI-Poised fragment Library (DSI-PL)

Sl. No.	Structures	No. in Manuscript	CAS Registry Number	"ID"	1H NMR Data Repository BMRB ID	ALATIS InChI
**421**	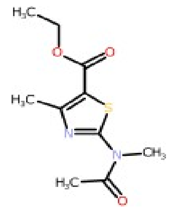	n.a.	3161-69-1	Z1230032143	bmse011291	InChI=1S/C10H14N2O3S/c1-5-15-9(14)8-6(2)11-10(16-8)12(4)7(3)13/h5H2,1-4H3
**422**	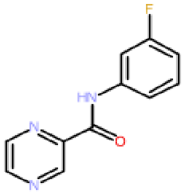	n.a.	320582-40-9	Z28143241	bmse011071	InChI=1S/C11H8FN3O/c12-8-2-1-3-9(6-8)15-11(16)10-7-13-4-5-14-10/h1-7H,(H,15,16)
**423**	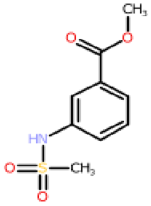	n.a.	32087-05-1	Z24758179	bmse011710	InChI=1S/C9H11NO4S/c1-14-9(11)7-4-3-5-8(6-7)10-15(2,12)13/h3-6,10H,1-2H3
**424**	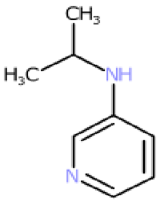	n.a.	32405-76-8	Z1259341037	bmse011267	InChI=1S/C8H12N2/c1-7(2)10-8-4-3-5-9-6-8/h3-7,10H,1-2H3
**425**	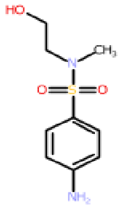	n.a.	328072-15-7	Z1673618163	bmse011296	InChI=1S/C9H14N2O3S/c1-11(6-7-12)15(13,14)9-4-2-8(10)3-5-9/h2-5,12H,6-7,10H2,1H3

Suppl Table 2: Details of the 768 compounds from the DSI-Poised fragment Library (DSI-PL)

Sl. No.	Structures	No. in Manuscript	CAS Registry Number	"ID"	^{1}H NMR Data Repository BMRB ID	ALATIS InChI
**426**	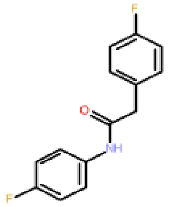	n.a.	328278-71-3	Z27805986	bmse011673	InChI=1S/C14H11F2NO/c15-11-3-1-10(2-4-11)9-14(18)17-13-7-5-12(16)6-8-13/h1-8H,9H2,(H,17,18)
**427**	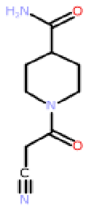	n.a.	328384-63-0	Z57627041	bmse011063	InChI=1S/C9H13N3O2/c10-4-1-8(13)12-5-2-7(3-6-12)9(11)14/h7H,1-3,5-6H2,(H2,11,14)
**428**	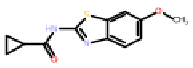	n.a.	32895-11-7	Z27678561	bmse011080	InChI=1S/C12H12N2O2S/c1-16-8-4-5-9-10(6-8)17-12(13-9)14-11(15)7-2-3-7/h4-7H,2-3H2,1H3,(H,13,14,15)
**429**	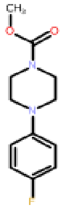	n.a.	329042-42-4	Z192955056	bmse011681	InChI=1S/C12H15FN2O2/c1-17-12(16)15-8-6-14(7-9-15)11-4-2-10(13)3-5-11/h2-5H,6-9H2,1H3
**430**	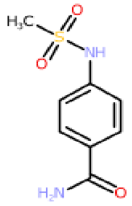	n.a.	329043-17-6	Z45656995	bmse011214	InChI=1S/C8H10N2O3S/c1-14(12,13)10-7-4-2-6(3-5-7)8(9)11/h2-5,10H,1H3,(H2,9,11)

Suppl Table 2: Details of the 768 compounds from the DSI-Poised fragment Library (DSI-PL)

Sl. No.	Structures	No. in Manuscript	CAS Registry Number	"ID"	1H NMR Data Repository BMRB ID	ALATIS InChI
**431**	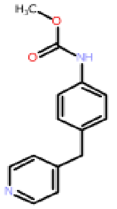	n.a.	329921-01-9	Z57190020	bmse011358	InChI=1S/C14H14N2O2/c1-18-14(17)16-13-4-2-11(3-5-13)10-12-6-8-15-9-7-12/h2-9H,10H2,1H3,(H,16,17)
**432**	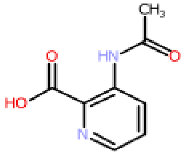	n.a.	3303-18-2	Z1815155460	bmse011109	InChI=1S/C8H8N2O3/c1-5(11)10-6-3-2-4-9-7(6)8(12)13/h2-4H,1H3,(H,10,11)(H,12,13)
**433**	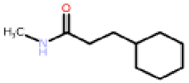	n.a.	330855-40-8	Z32016974	bmse011082	InChI=1S/C10H19NO/c1-11-10(12)8-7-9-5-3-2-4-6-9/h9H,2-8H2,1H3,(H,11,12)
**434**	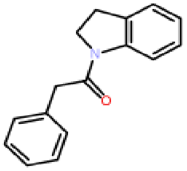	n.a.	331240-55-2	Z30624464	bmse011031	InChI=1S/C16H15NO/c18-16(12-13-6-2-1-3-7-13)17-11-10-14-8-4-5-9-15(14)17/h1-9H,10-12H2
**435**	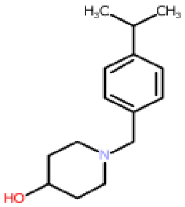	n.a.	331855-73-3	Z2856434874	bmse011313	InChI=1S/C15H23NO/c1-12(2)14-5-3-13(4-6-14)11-16-9-7-15(17)8-10-16/h3-6,12,15,17H,7-11H2,1-2H3

Suppl Table 2: Details of the 768 compounds from the DSI-Poised fragment Library (DSI-PL)

Sl. No.	Structures	No. in Manuscript	CAS Registry Number	"ID"	^{1}H NMR Data Repository BMRB ID	ALATIS InChI
**436**	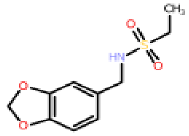	n.a.	332025-91-9	Z53860899	bmse011670	InChI=1S/C10H13NO4S/c1-2-16(12,13)11-6-8-3-4-9-10(5-8)15-7-14-9/h3-5,11H,2,6-7H2,1H3
**437**	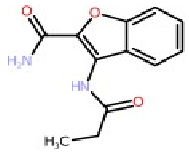	n.a.	332374-98-8	Z108545814	bmse011357	InChI=1S/C12H12N2O3/c1-2-9(15)14-10-7-5-3-4-6-8(7)17-11(10)12(13)16/h3-6H,2H2,1H3,(H2,13,16)(H,14,15)
**438**	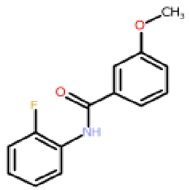	n.a.	333349-76-1	Z28290384	bmse011015	InChI=1S/C14H12FNO2/c1-18-11-6-4-5-10(9-11)14(17)16-13-8-3-2-7-12(13)15/h2-9H,1H3,(H,16,17)
**439**	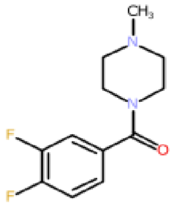	n.a.	333742-29-3	Z2856434829	bmse011662	InChI=1S/C12H14F2N2O/c1-15-4-6-16(7-5-15)12(17)9-2-3-10(13)11(14)8-9/h2-3,8H,4-7H2,1H3
**440**	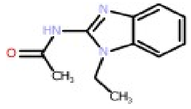	n.a.	333771-27-0	Z68639747	bmse011740	InChI=1S/C11H13N3O/c1-3-14-10-7-5-4-6-9(10)13-11(14)12-8(2)15/h4-7H,3H2,1-2H3,(H,12,13,15)

Suppl Table 2: Details of the 768 compounds from the DSI-Poised fragment Library (DSI-PL)

Sl. No.	Structures	No. in Manuscript	CAS Registry Number	"ID"	1H NMR Data Repository BMRB ID	ALATIS InChI
441	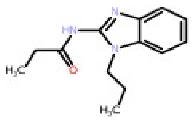	n.a.	333771-28-1	Z29077827	bmse011321	InChI=1S/C13H17N3O/c1-3-9-16-11-8-6-5-7-10(11)14-13(16)15-12(17)4-2/h5-8H,3-4,9H2,1-2H3,(H,14,15,17)
442	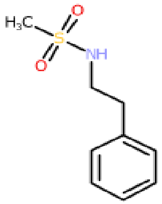	n.a.	33893-36-6	Z45617795	bmse011655	InChI=1S/C9H13NO2S/c1-13(11,12)10-8-7-9-5-3-2-4-6-9/h2-6,10H,7-8H2,1H3
443	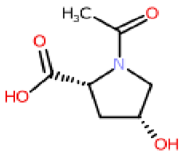	n.a.	33996-33-7	Z1824511473	bmse011468	InChI=1S/C7H11NO4/c1-4(9)8-3-5(10)2-6(8)7(11)12/h5-6,10H,2-3H2,1H3,(H,11,12)/t5-,6-/m1/s1
444	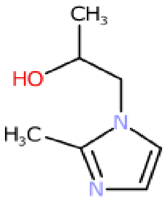	n.a.	34091-34-4	Z1362754425	bmse011540	InChI=1S/C7H12N2O/c1-6(10)5-9-4-3-8-7(9)2/h3-4,6,10H,5H2,1-2H3/t6-/m0/s1
445	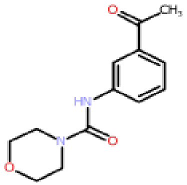	n.a.	341021-30-5	Z274555794	bmse011388	InChI=1S/C13H16N2O3/c1-10(16)11-3-2-4-12(9-11)14-13(17)15-5-7-18-8-6-15/h2-4,9H,5-8H2,1H3,(H,14,17)

Suppl Table 2: Details of the 768 compounds from the DSI-Poised fragment Library (DSI-PL)

Sl. No.	Structures	No. in Manuscript	CAS Registry Number	"ID"	^{1}H NMR Data Repository BMRB ID	ALATIS InChI
**446**	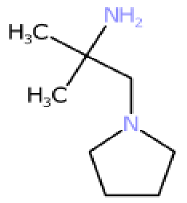	n.a.	34155-39-0	Z1741966630	bmse011609	InChI=1S/C8H18N2/c1-8(2,9)7-10-5-3-4-6-10/h3-7,9H2,1-2H3
**447**	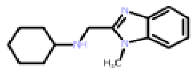	n.a.	342385-30-2	Z2856434855	bmse011381	InChI=1S/C15H21N3/c1-18-14-10-6-5-9-13(14)17-15(18)11-16-12-7-3-2-4-8-12/h5-6,9-10,12,16H,2-4,7-8,11H2,1H3
**448**	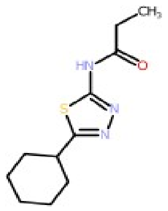	n.a.	342594-15-4	Z165063492	bmse011361	InChI=1S/C11H17N3OS/c1-2-9(15)12-11-14-13-10(16-11)8-6-4-3-5-7-8/h8H,2-7H2,1H3,(H,12,14,15)
**449**	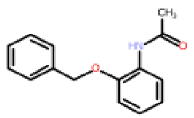	n.a.	34288-19-2	Z26365442	bmse011025	InChI=1S/C15H15NO2/c1-12(17)16-14-9-5-6-10-15(14)18-11-13-7-3-2-4-8-13/h2-10H,11H2,1H3,(H,16,17)
**450**	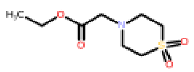	n.a.	343334-01-0	Z2856434920	bmse011252	InChI=1S/C8H15NO4S/c1-2-13-8(10)7-9-3-5-14(11,12)6-4-9/h2-7H2,1H3

Suppl Table 2: Details of the 768 compounds from the DSI-Poised fragment Library (DSI-PL)

Sl. No.	Structures	No. in Manuscript	CAS Registry Number	"ID"	^{1}H NMR Data Repository BMRB ID	ALATIS InChI
451	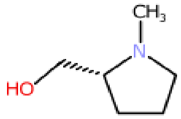	n.a.	#REF!	Z1002568728	bmse011475	InChI=1S/C6H13NO/c1-7-4-2-3-6(7)5-8/h6,8H,2-5H2,1H3/t6-/m1/s1
452	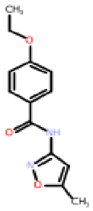	n.a.	346720-95-4	Z86948938	bmse011088	InChI=1S/C13H14N2O3/c1-3-17-11-6-4-10(5-7-11)13(16)14-12-8-9(2)18-15-12/h4-8H,3H2,1-2H3,(H,14,15,16)
453	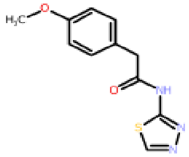	n.a.	346727-10-4	Z86416929	bmse011659	InChI=1S/C11H11N3O2S/c1-16-9-4-2-8(3-5-9)6-10(15)13-11-14-12-7-17-11/h2-5,7H,6H2,1H3,(H,13,14,15)
454	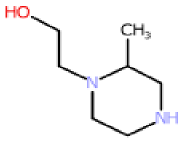	n.a.	3477-42-7	Z1741794237	bmse011550	InChI=1S/C7H16N2O/c1-7-6-8-2-3-9(7)4-5-10/h7-8,10H,2-6H2,1H3/t7-/m0/s1
455	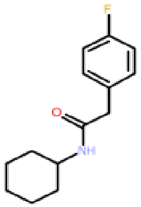	n.a.	348156-13-8	Z31484539	bmse011068	InChI=1S/C14H18FNO/c15-12-8-6-11(7-9-12)10-14(17)16-13-4-2-1-3-5-13/h6-9,13H,1-5,10H2,(H,16,17)

Suppl Table 2: Details of the 768 compounds from the DSI-Poised fragment Library (DSI-PL)

Sl. No.	Structures	No. in Manuscript	CAS Registry Number	"ID"	1H NMR Data Repository BMRB ID	ALATIS InChI
**456**	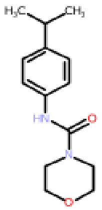	n.a.	349139-26-0	Z208334100	bmse011196	InChI=1S/C14H20N2O2/c1-11(2)12-3-5-13(6-4-12)15-14(17)16-7-9-18-10-8-16/h3-6,11H,7-10H2,1-2H3,(H,15,17)
**457**	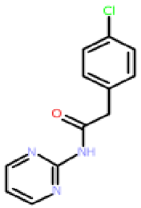	n.a.	349419-74-5	Z203581214	bmse011020	InChI=1S/C12H10ClN3O/c13-10-4-2-9(3-5-10)8-11(17)16-12-14-6-1-7-15-12/h1-7H,8H2,(H,14,15,16,17)
**458**	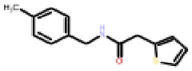	n.a.	349429-04-5	Z28429411	bmse011057	InChI=1S/C14H15NOS/c1-11-4-6-12(7-5-11)10-15-14(16)9-13-3-2-8-17-13/h2-8H,9-10H2,1H3,(H,15,16)
**459**	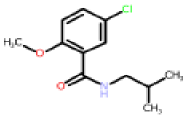	n.a.	349434-28-2	Z32386228	bmse011345	InChI=1S/C12H16ClNO2/c1-8(2)7-14-12(15)10-6-9(13)4-5-11(10)16-3/h4-6,8H,7H2,1-3H3,(H,14,15)
**460**	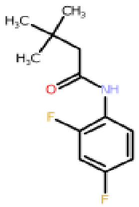	n.a.	349437-14-5	Z30802768	bmse011324	InChI=1S/C12H15F2NO/c1-12(2,3)7-11(16)15-10-5-4-8(13)6-9(10)14/h4-6H,7H2,1-3H3,(H,15,16)

Suppl Table 2: Details of the 768 compounds from the DSI-Poised fragment Library (DSI-PL)

Sl. No.	Structures	No. in Manuscript	CAS Registry Number	"ID"	^{1}H NMR Data Repository BMRB ID	ALATIS InChI
**461**	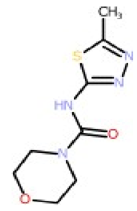	n.a.	349442-33-7	Z410633222	bmse011198	InChI=1S/C8H12N4O2S/c1-6-10-11-7(15-6)9-8(13)12-2-4-14-5-3-12/h2-5H2,1H3,(H,9,11,13)
**462**	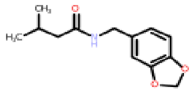	n.a.	349540-63-2	Z27695365	bmse011703	InChI=1S/C13H17NO3/c1-9(2)5-13(15)14-7-10-3-4-11-12(6-10)17-8-16-11/h3-4,6,9H,5,7-8H2,1-2H3,(H,14,15)
**463**	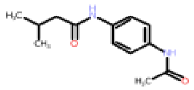	n.a.	349574-58-9	Z27653940	bmse011434	InChI=1S/C13H18N2O2/c1-9(2)8-13(17)15-12-6-4-11(5-7-12)14-10(3)16/h4-7,9H,8H2,1-3H3,(H,14,16)(H,15,17)
**464**	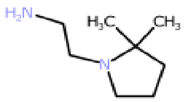	n.a.	35018-17-8	Z1741815708	bmse011574	InChI=1S/C8H18N2/c1-8(2)4-3-6-10(8)7-5-9/h3-7,9H2,1-2H3
**465**	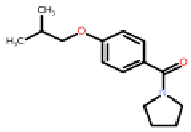	n.a.	350989-16-1	Z31480458	bmse011668	InChI=1S/C15H21NO2/c1-12(2)11-18-14-7-5-13(6-8-14)15(17)16-9-3-4-10-16/h5-8,12H,3-4,9-11H2,1-2H3

Suppl Table 2: Details of the 768 compounds from the DSI-Poised fragment Library (DSI-PL)

Sl. No.	Structures	No. in Manuscript	CAS Registry Number	"ID"	^{1}H NMR Data Repository BMRB ID	ALATIS InChI
466	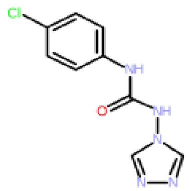	n.a.	351225-32-6	Z56259166	bmse011716	InChI=1S/C9H8ClN5O/c10-7-1-3-8(4-2-7)13-9(16)14-15-5-11-12-6-15/h1-6H,(H2,13,14,16)
467	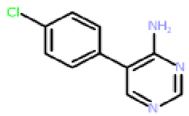	n.a.	35202-25-6	Z1262398388	bmse011273	InChI=1S/C10H8ClN3/c11-8-3-1-7(2-4-8)9-5-13-6-14-10(9)12/h1-6H,(H2,12,13,14)
468	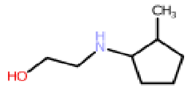	n.a.	35265-10-2	Z1396419547	bmse011559	InChI=1S/C8H17NO/c1-7-3-2-4-8(7)9-5-6-10/h7-10H,2-6H2,1H3/t7-,8-/m0/s1
469	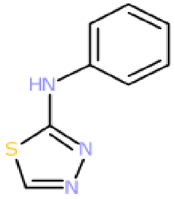	n.a.	3530-62-9	Z56347187	bmse011022	InChI=1S/C8H7N3S/c1-2-4-7(5-3-1)10-8-11-9-6-12-8/h1-6H,(H,10,11)
470	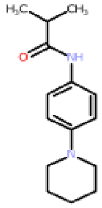	n.a.	354768-54-0	Z30595739	bmse011443	InChI=1S/C15H22N2O/c1-12(2)15(18)16-13-6-8-14(9-7-13)17-10-4-3-5-11-17/h6-9,12H,3-5,10-11H2,1-2H3,(H,16,18)

Suppl Table 2: Details of the 768 compounds from the DSI-Poised fragment Library (DSI-PL)

Sl. No.	Structures	No. in Manuscript	CAS Registry Number	"ID"	^{1}H NMR Data Repository BMRB ID	ALATIS InChI
**471**	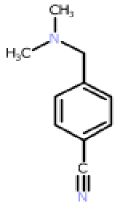	n.a.	35525-86-1	Z2856434815	bmse011567	InChI=1S/C10H12N2/c1-12(2)8-10-5-3-9(7-11)4-6-10/h3-6H,8H2,1-2H3
**472**	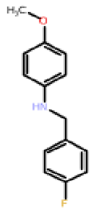	n.a.	356531-43-6	Z57328997	bmse011587	InChI=1S/C14H14FNO/c1-17-14-8-6-13(7-9-14)16-10-11-2-4-12(15)5-3-11/h2-9,16H,10H2,1H3
**473**	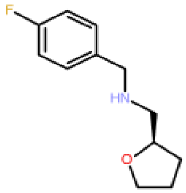	n.a.	356531-65-2	Z2856434824	bmse011743	InChI=1S/C12H16FNO/c13-11-5-3-10(4-6-11)8-14-9-12-2-1-7-15-12/h3-6,12,14H,1-2,7-9H2/t12-/m1/s1
**474**	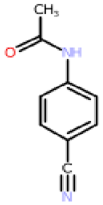	n.a.	35704-19-9	Z30612220	bmse011455	InChI=1S/C9H8N2O/c1-7(12)11-9-4-2-8(6-10)3-5-9/h2-5H,1H3,(H,11,12)
**475**	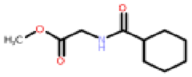	n.a.	35960-89-5	Z18618496	bmse011618	InChI=1S/C10H17NO3/c1-14-9(12)7-11-10(13)8-5-3-2-4-6-8/h8H,2-7H2,1H3,(H,11,13)

Suppl Table 2: Details of the 768 compounds from the DSI-Poised fragment Library (DSI-PL)

Sl. No.	Structures	No. in Manuscript	CAS Registry Number	"ID"	^{1}H NMR Data Repository BMRB ID	ALATIS InChI
**476**	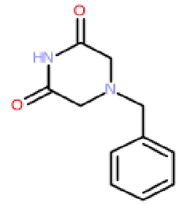	n.a.	35975-17-8	Z1157654117	bmse011767	InChI=1S/C11H12N2O2/c14-10-7-13(8-11(15)12-10)6-9-4-2-1-3-5-9/h1-5H,6-8H2,(H,12,14,15)
**477**	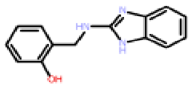	n.a.	364599-60-0	Z1587220559	bmse011372	InChI=1S/C14H13N3O/c18-13-8-4-1-5-10(13)9-15-14-16-11-6-2-3-7-12(11)17-14/h1-8,18H,9H2,(H2,15,16,17)
**478**	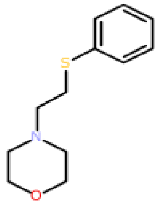	n.a.	364739-42-4	Z2856434865	bmse011647	InChI=1S/C12H17NOS/c1-2-4-12(5-3-1)15-11-8-13-6-9-14-10-7-13/h1-5H,6-11H2
**479**	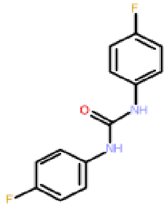	n.a.	370-22-9	Z44586758	bmse011036	InChI=1S/C13H10F2N2O/c14-9-1-5-11(6-2-9)16-13(18)17-12-7-3-10(15)4-8-12/h1-8H,(H2,16,17,18)
**480**	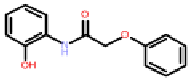	n.a.	3743-69-9	Z68195082	bmse011442	InChI=1S/C14H13NO3/c16-13-9-5-4-8-12(13)15-14(17)10-18-11-6-2-1-3-7-11/h1-9,16H,10H2,(H,15,17)

Suppl Table 2: Details of the 768 compounds from the DSI-Poised fragment Library (DSI-PL)

Sl. No.	Structures	No. in Manuscript	CAS Registry Number	"ID"	^{1}H NMR Data Repository BMRB ID	ALATIS InChI
**481**	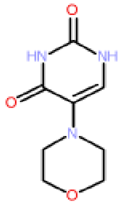	n.a.	37454-52-7	Z57282999	bmse011058	InChI=1S/C8H11N3O3/c12-7-6(5-9-8(13)10-7)11-1-3-14-4-2-11/h5H,1-4H2,(H2,9,10,12,13)
**482**	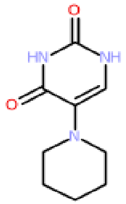	n.a.	37454-53-8	Z277468432	bmse011060	InChI=1S/C9H13N3O2/c13-8-7(6-10-9(14)11-8)12-4-2-1-3-5-12/h6H,1-5H2,(H2,10,11,13,14)
**483**	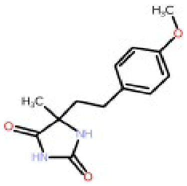	n.a.	374562-58-0	Z56772132	bmse011374	InChI=1S/C13H16N2O3/c1-13(11(16)14-12(17)15-13)8-7-9-3-5-10(18-2)6-4-9/h3-6H,7-8H2,1-2H3,(H2,14,15,16,17)/t13-/m1/s1
**484**	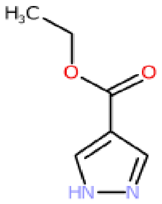	n.a.	37622-90-5	Z1124201124	bmse011396	InChI=1S/C6H8N2O2/c1-2-10-6(9)5-3-7-8-4-5/h3-4H,2H2,1H3,(H,7,8)
**485**	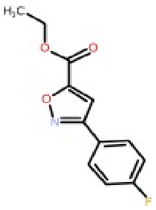	n.a.	377052-00-1	Z2106600670	n.a.	n.a.

Suppl Table 2: Details of the 768 compounds from the DSI-Poised fragment Library (DSI-PL)

Sl. No.	Structures	No. in Manuscript	CAS Registry Number	"ID"	^{1}H NMR Data Repository BMRB ID	ALATIS InChI
**486**	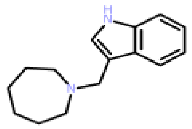	n.a.	378201-57-1	Z2856434848	bmse011368	InChI=1S/C15H20N2/c1-2-6-10-17(9-5-1)12-13-11-16-15-8-4-3-7-14(13)15/h3-4,7-8,11,16H,1-2,5-6,9-10,12H2
**487**	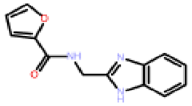	n.a.	378208-16-3	Z26781964	bmse011047	InChI=1S/C13H11N3O2/c17-13(11-6-3-7-18-11)14-8-12-15-9-4-1-2-5-10(9)16-12/h1-7H,8H2,(H,14,17)(H,15,16)
**488**	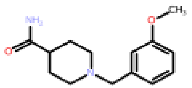	n.a.	380424-20-4	Z2856434854	bmse011299	InChI=1S/C14H20N2O2/c1-18-13-4-2-3-11(9-13)10-16-7-5-12(6-8-16)14(15)17/h2-4,9,12H,5-8,10H2,1H3,(H2,15,17)
**489**	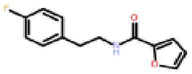	n.a.	380469-52-3	Z26769872	bmse011383	InChI=1S/C13H12FNO2/c14-11-5-3-10(4-6-11)7-8-15-13(16)12-2-1-9-17-12/h1-6,9H,7-8H2,(H,15,16)
**490**	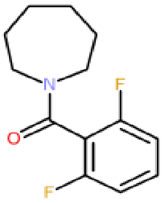	n.a.	385380-70-1	Z54226095	bmse011303	InChI=1S/C13H15F2NO/c14-10-6-5-7-11(15)12(10)13(17)16-8-3-1-2-4-9-16/h5-7H,1-4,8-9H2

Suppl Table 2: Details of the 768 compounds from the DSI-Poised fragment Library (DSI-PL)

Sl. No.	Structures	No. in Manuscript	CAS Registry Number	"ID"	^{1}H NMR Data Repository BMRB ID	ALATIS InChI
**491**	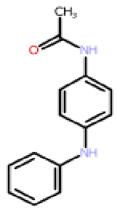	n.a.	38674-90-7	Z68404778	bmse011323	InChI=1S/C14H14N2O/c1-11(17)15-13-7-9-14(10-8-13)16-12-5-3-2-4-6-12/h2-10,16H,1H3,(H,15,17)
**492**	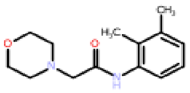	n.a.	389138-76-5	Z2856434834	bmse011336	InChI=1S/C14H20N2O2/c1-11-4-3-5-13(12(11)2)15-14(17)10-16-6-8-18-9-7-16/h3-5H,6-10H2,1-2H3,(H,15,17)
**493**	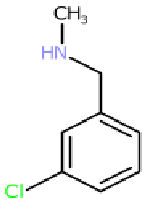	n.a.	39191-07-6	Z2856434770	bmse011579	InChI=1S/C8H10ClN/c1-10-6-7-3-2-4 8(9)5-7/h2-5,10H,6H2,1H3
**494**	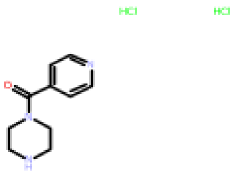	n.a.	39640-05-6	Z126932654	bmse011013	InChI=1S/C10H13N3O.2ClH/c14-10(9-1-3-11-4-2-9)13-7-5-12-6-8-13;;/h1-4,12H,5-8H2;2*1H
**495**	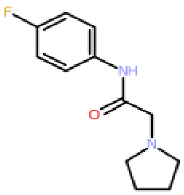	n.a.	397880-93-2	Z2856434942	bmse011421	InChI=1S/C12H15FN2O/c13-10-3-5-11(6-4-10)14-12(16)9-15-7-1-2-8-15/h3-6H,1-2,7-9H2,(H,14,16)

Suppl Table 2: Details of the 768 compounds from the DSI-Poised fragment Library (DSI-PL)

Sl. No.	Structures	No. in Manuscript	CAS Registry Number	"ID"	^{1}H NMR Data Repository BMRB ID	ALATIS InChI
**496**	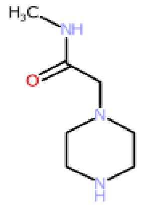	n.a.	39890-41-0	Z85934875	bmse011023	InChI=1S/C7H15N3O/c1-8-7(11)6-10-4-2-9-3-5-10/h9H,2-6H2,1H3,(H,8,11)
**497**	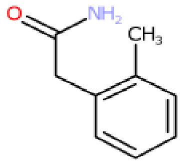	n.a.	40089-14-3	Z52214433	bmse011277	InChI=1S/C9H11NO/c1-7-4-2-3-5-8(7)6-9(10)11/h2-5H,6H2,1H3,(H2,10,11)
**498**	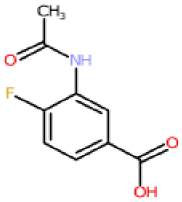	n.a.	401-37-6	Z364328788	bmse011745	InChI=1S/C9H8FNO3/c1-5(12)11-8-4-6(9(13)14)2-3-7(8)10/h2-4H,1H3,(H,11,12)(H,13,14)
**499**	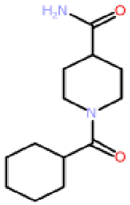	n.a.	401589-79-5	Z32399948	bmse011070	InChI=1S/C13H22N2O2/c14-12(16)10-6-8-15(9-7-10)13(17)11-4-2-1-3-5-11/h10-11H,1-9H2,(H2,14,16)
**500**	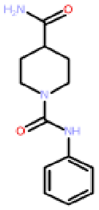	n.a.	401589-80-8	Z33545544	bmse011446	InChI=1S/C13H17N3O2/c14-12(17)10-6-8-16(9-7-10)13(18)15-11-4-2-1-3-5-11/h1-5,10H,6-9H2,(H2,14,17)(H,15,18)

Suppl Table 2: Details of the 768 compounds from the DSI-Poised fragment Library (DSI-PL)

Sl. No.	Structures	No. in Manuscript	CAS Registry Number	"ID"	^{1}H NMR Data Repository BMRB ID	ALATIS InChI
**501**	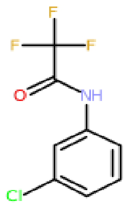	n.a.	40410-54-6	Z111810692	bmse011302	InChI=1S/C8H5ClF3NO/c9-5-2-1-3-6(4-5)13-7(14)8(10,11)12/h1-4H,(H,13,14)
**502**	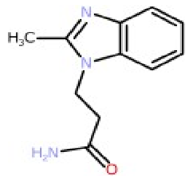	n.a.	40508-01-8	Z53116498	bmse011428	InChI=1S/C11H13N3O/c1-8-13-9-4-2-3-5-10(9)14(8)7-6-11(12)15/h2-5H,6-7H2,1H3,(H2,12,15)
**503**	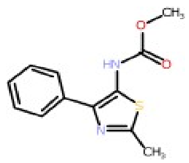	n.a.	41260-96-2	Z979145504	bmse011276	InChI=1S/C12H12N2O2S/c1-8-13-10(9-6-4-3-5-7-9)11(17-8)14-12(15)16-2/h3-7H,1-2H3,(H,14,15)
**504**	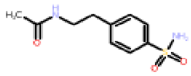	n.a.	41472-49-5	Z30857828	bmse011147	InChI=1S/C10H14N2O3S/c1-8(13)12-7-6-9-2-4-10(5-3-9)16(11,14)15/h2-5H,6-7H2,1H3,(H,12,13)(H2,11,14,15)
**505**	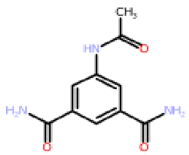	n.a.	414907-43-0	Z33486130	bmse011176	InChI=1S/C10H11N3O3/c1-5(14)13-8-3-6(9(11)15)2-7(4-8)10(12)16/h2-4H,1H3,(H2,11,15)(H2,12,16)(H,13,14)

Suppl Table 2: Details of the 768 compounds from the DSI-Poised fragment Library (DSI-PL)

Sl. No.	Structures	No. in Manuscript	CAS Registry Number	"ID"	1H NMR Data Repository BMRB ID	ALATIS InChI
**506**	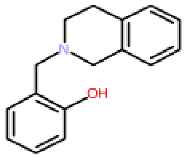	n.a.	415928-50-6	Z2856434881	bmse011709	InChI=1S/C16H17NO/c18-16-8-4-3-7-15(16)12-17-10-9-13-5-1-2-6-14(13)11-17/h1-8,18H,9-12H2
**507**	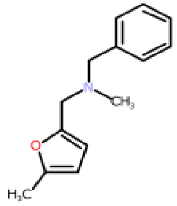	n.a.	415930-18-6	Z2856434896	bmse011486	InChI=1S/C14H17NO/c1-12-8-9-14(16-12)11-15(2)10-13-6-4-3-5-7-13/h3-9H,10-11H2,1-2H3
**508**	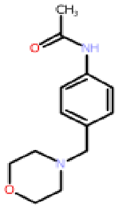	n.a.	415933-46-9	Z2856434875	bmse011192	InChI=1S/C13H18N2O2/c1-11(16)14-13-4-2-12(3-5-13)10-15-6-8-17-9-7-15/h2-5H,6-10H2,1H3,(H,14,16)
**509**	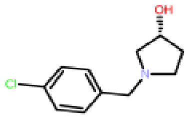	n.a.	415946-61-1	Z2856434858	bmse011628	InChI=1S/C11H14ClNO/c12-10-3-1-9(2-4-10)7-13-6-5-11(14)8-13/h1-4,11,14H,5-8H2/t11-/m1/s1
**510**	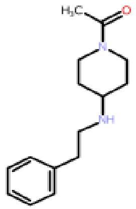	n.a.	415948-37-7	Z2856434883	bmse011657	InChI=1S/C15H22N2O/c1-13(18)17-11-8-15(9-12-17)16-10-7-14-5-3-2-4-6-14/h2-6,15-16H,7-12H2,1H3

Suppl Table 2: Details of the 768 compounds from the DSI-Poised fragment Library (DSI-PL)

Sl. No.	Structures	No. in Manuscript	CAS Registry Number	"ID"	^{1}H NMR Data Repository BMRB ID	ALATIS InChI
**511**	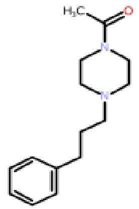	n.a.	415955-32-7	Z2856434884	bmse011663	InChI=1S/C15H22N2O/c1-14(18)17-12-10-16(11-13-17)9-5-8-15-6-3-2-4-7-15/h2-4,6-7H,5,8-13H2,1H3
**512**	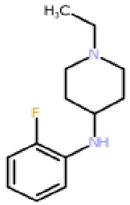	n.a.	416868-42-3	Z2856434868	bmse011672	InChI=1S/C13H19FN2/c1-2-16-9-7-11(8-10-16)15-13-6-4-3-5-12(13)14/h3-6,11,15H,2,7-10H2,1H3
**513**	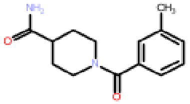	n.a.	418777-87-4	Z32400357	bmse011311	InChI=1S/C14H18N2O2/c1-10-3-2-4-12(9-10)14(18)16-7-5-11(6-8-16)13(15)17/h2-4,9,11H,5-8H2,1H3,(H2,15,17)
**514**	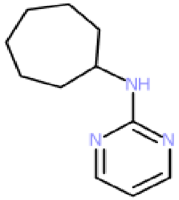	n.a.	419557-01-0	Z31244728	bmse011211	InChI=1S/C11H17N3/c1-2-4-7-10(6-3-1)14-11-12-8-5-9-13-11/h5,8-10H,1-4,6-7H2,(H,12,13,14)
**515**	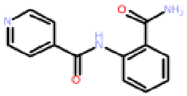	n.a.	419557-85-0	Z91797745	bmse011043	InChI=1S/C13H11N3O2/c14-12(17)10-3-1-2-4-11(10)16-13(18)9-5-7-15-8-6-9/h1-8H,(H2,14,17)(H,16,18)

Suppl Table 2: Details of the 768 compounds from the DSI-Poised fragment Library (DSI-PL)

Sl. No.	Structures	No. in Manuscript	CAS Registry Number	"ID"	^{1}H NMR Data Repository BMRB ID	ALATIS InChI
**516**	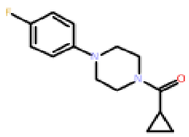	n.a.	419560-24-0	Z30620520	bmse011656	InChI=1S/C14H17FN2O/c15-12-3-5-13(6-4-12)16-7-9-17(10-8-16)14(18)11-1-2-11/h3-6,11H,1-2,7-10H2
**517**	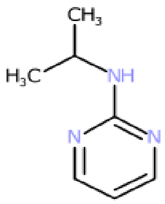	n.a.	4214-72-6	Z31190928	bmse011591	InChI=1S/C7H11N3/c1-6(2)10-7-8-4-3-5-9-7/h3-6H,1-2H3,(H,8,9,10)
**518**	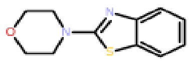	n.a.	4225-26-7	Z56767623	bmse011051	InChI=1S/C11H12N2OS/c1-2-4-10-9(3-1)12-11(15-10)13-5-7-14-8-6-13/h1-4H,5-8H2
**519**	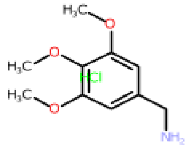	n.a.	42365-69-5	Z1741959530	bmse011445	InChI=1S/C10H15NO3.ClH/c1-12-8-4-7(6-11)5-9(13-2)10(8)14-3;/h4-5H,6,11H2,1-3H3;1H
**520**	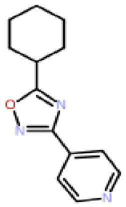	n.a.	423725-92-2	Z57715447	bmse011329	InChI=1S/C13H15N3O/c1-2-4-11(5-3-1)13-15-12(16-17-13)10-6-8-14-9-7-10/h6-9,11H,1-5H2

Suppl Table 2: Details of the 768 compounds from the DSI-Poised fragment Library (DSI-PL)

Sl. No.	Structures	No. in Manuscript	CAS Registry Number	"ID"	^{1}H NMR Data Repository BMRB ID	ALATIS InChI
**521**	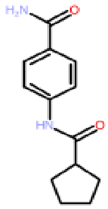	n.a.	424808-39-9	Z30242133	bmse011003	InChI=1S/C13H16N2O2/c14-12(16)9-5-7-11(8-6-9)15-13(17)10-3-1-2-4-10/h5-8,10H,1-4H2,(H2,14,16)(H,15,17)
**522**	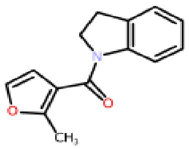	n.a.	424818-17-7	Z89307993	bmse011315	InChI=1S/C14H13NO2/c1-10-12(7-9-17-10)14(16)15-8-6-11-4-2-3-5-13(11)15/h2-5,7,9H,6,8H2,1H3
**523**	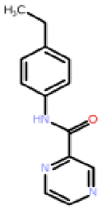	n.a.	424818-79-1	Z30917949	bmse011004	InChI=1S/C13H13N3O/c1-2-10-3-5-11(6-4-10)16-13(17)12-9-14-7-8-15-12/h3-9H,2H2,1H3,(H,16,17)
**524**	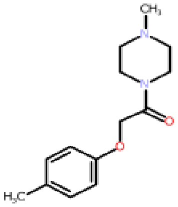	n.a.	426235-15-6	Z2856434836	bmse011658	InChI=1S/C14H20N2O2/c1-12-3-5-13(6-4-12)18-11-14(17)16-9-7-15(2)8-10-16/h3-6H,7-11H2,1-2H3
**525**	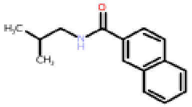	n.a.	429627-93-0	Z32385991	bmse011327	InChI=1S/C15H17NO/c1-11(2)10-16-15(17)14-8-7-12-5-3-4-6-13(12)9-14/h3-9,11H,10H2,1-2H3,(H,16,17)

Suppl Table 2: Details of the 768 compounds from the DSI-Poised fragment Library (DSI-PL)

Sl. No.	Structures	No. in Manuscript	CAS Registry Number	"ID"	^{1}H NMR Data Repository BMRB ID	ALATIS InChI
**526**	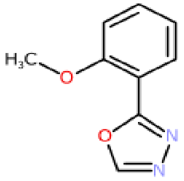	n.a.	42966-95-0	Z1359419878	bmse011395	InChI=1S/C9H8N2O2/c1-12-8-5-3-2-4-7(8)9-11-10-6-13-9/h2-6H,1H3
**527**	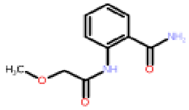	n.a.	430465-88-6	Z57260539	bmse011348	InChI=1S/C10H12N2O3/c1-15-6-9(13)12-8-5-3-2-4-7(8)10(11)14/h2-5H,6H2,1H3,(H2,11,14)(H,12,13)
**528**	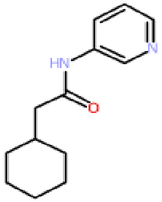	n.a.	431907-04-9	Z31792168	bmse011312	InChI=1S/C13H18N2O/c16-13(9-11-5-2-1-3-6-11)15-12-7-4-8-14-10-12/h4,7-8,10-11H,1-3,5-6,9H2,(H,15,16)
**529**	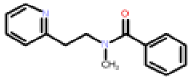	n.a.	431940-21-5	Z68277692	bmse011667	InChI=1S/C15H16N2O/c1-17(12-10-14-9-5-6-11-16-14)15(18)13-7-3-2-4-8-13/h2-9,11H,10,12H2,1H3
**530**	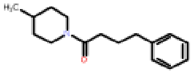	n.a.	432494-63-8	Z419884046	bmse011664	InChI=1S/C16H23NO/c1-14-10-12-17(13-11-14)16(18)9-5-8-15-6-3-2-4-7-15/h2-4,6-7,14H,5,8-13H2,1H3

Suppl Table 2: Details of the 768 compounds from the DSI-Poised fragment Library (DSI-PL)

Sl. No.	Structures	No. in Manuscript	CAS Registry Number	"ID"	^{1}H NMR Data Repository BMRB ID	ALATIS InChI
**531**	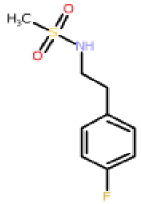	n.a.	432529-74-3	Z45705015	bmse011165	InChI=1S/C9H12FNO2S/c1-14(12,13)11-7-6-8-2-4-9(10)5-3-8/h2-5,11H,6-7H2,1H3
**532**	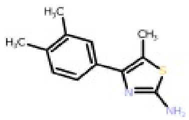	n.a.	438215-91-9	Z48847633	bmse011440	InChI=1S/C12H14N2S/c1-7-4-5-10(6-8(7)2)11-9(3)15-12(13)14-11/h4-6H,1-3H3,(H2,13,14)
**533**	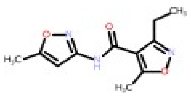	n.a.	438224-38-5	Z117233350	bmse011712	InChI=1S/C11H13N3O3/c1-4-8-10(7(3)17-13-8)11(15)12-9-5-6(2)16-14-9/h5H,4H2,1-3H3,(H,12,14,15)
**534**	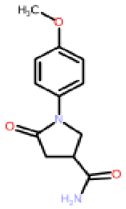	n.a.	443638-17-3	Z57324924	bmse011766	InChI=1S/C12H14N2O3/c1-17-10-4-2-9(3-5-10)14-7-8(12(13)16)6-11(14)15/h2-5,8H,6-7H2,1H3,(H2,13,16)/t8-/m1/s1
**535**	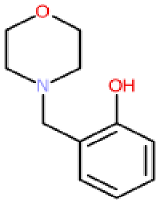	n.a.	4438-01-1	Z2856434887	bmse011362	InChI=1S/C11H15NO2/c13-11-4-2-1-3-10(11)9-12-5-7-14-8-6-12/h1-4,13H,5-9H2

Suppl Table 2: Details of the 768 compounds from the DSI-Poised fragment Library (DSI-PL)

Sl. No.	Structures	No. in Manuscript	CAS Registry Number	"ID"	1H NMR Data Repository BMRB ID	ALATIS InChI
**536**	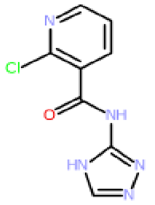	n.a.	444938-07-2	Z275154304	bmse011714	InChI=1S/C8H6ClN5O/c9-6-5(2-1-3-10-6)7(15)13-8-11-4-12-14-8/h1-4H,(H2,11,12,13,14,15)
**537**	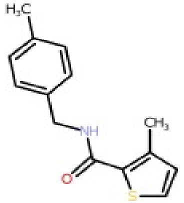	n.a.	445007-73-8	Z28429425	bmse011695	InChI=1S/C14H15NOS/c1-10-3-5-12(6-4-10)9-15-14(16)13-11(2)7-8-17-13/h3-8H,9H2,1-2H3,(H,15,16)
**538**	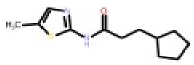	n.a.	445232-08-6	Z29325421	bmse011722	InChI=1S/C12H18N2OS/c1-9-8-13-12(16-9)14-11(15)7-6-10-4-2-3-5-10/h8,10H,2-7H2,1H3,(H,13,14,15)
**539**	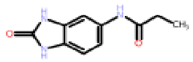	n.a.	445410-25-3	Z26794351	bmse011216	InChI=1S/C10H11N3O2/c1-2-9(14)11-6-3-4-7-8(5-6)13-10(15)12-7/h3-5H,2H2,1H3,(H,11,14)(H2,12,13,15)
**540**	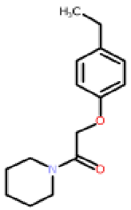	n.a.	448226-41-3	Z18839017	bmse011652	InChI=1S/C15H21NO2/c1-2-13-6-8-14(9-7-13)18-12-15(17)16-10-4-3-5-11-16/h6-9H,2-5,10-12H2,1H3

Suppl Table 2: Details of the 768 compounds from the DSI-Poised fragment Library (DSI-PL)

Sl. No.	Structures	No. in Manuscript	CAS Registry Number	"ID"	^{1}H NMR Data Repository BMRB ID	ALATIS InChI
**541**	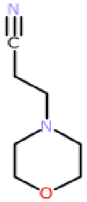	n.a.	4542-47-6	Z2856434767	bmse011738	InChI=1S/C7H12N2O/c8-2-1-3-9-4-6-10-7-5-9/h1,3-7H2
**542**	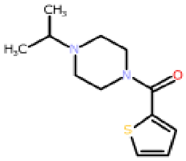	n.a.	461409-27-8	Z2856434826	bmse011702	InChI=1S/C12H18N2OS/c1-10(2)13-5-7-14(8-6-13)12(15)11-4-3-9-16-11/h3-4,9-10H,5-8H2,1-2H3
**543**	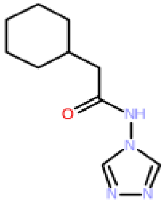	n.a.	462622-06-6	Z57261895	bmse011306	InChI=1S/C10H16N4O/c15-10(13-14-7-11-12-8-14)6-9-4-2-1-3-5-9/h7-9H,1-6H2,(H,13,15)
**544**	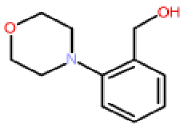	n.a.	465514-33-4	Z235361235	bmse011458	InChI=1S/C11H15NO2/c13-9-10-3-1-2-4-11(10)12-5-7-14-8-6-12/h1-4,13H,5-9H2
**545**	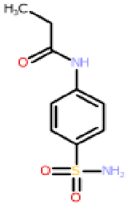	n.a.	4708-37-6	Z27682767	bmse011242	InChI=1S/C9H12N2O3S/c1-2-9(12)11-7-3-5-8(6-4-7)15(10,13)14/h3-6H,2H2,1H3,(H,11,12)(H2,10,13,14)

Suppl Table 2: Details of the 768 compounds from the DSI-Poised fragment Library (DSI-PL)

Sl. No.	Structures	No. in Manuscript	CAS Registry Number	"ID"	^{1}H NMR Data Repository BMRB ID	ALATIS InChI
**546**	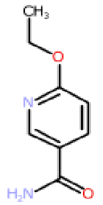	n.a.	473693-84-4	Z458894644	bmse011195	InChI=1S/C8H10N2O2/c1-2-12-7-4-3-6(5-10-7)8(9)11/h3-5H,2H2,1H3,(H2,9,11)
**547**	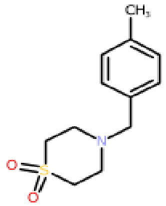	n.a.	477858-35-8	Z2856434929	bmse011318	InChI=1S/C12H17NO2S/c1-11-2-4-12(5-3-11)10-13-6-8-16(14,15)9-7-13/h2-5H,6-10H2,1H3
**548**	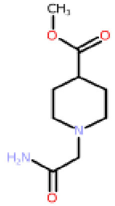	n.a.	477862-08-1	Z2856434804	bmse011293	InChI=1S/C9H16N2O3/c1-14-9(13)7-2-4-11(5-3-7)6-8(10)12/h7H,2-6H2,1H3,(H2,10,12)
**549**	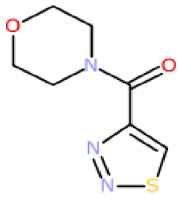	n.a.	478261-73-3	Z741055844	bmse011342	InChI=1S/C7H9N3O2S/c11-7(6-5-13-9-8-6)10-1-3-12-4-2-10/h5H,1-4H2
**550**	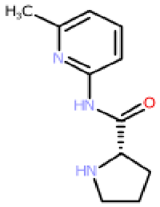	n.a.	478912-51-5	Z1267881672	bmse011284	InChI=1S/C11H15N3O/c1-8-4-2-6-10(13-8)14-11(15)9-5-3-7-12-9/h2,4,6,9,12H,3,5,7H2,1H3,(H,13,14,15)/t9-/m0/s1

Suppl Table 2: Details of the 768 compounds from the DSI-Poised fragment Library (DSI-PL)

Sl. No.	Structures	No. in Manuscript	CAS Registry Number	"ID"	^{1}H NMR Data Repository BMRB ID	ALATIS InChI
**551**	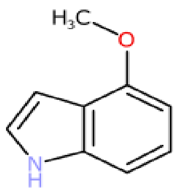	n.a.	4837-90-5	Z1429867185	bmse011151	InChI=1S/C9H9NO/c1-11-9-4-2-3-8-7(9)5-6-10-8/h2-6,10H,1H3
**552**	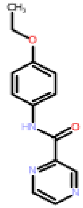	n.a.	484038-99-5	Z27808049	bmse011007	InChI=1S/C13H13N3O2/c1-2-18-11-5-3-10(4-6-11)16-13(17)12-9-14-7-8-15-12/h3-9H,2H2,1H3,(H,16,17)
**553**	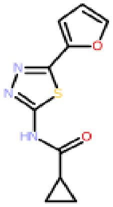	n.a.	484658-74-4	Z165141116	bmse011149	InChI=1S/C10H9N3O2S/c14-8(6-3-4-6)11-10-13-12-9(16-10)7-2-1-5-15-7/h1-2,5-6H,3-4H2,(H,11,13,14)
**554**	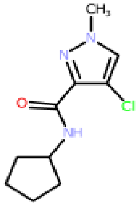	n.a.	489406-95-3	Z275151340	bmse011686	InChI=1S/C10H14ClN3O/c1-14-6-8(11)9(13-14)10(15)12-7-4-2-3-5-7/h6-7H,2-5H2,1H3,(H,12,15)
**555**	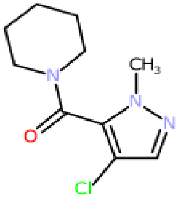	n.a.	491828-56-9	Z275179946	bmse011726	InChI=1S/C10H14ClN3O/c1-13-9(8(11)7-12-13)10(15)14-5-3-2-4-6-14/h7H,2-6H2,1H3

Suppl Table 2: Details of the 768 compounds from the DSI-Poised fragment Library (DSI-PL)

Sl. No.	Structures	No. in Manuscript	CAS Registry Number	"ID"	^{1}H NMR Data Repository BMRB ID	ALATIS InChI
**556**	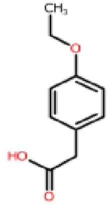	n.a.	4919-33-9	Z2856434918	bmse011437	InChI=1S/C10H12O3/c1-2-13-9-5-3-8(4-6-9)7-10(11)12/h3-6H,2,7H2,1H3,(H,11,12)
**557**	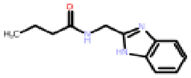	n.a.	497230-46-3	Z26781952	bmse011009	InChI=1S/C12H15N3O/c1-2-5-12(16)13-8-11-14-9-6-3-4-7-10(9)15-11/h3-4,6-7H,2,5,8H2,1H3,(H,13,16)(H,14,15)
**558**	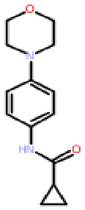	n.a.	499112-31-1	Z30485868	bmse011734	InChI=1S/C14H18N2O2/c17-14(11-1-2-11)15-12-3-5-13(6-4-12)16-7-9-18-10-8-16/h3-6,11H,1-2,7-10H2,(H,15,17)
**559**	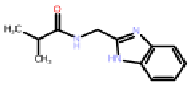	n.a.	500269-41-0	Z26781943	bmse011429	InChI=1S/C12H15N3O/c1-8(2)12(16)13-7-11-14-9-5-3-4-6-10(9)15-11/h3-6,8H,7H2,1-2H3,(H,13,16)(H,14,15)
**560**	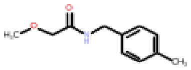	n.a.	503563-82-4	Z33297786	bmse011581	InChI=1S/C11H15NO2/c1-9-3-5-10(6-4-9)7-12-11(13)8-14-2/h3-6H,7-8H2,1-2H3,(H,12,13)

Suppl Table 2: Details of the 768 compounds from the DSI-Poised fragment Library (DSI-PL)

Sl. No.	Structures	No. in Manuscript	CAS Registry Number	"ID"	^{1}H NMR Data Repository BMRB ID	ALATIS InChI
**561**	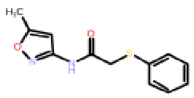	n.a.	505088-70-0	Z19750454	bmse011034	InChI=1S/C12H12N2O2S/c1-9-7-11(14-16-9)13-12(15)8-17-10-5-3-2-4-6-10/h2-7H,8H2,1H3,(H,13,14,15)
**562**	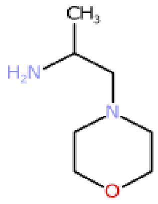	n.a.	50998-05-5	Z818732104	bmse011549	InChI=1S/C7H16N2O/c1-7(8)6-9-2-4-10-5-3-9/h7H,2-6,8H2,1H3/t7-/m0/s1
**563**	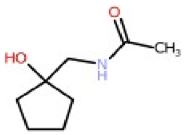	n.a.	51004-22-9	Z1203329531	bmse011129	InChI=1S/C8H15NO2/c1-7(10)9-6-8(11)4-2-3-5-8/h11H,2-6H2,1H3,(H,9,10)
**564**	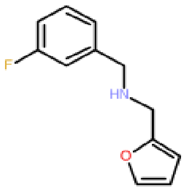	n.a.	510723-71-4	Z2737076969	bmse011689	InChI=1S/C12H12FNO/c13-11-4-1-3-10(7-11)8-14-9-12-5-2-6-15-12/h1-7,14H,8-9H2
**565**	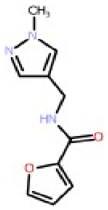	n.a.	514843-64-2	Z275179758	bmse011724	InChI=1S/C10H11N3O2/c1-13-7-8(6-12-13)5-11-10(14)9-3-2-4-15-9/h2-4,6-7H,5H2,1H3,(H,11,14)

Suppl Table 2: Details of the 768 compounds from the DSI-Poised fragment Library (DSI-PL)

Sl. No.	Structures	No. in Manuscript	CAS Registry Number	"ID"	^{1}H NMR Data Repository BMRB ID	ALATIS InChI
**566**	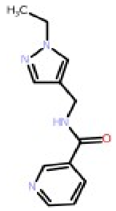	n.a.	514856-39-4	Z275181224	bmse011626	InChI=1S/C12H14N4O/c1-2-16-9-10(7-15-16)6-14-12(17)11-4-3-5-13-8-11/h3-5,7-9H,2,6H2,1H3,(H,14,17)
**567**	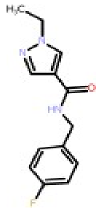	n.a.	515831-20-6	Z1203107138	bmse011699	InChI=1S/C13H14FN3O/c1-2-17-9-11(8-16-17)13(18)15-7-10-3-5-12(14)6-4-10/h3-6,8-9H,2,7H2,1H3,(H,15,18)
**568**	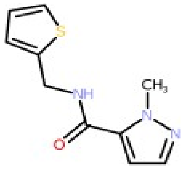	n.a.	515847-82-2	Z915492990	bmse011683	InChI=1S/C10H11N3OS/c1-13-9(4-5-12-13)10(14)11-7-8-3-2-6-15-8/h2-6H,7H2,1H3,(H,11,14)
**569**	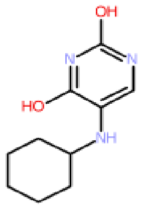	n.a.	5177-53-7	Z57283017	bmse011639	InChI=1S/C10H15N3O2/c14-9-8(6-11-10(15)13-9)12-7-4-2-1-3-5-7/h6-7,12H,1-5H2,(H2,11,13,14,15)
**570**	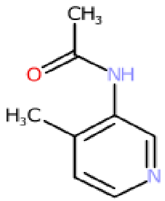	n.a.	52090-68-3	Z1129283193	bmse011158	InChI=1S/C8H10N2O/c1-6-3-4-9-5-8(6)10-7(2)11/h3-5H,1-2H3,(H,10,11)

Suppl Table 2: Details of the 768 compounds from the DSI-Poised fragment Library (DSI-PL)

Sl. No.	Structures	No. in Manuscript	CAS Registry Number	"ID"	^{1}H NMR Data Repository BMRB ID	ALATIS InChI
571	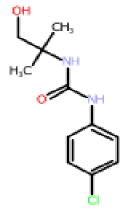	n.a.	522660-63-5	Z123970702	bmse011320	InChI=1S/C11H15ClN2O2/c1-11(2,7-15)14-10(16)13-9-5-3-8(12)4-6-9/h3-6,15H,7H2,1-2H3,(H2,13,14,16)
572	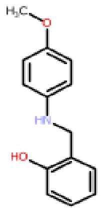	n.a.	52537-88-9	Z56943440	bmse011439	InChI=1S/C14H15NO2/c1-17-13-8-6-12(7-9-13)15-10-11-4-2-3-5-14(11)16/h2-9,15-16H,10H2,1H3
573	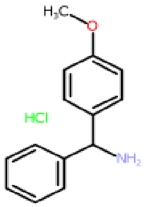	n.a.	5267-46-9	Z2574937229	bmse011751	InChI=1S/C14H15NO.ClH/c1-16-13-9-7-12(8-10-13)14(15)11-5-3-2-4-6-11;/h2-10,14H,15H2,1H3;1H/t14-;/m1./s1
574	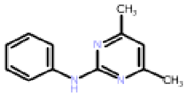	n.a.	53112-28-0	Z285675722	bmse011367	InChI=1S/C12H13N3/c1-9-8-10(2)14-12(13-9)15-11-6-4-3-5-7-11/h3-8H,1-2H3,(H,13,14,15)
575	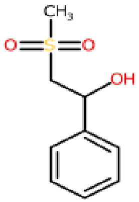	n.a.	5324-56-1	Z822382694	bmse011260	InChI=1S/C9H12O3S/c1-13(11,12)7-9(10)8-5-3-2-4-6-8/h2-6,9-10H,7H2,1H3/t9-/m0/s1

Suppl Table 2: Details of the 768 compounds from the DSI-Poised fragment Library (DSI-PL)

Sl. No.	Structures	No. in Manuscript	CAS Registry Number	"ID"	^{1}H NMR Data Repository BMRB ID	ALATIS InChI
**576**	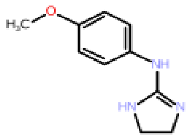	n.a.	53976-95-7	Z57186564	bmse011100	InChI=1S/C10H13N3O/c1-14-9-4-2-8(3-5-9)13-10-11-6-7-12-10/h2-5H,6-7H2,1H3,(H2,11,12,13)
**577**	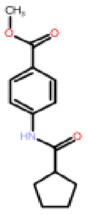	n.a.	540531-66-6	Z28226359	bmse011066	InChI=1S/C14H17NO3/c1-18-14(17)11-6-8-12(9-7-11)15-13(16)10-4-2-3-5-10/h6-10H,2-5H2,1H3,(H,15,16)
**578**	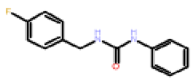	n.a.	541545-68-0	Z44609285	bmse011292	InChI=1S/C14H13FN2O/c15-12-8-6-11(7-9-12)10-16-14(18)17-13-4-2-1-3-5-13/h1-9H,10H2,(H2,16,17,18)
**579**	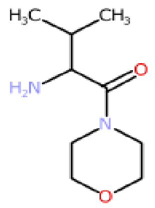	n.a.	54164-04-4	Z927400026	bmse011596	InChI=1S/C9H18N2O2/c1-7(2)8(10)9(12)11-3-5-13-6-4-11/h7-8H,3-6,10H2,1-2H3/t8-/m0/s1
**580**	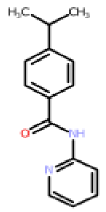	n.a.	543706-26-9	Z85525355	bmse011365	InChI=1S/C15H16N2O/c1-11(2)12-6-8-13(9-7-12)15(18)17-14-5-3-4-10-16-14/h3-11H,1-2H3,(H,16,17,18)

Suppl Table 2: Details of the 768 compounds from the DSI-Poised fragment Library (DSI-PL)

Sl. No.	Structures	No. in Manuscript	CAS Registry Number	"ID"	^{1}H NMR Data Repository BMRB ID	ALATIS InChI
**581**	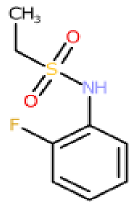	n.a.	544662-80-8	Z53825177	bmse011408	InChI=1S/C8H10FNO2S/c1-2-13(11,12)10-8-6-4-3-5-7(8)9/h3-6,10H,2H2,1H3
**582**	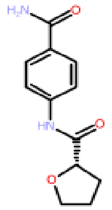	n.a.	544668-49-7	Z1545313172	bmse011346	InChI=1S/C12H14N2O3/c13-11(15)8-3-5-9(6-4-8)14-12(16)10-2-1-7-17-10/h3-6,10H,1-2,7H2,(H2,13,15)(H,14,16)/t10-/m0/s1
**583**	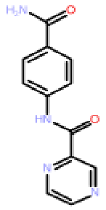	n.a.	544670-58-8	Z30242120	bmse011017	InChI=1S/C12H10N4O2/c13-11(17)8-1-3-9(4-2-8)16-12(18)10-7-14-5-6-15-10/h1-7H,(H2,13,17)(H,16,18)
**584**	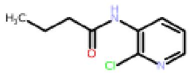	n.a.	546070-44-4	Z240654968	bmse011665	InChI=1S/C9H11ClN2O/c1-2-4-8(13)12-7-5-3-6-11-9(7)10/h3,5-6H,2,4H2,1H3,(H,12,13)
**585**	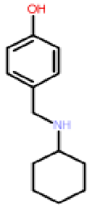	n.a.	5461-17-6	Z2241127906	bmse011246	InChI=1S/C13H19NO/c15-13-8-6-11(7-9-13)10-14-12-4-2-1-3-5-12/h6-9,12,14-15H,1-5,10H2

Suppl Table 2: Details of the 768 compounds from the DSI-Poised fragment Library (DSI-PL)

Sl. No.	Structures	No. in Manuscript	CAS Registry Number	"ID"	1H NMR Data Repository BMRB ID	ALATIS InChI
**586**	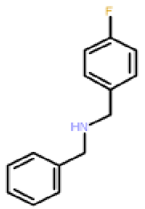	n.a.	55096-88-3	Z2856434783	bmse011753	InChI=1S/C14H14FN/c15-14-8-6-13(7-9-14)11-16-10-12-4-2-1-3-5-12/h1-9,16H,10-11H2
**587**	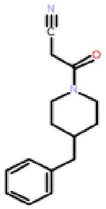	n.a.	551907-33-6	Z1688493887	bmse011691	InChI=1S/C15H18N2O/c16-9-6-15(18)17-10-7-14(8-11-17)12-13-4-2-1-3-5-13/h1-5,14H,6-8,10-12H2
**588**	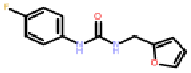	n.a.	551931-68-1	Z44584202	bmse011026	InChI=1S/C12H11FN2O2/c13-9-3-5-10(6-4-9)15-12(16)14-8-11-2-1-7-17-11/h1-7H,8H2,(H2,14,15,16)
**589**	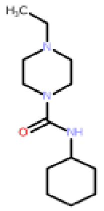	n.a.	553657-03-7	Z2856434830	bmse011671	InChI=1S/C13H25N3O/c1-2-15-8-10-16(11-9-15)13(17)14-12-6-4-3-5-7-12/h12H,2-11H2,1H3,(H,14,17)
**590**	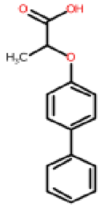	n.a.	5555-13-5	Z111782404	bmse011073	InChI=1S/C15H14O3/c1-11(15(16)17)18-14-9-7-13(8-10-14)12-5-3-2-4-6-12/h2-11H,1H3,(H,16,17)/t11-/m0/s1

Suppl Table 2: Details of the 768 compounds from the DSI-Poised fragment Library (DSI-PL)

Sl. No.	Structures	No. in Manuscript	CAS Registry Number	"ID"	^{1}H NMR Data Repository BMRB ID	ALATIS InChI
**591**	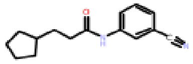	n.a.	556805-44-8	Z26548228	bmse011081	InChI=1S/C15H18N2O/c16-11-13-6-3-7-14(10-13)17-15(18)9-8-12-4-1-2-5-12/h3,6-7,10,12H,1-2,4-5,8-9H2,(H,17,18)
**592**	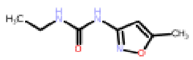	n.a.	55807-55-1	Z56880342	bmse011089	InChI=1S/C7H11N3O2/c1-3-8-7(11)9-6-4-5(2)12-10-6/h4H,3H2,1-2H3,(H2,8,9,10,11)
**593**	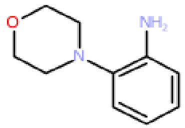	n.a.	5585-33-1	Z56785490	bmse011376	InChI=1S/C10H14N2O/c11-9-3-1-2-4-10(9)12-5-7-13-8-6-12/h1-4H,5-8,11H2
**594**	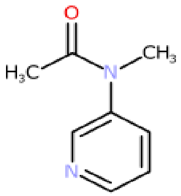	n.a.	55899-01-9	Z1203252645	bmse011598	InChI=1S/C8H10N2O/c1-7(11)10(2)8-4-3-5-9-6-8/h3-6H,1-2H3
**595**	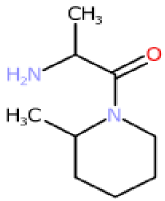	n.a.	56414-90-5	Z927412236	bmse011488	InChI=1S/C9H18N2O/c1-7-5-3-4-6-11(7)9(12)8(2)10/h7-8H,3-6,10H2,1-2H3/t7-,8-/m0/s1

Suppl Table 2: Details of the 768 compounds from the DSI-Poised fragment Library (DSI-PL)

Sl. No.	Structures	No. in Manuscript	CAS Registry Number	"ID"	^{1}H NMR Data Repository BMRB ID	ALATIS InChI
**596**	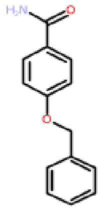	n.a.	56442-43-4	Z26312102	bmse011205	InChI=1S/C14H13NO2/c15-14(16)12-6-8-13(9-7-12)17-10-11-4-2-1-3-5-11/h1-9H,10H2,(H2,15,16)
**597**	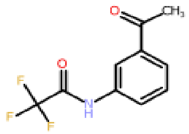	n.a.	56915-87-8	Z57111868	bmse011190	InChI=1S/C10H8F3NO2/c1-6(15)7-3-2-4-8(5-7)14-9(16)10(11,12)13/h2-5H,1H3,(H,14,16)
**598**	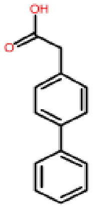	n.a.	5728-52-9	Z2856434779	bmse011053	InChI=1S/C14H12O2/c15-14(16)10-11-6-8-13(9-7-11)12-4-2-1-3-5-12/h1-9H,10H2,(H,15,16)
**599**	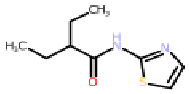	n.a.	574011-44-2	Z28173817	bmse011322	InChI=1S/C9H14N2OS/c1-3-7(4-2)8(12)11-9-10-5-6-13-9/h5-7H,3-4H2,1-2H3,(H,10,11,12)
**600**	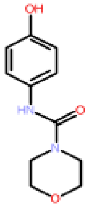	n.a.	57726-21-3	Z274554872	bmse011390	InChI=1S/C11H14N2O3/c14-10-3-1-9(2-4-10)12-11(15)13-5-7-16-8-6-13/h1-4,14H,5-8H2,(H,12,15)

Suppl Table 2: Details of the 768 compounds from the DSI-Poised fragment Library (DSI-PL)

Sl. No.	Structures	No. in Manuscript	CAS Registry Number	"ID"	^{1}H NMR Data Repository BMRB ID	ALATIS InChI
**601**	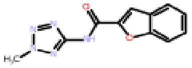	n.a.	577980-81-5	Z57292378	bmse011697	InChI=1S/C11H9N5O2/c1-16-14-11(13-15-16)12-10(17)9-6-7-4-2-3-5-8(7)18-9/h2-6H,1H3,(H,12,14,17)
**602**	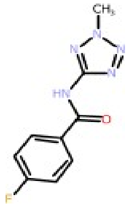	n.a.	578746-24-4	Z57292433	bmse011732	InChI=1S/C9H8FN5O/c1-15-13-9(12-14-15)11-8(16)6-2-4-7(10)5-3-6/h2-5H,1H3,(H,11,13,16)
**603**	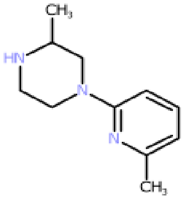	n.a.	582325-37-9	Z1267885772	bmse011521	InChI=1S/C11H17N3/c1-9-4-3-5-11(13-9)14-7-6-12-10(2)8-14/h3-5,10,12H,6-8H2,1-2H3/t10-/m1/s1
**604**	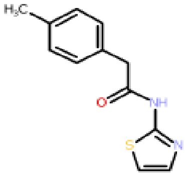	n.a.	587843-69-4	Z90713255	bmse011042	InChI=1S/C12H12N2OS/c1-9-2-4-10(5-3-9)8-11(15)14-12-13-6-7-16-12/h2-7H,8H2,1H3,(H,13,14,15)
**605**	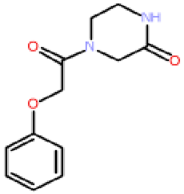	n.a.	587850-66-6	Z31602870	bmse011351	InChI=1S/C12H14N2O3/c15-11-8-14(7-6-13-11)12(16)9-17-10-4-2-1-3-5-10/h1-5H,6-9H2,(H,13,15)

Suppl Table 2: Details of the 768 compounds from the DSI-Poised fragment Library (DSI-PL)

Sl. No.	Structures	No. in Manuscript	CAS Registry Number	"ID"	^{1}H NMR Data Repository BMRB ID	ALATIS InChI
**606**	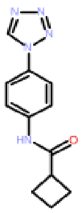	n.a.	606096-31-5	Z26552420	bmse011444	InChI=1S/C12H13N5O/c18-12(9-2-1-3-9)14-10-4-6-11(7-5-10)17-8-13-15-16-17/h4-9H,1-3H2,(H,14,18)
**607**	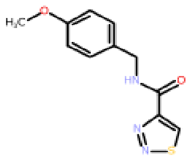	n.a.	606102-02-7	Z741218268	bmse011134	InChI=1S/C11H11N3O2S/c1-16-9-4-2-8(3-5-9)6-12-11(15)10-7-17-14-13-10/h2-5,7H,6H2,1H3,(H,12,15)
**608**	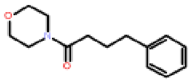	n.a.	61123-44-2	Z419995480	bmse011504	InChI=1S/C14H19NO2/c16-14(15-9-11-17-12-10-15)8-4-7-13-5-2-1-3-6-13/h1-3,5-6H,4,7-12H2
**609**	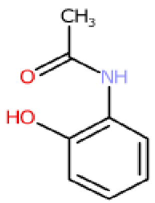	n.a.	614-80-2	Z57040482	bmse011002	InChI=1S/C8H9NO2/c1-6(10)9-7-4-2-3-5-8(7)11/h2-5,11H,1H3,(H,9,10)
**610**	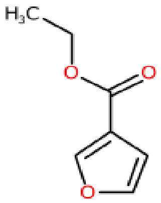	n.a.	614-98-2	Z293079056	bmse011642	InChI=1S/C7H8O3/c1-2-10-7(8)6-3-4-9-5-6/h3-5H,2H2,1H3

Suppl Table 2: Details of the 768 compounds from the DSI-Poised fragment Library (DSI-PL)

Sl. No.	Structures	No. in Manuscript	CAS Registry Number	"ID"	^{1}H NMR Data Repository BMRB ID	ALATIS InChI
**611**	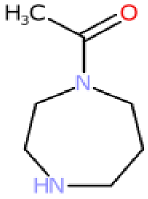	n.a.	61903-11-5	Z168883358	bmse011561	InChI=1S/C7H14N2O/c1-7(10)9-5-2-3-8-4-6-9/h8H,2-6H2,1H3
**612**	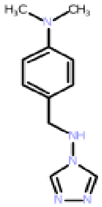	n.a.	6213-03-2	Z57258487	bmse011733	InChI=1S/C11H15N5/c1-15(2)11-5-3-10(4-6-11)7-14-16-8-12-13-9-16/h3-6,8-9,14H,7H2,1-2H3
**613**	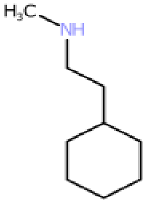	n.a.	62141-38-2	Z2856434901	bmse011576	InChI=1S/C9H19N/c1-10-8-7-9-5-3-2-4-6-9/h9-10H,2-8H2,1H3
**614**	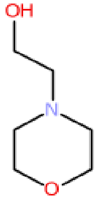	n.a.	622-40-2	Z2856434843	bmse011532	InChI=1S/C6H13NO2/c8-4-1-7-2-5-9-6-3-7/h8H,1-6H2
**615**	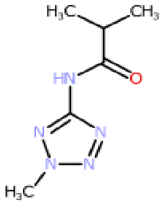	n.a.	62400-57-1	Z57292369	bmse011739	InChI=1S/C6H11N5O/c1-4(2)5(12)7-6-8-10-11(3)9-6/h4H,1-3H3,(H,7,9,12)

Suppl Table 2: Details of the 768 compounds from the DSI-Poised fragment Library (DSI-PL)

Sl. No.	Structures	No. in Manuscript	CAS Registry Number	"ID"	^{1}H NMR Data Repository BMRB ID	ALATIS InChI
**616**	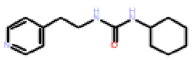	n.a.	626222-65-9	Z111507846	bmse011191	InChI=1S/C14H21N3O/c18-14(17-13-4-2-1-3-5-13)16-11-8-12-6-9-15-10-7-12/h6-7,9-10,13H,1-5,8,11H2,(H2,16,17,18)
**617**	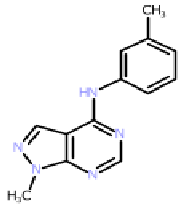	n.a.	6289-08-3	Z56983806	bmse011289	InChI=1S/C13H13N5/c1-9-4-3-5-10(6-9)17-12-11-7-16-18(2)13(11)15-8-14-12/h3-8H,1-2H3,(H,14,15,17)
**618**	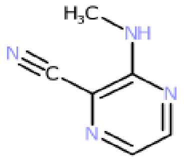	n.a.	63352-06-7	Z1896597864	bmse011138	InChI=1S/C6H6N4/c1-8-6-5(4-7)9-2-3-10-6/h2-3H,1H3,(H,8,10)
**619**	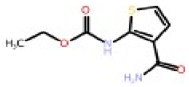	n.a.	63895-76-1	Z64450924	bmse011730	InChI=1S/C8H10N2O3S/c1-2-13-8(12)10-7-5(6(9)11)3-4-14-7/h3-4H,2H2,1H3,(H2,9,11)(H,10,12)
**620**	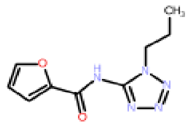	n.a.	639046-93-8	Z57446103	bmse011041	InChI=1S/C9H11N5O2/c1-2-5-14-9(11-12-13-14)10-8(15)7-4-3-6-16-7/h3-4,6H,2,5H2,1H3,(H,10,11,13,15)

Suppl Table 2: Details of the 768 compounds from the DSI-Poised fragment Library (DSI-PL)

Sl. No.	Structures	No. in Manuscript	CAS Registry Number	"ID"	^{1}H NMR Data Repository BMRB ID	ALATIS InChI
**621**	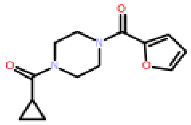	n.a.	6391-74-8	Z32327641	bmse011335	InChI=1S/C13H16N2O3/c16-12(10-3-4-10)14-5-7-15(8-6-14)13(17)11-2-1-9-18-11/h1-2,9-10H,3-8H2
**622**	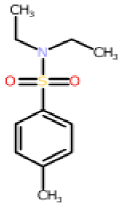	n.a.	649-15-0	Z45516134	bmse011413	InChI=1S/C11H17NO2S/c1-4-12(5-2)15(13,14)11-8-6-10(3)7-9-11/h6-9H,4-5H2,1-3H3
**623**	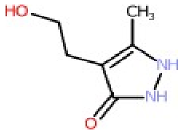	n.a.	65287-96-9	Z1259155959	bmse011263	InChI=1S/C6H10N2O2/c1-4-5(2-3-9)6(10)8-7-4/h9H,2-3H2,1H3,(H2,7,8,10)
**624**	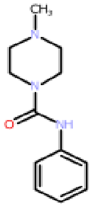	n.a.	65766-72-5	Z2856434890	bmse011340	InChI=1S/C12H17N3O/c1-14-7-9-15(10-8-14)12(16)13-11-5-3-2-4-6-11/h2-6H,7-10H2,1H3,(H,13,16)
**625**	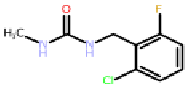	n.a.	660849-84-3	Z933840894	bmse011295	InChI=1S/C9H10ClFN2O/c1-12-9(14)13-5-6-7(10)3-2-4-8(6)11/h2-4H,5H2,1H3,(H2,12,13,14)

Suppl Table 2: Details of the 768 compounds from the DSI-Poised fragment Library (DSI-PL)

Sl. No.	Structures	No. in Manuscript	CAS Registry Number	"ID"	^{1}H NMR Data Repository BMRB ID	ALATIS InChI
**626**	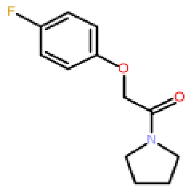	n.a.	667881-38-1	Z19735067	bmse011076	InChI=1S/C12H14FNO2/c13-10-3-5-11(6-4-10)16-9-12(15)14-7-1-2-8-14/h3-6H,1-2,7-9H2
**627**	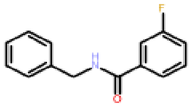	n.a.	671-01-2	Z27749656	bmse011316	InChI=1S/C14H12FNO/c15-13-8-4-7-12(9-13)14(17)16-10-11-5-2-1-3-6-11/h1-9H,10H2,(H,16,17)
**628**	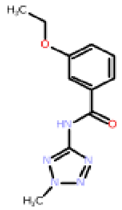	n.a.	673453-78-6	Z57292400	bmse011723	InChI=1S/C11H13N5O2/c1-3-18-9-6-4-5-8(7-9)10(17)12-11-13-15-16(2)14-11/h4-7H,3H2,1-2H3,(H,12,14,17)
**629**	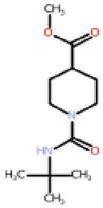	n.a.	675121-22-9	Z44567722	bmse011297	InChI=1S/C12H22N2O3/c1-12(2,3)13-11(16)14-7-5-9(6-8-14)10(15)17-4/h9H,5-8H2,1-4H3,(H,13,16)
**630**	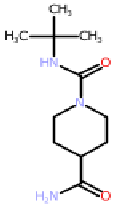	n.a.	676146-84-2	Z44603686	bmse011682	InChI=1S/C11H21N3O2/c1-11(2,3)13-10(16)14-6-4-8(5-7-14)9(12)15/h8H,4-7H2,1-3H3,(H2,12,15)(H,13,16)

Suppl Table 2: Details of the 768 compounds from the DSI-Poised fragment Library (DSI-PL)

Sl. No.	Structures	No. in Manuscript	CAS Registry Number	"ID"	^{1}H NMR Data Repository BMRB ID	ALATIS InChI
**631**	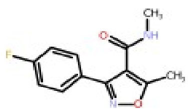	n.a.	67764-99-2	Z363104204	bmse011583	InChI=1S/C12H11FN2O2/c1-7-10(12(16)14-2)11(15-17-7)8-3-5-9(13)6-4-8/h3-6H,1-2H3,(H,14,16)
**632**	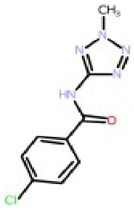	n.a.	679824-05-6	Z57292434	bmse011741	InChI=1S/C9H8ClN5O/c1-15-13-9(12-14-15)11-8(16)6-2-4-7(10)5-3-6/h2-5H,1H3,(H,11,13,16)
**633**	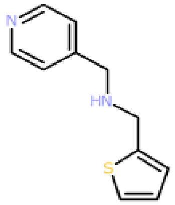	n.a.	680185-81-3	Z2856434816	bmse011761	InChI=1S/C11H12N2S/c1-2-11(14-7-1)9-13-8-10-3-5-12-6-4-10/h1-7,13H,8-9H2
**634**	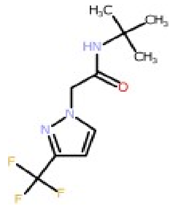	n.a.	680203-49-0	Z55009317	bmse011482	InChI=1S/C10H14F3N3O/c1-9(2,3)14-8(17)6-16-5-4-7(15-16)10(11,12)13/h4-5H,6H2,1-3H3,(H,14,17)
**635**	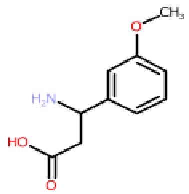	n.a.	68208-19-5	Z335451386	bmse011067	InChI=1S/C10H13NO3/c1-14-8-4-2-3-7(5-8)9(11)6-10(12)13/h2-5,9H,6,11H2,1H3,(H,12,13)/t9-/m0/s1

Suppl Table 2: Details of the 768 compounds from the DSI-Poised fragment Library (DSI-PL)

Sl. No.	Structures	No. in Manuscript	CAS Registry Number	"ID"	^{1}H NMR Data Repository BMRB ID	ALATIS InChI
**636**	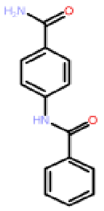	n.a.	68688-78-8	Z30242076	bmse011393	InChI=1S/C14H12N2O2/c15-13(17)10-6-8-12(9-7-10)16-14(18)11-4-2-1-3-5-11/h1-9H,(H2,15,17)(H,16,18)
**637**	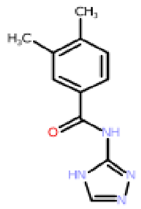	n.a.	690981-54-5	Z2856434827	bmse011179	InChI=1S/C11H12N4O/c1-7-3-4-9(5-8(7)2)10(16)14-11-12-6-13-15-11/h3-6H,1-2H3,(H2,12,13,14,15,16)
**638**	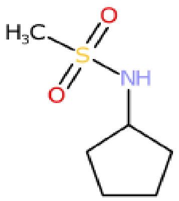	n.a.	69200-54-0	Z53825479	bmse011188	InChI=1S/C6H13NO2S/c1-10(8,9)7-6-4-2-3-5-6/h6-7H,2-5H2,1H3
**639**	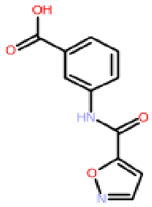	n.a.	693776-70-4	Z2856434931	bmse011637	InChI=1S/C11H8N2O4/c14-10(9-4-5-12-17-9)13-8-3-1-2-7(6-8)11(15)16/h1-6H,(H,13,14)(H,15,16)
**640**	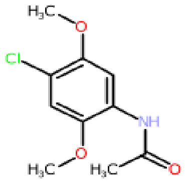	n.a.	6938-75-6	Z26968795	bmse011078	InChI=1S/C10H12ClNO3/c1-6(13)12-8-5-9(14-2)7(11)4-10(8)15-3/h4-5H,1-3H3,(H,12,13)

Suppl Table 2: Details of the 768 compounds from the DSI-Poised fragment Library (DSI-PL)

Sl. No.	Structures	No. in Manuscript	CAS Registry Number	"ID"	^{1}H NMR Data Repository BMRB ID	ALATIS InChI
641	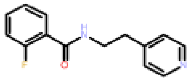	n.a.	696629-69-3	Z1310876699	bmse011330	InChI=1S/C14H13FN2O/c15-13-4-2-1-3-12(13)14(18)17-10-7-11-5-8-16-9-6-11/h1-6,8-9H,7,10H2,(H,17,18)
642	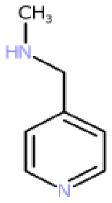	n.a.	6971-44-4	Z2856434786	bmse011735	InChI=1S/C7H10N2/c1-8-6-7-2-4-9-5-3-7/h2-5,8H,6H2,1H3
643	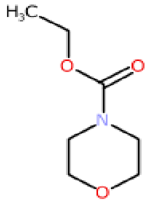	n.a.	6976-49-4	Z31721097	bmse011538	InChI=1S/C7H13NO3/c1-2-11-7(9)8-3-5-10-6-4-8/h2-6H2,1H3
644	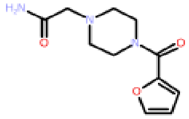	n.a.	697770-37-9	Z2856434793	bmse011181	InChI=1S/C11H15N3O3/c12-10(15)8-13-3-5-14(6-4-13)11(16)9-2-1-7-17-9/h1-2,7H,3-6,8H2,(H2,12,15)
645	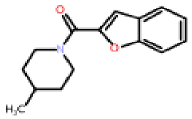	n.a.	701224-39-7	Z32665176	bmse011334	InChI=1S/C15H17NO2/c1-11-6-8-16(9-7-11)15(17)14-10-12-4-2-3-5-13(12)18-14/h2-5,10-11H,6-9H2,1H3

Suppl Table 2: Details of the 768 compounds from the DSI-Poised fragment Library (DSI-PL)

Sl. No.	Structures	No. in Manuscript	CAS Registry Number	"ID"	^{1}H NMR Data Repository BMRB ID	ALATIS InChI
**646**	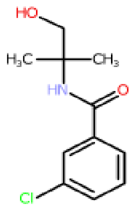	n.a.	701923-86-6	Z68299550	bmse011308	InChI=1S/C11H14ClNO2/c1-11(2,7-14)13-10(15)8-4-3-5-9(12)6-8/h3-6,14H,7H2,1-2H3,(H,13,15)
**647**	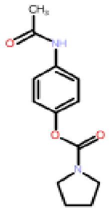	n.a.	701973-29-7	Z274555566	bmse011389	InChI=1S/C13H16N2O3/c1-10(16)14-11-4-6-12(7-5-11)18-13(17)15-8-2-3-9-15/h4-7H,2-3,8-9H2,1H3,(H,14,16)
**648**	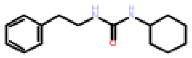	n.a.	70243-17-3	Z44585920	bmse011380	InChI=1S/C15H22N2O/c18-15(17-14-9-5-2-6-10-14)16-12-11-13-7-3-1-4-8-13/h1,3-4,7-8,14H,2,5-6,9-12H2,(H2,16,17,18)
**649**	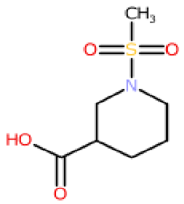	n.a.	702670-29-9	Z133632670	bmse011457	InChI=1S/C7H13NO4S/c1-13(11,12)8-4-2-3-6(5-8)7(9)10/h6H,2-5H2,1H3,(H,9,10)/t6-/m0/s1
**650**	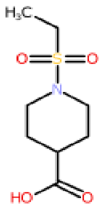	n.a.	702670-32-4	Z53825020	bmse011452	InChI=1S/C8H15NO4S/c1-2-14(12,13)9-5-3-7(4-6-9)8(10)11/h7H,2-6H2,1H3,(H,10,11)

Suppl Table 2: Details of the 768 compounds from the DSI-Poised fragment Library (DSI-PL)

Sl. No.	Structures	No. in Manuscript	CAS Registry Number	"ID"	^{1}H NMR Data Repository BMRB ID	ALATIS InChI
**651**	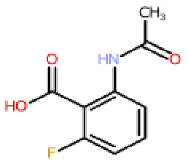	n.a.	70413-91-1	Z1229798311	bmse011265	InChI=1S/C9H8FNO3/c1-5(12)11-7-4-2-3-6(10)8(7)9(13)14/h2-4H,1H3,(H,11,12)(H,13,14)
**652**	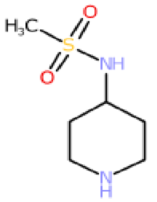	n.a.	70724-72-0	Z212045094	bmse011453	InChI=1S/C6H14N2O2S/c1-11(9,10)8-6-2-4-7-5-3-6/h6-8H,2-5H2,1H3
**653**	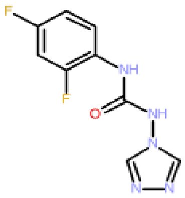	n.a.	708216-82-4	Z56259192	bmse011633	InChI=1S/C9H7F2N5O/c10-6-1-2-8(7(11)3-6)14-9(17)15-16-4-12-13-5-16/h1-5H,(H2,14,15,17)
**654**	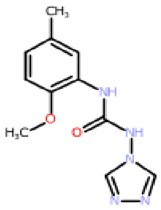	n.a.	708236-68-4	Z2234920345	bmse011731	InChI=1S/C11H13N5O2/c1-8-3-4-10(18-2)9(5-8)14-11(17)15-16-6-12-13-7-16/h3-7H,1-2H3,(H2,14,15,17)
**655**	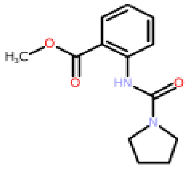	n.a.	708241-16-1	Z281077318	bmse011075	InChI=1S/C13H16N2O3/c1-18-12(16)10-6-2-3-7-11(10)14-13(17)15-8-4-5-9-15/h2-3,6-7H,4-5,8-9H2,1H3,(H,14,17)

Suppl Table 2: Details of the 768 compounds from the DSI-Poised fragment Library (DSI-PL)

Sl. No.	Structures	No. in Manuscript	CAS Registry Number	"ID"	^{1}H NMR Data Repository BMRB ID	ALATIS InChI
**656**	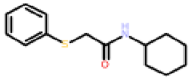	n.a.	71433-02-8	Z19751622	bmse011696	InChI=1S/C14H19NOS/c16-14(15-12-7-3-1-4-8-12)11-17-13-9-5-2-6-10-13/h2,5-6,9-10,12H,1,3-4,7-8,11H2,(H,15,16)
**657**	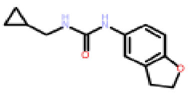	n.a.	717098-24-3	Z413792090	bmse011634	InChI=1S/C13H16N2O2/c16-13(14-8-9-1-2-9)15-11-3-4-12-10(7-11)5-6-17-12/h3-4,7,9H,1-2,5-6,8H2,(H2,14,15,16)
**658**	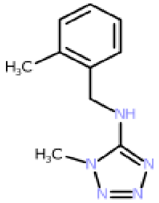	n.a.	717830-48-3	Z57778470	bmse011298	InChI=1S/C10H13N5/c1-8-5-3-4-6-9(8)7-11-10-12-13-14-15(10)2/h3-6H,7H2,1-2H3,(H,11,12,14)
**659**	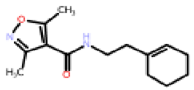	n.a.	717857-92-6	Z69092635	bmse011304	InChI=1S/C14H20N2O2/c1-10-13(11(2)18-16-10)14(17)15-9-8-12-6-4-3-5-7-12/h6H,3-5,7-9H2,1-2H3,(H,15,17)
**660**	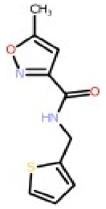	n.a.	717857-97-1	Z29692148	bmse011086	InChI=1S/C10H10N2O2S/c1-7-5-9(12-14-7)10(13)11-6-8-3-2-4-15-8/h2-5H,6H2,1H3,(H,11,13)

Suppl Table 2: Details of the 768 compounds from the DSI-Poised fragment Library (DSI-PL)

Sl. No.	Structures	No. in Manuscript	CAS Registry Number	"ID"	1H NMR Data Repository BMRB ID	ALATIS InChI
**661**	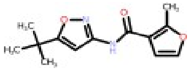	n.a.	717872-08-7	Z1086293874	bmse011685	InChI=1S/C13H16N2O3/c1-8-9(5-6-17-8)12(16)14-11-7-10(18-15-11)13(2,3)4/h5-7H,1-4H3,(H,14,15,16)
**662**	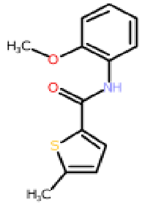	n.a.	717873-31-9	Z27797417	bmse011046	InChI=1S/C13H13NO2S/c1-9-7-8-12(17-9)13(15)14-10-5-3-4-6-11(10)16-2/h3-8H,1-2H3,(H,14,15)
**663**	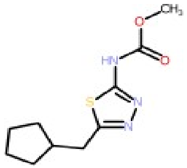	n.a.	717873-65-9	Z1119505742	bmse011355	InChI=1S/C10H15N3O2S/c1-15-10(14)11-9-13-12-8(16-9)6-7-4-2-3-5-7/h7H,2-6H2,1H3,(H,11,13,14)
**664**	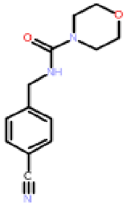	n.a.	720663-80-9	Z509756472	bmse011183	InChI=1S/C13H15N3O2/c14-9-11-1-3-12(4-2-11)10-15-13(17)16-5-7-18-8-6-16/h1-4H,5-8,10H2,(H,15,17)
**665**	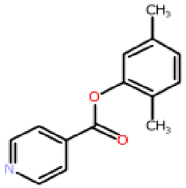	n.a.	720670-03-1	Z30891796	bmse011294	InChI=1S/C14H13NO2/c1-10-3-4-11(2)13(9-10)17-14(16)12-5-7-15-8-6-12/h3-9H,1-2H3

Suppl Table 2: Details of the 768 compounds from the DSI-Poised fragment Library (DSI-PL)

Sl. No.	Structures	No. in Manuscript	CAS Registry Number	"ID"	^{1}H NMR Data Repository BMRB ID	ALATIS InChI
**666**	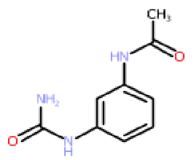	n.a.	720670-20-2	Z198195770	bmse011684	InChI=1S/C9H11N3O2/c1-6(13)11-7-3-2-4-8(5-7)12-9(10)14/h2-5H,1H3,(H,11,13)(H3,10,12,14)
**667**	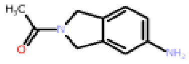	n.a.	722444-62-4	Z1354416068	bmse011394	InChI=1S/C10H12N2O/c1-7(13)12-5-8-2-3-10(11)4-9(8)6-12/h2-4H,5-6,11H2,1H3
**668**	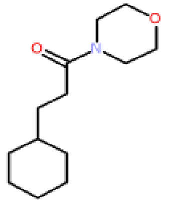	n.a.	72299-30-0	Z31721798	bmse011090	InChI=1S/C13H23NO2/c15-13(14-8-10-16-11-9-14)7-6-12-4-2-1-3-5-12/h12H,1-11H2
**669**	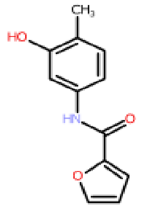	n.a.	723257-51-0	Z1430613384	bmse011307	InChI=1S/C12H11NO3/c1-8-4-5-9(7-10(8)14)13-12(15)11-3-2-6-16-11/h2-7,14H,1H3,(H,13,15)
**670**	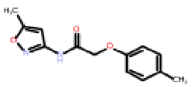	n.a.	723260-99-9	Z19731563	bmse011045	InChI=1S/C13H14N2O3/c1-9-3-5-11(6-4-9)17-8-13(16)14-12-7-10(2)18-15-12/h3-7H,8H2,1-2H3,(H,14,15,16)

Suppl Table 2: Details of the 768 compounds from the DSI-Poised fragment Library (DSI-PL)

Sl. No.	Structures	No. in Manuscript	CAS Registry Number	"ID"	^{1}H NMR Data Repository BMRB ID	ALATIS InChI
**671**	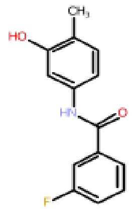	n.a.	723261-32-3	Z1430613393	bmse011309	InChI=1S/C14H12FNO2/c1-9-5-6-12(8-13(9)17)16-14(18)10-3-2-4-11(15)7-10/h2-8,17H,1H3,(H,16,18)
**672**	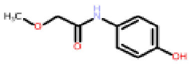	n.a.	723757-41-3	Z943693514	bmse011285	InChI=1S/C9H11NO3/c1-13-6-9(12)10-7-2-4-8(11)5-3-7/h2-5,11H,6H2,1H3,(H,10,12)
**673**	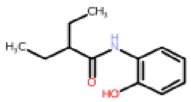	n.a.	723757-74-2	Z1331830630	bmse011728	InChI=1S/C12H17NO2/c1-3-9(4-2)12(15)13-10-7-5-6-8-11(10)14/h5-9,14H,3-4H2,1-2H3,(H,13,15)
**674**	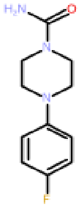	n.a.	724455-68-9	Z198194394	bmse011693	InChI=1S/C11H14FN3O/c12-9-1-3-10(4-2-9)14-5-7-15(8-6-14)11(13)16/h1-4H,5-8H2,(H2,13,16)
**675**	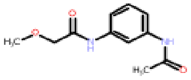	n.a.	724734-86-5	Z31735562	bmse011207	InChI=1S/C11H14N2O3/c1-8(14)12-9-4-3-5-10(6-9)13-11(15)7-16-2/h3-6H,7H2,1-2H3,(H,12,14)(H,13,15)

Suppl Table 2: Details of the 768 compounds from the DSI-Poised fragment Library (DSI-PL)

Sl. No.	Structures	No. in Manuscript	CAS Registry Number	"ID"	^{1}H NMR Data Repository BMRB ID	ALATIS InChI
**676**	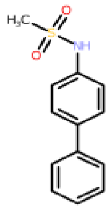	n.a.	724743-56-0	Z45641455	bmse011687	InChI=1S/C13H13NO2S/c1-17(15,16)14-13-9-7-12(8-10-13)11-5-3-2-4-6-11/h2-10,14H,1H3
**677**	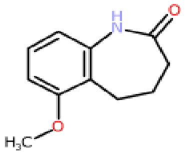	n.a.	72503-43-6	Z1507502062	bmse011409	InChI=1S/C11H13NO2/c1-14-10-6-3-5-9-8(10)4-2-7-11(13)12-9/h3,5-6H,2,4,7H2,1H3,(H,12,13)
**678**	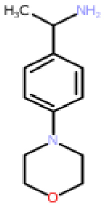	n.a.	728024-36-0	Z1259341012	bmse011266	InChI=1S/C12H18N2O/c1-10(13)11-2-4-12(5-3-11)14-6-8-15-9-7-14/h2-5,10H,6-9,13H2,1H3/t10-/m0/s1
**679**	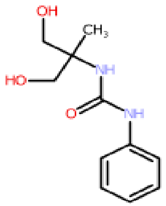	n.a.	74548-62-2	Z57472297	bmse011623	InChI=1S/C11H16N2O3/c1-11(7-14,8-15)13-10(16)12-9-5-3-2-4-6-9/h2-6,14-15H,7-8H2,1H3,(H2,12,13,16)
**680**	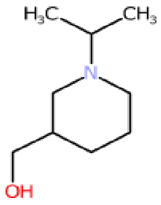	n.a.	752970-45-9	Z103740620	bmse011573	InChI=1S/C9H19NO/c1-8(2)10-5-3-4-9(6-10)7-11/h8-9,11H,3-7H2,1-2H3/t9-/m0/s1

Suppl Table 2: Details of the 768 compounds from the DSI-Poised fragment Library (DSI-PL)

Sl. No.	Structures	No. in Manuscript	CAS Registry Number	"ID"	^{1}H NMR Data Repository BMRB ID	ALATIS InChI
**681**	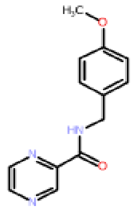	n.a.	75908-56-4	Z30871350	bmse011079	InChI=1S/C13H13N3O2/c1-18-11-4-2-10(3-5-11)8-16-13(17)12-9-14-6-7-15-12/h2-7,9H,8H2,1H3,(H,16,17)
**682**	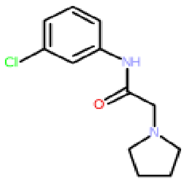	n.a.	75997-68-1	Z2735692823	bmse011011	InChI=1S/C12H15ClN2O/c13-10-4-3-5-11(8-10)14-12(16)9-15-6-1-2-7-15/h3-5,8H,1-2,6-7,9H2,(H,14,16)
**683**	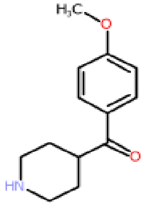	n.a.	76362-12-4	Z1741973467	bmse011018	InChI=1S/C13H17NO2/c1-16-12-4-2-10(3-5-12)13(15)11-6-8-14-9-7-11/h2-5,11,14H,6-9H2,1H3
**684**	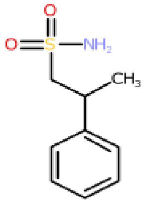	n.a.	76653-16-2	Z1407673036	bmse011400	InChI=1S/C9H13NO2S/c1-8(7-13(10,11)12)9-5-3-2-4-6-9/h2-6,8H,7H2,1H3,(H2,10,11,12)/t8-/m0/s1
**685**	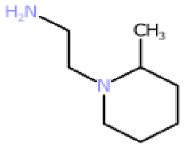	n.a.	768-08-1	Z1245580425	bmse011606	InChI=1S/C8H18N2/c1-8-4-2-3-6-10(8)7-5-9/h8H,2-7,9H2,1H3/t8-/m0/s1

Suppl Table 2: Details of the 768 compounds from the DSI-Poised fragment Library (DSI-PL)

Sl. No.	Structures	No. in Manuscript	CAS Registry Number	"ID"	^{1}H NMR Data Repository BMRB ID	ALATIS InChI
**686**	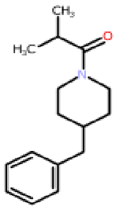	n.a.	77251-48-0	Z30162334	bmse011660	InChI=1S/C16H23NO/c1-13(2)16(18)17-10-8-15(9-11-17)12-14-6-4-3-5-7-14/h3-7,13,15H,8-12H2,1-2H3
**687**	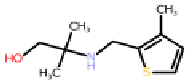	n.a.	774193-68-9	Z2856434821	bmse011700	InChI=1S/C10H17NOS/c1-8-4-5-13-9(8)6-11-10(2,3)7-12/h4-5,11-12H,6-7H2,1-3H3
**688**	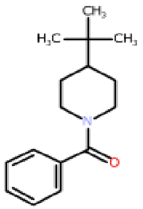	n.a.	787-50-8	Z1217741507	bmse011654	InChI=1S/C16H23NO/c1-16(2,3)14-9-11-17(12-10-14)15(18)13-7-5-4-6-8-13/h4-8,14H,9-12H2,1-3H3
**689**	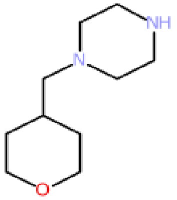	n.a.	787518-60-9	Z1267773591	bmse011553	InChI=1S/C10H20N2O/c1-7-13-8-2-10(1)9-12-5-3-11-4-6-12/h10-11H,1-9H2
**690**	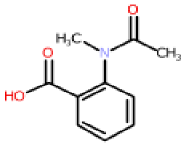	n.a.	78944-67-9	Z223688272	bmse011456	InChI=1S/C10H11NO3/c1-7(12)11(2)9-6-4-3-5-8(9)10(13)14/h3-6H,1-2H3,(H,13,14)

Suppl Table 2: Details of the 768 compounds from the DSI-Poised fragment Library (DSI-PL)

Sl. No.	Structures	No. in Manuscript	CAS Registry Number	"ID"	^{1}H NMR Data Repository BMRB ID	ALATIS InChI
**691**	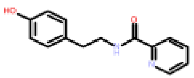	n.a.	791805-78-2	Z730649594	bmse011669	InChI=1S/C14H14N2O2/c17-12-6-4-11(5-7-12)8-10-16-14(18)13-3-1-2-9-15-13/h1-7,9,17H,8,10H2,(H,16,18)
**692**	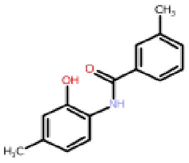	n.a.	791831-63-5	Z33452549	bmse011029	InChI=1S/C15H15NO2/c1-10-4-3-5-12(8-10)15(18)16-13-7-6-11(2)9-14(13)17/h3-9,17H,1-2H3,(H,16,18)
**693**	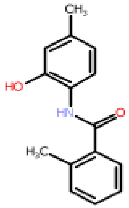	n.a.	791840-46-5	Z33452282	bmse011069	InChI=1S/C15H15NO2/c1-10-7-8-13(14(17)9-10)16-15(18)12-6-4-3-5-11(12)2/h3-9,17H,1-2H3,(H,16,18)
**694**	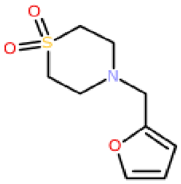	n.a.	79206-94-3	Z2856434926	bmse011640	InChI=1S/C9H13NO3S/c11-14(12)6-3-10(4-7-14)8-9-2-1-5-13-9/h1-2,5H,3-4,6-8H2
**695**	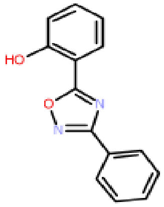	n.a.	79349-24-9	Z276351322	n.a.	n.a.

Suppl Table 2: Details of the 768 compounds from the DSI-Poised fragment Library (DSI-PL)

Sl. No.	Structures	No. in Manuscript	CAS Registry Number	"ID"	^{1}H NMR Data Repository BMRB ID	ALATIS InChI
**696**	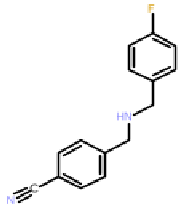	n.a.	797769-58-5	Z2856434851	bmse011653	InChI=1S/C15H13FN2/c16-15-7-5-14(6-8-15)11-18-10-13-3-1-12(9-17)2-4-13/h1-8,18H,10-11H2
**697**	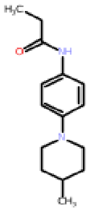	n.a.	797777-96-9	Z57344995	bmse011364	InChI=1S/C15H22N2O/c1-3-15(18)16-13-4-6-14(7-5-13)17-10-8-12(2)9-11-17/h4-7,12H,3,8-11H2,1-2H3,(H,16,18)
**698**	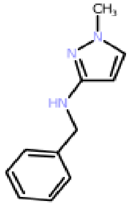	n.a.	81574-05-2	Z1401333862	bmse011526	InChI=1S/C11H13N3/c1-14-8-7-11(13-14)12-9-10-5-3-2-4-6-10/h2-8H,9H2,1H3,(H,12,13)
**699**	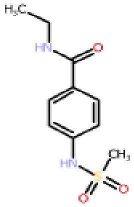	n.a.	824974-96-1	Z57614330	bmse011729	InChI=1S/C10H14N2O3S/c1-3-11-10(13)8-4-6-9(7-5-8)12-16(2,14)15/h4 7,12H,3H2,1-2H3,(H,11,13)
**700**	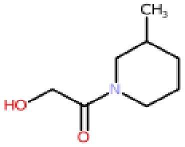	n.a.	82554-10-7	Z228585534	bmse011566	InChI=1S/C8H15NO2/c1-7-3-2-4-9(5- 7)8(11)6-10/h7,10H,2-6H2,1H3/t7-/m0/s1

Suppl Table 2: Details of the 768 compounds from the DSI-Poised fragment Library (DSI-PL)

Sl. No.	Structures	No. in Manuscript	CAS Registry Number	"ID"	^{1}H NMR Data Repository BMRB ID	ALATIS InChI
**701**	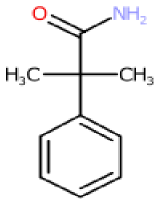	n.a.	826-54-0	Z270979606	bmse011161	InChI=1S/C10H13NO/c1-10(2,9(11)12)8-6-4-3-5-7-8/h3-7H,1-2H3,(H2,11,12)
**702**	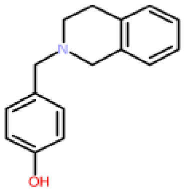	n.a.	82965-56-8	Z2856434812	bmse011625	InChI=1S/C16H17NO/c18-16-7-5-13(6-8-16)11-17-10-9-14-3-1-2-4-15(14)12-17/h1-8,18H,9-12H2
**703**	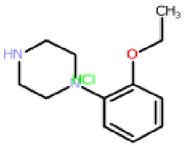	n.a.	83081-75-8	Z425387594	bmse011092	InChI=1S/C12H18N2O.ClH/c1-2-15-12-6-4-3-5-11(12)14-9-7-13-8-10-14;/h3-6,13H,2,7-10H2,1H3;1H
**704**	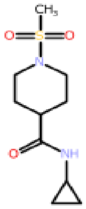	n.a.	832119-05-8	Z227998000	bmse011354	InChI=1S/C10H18N2O3S/c1-16(14,15)12-6-4-8(5-7-12)10(13)11-9-2-3-9/h8-9H,2-7H2,1H3,(H,11,13)
**705**	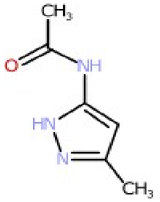	n.a.	83725-05-7	Z239136710	bmse011171	InChI=1S/C6H9N3O/c1-4-3-6(9-8-4)7-5(2)10/h3H,1-2H3,(H2,7,8,9,10)

Suppl Table 2: Details of the 768 compounds from the DSI-Poised fragment Library (DSI-PL)

Sl. No.	Structures	No. in Manuscript	CAS Registry Number	"ID"	^{1}H NMR Data Repository BMRB ID	ALATIS InChI
**706**	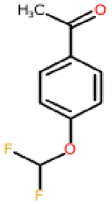	n.a.	83882-67-1	Z55671900	bmse011529	InChI=1S/C9H8F2O2/c1-6(12)7-2-4-8(5-3-7)13-9(10)11/h2-5,9H,1H3
**707**	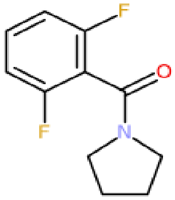	n.a.	84044-77-9	Z54226006	bmse011231	InChI=1S/C11H11F2NO/c12-8-4-3-5-9(13)10(8)11(15)14-6-1-2-7-14/h3-5H,1-2,6-7H2
**708**	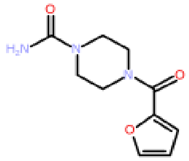	n.a.	84655-78-7	Z198194396	bmse011690	InChI=1S/C10H13N3O3/c11-10(15)13-5-3-12(4-6-13)9(14)8-2-1-7-16-8/h1-2,7H,3-6H2,(H2,11,15)
**709**	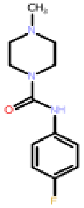	n.a.	84882-87-1	Z2856434944	bmse011056	InChI=1S/C12H16FN3O/c1-15-6-8-16(9-7-15)12(17)14-11-4-2-10(13)3-5-11/h2-5H,6-9H2,1H3,(H,14,17)
**710**	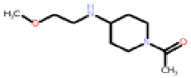	n.a.	849021-41-6	Z2856434813	bmse011648	InChI=1S/C10H20N2O2/c1-9(13)12-6-3-10(4-7-12)11-5-8-14-2/h10-11H,3-8H2,1-2H3

Suppl Table 2: Details of the 768 compounds from the DSI-Poised fragment Library (DSI-PL)

Sl. No.	Structures	No. in Manuscript	CAS Registry Number	"ID"	^{1}H NMR Data Repository BMRB ID	ALATIS InChI
**711**	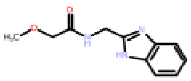	n.a.	850921-03-8	Z111529496	bmse011227	InChI=1S/C11H13N3O2/c1-16-7-11(15)12-6-10-13-8-4-2-3-5-9(8)14-10/h2-5H,6-7H2,1H3,(H,12,15)(H,13,14)
**712**	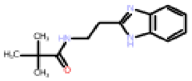	n.a.	850923-38-5	Z111716368	bmse011010	InChI=1S/C14H19N3O/c1-14(2,3)13(18)15-9-8-12-16-10-6-4-5-7-11(10)17-12/h4-7H,8-9H2,1-3H3,(H,15,18)(H,16,17)
**713**	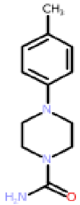	n.a.	85474-81-3	Z1650868495	bmse011332	InChI=1S/C12H17N3O/c1-10-2-4-11(5-3-10)14-6-8-15(9-7-14)12(13)16/h2-5H,6-9H2,1H3,(H2,13,16)
**714**	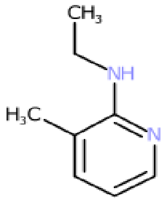	n.a.	856836-17-4	Z1992316287	bmse011564	InChI=1S/C8H12N2/c1-3-9-8-7(2)5-4-6-10-8/h4-6H,3H2,1-2H3,(H,9,10)
**715**	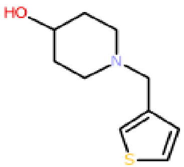	n.a.	864388-84-1	Z2856434899	bmse011274	InChI=1S/C10H15NOS/c12-10-1-4-11(5-2-10)7-9-3-6-13-8-9/h3,6,8,10,12H,1-2,4-5,7H2

Suppl Table 2: Details of the 768 compounds from the DSI-Poised fragment Library (DSI-PL)

Sl. No.	Structures	No. in Manuscript	CAS Registry Number	"ID"	^{1}H NMR Data Repository BMRB ID	ALATIS InChI
**716**	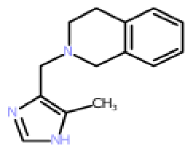	n.a.	864416-90-0	Z2856434879	bmse011705	InChI=1S/C14H17N3/c1-11-14(16-10-15-11)9-17-7-6-12-4-2-3-5-13(12)8-17/h2-5,10H,6-9H2,1H3,(H,15,16)
**717**	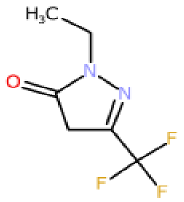	n.a.	866472-53-9	Z1268152398	bmse011038	InChI=1S/C6H7F3N2O/c1-2-11-5(12)3-4(10-11)6(7,8)9/h2-3H2,1H3
**718**	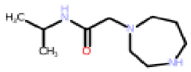	n.a.	87055-39-8	Z1262254278	bmse011603	InChI=1S/C10H21N3O/c1-9(2)12-10(14)8-13-6-3-4-11-5-7-13/h9,11H,3-8H2,1-2H3,(H,12,14)
**719**	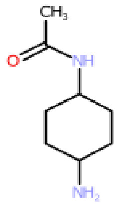	n.a.	873537-23-6	Z1787158625	bmse011462	InChI=1S/C8H16N2O/c1-6(11)10-8-4-2-7(9)3-5-8/h7-8H,2-5,9H2,1H3,(H,10,11)/t7-,8-
**720**	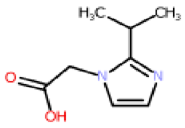	n.a.	876717-08-7	Z2446040567	bmse011162	InChI=1S/C8H12N2O2/c1-6(2)8-9-3-4-10(8)5-7(11)12/h3-4,6H,5H2,1-2H3,(H,11,12)

Suppl Table 2: Details of the 768 compounds from the DSI-Poised fragment Library (DSI-PL)

Sl. No.	Structures	No. in Manuscript	CAS Registry Number	"ID"	^{1}H NMR Data Repository BMRB ID	ALATIS InChI
**721**	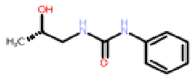	n.a.	87919-24-2	Z1563512128	bmse011369	InChI=1S/C10H14N2O2/c1-8(13)7-11-10(14)12-9-5-3-2-4-6-9/h2-6,8,13H,7H2,1H3,(H2,11,12,14)/t8-/m0/s1
**722**	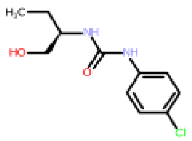	n.a.	87919-30-0	Z1593306637	bmse011370	InChI=1S/C11H15ClN2O2/c1-2-9(7-15)13-11(16)14-10-5-3-8(12)4-6-10/h3-6,9,15H,2,7H2,1H3,(H2,13,14,16)/t9-/m1/s1
**723**	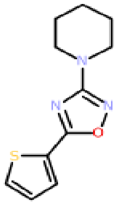	n.a.	883013-61-4	Z1623890017	bmse011646	InChI=1S/C11H13N3OS/c1-2-6-14(7-3-1)11-12-10(15-13-11)9-5-4-8-16-9/h4-5,8H,1-3,6-7H2
**724**	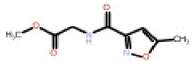	n.a.	883043-92-3	Z375990520	bmse011649	InChI=1S/C8H10N2O4/c1-5-3-6(10-14-5)8(12)9-4-7(11)13-2/h3H,4H2,1-2H3,(H,9,12)
**725**	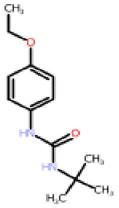	n.a.	883095-34-9	Z44586802	bmse011343	InChI=1S/C13H20N2O2/c1-5-17-11-8-6-10(7-9-11)14-12(16)15-13(2,3)4/h6-9H,5H2,1-4H3,(H2,14,15,16)

Suppl Table 2: Details of the 768 compounds from the DSI-Poised fragment Library (DSI-PL)

Sl. No.	Structures	No. in Manuscript	CAS Registry Number	"ID"	1H NMR Data Repository BMRB ID	ALATIS InChI
**726**	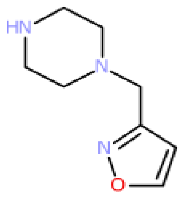	n.a.	885952-54-5	Z1263714198	bmse011610	InChI=1S/C8H13N3O/c1-6-12-10-8(1)7-11-4-2-9-3-5-11/h1,6,9H,2-5,7H2
**727**	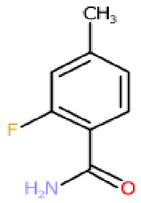	n.a.	886761-61-1	Z1343633025	bmse011399	InChI=1S/C8H8FNO/c1-5-2-3-6(8(10)11)7(9)4-5/h2-4H,1H3,(H2,10,11)
**728**	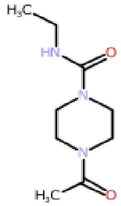	n.a.	887577-54-0	Z44590919	bmse011174	InChI=1S/C9H17N3O2/c1-3-10-9(14)12-6-4-11(5-7-12)8(2)13/h3-7H2,1-2H3,(H,10,14)
**729**	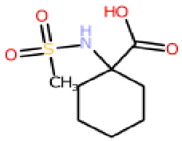	n.a.	887842-05-9	Z133622412	bmse011197	InChI=1S/C8H15NO4S/c1-14(12,13)9-8(7(10)11)5-3-2-4-6-8/h9H,2-6H2,1H3,(H,10,11)
**730**	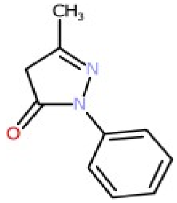	n.a.	89-25-8	Z50145861	bmse011258	InChI=1S/C10H10N2O/c1-8-7-10(13)12(11-8)9-5-3-2-4-6-9/h2-6H,7H2,1H3

Suppl Table 2: Details of the 768 compounds from the DSI-Poised fragment Library (DSI-PL)

Sl. No.	Structures	No. in Manuscript	CAS Registry Number	"ID"	1H NMR Data Repository BMRB ID	ALATIS InChI
**731**	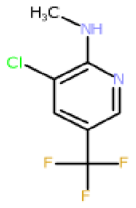	n.a.	89810-01-5	Z54628157	bmse011747	InChI=1S/C7H6ClF3N2/c1-12-6-5(8)2-4(3-13-6)7(9,10)11/h2-3H,1H3,(H,12,13)
**732**	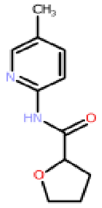	n.a.	899020-94-1	Z71580604	bmse011530	InChI=1S/C11H14N2O2/c1-8-4-5-10(12-7-8)13-11(14)9-3-2-6-15-9/h4-5,7,9H,2-3,6H2,1H3,(H,12,13,14)/t9-/m1/s1
**733**	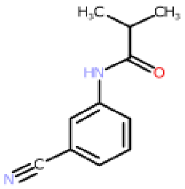	n.a.	900710-59-0	Z26548083	bmse011019	InChI=1S/C11H12N2O/c1-8(2)11(14)13-10-5-3-4-9(6-10)7-12/h3-6,8H,1-2H3,(H,13,14)
**734**	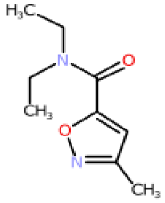	n.a.	902623-58-9	Z135439900	bmse011595	InChI=1S/C9H14N2O2/c1-4-11(5-2)9(12)8-6-7(3)10-13-8/h6H,4-5H2,1-3H3
**735**	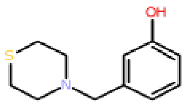	n.a.	90287-65-3	Z2856434857	bmse011707	InChI=1S/C11H15NOS/c13-11-3-1-2-10(8-11)9-12-4-6-14-7-5-12/h1-3,8,13H,4-7,9H2

Suppl Table 2: Details of the 768 compounds from the DSI-Poised fragment Library (DSI-PL)

Sl. No.	Structures	No. in Manuscript	CAS Registry Number	"ID"	1H NMR Data Repository BMRB ID	ALATIS InChI
**736**	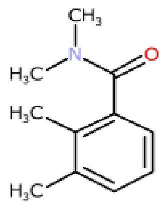	n.a.	90525-99-8	Z32014663	bmse011593	InChI=1S/C11H15NO/c1-8-6-5-7-10(9(8)2)11(13)12(3)4/h5-7H,1-4H3
**737**	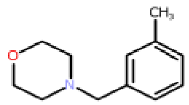	n.a.	90754-64-6	Z2856434862	bmse011571	InChI=1S/C12H17NO/c1-11-3-2-4-12(9-11)10-13-5-7-14-8-6-13/h2-4,9H,5-8,10H2,1H3
**738**	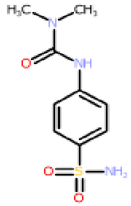	n.a.	91328-92-6	Z123856654	bmse011027	InChI=1S/C9H13N3O3S/c1-12(2)9(13)11-7-3-5-8(6-4-7)16(10,14)15/h3-6H,1-2H3,(H,11,13)(H2,10,14,15)
**739**	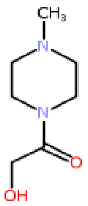	n.a.	91406-27-8	Z228585842	bmse011280	InChI=1S/C7H14N2O2/c1-8-2-4-9(5-3-8)7(11)6-10/h10H,2-6H2,1H3
**740**	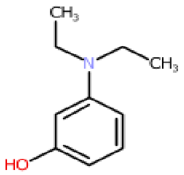	n.a.	91-68-9	Z2856434814	bmse011268	InChI=1S/C10H15NO/c1-3-11(4-2)9-6-5-7-10(12)8-9/h5-8,12H,3-4H2,1-2H3

Suppl Table 2: Details of the 768 compounds from the DSI-Poised fragment Library (DSI-PL)

Sl. No.	Structures	No. in Manuscript	CAS Registry Number	"ID"	^{1}H NMR Data Repository BMRB ID	ALATIS InChI
**741**	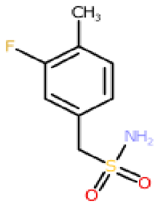	n.a.	919354-68-0	Z1703168683	bmse011098	InChI=1S/C8H10FNO2S/c1-6-2-3-7(4-8(6)9)5-13(10,11)12/h2-4H,5H2,1H3,(H2,10,11,12)
**742**	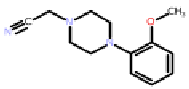	n.a.	92043-13-5	Z2856434807	bmse011314	InChI=1S/C13H17N3O/c1-17-13-5-3-2-4-12(13)16-10-8-15(7-6-14)9-11-16/h2-5H,7-11H2,1H3
**743**	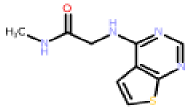	n.a.	923187-09-1	Z52584368	bmse011105	InChI=1S/C9H10N4OS/c1-10-7(14)4-11-8-6-2-3-15-9(6)13-5-12-8/h2-3,5H,4H2,1H3,(H,10,14)(H,11,12,13)
**744**	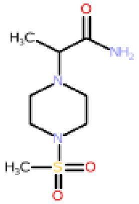	n.a.	923681-46-3	Z134785326	bmse011209	InChI=1S/C8H17N3O3S/c1-7(8(9)12)10-3-5-11(6-4-10)15(2,13)14/h7H,3-6H2,1-2H3,(H2,9,12)/t7-/m1/s1
**745**	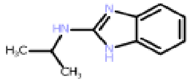	n.a.	924872-04-8	Z2467208649	bmse011588	InChI=1S/C10H13N3/c1-7(2)11-10-12-8-5-3-4-6-9(8)13-10/h3-7H,1-2H3,(H2,11,12,13)

Suppl Table 2: Details of the 768 compounds from the DSI-Poised fragment Library (DSI-PL)

Sl. No.	Structures	No. in Manuscript	CAS Registry Number	"ID"	^{1}H NMR Data Repository BMRB ID	ALATIS InChI
**746**	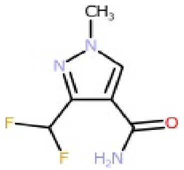	n.a.	925689-10-7	Z1515654336	bmse011424	InChI=1S/C6H7F2N3O/c1-11-2-3(6(9)12)4(10-11)5(7)8/h2,5H,1H3,(H2,9,12)
**747**	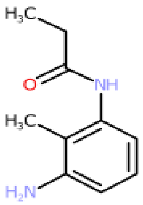	n.a.	926235-23-6	Z235361315	bmse011459	InChI=1S/C10H14N2O/c1-3-10(13)12-9-6-4-5-8(11)7(9)2/h4-6H,3,11H2,1-2H3,(H,12,13)
**748**	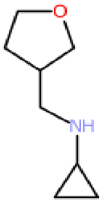	n.a.	926239-80-7	Z818727262	bmse011600	InChI=1S/C8H15NO/c1-2-8(1)9-5-7-3-4-10-6-7/h7-9H,1-6H2/t7-/m1/s1
**749**	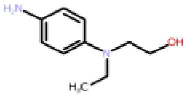	n.a.	92-65-9	Z752989138	bmse011495	InChI=1S/C10H16N2O/c1-2-12(7-8-13)10-5-3-9(11)4-6-10/h3-6,13H,2,7-8,11H2,1H3
**750**	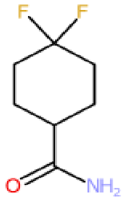	n.a.	927209-98-1	Z1526748660	bmse011153	InChI=1S/C7H11F2NO/c8-7(9)3-1-5(2-4-7)6(10)11/h5H,1-4H2,(H2,10,11)

Suppl Table 2: Details of the 768 compounds from the DSI-Poised fragment Library (DSI-PL)

Sl. No.	Structures	No. in Manuscript	CAS Registry Number	"ID"	1H NMR Data Repository BMRB ID	ALATIS InChI
**751**	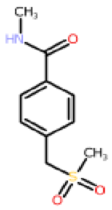	n.a.	930939-62-1	Z30272045	bmse011049	InChI=1S/C10H13NO3S/c1-11-10(12)9-5-3-8(4-6-9)7-15(2,13)14/h3-6H,7H2,1-2H3,(H,11,12)
**752**	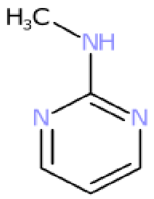	n.a.	931-61-3	Z54628578	bmse011193	InChI=1S/C5H7N3/c1-6-5-7-3-2-4-8-5/h2-4H,1H3,(H,6,7,8)
**753**	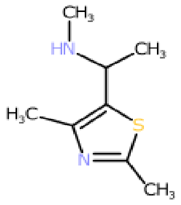	n.a.	933718-01-5	Z1266823232	bmse011757	InChI=1S/C8H14N2S/c1-5(9-4)8-6(2)10-7(3)11-8/h5,9H,1-4H3/t5-/m0/s1
**754**	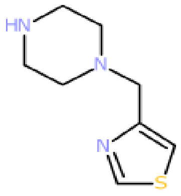	n.a.	933737-25-8	Z812517112	bmse011466	InChI=1S/C8H13N3S/c1-3-11(4-2-9-1)5-8-6-12-7-10-8/h6-7,9H,1-5H2
**755**	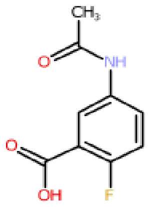	n.a.	939909-22-5	Z364321922	bmse011262	InChI=1S/C9H8FNO3/c1-5(12)11-6-2-3-8(10)7(4-6)9(13)14/h2-4H,1H3,(H,11,12)(H,13,14)

Suppl Table 2: Details of the 768 compounds from the DSI-Poised fragment Library (DSI-PL)

Sl. No.	Structures	No. in Manuscript	CAS Registry Number	"ID"	1H NMR Data Repository BMRB ID	ALATIS InChI
**756**	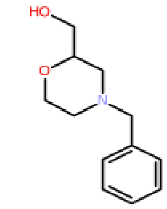	n.a.	943442-96-4	Z1259086950	bmse011736	InChI=1S/C12H17NO2/c14-10-12-9-13(6-7-15-12)8-11-4-2-1-3-5-11/h1-5,12,14H,6-10H2/t12-/m0/s1
**757**	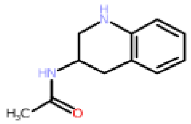	n.a.	945-80-2	Z1492796719	bmse011407	InChI=1S/C11H14N2O/c1-8(14)13-10-6-9-4-2-3-5-11(9)12-7-10/h2-5,10,12H,6-7H2,1H3,(H,13,14)/t10-/m0/s1
**758**	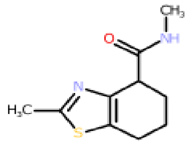	n.a.	95203-31-9	Z1904249627	bmse011121	InChI=1S/C10H14N2OS/c1-6-12-9-7(10(13)11-2)4-3-5-8(9)14-6/h7H,3-5H2,1-2H3,(H,11,13)/t7-/m1/s1
**759**	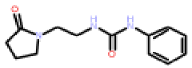	n.a.	95356-60-8	Z2472938267	bmse011184	InChI=1S/C13H17N3O2/c17-12-7-4-9-16(12)10-8-14-13(18)15-11-5-2-1-3-6-11/h1-3,5-6H,4,7-10H2,(H2,14,15,18)
**760**	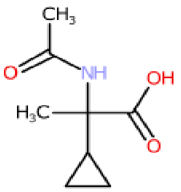	n.a.	95525-82-9	Z1259162160	bmse011264	InChI=1S/C8H13NO3/c1-5(10)9-8(2,7(11)12)6-3-4-6/h6H,3-4H2,1-2H3,(H,9,10)(H,11,12)/t8-/m1/s1

Suppl Table 2: Details of the 768 compounds from the DSI-Poised fragment Library (DSI-PL)

Sl. No.	Structures	No. in Manuscript	CAS Registry Number	"ID"	^{1}H NMR Data Repository BMRB ID	ALATIS InChI
**761**	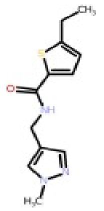	n.a.	955901-04-9	Z804566442	bmse011627	InChI=1S/C12H15N3OS/c1-3-10-4-5-11(17-10)12(16)13-6-9-7-14-15(2)8-9/h4-5,7-8H,3,6H2,1-2H3,(H,13,16)
**762**	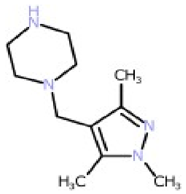	n.a.	957514-00-0	Z1263820300	bmse011279	InChI=1S/C11H20N4/c1-9-11(10(2)14(3)13-9)8-15-6-4-12-5-7-15/h12H,4-8H2,1-3H3
**763**	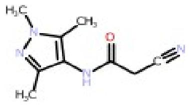	n.a.	957942-27-7	Z1688504114	bmse011676	InChI=1S/C9H12N4O/c1-6-9(7(2)13(3)12-6)11-8(14)4-5-10/h4H2,1-3H3,(H,11,14)
**764**	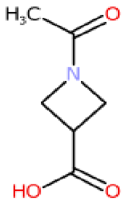	n.a.	97628-91-6	Z1868430535	bmse011467	InChI=1S/C6H9NO3/c1-4(8)7-2-5(3-7)6(9)10/h5H,2-3H2,1H3,(H,9,10)
**765**	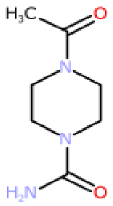	n.a.	98337-79-2	Z90122368	bmse011021	InChI=1S/C7H13N3O2/c1-6(11)9-2-4-10(5-3-9)7(8)12/h2-5H2,1H3,(H2,8,12)

Suppl Table 2: Details of the 768 compounds from the DSI-Poised fragment Library (DSI-PL)

Sl. No.	Structures	No. in Manuscript	CAS Registry Number	"ID"	^{1}H NMR Data Repository BMRB ID	ALATIS InChI
**766**	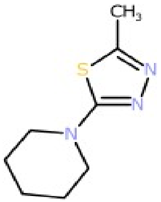	n.a.	98427-81-7	Z1251207602	bmse011513	InChI=1S/C8H13N3S/c1-7-9-10-8(12-7)11-5-3-2-4-6-11/h2-6H2,1H3
**767**	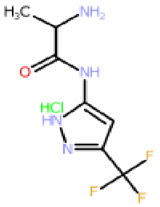	n.a.	n.a.	Z1622626423	bmse011103	InChI=1S/C7H9F3N4O.ClH/c1-3(11)6(15)12-5-2-4(13-14-5)7(8,9)10;/h2-3H,11H2,1H3,(H2,12,13,14,15);1H/t3-;/m0./s1
**768**	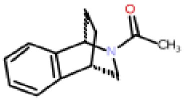	n.a.	n.a.	Z2017861827	bmse011133	InChI=1S/C13H15NO/c1-9(15)14-8-10-6-7-13(14)12-5-3-2-4-11(10)12/h2-5,10,13H,6-8H2,1H3/t10-,13-/m1/s1
